# Assessment and Reconstruction of Novel HSP90 Genes: Duplications, Gains and Losses in Fungal and Animal Lineages

**DOI:** 10.1371/journal.pone.0073217

**Published:** 2013-09-16

**Authors:** Chrysoula N. Pantzartzi, Elena Drosopoulou, Zacharias G. Scouras

**Affiliations:** 1 Department of Genetics, Development and Molecular Biology, School of Biology, Faculty of Sciences, Aristotle University of Thessaloniki, Thessaloniki, Greece; 2 Department of Transcriptional Regulation, Institute of Molecular Genetics, Prague, Czech Republic; Université Claude Bernard – Lyon 1, France

## Abstract

Hsp90s, members of the Heat Shock Protein class, protect the structure and function of proteins and play a significant task in cellular homeostasis and signal transduction. In order to determine the number of *hsp90* gene copies and encoded proteins in fungal and animal lineages and through that key duplication events that this family has undergone, we collected and evaluated Hsp90 protein sequences and corresponding Expressed Sequence Tags and analyzed available genomes from various taxa. We provide evidence for duplication events affecting either single species or wider taxonomic groups. With regard to Fungi, duplicated genes have been detected in several lineages. In invertebrates, we demonstrate key duplication events in certain clades of Arthropoda and Mollusca, and a possible gene loss event in a hymenopteran family. Finally, we infer that the duplication event responsible for the two (a and b) isoforms in vertebrates occurred probably shortly after the split of Hyperoartia and Gnathostomata.

## Introduction

Heat Shock Proteins (HSPs) facilitate protein folding and guard the proteome from the dangers of misfolding and aggregation [1]. They are expressed as responses to adverse environmental or chemical stresses, such as heat or cold shock, hypoxia, salinity, heavy metals and pathophysiological situations and play important role in cell survival [2], [3].

Hsp90s account for 1–2% of all cellular proteins in most cells under non-stress conditions. Their function is dependent on the interaction with many co-chaperones [4]. They either prevent aggregation of newly synthesized or misfolded proteins, assisting in their proper folding, or direct them for proteasomal degradation [5], [6]. Their client proteins are involved in signal transduction, transcription and apoptosis [7]–[9]. In recent years, many studies have focused on the role of this family in cancer [10], [11].

HSP90s are essential for viability under all conditions in eukaryotes; in contrast, deletion of the bacterial HtpG (High temperature protein G) is not lethal [12], [13]. Hsp90s are found in all organisms, except Archaea [14], and are highly conserved, thus providing an excellent model for evolutionary studies.

Results from previous analyses in eukaryotes indicate that members of the Hsp90 gene family have undergone major duplication events, which led to isoforms with cellular compartmentalization, namely cytoplasmic, endoplasmic, mitochondrial and chloroplastic forms [15]–[18]. In all vertebrates studied so far, there are two known cytoplasmic isoforms, namely inducible (a) and cognate (b) (or AA and AB, respectively, according to [19]) which are considered the result of a duplication event that occurred within the vertebrate lineage [15], [20], [21]. Several additional duplication events at different lineages seem to have resulted in the variable number of total cytoplasmic gene copies observed among vertebrate species [18]. In human, for example, 13 cytoplasmic genes have been identified, 9 of which are pseudogenes [19]. In invertebrates, the numbers of cytoplasmic gene copies and encoded proteins are not uniform. There exist some invertebrate species in which a single gene encodes for a unique cytoplasmic Hsp90 (e.g. nematodes and *Drosophila*) [22]–[25]. Two gene copies seem to encode for a unique cytoplasmic homolog in *Anopheles albimanus* (Diptera) and *Mytilus galloprovincialis* (Mollusca) [26], [27], while two cytoplasmic Hsp90s with tissue-specific expression patterns and differing roles in physiological and stressful conditions have been identified in the crab *Portunus trituberculatus* (Crustacea) [28]. In Fungi, single cytoplasmic genes have been reported [29]–[31] with the exception of *Saccharomyces cerevisiae*, which expresses an inducible and a cognate isoform [32]–[34].

Whole-genome duplication (WGD) and small-scale duplications (SSD) are considered important evolutionary mechanisms [35]. Some of the models (reviewed in [36]) developed in order to explain the retention of both genes following gene duplication, include the evolution of a new function in one of the duplicates, the division of ancestral functions among duplicates and the retention of all functions in both duplicates. The rate of retention of duplicates varies after a WGD or a SSD, depending on the gene functional or developmental specialization. For example, for stress response genes, higher duplicate retention has been noted after SSD [35].

Numerous studies have focused on the identification and expression patterns [28], [37]–[40], as well as on the phylogenetic relationships across the HSP90 family members [15], [17], [18], [41]–[43]. Development of tools and accumulation of genome-wide information could facilitate the elucidation of distribution patterns and evolutionary relationships of HSP90 family members. In the present study, we aimed at determining the number of extant HSP90 cytoplasmic family members in fungal and animal lineages and describe the minimal history of their putative duplication events. We collected Hsp90 sequences available in UniProtKB [44] and the NCBI Protein database [45] and we enriched this dataset with newly identified *hsp90* genes and their predicted protein sequences, according to complete genomes as well as Expressed Sequence Tags (ESTs).

## Methods

### Protein sequences retrieval

Fungal and Metazoan sequences belonging to the HSP90 family, bearing the consensus signature of the family, were retrieved from PROSITE [46] and UniProtKB [44]. There are a total of 3,668 Hsp90 sequences in PROSITE Release 20.85 (27-September-2012), 170 of which originate from Fungi and 655 from Metazoa. Hsp90 protein sequences were also collected through BLASTP searches against the NCBI Protein database [45], using the *Mytilus galloprovincialis* MgHsp90 (UniProtKB AC CAJ85741) and the human cytoplasmic isoforms (AC P07900 and P08238 for a and b isoforms, respectively) as queries. Complete or nearly complete cytoplasmic sequences (>630aa), ending with the characteristic carboxy-terminal motif MEEVD [15], were further analyzed at the level of either Phylum (e.g. Chordata) or Kingdom (e.g. Fungi). We wanted to elaborate on previously reported gene duplications and document other possible duplication events in the same lineages, not described to date. Therefore, we focused on phyla/kingdoms for which different representatives both with single and multiple copies have been described. Besides Arthropoda, Mollusca and Chordata, data concerning the rest of Metazoa phyla were either absent or consisted of partial sequences or single sequences per taxon, thus they were omitted from further analysis.

### Whole-genome analyses

Available genomes analyzed in the present study were retrieved from the FlyBase [47], AphidBase [48], VectorBase [49], Ensembl [50] and GenBank (WGS division, [51]) databases as well as from the JGI [52], the OIST Marine Genomics Unit, the Broad Institute of Harvard and MIT, the Elephant shark genome sequencing Project and the FUGU Genome Project websites. In order to determine the *hsp90* gene copy number of each species, BLAST [53] searches were performed against the corresponding genomes using as queries known Hsp90 sequences of the same or closely related species (accepted E-value was zero). GENSCAN [54] and BLASTX [53] were used for the prediction of putative coding sequences (cds), SpliceView [55] was used for the prediction of possible splicing sites, while predicted coding sequences were translated with Transeq [56].

### ESTs (Expressed Sequenced Tags) retrieval and analysis

For various taxa or taxonomic groups (e.g. Chondricthyes), only few available genomes, but no Hsp90 sequences were available in public databases. In order to include representatives from these groups, we performed BLASTN and TBLASTN searches, in order to retrieve ESTs from GenBank (ESTs division, [51]), that exhibit homology to known *hsp90* sequences (accepted E-value was zero or very close to zero). The MgHsp90 and the human cytoplasmic isoforms were used as queries, for molluscan and chordate ESTs respectively. Wise2 (EMBOSS, [57]), GENSCAN [54], ORFinder (NCBI) and BLAST [53] were used for the assembly of putative amino-acid sequences from collected ESTs. ESTs were not used as a confirmation of the functionality of genes.

### Alignments and tree construction

**Figure 1:**
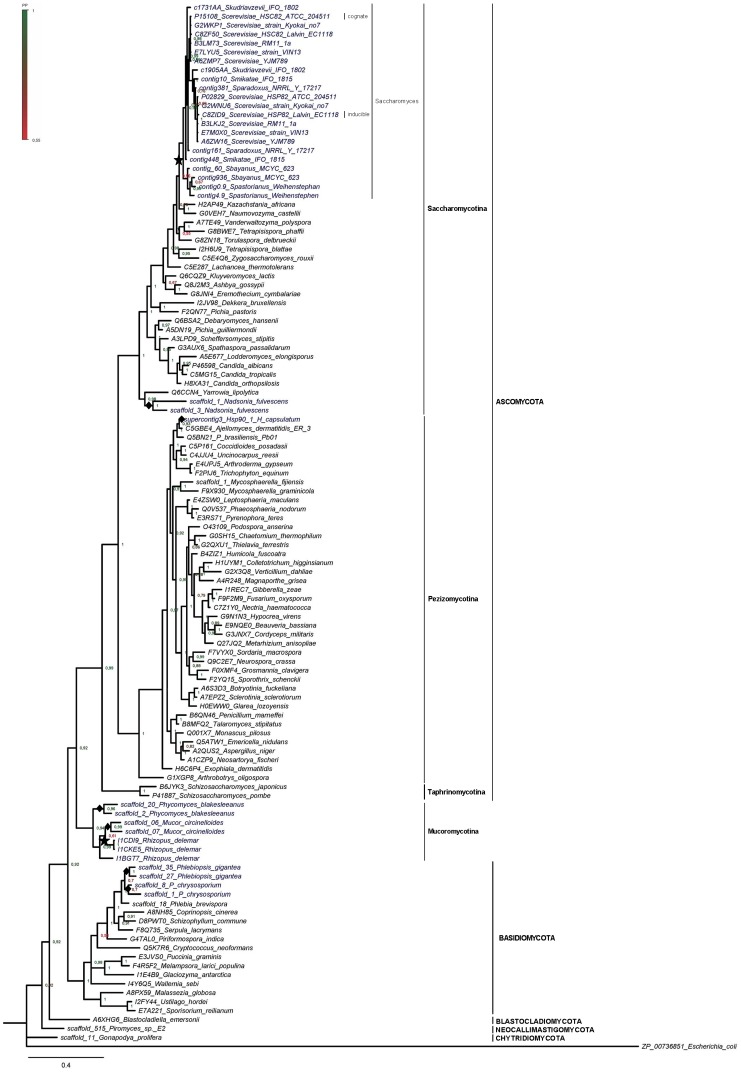
Bayesian inference phylogenetic tree based on Hsp90 protein sequences from Fungi. Species in which multiple *hsp90* genes have been detected are in dark blue. Filled diamonds denote putative species-specific duplication events, predicted by this study. Stars represent whole-genome duplications reported by previous studies. Numbers at nodes represent Posterior Probability (PP) values. Scale bar: substitutions/site.

**Figure 2:**
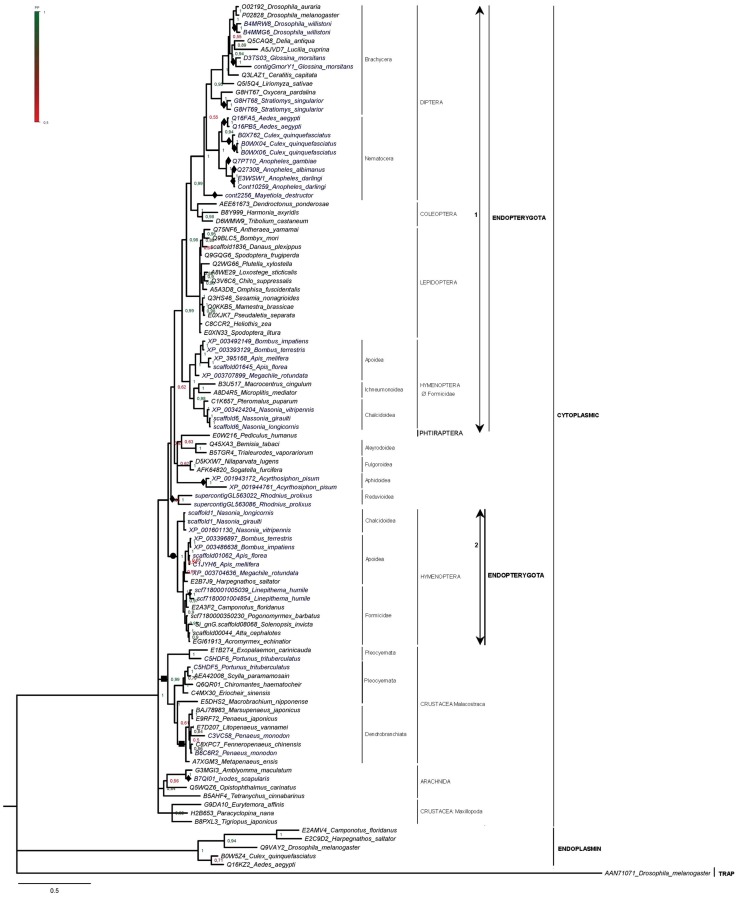
Bayesian inference phylogenetic tree based on Hsp90 protein sequences from Arthropoda. Species in which multiple *hsp90* genes have been detected are in dark blue. Filled diamonds denote putative species-specific duplication events predicted by this study. Filled squares denote duplication events in the common ancestor of a wide taxonomic group (e.g. Pleocyemata), predicted by this study. Filled circle shows the gain of type 2 isoform in Hymenoptera; empty-strikethrough circle shows loss of type 1 isoform in Formicidae. Numbers at nodes represent Posterior Probability (PP) values. Scale bar: substitutions/site.

**Figure 3:**
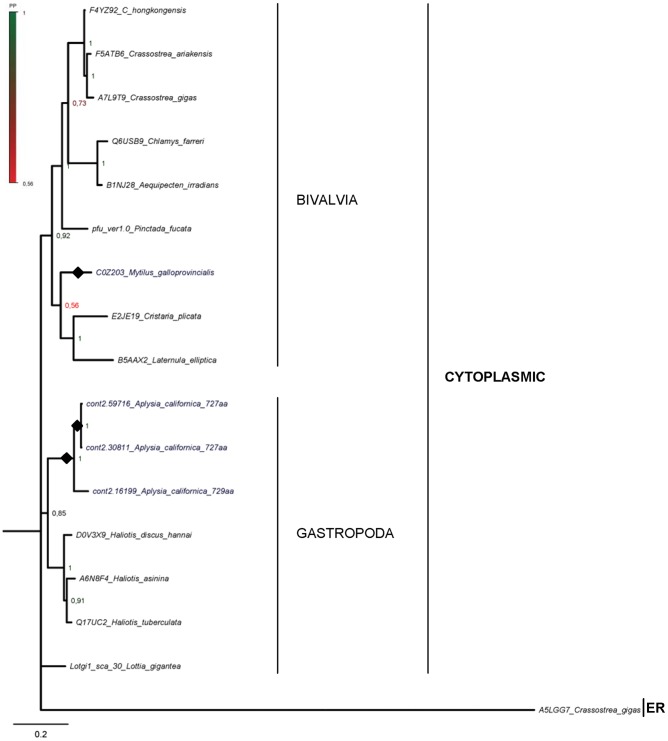
Bayesian inference phylogenetic tree based on Hsp90 protein sequences from Mollusca. Species in which multiple *hsp90* genes have been detected are in dark blue. Filled diamonds denote putative species-specific duplication events, predicted by this study. Numbers at nodes represent Posterior Probability (PP) values. Scale bar: substitutions/site.

**Figure 4:**
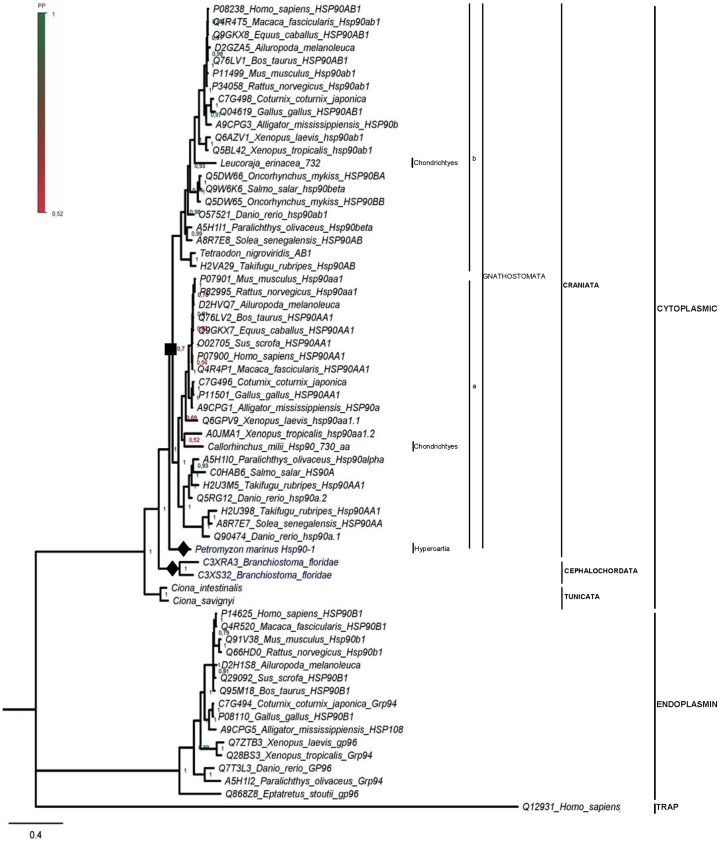
Bayesian inference phylogenetic tree based on Hsp90 protein sequences from Chordata. Species in which multiple *hsp90* genes have been detected are in dark blue. Filled diamonds denote putative species-specific duplication events, predicted by this study. Filled square denotes the duplication event resulting in the cognate and inducible isoforms of vertebrates. Numbers at nodes represent Posterior Probability (PP) values. Scale bar: substitutions/site.

Pairwise identities and similarities for protein sequences were calculated using the Needle module (EMBOSS, [57]), applying the BLOSUM62 matrix. Multiple alignments were performed using ClustalW [58]. Alignments were manually inspected to avoid errors owing to the program settings and in order to remove 1) low complexity regions or ambiguously aligned regions of the sequences, i.e. parts of the N-terminal and C-terminal ends and of the middle variable region (according to [15]), 2) parts of the alignment where some sequences contained gaps due to non-sequenced regions in the genome. Phylogenetic analysis using multiple protein sequence alignments was performed under Bayesian inference (BI) in MrBayes 3.1.2 [59] on XSEDE (Extreme Science and Engineering Discovery Environment) through the CIPRES (Cyberinfrastructure for Phylogenetic Research) Science Gateway v3.3 [60]. The best substitution model predicted by the Model Selection tool incorporated in MEGA5 [61] was the Jones, Taylor, and Thornton (JTT) model (gamma distributed). Two independent, simultaneous analyses were run for 10^7^ generations, each starting from different random trees with four chains (one cold and three incrementally heated) and sampling every 1000 generations. 2500 sampled generations were discarded as “burn-in”. A majority-rule consensus topology was created with the remaining samples, pooled together from the independent runs. The frequencies of each node of the consensus tree were represented as posterior probabilities. MEGA5 was used for the construction of Maximum Likelihood (ML) [62] trees. Tree topologies were evaluated applying the bootstrap test (100 pseudo-replicates) [63]. In regard to gaps handling, the “include all sites” option was used. The accession numbers of sequences used in the phylogenetic tree construction are included in Figures 1–4, Figures S2–S5 and Tables S1–S2.

## Results and Discussion

### The cytoplasmic Hsp90s and putative duplications in Fungi

Our analyses of fungal genomes supports the presence of more than one gene copies in several species (Figure 1, Figures S1 and S2, Table S1), besides the known case of two cytoplasmic isoforms in *Saccharomyces cerevisiae* [33], [34]. Analysis of the available genome from *Ajellomyces capsulatus* strain H143, through the BROAD Institute (Table S1, Figure S1), reveals that there are actually two identical *hsp90* copies (both at nucleotide and aminoacid level), tandemly arranged; one of them is complete (702 aa) and one truncated (612 aa), due to non-sequenced regions in the genome (data not shown). Duplicated genes have been also observed in species from Ascomycota, Basidiomycota and Mucoromycotina.

In the constructed trees (Figure 1 and Figure S2), the grouping of the cognate and inducible isoforms of *S. cerevisiae* with the proteins from the other *Saccharomyces* species is not highly supported. On the other hand, clustering of each of the two copies from the non ATCC *S. cerevisiae* strains with either the cognate or the inducible isoform from the ATCC *S. cerevisiae* is highly supported. It should be noted, however, that cognate and inducible genes have been experimentally verified only for the ATCC *S. cerevisiae* strain [33], [34]. Two sub-clades are formed within the clade of *Saccharomyces* genus (Figure 1); one consists of the isoforms from *S. cerevisiae*, *S. paradoxus*, *S. kudriavzevii* and *S. mikatae*; in the other sub-clade, the lager brewing yeast *S. pastorianus Weihenstephan*, an allopolyploid interspecies hybrid, is clustered with *S. bayanus*, one of the two species from which it originates [64].

The two Hsp90 proteins in *Saccharomyces* species are probably the result of the Whole-Genome Duplication supported by several studies [65]–[68], after which both copies were retained in the genome. The most striking physiological difference between *Saccharomyces* and other yeasts is its ability to ferment sugars vigorously under anaerobic conditions, producing ethanol [68]. Hsp90s are implicated in alcoholic fermentation [69], hence, their retention after the WGD may have been instrumental in its evolutionary adaptation to anaerobic growth. The retention and differential regulation of the *hsp* genes in the *Saccharomyces* genome is also in accordance with the observation that paralogs in yeast genomes diversify most frequently at the regulation level, in order to meet with diverse ecological niches [70].

An independent duplication probably led to the copies observed in *A. capsulatus*. Evidence has been found that an ancient WGD as well as recent gene duplications in *Rhizopus delemar* (Fungi; Mucoromycotina) led to the expansion of gene families related, among others, to signal transduction [71]. An interpretation for our data (Figure 1 and Figure S2) could be that independent duplication events took place also in *Mucor circinelloides* and *Phycomyces blakesleeanus*.

### The cytoplasmic Hsp90s and putative duplications in Arthropoda

Through our genome analyses we identified several arthropod species, not included in previous studies, with one or more *hsp90* cytoplasmic copies. For some species we verified the number of protein sequences recorded in databases, whereas for others we showed that additional *hsp90* copies exist (Figure 2, Figures S1 and S3 and Table S1).

Even though single copies have been identified in all available representatives from the orders of Coleoptera and Lepidoptera, two or more gene copies exist in the genome of several dipteran species, notably *Drosophila willistoni*, *Glossina morsitans* and *Culex quinquefasciatus* (Figure 2, Figure S3 and Table S1). We also show that in Hymenoptera two copies encoding for two different Hsp90 isoforms exist in several species from the superfamilies of Apoidea and Chalcidoidea, yet, there are single copies in the family of Formicidae, with the sole exception of *Linepithema humile* (Table S1). Lack of complete genomes from representatives of Ichneumonoidea does not allow us to conclude as to the gene copy number in this superfamily.

Using representative protein sequences (Table S2) and sequences assembled in the present study (Table S1 and Figure S1), BI and ML trees were constructed (Figure 2 and Figure S3). Several duplication events seem to have taken place at various points during the evolution of Arthropoda, most of them species-specific (e.g. *G. morsitans, L. humile*). In all studied mosquito species multiple *hsp90* copies have been found (Figure 2, Figure S3 and Table S1), which probably resulted from independent duplications in each species. For Hymenoptera, it appears that the isoform previously characterized as ‘traditional’ [72] existed in the common ancestor of all Endopterygota according to the constructed trees (type 1, Figure 2 and Figure S3), but was lost in the family of Formicidae. One duplication event probably took place after the radiation of Hymenoptera from the rest of the Endopterygota, leading to the second isoform of Hymenoptera (type 2, Figure 2 and Figure S3). This isoform was previously considered as *Apis melifera*-specific [72], yet our study shows that it is also present in Apoidea, Chalcidoidae and Formicidae. The two types differ both in nucleotide sequence and genomic structure (data not shown).

Our trees also suggest the occurrence of at least two duplication events in the crustacean lineage (Figure 2 and Figure S3). The first one, supported by the two isoforms from *Portunus trituberculatus*, probably took place within Decapoda before the divergence of Pleocyemata and Dendrobranchiata. The second one, responsible for the *Penaeus monodon* isoforms, seems to have taken place within Dendrobranchiata.

Several factors, such as transposable elements and habitat preferences, can account for the duplication events and retention of multiple gene copies observed in various lineages of Arthropoda [73]–[90].

The expression of heat shock protein genes in insects, as a response mechanism to stress, has been the object of several studies (reviewed in [73]) and revealed that insects adopt different defensive strategies, correlated with exposure to various biotic and abiotic agents. For example, up-regulation of Hsps contributes to dehydration tolerance in some insects [74], nonetheless their expression is not influenced by dehydration in *D. melanogaster* [75]. *D. willistoni*, a tropical species and the only *Drosophila* species found to bear two *hsp90* copies, has habitat differences with related species, including acclimation of metabolic rates [76], [77]. Expression patterns of the *A. melifera* (Hymenoptera) specific isoform (Figure 2, type 2) are caste- and age- dependent [78]. Retention of this isoform and loss of the insect specific isoform in ants (Formicidae) could correlate with the significant diversity in their lifestyles, their organization in populous colonies and delegation of reproductive and non-reproductive roles among the members of the colonies [79]. *L. humile* is one of the most widely distributed destructive invasive ant species [80]; it seems to have several species-specific duplications not found in other taxa [81] and a similar duplication could account for the two *hsp90* genes copies.

Transposable Elements (TEs) have a well-established role in the origin of new genes and genome evolution of eukaryotes [82]–[84] and could also be correlated with the duplicated genes in dipteran species. *D. willistoni* is considered an exceptional outlier in regard to other *Drosophila* species by several criteria, among which the increased content in TEs (15.57% as opposed to just 5.35% in *D. melanogaster*), some of which seem to be ancient in the *D. willistoni* genome [85]–[87]. TEs also constitute approximately 16% of the eukaryotic component and more than 60% of the heterochromatic component of the *Anopheles gambiae* genome [88], [89] and 50% of the *Aedes aegypti* genome [90]. Furthermore, remnants of different TE families have been identified in the regions flanking the *hsp90* copies of several mosquito species (NW_001810125.1, NW_001811357.1, data not shown).

Up to now, the majority of arthropods were considered to possess a single cytoplasmic *hsp90* [18]. Nevertheless, it has been reported that two genes encoding the same aminoacid sequence exist in the genome of the mosquito *A. albimanus* [26], that *A. melifera* possesses two cytoplasmic Hsp90 isoforms [72] and multiple genes exist in *A. gambiae* [18]. The only case where two isoforms have been reported in Crustacea is that of *P. trituberculatus* [28]. The fact that single genes have been reported for specific arthropoda species could be attributed to lack of genome-wide studies (e.g. due to the nature of experimental approaches) or loss of duplicated genes. Our analyses support the existence of multiple genes in different species and point out the need for high-throughput analyses of genomes from crustacean and other arthropod lineages (e.g. Ichneumonoidea), in order to delineate the actual gene copy number and evolutionary course of HSP90 family in this Phylum.

### The cytoplasmic Hsp90s and putative duplications in Mollusca

In order to enrich the existing dataset of available molluscan Hsp90 sequences and investigate the existence of single or multiple Hsp90 genes/isoforms within Mollusca, we analyzed recently released genomes of bivalve and gastropod species, as well as publicly available ESTs from bivalve, gastropod and cephalopod species (Figure S1, Tables S1 and S3).

In Bivalvia, our analysis of the *Crassostrea gigas* genome verified that there is a single gene copy encoding for an Hsp90 homolog (Figure 3, Figure S4 and Table S1). The current release of *Pinctada fucata* genome consists of scaffolds with relatively small size. Combining the results from TBLASTN comparisons against its genome with the available *P. fucata* cDNA sequences, we were able to assemble a unique Hsp90 sequence (Figure S1). A single gene is also supported by available ESTs from *M. californianus* (Table S3), yet only a partial sequence could be assembled (Figure S1). The gastropod *Lottia gigantea* seems to possess a single gene copy, as verified by analysis of genome and available ESTs (Table S1 and Table S3). On the contrary, three contigs have been identified to contain *hsp90* sequences in another gastropod, *Aplysia californica*. The *hsp90* coding sequences (cds) in cont2.59716 and cont2.16119 are 86% and 94% identical at nucleotide and protein level, respectively, while those in cont2.30811 and cont2.59716 differ by three nucleotides and one amino-acid residue; flanking regions are dissimilar in both comparisons. Few ESTs were collected from the cephalopods *Euprymna scolopes* and *Idiosepius paradoxus* (Table S3); there seem to be different populations of ESTs in each species (data not shown), but a complete sequence could not be assembled due to limited data availability.

In the constructed trees (Figure 3 and Figure S4), cytoplasmic Hsp90s from Mollusca are clustered in clades according to their taxonomic classification. The *A. californica* proteins form a separate clade, indicating that they are the result of on independent duplication event.

Members of the Mollusca were either absent or under-represented in previous phylogenetic analyses concerning the Hsp90 family [15], [18], since there are only few Hsp90 cDNA sequences publicly available for the Phylum. We show here, that besides the two *hsp90* gene copies recently isolated in *Mytilus galloprovincialis* [27], [91], other molluscan taxa seem to possess multiple *hsp90* gene copies. A recent comparative genome structure analysis of three molluscan species, i.e. scallops (Bivalvia), pygmy squid and nautilus (Cephalopoda) showed that large-scale duplication events occurred after divergence from Gastropoda [92]. Phylogenetic trees point to a single duplication event that occurred in the cephalopod lineage, yet it is not clear whether the duplication events can be traced back to a common molluscan ancestor of these species [92]. Due to the lack of sufficient number of complete molluscan genomes, it is not feasible to determine whether the observed copies in *M. galloprovincialis* and *A. californica* and the different ESTs populations in the two cephalopods are the result of a species-specific duplication event or are related to an old event that took place in a common molluscan ancestor.

### The cytoplasmic Hsp90s and putative duplications in Chordata

Data in public databases concerning the HSP90 family in the class of Chondrichthyes (Craniata; Vertebrata; Gnathostomata) are restricted to one partial Hsp90 sequence from *Scyliorhinus torazame* (AC AAG22091), few ESTs and the *Callorhinchus milii* genome. Analysis of the low-coverage (1.4x) *C. milii* genome revealed at least one *hsp90* locus (Table S1); in combination with available ESTs (Table S4), a complete Hsp90 sequence was assembled (Figure S1), while a second group consisting of only few ESTs was identified (Table S4). Our analysis of overlapping ESTs from *Leucoraja erinacea* (Table S4) resulted in a complete amino-acid sequence (Figure S1). ESTs collected from *Torpedo californica* and *Squalus acanthias* were only partially overlapping, thus a complete sequence could not be assembled.

For Petromyzontiformes (Craniata; Vertebrata; Hyperoartia), two scaffolds encoding for Hsp90 homologs were detected through BLAST searches against the sea lamprey *Petromyzon marinus* genome (Table S1). A partial amino-acid sequence is predicted to be encoded by scaffold GL498392. Using this sequence, as well as overlapping ESTs collected from *P*. *marinus* cDNA libraries (Table S4, second group) an amino-acid sequence of 611 residues was assembled (Table S1 and Figure S1).

*Takifugu rubripes* and *Tetraodon nigroviridis* (Vertebrata; Gnathostomata; Teleostomi; Euteleostomi; Actinopterygii) possess some of the smallest known vertebrate genomes, whose analyses and comparison with the human genome supports a Whole-Genome Duplication in the teleost fish lineage [93], [94]. For *T*. *rubripes*, we verified (Table S1) that the three cytoplasmic Hsp90 homologs recorded in PROSITE are encoded by distinct genomic regions located on the 14^th^ chromosome; the first two copies (characterized as AA1) are tandemly arranged and are in a head-to-head arrangement with the third gene (AB). Our analysis of *T*. *nigroviridis* draft genome reveals that similarly to *T. rubripes*, *T. nigroviridis* seems to possess one AB and two AA isoforms, still, non-sequenced regions in the genome allowed us to assemble only the complete AB isoform (Table S1 and Figure S1).

For the subphylum of Cephalochordata, we found two uncharacterized sequences from *Branchiostoma floridae* (Table S1) that bear all seven signatures of the HSP90 family [15], show approximately 80% identity with the *Mytilus* and human cytoplasmic Hsp90 isoforms, indicating that they belong to the HSP90 family, and verified that they are encoded by two discrete *hsp90* copies tandemly arranged in the *B. floridae* genome (Table S1, data not shown).

The subphylum of Tunicata was represented in a previous study [18] by a single sequence derived from a *Ciona intestinalis* (class Ascidiacea) cDNA clone (AC AK115284). The predicted amino-acid sequence (Figure S1) was used in TBLASTN searches against the *C. intestinalis* and *C. savignyi* genome assemblies and revealed the existence of single loci coding for a cytoplasmic Hsp90 in each species (Table S1 and Figure S1).

BI and ML trees were constructed (Figure 4 and Figure S5), using publicly available complete sequences from Chordata (Table S2), as well as the deduced complete sequences identified in the present study (Figure S1). Another tree was constructed using additionally the partial *P. marinus* Hsp90-2 sequence (data not shown). The deduced *C. milii* Hsp90 clusters with the a isoforms, the *L. erinacea* sequence is clustered with the cytoplasmic b isoform, while the sequences from *P. marinus* form a branch separately from the a and b isoforms of Gnathostomata. The two sequences from *Branchiostoma* cluster in a separate clade, sister to the clade of Craniata (Figure 4 and Figure S5).

To date, chordate representative sequences used in Hsp90 phylogenetic analyses were derived mainly from the classes of Actinopterygii and Sarcopterygii [15], [18]. Our search through complete genomes and available ESTs resulted in the identification/chacterization of additional Hsp90 Craniata sequences from the class of Chondrichtyes and the order of Petromyzontiformes, as well as sequences from the subphyla of Cephalochordota and Tunicata. Evidence has been found for two rounds of genome duplication (namely 1R and 2R) both before and after the split between jawless vertebrates (Hyperotreti and Cephalochordata) and jawed vertebrates (Gnathostomata), approximately 520 to 550 MYA [95]–[97]. These genome duplications took place after the divergence of tunicates but before the split between Chondrichthyes and Euteleostomi (bony vertebrates). Most of the duplicate genes resulting from these whole-genome events have been lost; yet, a number of genes involved in developmental processes are retained [95]. The lamprey appears to have diverged between the two rounds of duplication; therefore, it is possible that the two genes in *P. marinus* are the result of the first round. On the other hand, an independent duplication event is required to account for the different copies of the cytoplasmic *hsp90* genes detected in *B. floridae*. The clustering pattern of AA isoforms in *Takifugu* and *Tetraodon* maybe indicative of the fishes-specific genome duplication, namely 3R, estimated to have taken place around 350 MYA [93], [94].

It has been suggested that the duplication event which generated the a and b Hsp90 isoforms took place within the lineage of vertebrates, shortly before the emergence of the teleosts from the rest of the vertebrate lineage, approximately 500 MYA [15], [20], [21]. Our results indicate that the two cytoplasmic isoforms also exist in Chondricthyes; therefore we set this gene duplication event earlier in the vertebrate evolution, probably within Gnathostomata, before the separation of Euteleostomi and Chondricthyes and after their separation from Hyperoartia.

## Conclusions

In the present study we sought to analyze the evolution of the HSP90 family, through the gene copy numbers and putative duplication events, focusing on the cytoplasmic members of Fungi and Metazoa. We detected and retrieved Hsp90s in sequence databases, analyzed genome and ESTs sequences, in order to enrich our dataset with taxonomic groups not present in previous studies. Overall, we provide evidence for duplicated genes in several fungal and animal species that in most cases seem to be the outcome of independent duplication events within each species; nonetheless we suggest that some duplication events affected a wider taxonomic group. The duplicated genes detected in some species could be the result of known whole-genome duplications, as in the case of *Saccharomyces*, or the result of small-scale duplications. In addition, we infer that a gene loss took place in a hymenopteran family. Retention or loss of duplicated genes could be correlated to environmental stimuli or the habitual needs of various taxa. Finally, we were able to make a more precise estimation concerning the duplication event responsible for the cognate and inducible isoforms in vertebrates, and place it shortly after the split of Hyperoartia from Gnathostomata. Even though there is a significant increase of genome-wide information, still the need for high-throughput analyses of various taxonomic groups (e.g. Mollusca) is compelling, in order to infer the steps in the evolution of the HSP90 family in a more conclusive manner.

## Supporting Information

Figure S1 **Hsp90s detected in genomes and ESTs analyzed in this study.** Information on species and sequences are provided in Tables S1, S3–S4. Non-sequenced regions in genomic sequences are represented by a string of Ns.
(DOC)

Figure S2 **ML tree using Hsp90 protein sequences from Fungi.** Species in which multiple *hsp90* genes have been detected are in bold and italics. Filled diamonds denote putative species-specific duplication events, predicted by this study. Stars represent whole-genome duplications reported by previous studies. Numbers represent bootstrap values (percentages); values below 50% are not shown.
(TIFF)

Figure S3 **ML tree using Hsp90 protein sequences from Arthropoda.** Species in which multiple *hsp90* genes have been detected are in bold and italics. Filled diamonds denote putative species-specific duplication events, predicted by this study. Filled squares denote duplication events in the common ancestor of a wide taxonomic group (e.g.Pleocyemata), predicted by this study. Filled circle shows the gain of type 2 isoform in Hymenoptera; empty-strikethrough circle shows loss of type 1 isoform in Formicidae. Numbers represent bootstrap values (percentages); values below 50% are not shown.
(TIF)

Figure S4 **ML tree using Hsp90 protein sequences from Mollusca.** Species in which multiple *hsp90* genes have been detected are in bold and italics. Filled diamonds denote putative species-specific duplication events, predicted by this study. Numbers represent bootstrap values (percentages); values below 50% are not shown.
(TIFF)

Figure S5 **ML trees using Hsp90 protein sequences from Chordata.** Species in which multiple *hsp90* genes have been detected are in bold and italics. Filled diamonds denote putative species-specific duplication events, predicted by this study. Filled square denotes the duplication event resulting in the cognate and inducible isoforms of vertebrates. Numbers represent bootstrap values (percentages); values below 50% are not shown.
(TIF)

Table S1 **Species for which complete genomes were analyzed in this study, databases through which they were assessed and derived Hsp90 cytoplasmic sequences.**
(DOC)

Table S2 **The species for which Hsp90s annotated in UniProt/TrEMBL were used in this study.**
(DOC)

Table S3 **Molluscan ESTs bearing** ***hsp90*** **sequences, analyzed in the present study.**
(DOC)

Table S4 ***Petromyzon marinus, Callorhinchus milii*** **and** ***Leucoraja erinacea*** **ESTs bearing** ***hsp90*** **sequences, analyzed in the present study.**
(DOC)
