# Supplementary material for: Assessment and Reconstruction of Novel HSP90 Genes: Duplications, Gains and Losses in Fungal and Animal Lineages
Source: PLoS One. 2013 Sep 16;8(9):e73217. doi: 10.1371/journal.pone.0073217 (PMC3774752; doi:10.1371/journal.pone.0073217)
Supplement: Figure S1 — Hsp90s detected in genomes and ESTs analyzed in this study. Information on species and sequences are provided in Tables S1, S3–S4. Non-sequenced regions in genomic sequences are represented by a string of Ns. (DOC) [file pone.0073217.s001.doc]

**FUNGI**

>scaffold_11_*Gonapodya_prolifera*_cds_2118bp

ATGTCTGCTGCTGACACTGAGACGTTCGCCTTCCAGGCCGAGATCTCCCAGTTAATGTCTCTAATCATCA

ACACCTTCTACTCCAACAAGGAGATTTTCCTTCGAGAATTGATCTCTAACGCCTCGGACGCTCTTGACAA

GATTCGATACCTCTCCCTCACCGACTCCAGCCAACTCGACTCCGGCAAGGATCTGTTCATCCGAATCATT

CCTGACAAGGAGAACAAGACCCTCACCATTCGCGATGCCGGTATCGGTATGACCAAGGCCGACCTTGTCA

ACAACCTCGGTACCATTGCCAAGTCTGGCACCAAGGCCTTCATGGAGGCCCTCCAGTCCGGCGCTGACAT

TTCCATGATCGGACAGTTCGGTGTCGGTTTCTACTCCGCCTACCTCGTCGCCGACCGCGTGCAGGTCATC

ACCAAGCACAACGACGACGAGCAGTACATTTGGGAGTCTGCTGCCGGCGGTAGCTTCACCATCACTCCCG

ACGAGTCCAGCTCCCTCGGGCGTGGTACTGCCATCCGCCTCTACCTGAAAGAGGACCAGCTCGAGTACCT

TGAGGAGCGCCGCATCAAGGAGGTCGTGAAGAAGCACTCCGAGTTCATCAACTACCCTATCGAGCTCGAG

ATTGTCAAGGAAGTCGAGGAGGAGGTTGAGGATGACGAGGAGGAGAAGAAGGACGAGGACAAGGACGAGA

CCAAGATTGAAGAGGTTGAGGACGACGAGGAGAAGAAGGACAAGAAGAAGAAGAAGGTCAAGAAACAAAC

TACTGAGGAGGAGGTTCTCAACAAGACCAAACCCATCTGGACCCGCAACCCTGAGGAGATCAAGGAGGAG

GAGTACCACGCCTTTTACAAGGCCATCTCCAACGACTGGGAGGACAGTCTTGCTTACAAGCACTTCTCCG

TCGAGGGTCAGCTCGAGTTCCGTGCTATCCTCTTCGTTCCCCGCCGCGCGCCCTTCGACCTGTTCGAAAT

GCGCAAGAAGCGCAACAACATCAAACTCTACGTCAGGCGTGTGTTCATCACTGATGACTGCGAGGACCTT

ATTCCCGAGTGGCTTTCCTTTGTCAAGGGAATTGTCGACTCCGAGGACCTTCCCCTCAACATCTCCCGTG

AGATGCTCCAACAGAACAAGATCTTGAAGGTTATCCGCAAGAACTTAGTCAAGAAGTGCATTGAGCTCTT

CACCGAAATCTCCGAGGACAAGGAGAAGTTTAAGACCTTCCACGAGGCCTTCGGAAAGAACATCAAGCTC

GGAGTTCACGAGGACAGCACCAACCGCAACAAGCTTGCCGAGCTCCTCCGCTTCAACAGCACCAAGTCTG

GCGAAGAGCTCACTTCGTTCAAGGACTACATTACGCGCATGCCCGAGAAGCAGAAGAACATCTACTACAT

CACCGGAGAGTCAAAGGCCGCCGTTGAGCACTCGCCTTTCCTCGAGGTGCTCAAGAAGAAGGGCTTCGAA

GTTCTCTACCTTGTTGACCCCATTGATGAGTACATGATCCAGCAGCTCAAGGACTACGAGGGCAAGAAGC

TCCAGAGTGCCACCAAGGAGGGCCTTGAGCTCGAGGAGGATGAGGACGAGAAGAAGAAGTTCGAGGAGGA

GAAGGCTGCCTTCGAGGCCCTCGGAAAAGCGATGAAGGACATTCTCGGCGACCGTGTGGAGAAAGTTACC

GTTTCCAACCGTATTGTCGACGCTCCTTGCGTTCTTGTCACAGGCCAGTTCGGTTGGTCCGCCAACATGG

AGCGGATTATGAAAGCTCAAGCTCTCCGTGACTCGAGCATGTCTGCCTACATGGCCTCGAAGAAGACCAT

GGAGATCAACCCGCAAAACCCTATCATCCGCTCCCTGAAGTCCAAGGTCGAGGCCGACAAGAACGACAAG

ACCGTAAAGGACTTGGTTTTCCTCCTCTTCGAGACCTCTCTCCTCCAATCCGGATTCAACCTGGAAGACC

CATCCGGCTTCGCCTCCCGTATCCACCGAATGATCAAACTTGGTCTGTCGATCGACGACGACGAAGAGGT

CGGCGAGTCTGGAGATGGCGCAATTGACGAGGACATGCCCCCTCTGGAAGAAGGCGGTGCTGAGTCCAAG

ATGGAGGAGGTTGACTAA

>scaffold_11_*Gonapodya_prolifera*_705aa

MSAADTETFAFQAEISQLMSLIINTFYSNKEIFLRELISNASDALDKIRYLSLTDSSQLDSGKDLFIRII

PDKENKTLTIRDAGIGMTKADLVNNLGTIAKSGTKAFMEALQSGADISMIGQFGVGFYSAYLVADRVQVI

TKHNDDEQYIWESAAGGSFTITPDESSSLGRGTAIRLYLKEDQLEYLEERRIKEVVKKHSEFINYPIELE

IVKEVEEEVEDDEEEKKDEDKDETKIEEVEDDEEKKDKKKKKVKKQTTEEEVLNKTKPIWTRNPEEIKEE

EYHAFYKAISNDWEDSLAYKHFSVEGQLEFRAILFVPRRAPFDLFEMRKKRNNIKLYVRRVFITDDCEDL

IPEWLSFVKGIVDSEDLPLNISREMLQQNKILKVIRKNLVKKCIELFTEISEDKEKFKTFHEAFGKNIKL

GVHEDSTNRNKLAELLRFNSTKSGEELTSFKDYITRMPEKQKNIYYITGESKAAVEHSPFLEVLKKKGFE

VLYLVDPIDEYMIQQLKDYEGKKLQSATKEGLELEEDEDEKKKFEEEKAAFEALGKAMKDILGDRVEKVT

VSNRIVDAPCVLVTGQFGWSANMERIMKAQALRDSSMSAYMASKKTMEINPQNPIIRSLKSKVEADKNDK

TVKDLVFLLFETSLLQSGFNLEDPSGFASRIHRMIKLGLSIDDDEEVGESGDGAIDEDMPPLEEGGAESK

MEEVD

>supercontig3­_Hsp90-1_*Ajellomyces_capsulatus_H143_*cds_2109bp

ATGTCGTCTGAAACTTTCGAGTTCCAGGCTGAGATTTCTCAGCTCCTCTCCCTCATTATCAACACCGTCT

ACTCCAACAAAGAGATCTTCCTGCGAGAACTCATTTCCAACTGTTCCGATGCTCTTGATAAAATCCGCTA

TGAGGCGCTTTCCGACCCAAGCAAGCTCGACTCCAACAAGGATCTCCGCATCGATATCATCCCCGACAAG

GAAAACAAGACCCTGACCATAAGCGATACCGGTATCGGTATGACCAAGGCTGATCTCGTCAACAACCTGG

GTACCATCGCCCGCTCGGGTACTAAGCAATTTATGGAAGCGCTCACTGCTGGTGCGGATATTTCTATGAT

TGGCCAATTCGGTGTTGGTTTCTACTCTGCCTACCTCGTTGCCGATAAGGTGACCGTCATTTCCAAGCAC

AACGATGACGAACAGTACATCTGGGAGTCCAGTGCTGGTGGCACCTTCAAAATCACCCAGGACACCGATG

GAGAGCCACTTGGCCGTGGTACCAAGATGATCCTTCATCTCAAGGACGAGCAGACCGAATACCTGAATGA

GAGCAAGATCAAGGAAGTCGTGAAGAAGCACTCTGAGTTCATCTCCTACCCCATCTACCTTCATGTCCTC

AAGGAGACTGAGAAGGAGGTTCCCGATGAAGATGCTGAGGAGGTGAAGGACGAGGGTGACGACAAGGCTC

CCAAGGTCGAAGAGGTCGATGACGACGAAGAGGACAAAACGAAAGAGAAGAAGACCAAGAAAATCAAGGA

GACTAAGATCGAGGAGGAAGAGCTAAACAAGACCAAGCCTATCTGGACTCGCAATCCTGCTGATATTACT

CAGGAGGAATATGCTTCCTTCTACAAGACTCTTTCCAACGACTGGGAGGACCATCTTGCTGTGAAGCACT

TCTCCGTCGAAGGTCAACTCGAGTTCCGTGCGATCCTCTTCGTTCCCAAGCGTGCTCCTTTCGATCTCTT

CGAGACCAAGAAGACTAAGAACAACATCAAGCTCTACGTCCGCCGTGTCTTCATTACTGATGATGCTACC

GATCTCATTCCCGAATGGCTCAGCTTCATCAAGGGTGTTGTCGACTCTGAGGACCTTCCTCTCAACCTCT

CTCGTGAGACTCTCCAGCAGAACAAAATTATGAAGGTCATCAAGAAGAACATCGTCAAAAAGACCCTCGA

GCTTTTCAATGAGATTGCGGAAGACCGTGAGCAATTCGACAAGTTCTACTCTGCTTTCAGCAAGAACATC

AAACTCGGTATCCATGAGGATGCCCAGAACCGCCCTGCTCTTGCGAAACTCCTCCGCTTCAACTCCACCA

AGTCTGGTGACGAGACCACCTCTCTTACCGACTATGTCACCCGCATGCAAGAGCACCAGAAACAAATGTA

CTACATCACTGGCGAGTCTCTCAAGGCTGTTCAGAAGTCTCCCTTCCTCGATACCCTTAAGGAGAAGAAC

TTCGAGGTTCTCTTCCTTGTTGATCCCATTGATGAGTACGCCATGACCCAGCTCAAGGAGTTCGATGGCA

AGAAGCTCGTCGACATCACCAAGGACTTCGAGCTCGAGGAAACGGAGGAGGAGAAGAAGGCCCGCGAAGC

CGAAGAGAAGGAATACGAAGGCCTCGCCAAGAGCTTGAAGAACGTCCTTGGCGACAAGGTCGAGAAGGTT

GTTGTCTCCCACAAACTTATCGGCTCCCCCTGTGCCATCCGCACTGGCCAATTCGGCTGGTCCGCCAACA

TGGAACGTATCATGAAAGCGCAAGCCCTCCGCGACACTTCCATGAGCTCCTACATGTCCTCCAAAAAGAC

CTTCGAAATCTCGCCAAAGTCCCCAATCATCCAGGAGCTCAAGAAGAAGGTCGAGGCCGACGGCGAGAAC

GACCGCACAGTCAAATCCATCACCCAACTCCTTTTCGAAACCTCTCTTCTCGTCTCGGGCTTCACCATCG

AGGAGCCTTCCGGCTTTGCGGAGCGCATTCACAAGCTCGTTTCTCTTGGTTTGAACATTGATGAGGATGC

TGAAACTTCCGAGGAGAAAGAGGCAGACACTGTCGTCGCTGAAGCTCCGGGTGAGAGCGCTATGGAGGAG

GTTGATTAG

>supercontig3­_Hsp90-1_*Ajellomyces_capsulatus_H143_*702aa

MSSETFEFQAEISQLLSLIINTVYSNKEIFLRELISNCSDALDKIRYEALSDPSKLDSNKDLRIDIIPDK

ENKTLTISDTGIGMTKADLVNNLGTIARSGTKQFMEALTAGADISMIGQFGVGFYSAYLVADKVTVISKH

NDDEQYIWESSAGGTFKITQDTDGEPLGRGTKMILHLKDEQTEYLNESKIKEVVKKHSEFISYPIYLHVL

KETEKEVPDEDAEEVKDEGDDKAPKVEEVDDDEEDKTKEKKTKKIKETKIEEEELNKTKPIWTRNPADIT

QEEYASFYKTLSNDWEDHLAVKHFSVEGQLEFRAILFVPKRAPFDLFETKKTKNNIKLYVRRVFITDDAT

DLIPEWLSFIKGVVDSEDLPLNLSRETLQQNKIMKVIKKNIVKKTLELFNEIAEDREQFDKFYSAFSKNI

KLGIHEDAQNRPALAKLLRFNSTKSGDETTSLTDYVTRMQEHQKQMYYITGESLKAVQKSPFLDTLKEKN

FEVLFLVDPIDEYAMTQLKEFDGKKLVDITKDFELEETEEEKKAREAEEKEYEGLAKSLKNVLGDKVEKV

VVSHKLIGSPCAIRTGQFGWSANMERIMKAQALRDTSMSSYMSSKKTFEISPKSPIIQELKKKVEADGEN

DRTVKSITQLLFETSLLVSGFTIEEPSGFAERIHKLVSLGLNIDEDAETSEEKEADTVVAEAPGESAMEE

VD

>supercontig3­_Hsp90-2_*Ajellomyces_capsulatus_H143_*cds_1838bp

ATGTCGTCTGAAACTTTCGAGTTCCAGGCTGAGATTTCTCAGCTCCTCTCCCTCATTATCAACACCGTCT

ACTCCAACAAAGAGATCTTCCTGCGAGAACTCATTTCCAACTGTTCCGATGCTCTTGATAAAATCCGCTA

TGAGGCGCTTTCCGACCCAAGCAAGCTCGACTCCAACAAGGATCTCCGCATCGATATCATCCCCGACAAG

GAAAACAAGACCCTGACCATAAGCGATACCGGTATCGGTATGACCAAGGCTGATCTCGTCAACAACCTGG

GTACCATCGCCCGCTCGGGTACTAAGCAATTTATGGAAGCGCTCACTGCTGGTGCGGATATTTCTATGAT

TGGCCAATTCGGTGTTGGTTTCTACTCTGCCTACCTCGTTGCCGATAAGGTGACCGTCATTTCCAAGCAC

AACGATGACGAACAGTACATCTGGGAGTCCAGTGCTGGTGGCACCTTCAAAATCACCCAGGACACCGATG

GAGAGCCACTTGGCCGTGGTACCAAGATGATCCTTCATCTCAAGGACGAGCAGACCGAATACCTGAATGA

GAGCAAGATCAAGGAAGTCGTGAAGAAGCACTCTGAGTTCATCTCCTACCCCATCTACCTTCATGTCCTC

AAGGAGACTGAGAAGGAGGTTCCCGATGAAGATGCTGAGGAGGTGAAGGACGAGGGTGACGACAAGGCTC

CCAAGGTCGAAGAGGTCGATGACGACGAAGAGGACAAAACGAAAGAGAAGAAGACCAAGAAAATCAAGGA

GACTAAGATCGAGGAGGAAGAGCTAAACAAGACCAAGCCTATCTGGACTCGCAATCCTGCTGATATTACT

CAGGAGGAATATGCTTCCTTCTACAAGACTCTTTCCAACGACTGGGAGGACCATCTTGCTGTGAAGCACT

TCTCCGTCGAAGGTCAACTCGAGTTCCGTGCGATCCTCTTCGTTCCCAAGCGTGCTCCTTTCGATCTCTT

CGAGACCAAGAAGACTAAGAACAACATCAAGCTCTACGTCCGCCGTGTCTTCATTACTGATGATGCTACC

GATCTCATTCCCGAATGGCTCAGCTTCATCAAGGGTGTTGTCGACTCTGAGGACCTTCCTCTCAACCTCT

CTCGTGAGACTCTCCAGCAGAACAAAATTATGAAGGTCATCAAGAAGAACATCGTCAAAAAGACCCTCGA

GCTTTTCAATGAGATTGCGGAAGACCGTGAGCAATTCGACAAGTTCTACTCTGCTTTCAGCAAGAACATC

AAACTCGGTATCCATGAGGATGCCCAGAACCGCCCTGCTCTTGCGAAACTCCTCCGCTTCAACTCCACCA

AGTCTGGTGACGAGACCACCTCTCTTACCGACTATGTCACCCGCATGCAAGAGCACCAGAAACAAATGTA

CTACATCACTGGCGAGTCTCTCAAGGCTGTTCAGAAGTCTCCCTTCCTCGATACCCTTAAGGAGAAGAAC

TTCGAGGTTCTCTTCCTTGTTGATCCCATTGATGAGTACGCCATGACCCAGCTCAAGGAGTTCGATGGCA

AGAAGCTCGTCGACATCACCAAGGACTTCGAGCTCGAGGAAACGGAGGAGGAGAAGAAGGCCCGCGAAGC

CGAAGAGAAGGAATACGAAGGCCTCGCCAAGAGCTTGAAGAACGTCCTTGGCGACAAGGTCGAGAAGGTT

GTTGTCTCCCACAAACTTATCGGCTCCCCCTGTGCCATCCGCACTGGCCAATTCGGCTGGTCCGCCAACA

TGGAACGTATCATGAAAGCGCAAGCCCTCCGCGACACTTCCATGAGCTCCTACATGTCCTCCAAAAAGAC

CTTCGAAATCTCGCCAAA

>supercontig3­_Hsp90-2_*Ajellomyces_capsulatus_H143_*612aa

MSSETFEFQAEISQLLSLIINTVYSNKEIFLRELISNCSDALDKIRYEALSDPSKLDSNKDLRIDIIPDK

ENKTLTISDTGIGMTKADLVNNLGTIARSGTKQFMEALTAGADISMIGQFGVGFYSAYLVADKVTVISKH

NDDEQYIWESSAGGTFKITQDTDGEPLGRGTKMILHLKDEQTEYLNESKIKEVVKKHSEFISYPIYLHVL

KETEKEVPDEDAEEVKDEGDDKAPKVEEVDDDEEDKTKEKKTKKIKETKIEEEELNKTKPIWTRNPADIT

QEEYASFYKTLSNDWEDHLAVKHFSVEGQLEFRAILFVPKRAPFDLFETKKTKNNIKLYVRRVFITDDAT

DLIPEWLSFIKGVVDSEDLPLNLSRETLQQNKIMKVIKKNIVKKTLELFNEIAEDREQFDKFYSAFSKNI

KLGIHEDAQNRPALAKLLRFNSTKSGDETTSLTDYVTRMQEHQKQMYYITGESLKAVQKSPFLDTLKEKN

FEVLFLVDPIDEYAMTQLKEFDGKKLVDITKDFELEETEEEKKAREAEEKEYEGLAKSLKNVLGDKVEKV

VVSHKLIGSPCAIRTGQFGWSANMERIMKAQALRDTSMSSYMSSKKTFEISP

>scaffold_06_*Mucor_circinelloides*_CBS277.49_cds_2100bp

ATGAGCGCTGAAACAGAAACATTTTCTTTCCAAGCTGAGATCTCTCAGTTGATGAGTCTGATCATCAACA

CCTTCTATTCCAACAAGGAAATCTTCTTGCGTGAATTGATCTCGAATGCCTCTGATGCTTTGGACAAGGT

GCGTTATCAATCCTTGACGGACCCTTCTGTGCTCGATTCTGAAAAGAATCTCTACATCCGCATCACGCCT

GACAAGGAAAACAACATTCTCTCCATCCGTGATACTGGTATTGGTATGACCAAGGCTGATTTGGTCAACA

ACTTGGGTACCATTGCCAAGTCTGGTACCAAGGCTTTCATGGAAGCTCTCTCCTCTGGTGCTGATATCTC

CATGATTGGTCAATTCGGTGTTGGTTTCTACTCTGCCTACCTCGTGGCTGACAAGGTCCAAGTCATCACC

AAGCACAATGATGACGAGCAATACATTTGGGAATCTGCTGCTGGTGGTTCTTTCACTATTACTCGTGACG

AGGTCAACCCCTCTCTTGGTCGTGGTACTGAAATGCGTCTCTTCATGAAGGAGGATCAACTTGAATACCT

TGACGAGAAGAAGATCAAGGACATTGTCAAGAAGCACTCTGAGTTCATCTCTTATCCCATCCAATTGGTC

GTTGAAAAGGAAGTCGAGAAGGAGGTCTCTGACGACGAATCTGCCGAACCTGCCTCTGAAGGTGCCAAGA

TTGAAGAGGTCACTGACGAGGACGACAAGAAGGACGAAAAGAAGAAGAAGACCATCAAGGAAATGGTCAC

TGAAAACGAAGAGTTGAACAAGACCAAGCCTCTCTGGACTCGCAACCCTGAAGATGTCAAGCCTGAAGAG

TACTCTGAATTCTACAAGGCCCTCACCAACGACTGGGAAGACCAATTGGCCGTCAAGCATTTCTCTGTGG

AAGGTCAACTCGAATTCCGTGCTATCCTGTTTGTCCCCAAGCGTGCTCCCTTTGACATGTTTGAAACCAA

GAAGAAGAGAAACAACATCAAGCTCTACGTCCGTCGTGTCTTCATCATGGACGACTGCGATGAATTGATT

CCTGAATGGCTCGGCTTCATCAAGGGTGTGGTTGATTCTGAGGATCTCCCTCTCAACATTTCTCGTGAGA

TGTTGCAACAAAACAAGATCTTGAAGGTGATCCGCAAGAACTTGGTCAAGAAGTGTCTTGAAATGTTCCA

AGAGATTGCTGAGGACAAGGAGCAATTTGACAAGTTCTACGAGGCCTTTGGTAAGAACATCAAGCTCGGT

ATCCACGAAGACACCCAAAACCGTGCCAAGCTCGCTGACCTGTTGCGCTACTACTCTACCAAGTCTGGTG

ATGAGATGACCTCCTTCAAGGACTACATTACTCGTATGCCCGAGAAGCAAAAGAACATTTACTACATTAC

TGGTGAATCTCGCACTGCTGTTGAAAACTCGCCTTTCCTCGAAGGTTTCAAGAAGAAGGGTATTGAGGTG

CTCTTGATGACTGATCCCATTGATGAGTATGCTACCACTCAATTGAAGGAATACGAGGATCACAAGTTGG

TCTGTATCACCAAGGAGGGTGCTGAAATCGAGGAGGATGAAGAAGAGAAGAAAGCACGCGAGGCTGAACA

AAAGGAGTATGAAGGTCTCTGCAAGACTGTCAAGGATATTCTCGGTGACAAGGTGGAGAAGGTCGTTCTC

TCCAACATCTTGACAGACTCTCCCTGTGTCTTGACTACCGGTCAATTTGGTTGGTCTGCCAACATGGAAC

GTATTATGAAGGCTCAGGCTCTGCGCGATTCTTCCATGTCTAGCTACATGGCTTCAAAGAAGACTTTGGA

ACTGAACCCCAACCACCCCATCATCAAGGCGCTTCGTGCCAAGGCTTCTGTCGATGCTGGTGATCGTACT

GTCAAGGATCTTGTTACCTTGTTGTACGAGACTTCCCTGTTGACTTCTGGTTTCTCTTTGGATGATCCCA

GCTCATTTGCTAGCCGTATTAACCGCATGGTCTCTCTTGGTCTCTCTATTGATGAGGATGAGCTCCCCAC

TGCTGAACCTGCCACTGAAACCCCTGCTGAAGACAATACCGAAGTCTCCAAGATGGAAGAAGTTGATTAG

>scaffold_06_ *Mucor_circinelloides*_CBS277.49_699aa

MSAETETFSFQAEISQLMSLIINTFYSNKEIFLRELISNASDALDKVRYQSLTDPSVLDSEKNLYIRITP

DKENNILSIRDTGIGMTKADLVNNLGTIAKSGTKAFMEALSSGADISMIGQFGVGFYSAYLVADKVQVIT

KHNDDEQYIWESAAGGSFTITRDEVNPSLGRGTEMRLFMKEDQLEYLDEKKIKDIVKKHSEFISYPIQLV

VEKEVEKEVSDDESAEPASEGAKIEEVTDEDDKKDEKKKKTIKEMVTENEELNKTKPLWTRNPEDVKPEE

YSEFYKALTNDWEDQLAVKHFSVEGQLEFRAILFVPKRAPFDMFETKKKRNNIKLYVRRVFIMDDCDELI

PEWLGFIKGVVDSEDLPLNISREMLQQNKILKVIRKNLVKKCLEMFQEIAEDKEQFDKFYEAFGKNIKLG

IHEDTQNRAKLADLLRYYSTKSGDEMTSFKDYITRMPEKQKNIYYITGESRTAVENSPFLEGFKKKGIEV

LLMTDPIDEYATTQLKEYEDHKLVCITKEGAEIEEDEEEKKAREAEQKEYEGLCKTVKDILGDKVEKVVL

SNILTDSPCVLTTGQFGWSANMERIMKAQALRDSSMSSYMASKKTLELNPNHPIIKALRAKASVDAGDRT

VKDLVTLLYETSLLTSGFSLDDPSSFASRINRMVSLGLSIDEDELPTAEPATETPAEDNTEVSKMEEVD

>scaffold_07_*Mucor_circinelloides*_CBS277.49_cds_2103bp

ATGTCTGCCGAAACCGAGACCTTTTCTTTCCAAGCTGAGATTTCTCAGTTGATGAGTTTGATCATCAACA

CCTTCTATTCTAACAAGGAAATTTTCTTGCGTGAATTGATCTCCAACTCTTCTGATGCTTTGGACAAGGT

TCGTTACCAATCCTTGACTGATCCCTCCGTCCTCGACGCTGAGAAGGATCTCTACATCCGTATCACCCCT

GACAAGGAAAACAACATCTTGTCCATCCGTGATACTGGTATCGGTATGACCAAGGCTGATTTGGTCAACA

ACTTGGGTACAATCGCCAAGTCTGGTACCAAGGCTTTCATGGAGGCCCTCTCCTCCGGTGCTGATATCTC

CATGATTGGTCAATTCGGTGTCGGTTTCTACTCTGCTTACCTCGTTGCTGACAAGGTCCAAGTCATCACC

AAGCACAACGATGACGAGCAATACATCTGGGAGTCTGCTGCCGGTGGTTCTTTCACCATCACCCGTGACG

AAGTCAACCCTTCCATTGGTCGTGGTACTGAGATGCGTCTCTTCATGAAGGAAGATCAACTCGAATATCT

CGACGAGAAGAAGATCAAGGATATCGTCAAGAAGCACTCCGAGTTCATCTCTTACCCTATCCAACTCGTT

GTTGAGAAGGAAGTCGAAAAGGAGGTCTCTGATGACGAGGAAGAGATTGTCGCTGAAGAGGGTGCCAAGA

TCGAAGAGGTTACCGATGAAGATGACAAGAAGGATGAGAAGAAGAAGAAGACCATCAAGGAAACCGTTGT

TGAGAACGAGGAGCTCAACAAGACCAAGCCTCTCTGGACCCGTAACCCTGAGGATGTCAAGCCCGAGGAA

TACTCTGAATTCTACAAGGCTCTCTCCAACGATTGGGAGGATCATTTGGCTGTCAAGCACTTCTCTGTTG

AAGGTCAACTCGAATTCCGTGCCATTCTCTACGTTCCCAAGCGCGCTCCTTTCGATATGTTTGAGACCAA

GAAGAAGAGAAACAACATCAAGCTCTACGTCCGTCGCGTCTTCATCATGGATGACTGTGAGGACTTGATG

CCTGAATGGCTCAGCTTCATCAAGGGTGTCGTCGATTCTGAAGATCTCCCTCTCAACATTTCTCGTGAAA

TGCTCCAACAAAACAAGATCTTGAAGGTCATCCGCAAGAACTTGGTCAAGAAGTGTCTTGAAATGTTCCA

AGAAATCTCTGAGGACAAGGAGAACTTTGACAAGTTCTACGAAGCTTTCAGCAAGAACATCAAGCTCGGT

ATTCACGAAGATACTCAAAACCGTAACAAGCTCGCTGAATTGTTGCGTTACCACTCTACCAAGTCTGGTG

ATGAGATGACCTCCTTCAAGGATTACGTTACCCGTATGCCCGAGAAGCAAAAGAACATTTACTACATCAC

TGGCGAATCTCGCGCTGCCGTCGAGAACTCTCCTTTCCTCGAAGGTTTCAAGAAGAAGGGAATTGAAGTC

CTCTTGATGACTGACCCCATTGATGAATATGCCACTACTCAACTCAAGGAATACGAGGATAAGAAGTTGG

TCTGTATCACCAAGGATGGTCTTGAGCTCGAAGAAGATGAGGAGGAGAAGAAGGCTCGTGAGGCTGAGAA

GGCTGAATATGAAGGTCTCTGCAAGACTGTCAAGGATATCCTCGGTGAGAAGGTCGAGAAGGTTGTCCTC

TCTTCCATGTTGACTGATTCCCCCTGTGTTTTGACCACTGGTCAATTCGGCTGGTCCGCTAACATGGAAC

GTATCATGAAGGCTCAAGCTCTCCGTGATTCTTCCATGTCCAGTTACATGGCCTCCAAGAAGACTTTGGA

ATTGAACCCTGACCACCCCATCATCAAGGCCCTCAAGTCCAAGGTTTCTGCTGATGCCAATGACCGCACT

GCCAAGGATCTTGTTACCTTGTTGTACGAGACCTCTTTGTTGACCTCTGGTTTCACTCTTGATGATCCCA

GCTCTTTCGCTACCCGTATCAACCGTATGGTTTCTCTCGGTCTTTCTATTGACGAAGACGAGCTTCCTAC

TGCTGACGAGCCTGCTACTGAAGCCGCTGCTGATGATAACGCTGAGGTTTCCAAGATGGAGGAGGTTGAT

TAA

>scaffold_07_[*Mucor_circinelloides_*CBS277.49*_*700aa](http://genome.jgi.doe.gov/Mucci2)

MSAETETFSFQAEISQLMSLIINTFYSNKEIFLRELISNSSDALDKVRYQSLTDPSVLDAEKDLYIRITP

DKENNILSIRDTGIGMTKADLVNNLGTIAKSGTKAFMEALSSGADISMIGQFGVGFYSAYLVADKVQVIT

KHNDDEQYIWESAAGGSFTITRDEVNPSIGRGTEMRLFMKEDQLEYLDEKKIKDIVKKHSEFISYPIQLV

VEKEVEKEVSDDEEEIVAEEGAKIEEVTDEDDKKDEKKKKTIKETVVENEELNKTKPLWTRNPEDVKPEE

YSEFYKALSNDWEDHLAVKHFSVEGQLEFRAILYVPKRAPFDMFETKKKRNNIKLYVRRVFIMDDCEDLM

PEWLSFIKGVVDSEDLPLNISREMLQQNKILKVIRKNLVKKCLEMFQEISEDKENFDKFYEAFSKNIKLG

IHEDTQNRNKLAELLRYHSTKSGDEMTSFKDYVTRMPEKQKNIYYITGESRAAVENSPFLEGFKKKGIEV

LLMTDPIDEYATTQLKEYEDKKLVCITKDGLELEEDEEEKKAREAEKAEYEGLCKTVKDILGEKVEKVVL

SSMLTDSPCVLTTGQFGWSANMERIMKAQALRDSSMSSYMASKKTLELNPDHPIIKALKSKVSADANDRT

AKDLVTLLYETSLLTSGFTLDDPSSFATRINRMVSLGLSIDEDELPTADEPATEAAADDNAEVSKMEEVD

>scaffold_1_*Mycosphaerella fijiensis*_cds_2109bp

ATGTCGTCCGAGACATTCGAGTTCCAGGCGGAGATCTCGCAGCTTCTGTCCTTGATCATCAACACCGTCT

ACAGCAACAAGGAGATCTTCTTGCGAGAAATCATCTCCAACTCCTCCGATGCACTAGACAAGATCCGCTA

TGAAGCTCTATCAGATCCCAGCAAGCTCGACTCCGGCAAGGACCTGCGCATCGACCTCATCCCCAACAAG

GAGGCCAAGACCCTCACCATCCGCGACACTGGTATCGGTATGACCAAGGCCGACCTTGTCAACAACCTCG

GAACCATCGCCCGCTCTGGCACCAAGCAGTTCATGGAGGCCCTCTCCGCTGGCGCTGATGTTTCCATGAT

TGGTCAATTCGGTGTCGGTTTCTACTCCGCATACCTTGTCGCAGACCGCGTGACCGTCGTCTCAAAGCAC

AACGATGACGAGCAGTACATCTGGGAGTCTTCCGCTGGTGGCACTTTCTCCATCTCCCAAGACACCGACG

GCGAGCCACTCGGCCGTGGCACCAAGATCATTCTCCACCTCAAGGATGAGCAGACTGACTACCTGAACGA

GAGCAAGATCAAGGAAGTTGTGAAGAAGCACTCCGAATTCATCTCCTACCCAATCTACCTCCACGTGCTC

AAGGAGACCGAGAAGGAGGTGCCTGATGAGGAGGCCGAGGAGACCAAGGAAGAGGACGCCGACAACAAGC

CAAAGGTCGAGGAAGTTGACGACGAGGAAGAGGAGAAGAAGGAGAAGAAGACCAAGAAGATCAAGGAGTC

CAAGATTGAGGAGGAAGAGCTCAACAAGACCAAGCCCATCTGGACTCGTAACCCACAAGACATTTCGCAA

GAAGAATACGGCGCCTTCTACAAGTCGCTCAGCAATGACTGGGAAGATCACCTCGCAGTCAAGCACTTCT

CCGTTGAGGGTCAGCTCGAATTCCGCGCCATCCTCTTCGTTCCAAAGCGCGCACCATTCGACCTCTTCGA

GACGAAGAAGACTAAGAACAATATCAAGCTCTACGTACGCCGTGTCTTCATCACTGATGATGCCACCGAT

CTCGTCCCAGAGTGGCTCTCCTTCGTCAAGGGTGTCGTCGACTCTGAGGATCTCCCACTCAACCTGTCCC

GTGAGACTCTCCAACAAAACAAGATCATGAAGGTCATCAAGAAGAACATTGTCAAGAAGACTCTCGAGCT

CTTCCAGGAGATTGCCGAAGACAAGGAGCAGTTCGACAAGTTCTACAGTGCTTTCAGCAAGAACATCAAG

CTCGGTATCCACGAGGACACCCAGAACCGCCAGGCTCTTGCCAAGCTCCTCCGCTACAACTCCACCAAGT

CCACCGAGGAGACTACCTCCCTCGCCGACTACGTCACCCGCATGCCAGAGCACCAGAAGCAGATGTACTA

CATCACCGGCGAGTCCATCAAGGCTGTTGAGAAATCGCCATTCCTTGACGCCCTCAAGGCCAAGAACTTT

GAGGTCCTCTTCCTCGTTGATCCAATTGACGAGTACGCCTTCACCCAGCTCAAGGAGTATGACGGCAAGA

AGCTTGTCGACATCACTAAGGACTTCGAGCTTGAGGAGACTGAGGAAGAGAAGAAGCAGCGCGAGACGGA

GGAGAAGGAGTATGAGGCTCTCGCCAAGTCGCTCAAGAACGTTCTTGGTGACAAGGTCGAGAAGGTTGTC

GTCTCTCACAAGCTTGTCGGCTCGCCTTGTGCTATCCGTACCGGCCAGTTCGGTTGGTCTGCCAACATGG

AGCGTATCATGAAGGCTCAGGCTCTCCGTGACACGTCCATGTCCAGCTACATGAGCAGCAAGAAGACCTT

CGAGATCTCGCCCAAGAGCCCGATCATCAAGGAGCTCAAGAAGAAGGTTGAGACTGACGGCGAGAACGAC

AGCACCGTCAAGAGTATTACCACCCTTCTCTACGAGACCTCGCTGCTGGTCTCTGGCTTCACTATTGATG

AGCCAGCTAACTACGCCGAGCGCATTCACAAGCTCGTCTCCCTCGGCCTCAACGTCGACGAGGACGTCGA

GACTGCCGAGGAGGCTGCCGCACCAGCTGCTGCACCAGCAGACGGTGCCACTGAGAGCGCGATGGAGGAG

GTCGACTAA

>scaffold_1_*Mycosphaerella fijiensis*_702aa

MSSETFEFQAEISQLLSLIINTVYSNKEIFLREIISNSSDALDKIRYEALSDPSKLDSGKDLRIDLIPNK

EAKTLTIRDTGIGMTKADLVNNLGTIARSGTKQFMEALSAGADVSMIGQFGVGFYSAYLVADRVTVVSKH

NDDEQYIWESSAGGTFSISQDTDGEPLGRGTKIILHLKDEQTDYLNESKIKEVVKKHSEFISYPIYLHVL

KETEKEVPDEEAEETKEEDADNKPKVEEVDDEEEEKKEKKTKKIKESKIEEEELNKTKPIWTRNPQDISQ

EEYGAFYKSLSNDWEDHLAVKHFSVEGQLEFRAILFVPKRAPFDLFETKKTKNNIKLYVRRVFITDDATD

LVPEWLSFVKGVVDSEDLPLNLSRETLQQNKIMKVIKKNIVKKTLELFQEIAEDKEQFDKFYSAFSKNIK

LGIHEDTQNRQALAKLLRYNSTKSTEETTSLADYVTRMPEHQKQMYYITGESIKAVEKSPFLDALKAKNF

EVLFLVDPIDEYAFTQLKEYDGKKLVDITKDFELEETEEEKKQRETEEKEYEALAKSLKNVLGDKVEKVV

VSHKLVGSPCAIRTGQFGWSANMERIMKAQALRDTSMSSYMSSKKTFEISPKSPIIKELKKKVETDGEND

STVKSITTLLYETSLLVSGFTIDEPANYAERIHKLVSLGLNVDEDVETAEEAAAPAAAPADGATESAMEE

VD

>scaffold_1_*Nadsonia_fulvescens_var._elongate_DSM_6958_*cds_2088bp

ATGAGTTTGTTCATTAACACGGTCTACTCAAATAAAGAAATCTTTCTTCGTGAACTAATCTCTAATGCTT

CGGATGCTCTTGATAAAATCCGATACGAGGCACTCTCGGATCCTAAGAAATTAGAAACTGAGCCTGAGCT

CTTCATTCGATTAACTCCTAAAAAGGAGCAAAAGGTTTTGGAAATCCGTGATTCAGGTATTGGCATGACG

AAGGCTGATTTAGTAAACAATCTTGGTACTATTGCCAAGTCTGGTACTAAGGCCTTCATGGAAGCCTTAA

CCGCTGGTGCTGATATTTCCATGATTGGTCAATTTGGTGTAGGTTTCTACTCTCTTTTCTTAGTTTCGGA

TAAAGTACAAGTTATTTCCAAAAATAATGATGATGAACAATATATTTGGGAATCATCAGCCGGTGGTAAA

TTCACCGTCACTCTTGACGAAGAGAACGAGAAAATCTCCCGGGGTACCATTCTGCGCCTTTTCTTGAAAG

ATGACCAACTTGAATACCTGGAGGAGAAGAGGATCAAGGATGTGGTCAAGAAGCATTCTGAATTTGTTGC

TTACCCAATTCAATTAGTTGTAGCCAAAGAAGTTGAAAAAGAAGCTGTACCCGAAGAACCAGAAAAGGCC

CCTATCTCTGAAGATAAGGCTCCCAAAATTGAAGAAGTGGTGGATGAAGAGGGAGTAAGTGAAGAGGAAA

AAAAAGAGGGAAAAAAAATTCAAAAGATCAAAGAAATTGTAAATGAACTGGAAGAGCTTAATACTACAAA

ACCAATATGGACTAGAAACCCTTCGGATATCACCAAGGAAGAGTATGCTGCTTTTTACAAGTCGATTTCT

AATGATTGGGAAGATCACTTGGCGGTTAAGCACTTTTCAGTTGAAGGACAGTTGGAATTTAAAGCCATTC

TTTTCATTCCCAGTCGTGCTCCGTTTGATCTCTTTGAATCCAAGAAGAAAAAATCAAATATTAAACTGTA

TGTTAGAAGAGTGTTTATCACGGATGATTGCGAAGAGCTGATTCCTGAATGGCTTTCGTTTGTTAAAGGC

GTTGTGGATTCCGAGGATCTTCCGTTAAATTTGTCGCGTGAAATGCTTCAACAGAATAAGATCTTGAAGG

TTATCAGGAAAAATCTTGTCAAAAAAATTTTGGAAACTTTCGGTGAAATTGCGGAAGATCGTGAGCAATT

TGATAAATTTTATACCGCCTTCTCTAAGAATCTTAAATTAGGAATCCATGAAGATGCCCAAAACAGAAAT

GCTATTGCTAAGCTTCTCCGGTTCAATTCGACTAAATCAAACGATGAGCTGACCTCATTTGCGGACTATA

TAACTCGAATGAAAGAAAAACAGAAAAATATTTATTACATCACTGGGGAATCAATAAAAGCAGTTGAAAA

ATCACCTTTTCTTGATGCTCTCAAGAAGCGTGACTTTGAGGTTCTTTATATGGTTGATCCCATTGATGAA

TATGCTATGACCCAACAAAAAGAGTTTGATGACAAAAAACTAGTAGATATCACAAAAGACTTTGAACTGG

AAGAGAATGAAGAAGAAAAGAAATCCAGAGAAGAAGAAGAAAAGAGTTACGAGCCATTGGTCAAGTCTAT

CAAGGAAGTGCTTGGTGACAAAGTTGAAAAGGTAGTTGTTTCTTATAAGCTAATTGGGGCCCCTGCTGCT

ATCCGCACTGGTCAATTTGGTTGGTCGGCTAATATGGAGCGTATAATGAAAGCACAAGCTCTTCGGGATA

CTTCAATGTCGTCTTATATGTCAGCAAAAAAAACATTTGAAATTTCACCTCGTTCGCCCATCATTCAAGT

TATGAAGCAAAAGGTTGAAAATGATGAAGCCTCAGATAAAACCTTTAAGGATTTAACTACGCTTTTGTAT

GAAACTGCCCTTCTCACTTCAGGCTTTTCTCTTGAAGAGCCTCACTCCTTTGCCAATAGAATCAATCGCT

TGGTTTCGCTAGGGCTTAACATCGAAGAAGAAGAAGAAAATTTTGAATCACTGAAGACCGAGGACGTGGA

ACAAGAGCCAGTAGCTTCTGAGAATGTGGCAGGCTCGGCCATGGAAGAAGTTGATTAA

>scaffold_1_*Nadsonia_fulvescens_var._elongate_DSM_6958_*695aa

MSLFINTVYSNKEIFLRELISNASDALDKIRYEALSDPKKLETEPELFIRLTPKKEQKVLEIRDSGIGMT

KADLVNNLGTIAKSGTKAFMEALTAGADISMIGQFGVGFYSLFLVSDKVQVISKNNDDEQYIWESSAGGK

FTVTLDEENEKISRGTILRLFLKDDQLEYLEEKRIKDVVKKHSEFVAYPIQLVVAKEVEKEAVPEEPEKA

PISEDKAPKIEEVVDEEGVSEEEKKEGKKIQKIKEIVNELEELNTTKPIWTRNPSDITKEEYAAFYKSIS

NDWEDHLAVKHFSVEGQLEFKAILFIPSRAPFDLFESKKKKSNIKLYVRRVFITDDCEELIPEWLSFVKG

VVDSEDLPLNLSREMLQQNKILKVIRKNLVKKILETFGEIAEDREQFDKFYTAFSKNLKLGIHEDAQNRN

AIAKLLRFNSTKSNDELTSFADYITRMKEKQKNIYYITGESIKAVEKSPFLDALKKRDFEVLYMVDPIDE

YAMTQQKEFDDKKLVDITKDFELEENEEEKKSREEEEKSYEPLVKSIKEVLGDKVEKVVVSYKLIGAPAA

IRTGQFGWSANMERIMKAQALRDTSMSSYMSAKKTFEISPRSPIIQVMKQKVENDEASDKTFKDLTTLLY

ETALLTSGFSLEEPHSFANRINRLVSLGLNIEEEEENFESLKTEDVEQEPVASENVAGSAMEEVD

>scaffold_3_*Nadsonia_fulvescens_var._elongata_DSM_6958_*cds_2109bp

ATGTCCTCTGAATCGTTTGAATTCCAAGCTGAGATCTCTCAGTTGATGAGTTTGTTCATCAACACCGTCT

ACTCTAACAAAGAAATTTTCCTTAGAGAATTAATCTCCAATGCCTCAGATGCCATTGACAAGATTCGTTA

CGAAGCCTTATCTAACCCCGCTCTACTCGACACTGAACCTGAATTGTTCATTCGTTTGACCCCCAAACCT

GAAGAACACATTTTGGAAATTCGTGATTCCGGTATTGGTATGACCAAGGCCGACTTGGTCAACAACTTGG

GTACAATCGCCAAGTCCGGTACCAAGGCCTTCATGGAAGCCCTCTCTGCCGGTGCCGATGTCTCTATGAT

TGGTCAATTCGGTGTTGGTTTCTACTCTCTTTTCCTTGTCGCCGATAAGGTTCAAGTCATCTCCAAGCAC

AACGACGACGAACAATACATCTGGGAATCTTCAGCTGGTGGTAAGTTCACCGTTACCCTCGATGAAGTTA

ACGAAAGAATTACCCGTGGTACCGTTCTCCGTCTCTTCATGAAGGAAGATCAATTAGAATACCTCGAAGA

AAAGAAGATTAAGGATATTGTCAAGCGTCACTCTGAATTCGTCGCCTACCCTATTCAATTAGTCGTTACC

AAGGAAGTCGAACGTGAAGTTCCCGAAGAAGAATCTGAATCCAAGGTTGAAGAAGTTGAAGATGACAAGA

AGCCCAAGATTGAAGAAGTCGAAGATGAAGAAGATAAGAAGGAAAAGAAGACCAAGACCATCAAGGAAAC

CGTTACCGAAAACGAAGAATTAAACAAGGTCAAGCCACTTTGGACCCGTAACCCCTCCGATATCACCAAG

GAAGAATACGCTGCCTTCTACAAGTCCATCTCCAACGATTGGGAAGATCACCTTGCCGTGAAGCACTTCT

CCGTCGAAGGTCAATTAGAATTCAGAGCTATCTTATTCGTTCCTTCCCGTGCTCCCTTCGATTTATTCGA

ATCTAAGAAGAAGAAGTCTAATATCAAGCTTTACGTCCGTCGTGTCTTTATCACTGATGATGCCGAAGAA

TTAATTCCTGAATGGCTTTCTTTCGTTAAGGGTGTTGTCGACTCTGAAGATTTACCCCTTAATCTTTCCC

GTGAAATGTTACAACAAAACAAGATTCTTAAGGTTATTAGAAAGAATATCGTTAAGAAGATTCTTGAAAC

CTTCAACGAAATCGCTGAAGACCGTGAACAATTCGATAAATTCTACACTGCCTTTGGTAAGAACTTGAAG

TTGGGTATTCACGAAGATGCTCAAAACCGTACTGCTTTGGCCAAGTTATTGAGATACAACTCCACCAAGT

CTAGTGATGAATTGACCTCTTTCGCTGATTACATTACCCGCATGCCCGAACACCAAAAGAATATTTACTA

CATCACTGGTGAATCCATCAAGAGTGTTGAAAACTCCCCCTTCCTTGATGCCCTCAAGCAAAAGAACTTT

GAAGTTCTTTACCTTGTTGATCCCATTGACGAATATGCCATGACTCAACTCAAGGAATTTGAAGACAAGA

AGTTGGTGGACATCACTAAGGACTTTGAACTCGAAGAATCTGATGAAGAAAAGGCCCTTCGTGAAACTGA

AGAAAAGGAATACGAATCCCTTACCAAGTCTATTAAGGAAATCTTGGGTGAACAAGTCGAAAAGGTTGTT

GTCTCCCACAAGCTTGTGGATGCTCCGGCTGCTATCCGTACCGGTCAATTTGGCTGGTCCGCCAACATGG

AACGTATCATGAAGGCTCAAGCCCTCCGTGATACCTCTATGTCATCCTACATGGCCTCCAAGAAGACTTT

TGAAATCTCGCCACGTTCCCCCATCATTAGAGAGTTAAAGAATAAGGTTGAAGCCGATGGTGCTGATGAC

AAGACCGTTAAGGACTTGACCACCCTGCTTTATGAAACCGCTCTCTTAACCTCCGGCTTCACCCTTGAAG

AACCCAACTCTTTTGCCGCCAGAATCAATCGTTTGATTTCTCTTGGTTTGGACATTGATGTCACCGAAGA

AGAAAAGGTGTCTGAAGTTTCTGACGAAAAGGTCGAAGATGAAGTTGTTGCCGATTCCGCCATGGAAGAA

GTTGATTAA

>scaffold_3_*Nadsonia_fulvescens_var._elongata_DSM_6958_*702aa

MSSESFEFQAEISQLMSLFINTVYSNKEIFLRELISNASDAIDKIRYEALSNPALLDTEPELFIRLTPKP

EEHILEIRDSGIGMTKADLVNNLGTIAKSGTKAFMEALSAGADVSMIGQFGVGFYSLFLVADKVQVISKH

NDDEQYIWESSAGGKFTVTLDEVNERITRGTVLRLFMKEDQLEYLEEKKIKDIVKRHSEFVAYPIQLVVT

KEVEREVPEEESESKVEEVEDDKKPKIEEVEDEEDKKEKKTKTIKETVTENEELNKVKPLWTRNPSDITK

EEYAAFYKSISNDWEDHLAVKHFSVEGQLEFRAILFVPSRAPFDLFESKKKKSNIKLYVRRVFITDDAEE

LIPEWLSFVKGVVDSEDLPLNLSREMLQQNKILKVIRKNIVKKILETFNEIAEDREQFDKFYTAFGKNLK

LGIHEDAQNRTALAKLLRYNSTKSSDELTSFADYITRMPEHQKNIYYITGESIKSVENSPFLDALKQKNF

EVLYLVDPIDEYAMTQLKEFEDKKLVDITKDFELEESDEEKALRETEEKEYESLTKSIKEILGEQVEKVV

VSHKLVDAPAAIRTGQFGWSANMERIMKAQALRDTSMSSYMASKKTFEISPRSPIIRELKNKVEADGADD

KTVKDLTTLLYETALLTSGFTLEEPNSFAARINRLISLGLDIDVTEEEKVSEVSDEKVEDEVVADSAMEE

VD

>scaffold_1_*Phanerochaete_chrysosporium*_cds_2106bp

ATGGCCACTGAGAGCTTCGGCTTCCAGGCTGAGATCTCACAGCTCTTGGACCTAATTATCAACACTTTCT

ACTCCAACAAGGAGATCTTCTTGCGGGAGCTCATCTCTAACGCTTCGGATGCTCTCGACAAGATCCGCTA

CCAGTCGCTCACCGACCCCTCGGTGCTCGACTCGGGCAAGGACCTCTACATCCGCATTACCCCTGACAAG

GAGAATAAGGTCCTCTCCATCCGGGATACTGGTATCGGCATGACCAAGGCCGACATGGTTAACAATCTCG

GTACGATCGCGAAGTCCGGCACCAAGGGCTTCATGGAAGCTCTTAGCTCCGGTGCCGATATCTCGATGAT

CGGCCAGTTCGGTGTCGGTTTCTACTCCGCGTACCTCGTCGCCGAGCGTGTGCAGGTCATCTCGAAGCAC

AATGACGATGAGCAGTACATCTGGGAGTCGTCTGCCGGCGGTACCTTCACTGTCACGCCCGACCACGTCA

ACCCTCCGCTTGGCCGTGGTACCGAGATCCGCCTTTTCCTCAAGGAGGACCAGCTTGAGTACCTCGAGGA

GAAGCGTATCAAGGACATCGTCAAGAAGCACTCTGAGTTCATCTCGTACCCGATCCAGCTTACCGTCACT

AAGGAGGTCGAGAAGGAGGTCGAGGATGACGAGGAGATGAAGGAGGACAAAGAGGAGGACAAGGAGAAGC

CGAAGGTCGAGGAGGTCGACGAGGAGGAAGATGAAAAGAAGAAGAAGACTAAGAAGGTCAAGGAGAAGAC

CGTCTCTCAGGAGGAACTCAATAAGACGAAGCCGATCTGGACTCGCAACCCGTCTGAGATCACTCCTGAA

GAGTACGCCTCCTTCTACAAGAGCTTGACTAACGACTGGGAGGACCACCTCGCCGTGAAGCACTTCTCAG

TTGAAGGTCAGCTCGAGTTCAAGGCTATCCTCTTCGTGCCGAGGCGCGCGCCGTTCGACCTCTTCGAGAC

CAAGCGCAAGCGCAACAACATCAAGCTCTATGTTCGCCGCGTCTTCATCATGGACGACTGCGAGGATCTC

ATTCCCGAGTACCTCAACTTCGTCAAGGGTATCGTCGACTCCGAGGATCTGCCACTCAACATCTCGCGTG

AGACGCTTCAGCAGAACAAGATCCTCAAGGTCATCCGCAAGAACATCGTTAAGAAGTGCCTCGACTTGTT

CTCGGAGATTGCTGAGGATAAGGACAACTTCAACAAGTTCTACGAGGCCTTCAGCAAGAACATCAAGCTC

GGTATCCACGAGGACGCTCAGAACCGGAGCAAGCTTGCCGAGTTCTTGCGCTTCTACTCGACCAAGTCCC

TCGATGAGATGACTTCGCTGAAGGACTACATCACTCGCATGCCTGAGGTCCAGAAGAACATCTACTACCT

CACCGGCGAGTCGCTCTCCGCCCTCAAGGATTCTCCGTTCCTGGAGGTCTTCAAGCGCAAGGGCTTCGAG

GTCCTCCTCCTTGTCGACCCGATCGACGAGTACGCCATCACTCAGCTCAAGGAGTTCGACGGTCACAAGC

TCGTCTGTGTCTCCAAGGAGGGTCTCGAGCTCGAGGAGACCGAGGAGGAGAAGAAGGCGCGCGAGGAGGA

GGCCAAGCAGTTCGAGGACCTCTGCAAGGCTGTCAAGGAGGCTCTCGGCGACAAGGTCGAGAAGGTCGTC

GTCTCCAACCGCATCACCGACTCGCCATGCGTCCTCGTCACCGGTCAGTTCGGCTGGTCGTCGAACATGG

AGCGCATCATGAAGGCGCAGGCGCTTCGCGACTCGTCGATGTCGTCGTACATGGCTTCGAAGAAGACGCT

CGAGCTCAACCCACACAACGCCATCATCAAGGAGCTCAAGAAGAAGGTTGCCGAGGACAAGGCCGACAAG

TCCGTCCGCGACCTCACGTACCTCCTGTTCGAGACTGCGCTCCTCACCTCCGGCTTCTCGCTCGACGACC

CGACATCGTTCGCGAAGCGCATCCACCGTATGATTGCTCTCGGCCTGGACGTCGAGGACGAGCCCGAGGC

ACAACCAGCCGCAAGCACTTCGGAGGTTCCTCCAGCAGATGCTGCGTCGGCGTCTGCGATGGAGGAGATC

GACTAA

>scaffold_1_*Phanerochaete_chrysosporium*_701aa

MATESFGFQAEISQLLDLIINTFYSNKEIFLRELISNASDALDKIRYQSLTDPSVLDSGKDLYIRITPDK

ENKVLSIRDTGIGMTKADMVNNLGTIAKSGTKGFMEALSSGADISMIGQFGVGFYSAYLVAERVQVISKH

NDDEQYIWESSAGGTFTVTPDHVNPPLGRGTEIRLFLKEDQLEYLEEKRIKDIVKKHSEFISYPIQLTVT

KEVEKEVEDDEEMKEDKEEDKEKPKVEEVDEEEDEKKKKTKKVKEKTVSQEELNKTKPIWTRNPSEITPE

EYASFYKSLTNDWEDHLAVKHFSVEGQLEFKAILFVPRRAPFDLFETKRKRNNIKLYVRRVFIMDDCEDL

IPEYLNFVKGIVDSEDLPLNISRETLQQNKILKVIRKNIVKKCLDLFSEIAEDKDNFNKFYEAFSKNIKL

GIHEDAQNRSKLAEFLRFYSTKSLDEMTSLKDYITRMPEVQKNIYYLTGESLSALKDSPFLEVFKRKGFE

VLLLVDPIDEYAITQLKEFDGHKLVCVSKEGLELEETEEEKKAREEEAKQFEDLCKAVKEALGDKVEKVV

VSNRITDSPCVLVTGQFGWSSNMERIMKAQALRDSSMSSYMASKKTLELNPHNAIIKELKKKVAEDKADK

SVRDLTYLLFETALLTSGFSLDDPTSFAKRIHRMIALGLDVEDEPEAQPAASTSEVPPADAASASAMEEI

D

>scaffold_8_*Phanerochaete_chrysosporium*_cds_2100bp

ATGGCCTCCGAGAGCTTTGGCTTCCAGGCTGAGATCTCCCAGCTCCTCGATCTCATCATCAACACCTTCT

ACTCCAACAAGGAGATCTTCCTTCGCGAGCTCATCTCCAACGCGTCCGATGCCCTCGACAAGATCCGCTA

CCAGTCGTTGACCGACCCCTCCGTCCTTGACTCGGGCAAGGACCTCTTCATCCGCATCACCCCTGACAAG

GAGAACAAGATCCTCTCCATCCGCGATACCGGTATCGGTATGACCAAGGCGGACATGGTCAACAACCTCG

GTACGATCGCCAAGTCCGGCACCAAGGGCTTCATGGAGGCTCTGAGCTCCGGCGCCGACATCTCCATGAT

CGGTCAGTTCGGTGTCGGTTTCTACTCCGCGTACCTCGTCGCCGAGCGCGTCCAGGTCATCTCCAAGCAC

AATGACGACGAGCAGTACATCTGGGAGTCTGCTGCGGGTGGTACTTTCACCATCACCCCCGACACCGTCA

ACCCCCCTCTCGGCCGCGGTACCGAGATCCGCCTCTACCTCAAGGAGGACCAGCTCGAGTACCTTGAGGA

GAAGCGCATCAAGGAGATCGTGAAGAAGCACTCCGAGTTCATCTCCTACCCCATTCAGTTGGCGGTGACC

AAGGAGGTCGAGAAGGAGGTTGAGGACGAGGAGGAGGCCGCCGAGGAGGAGGACAAGGAGAAGCCAAAGG

TCGAGGAGGTTGATGACGAGGAGGACAAGAAGAAGAAGACCAAGAAGGTCAAGGAGAAGACCGTCGAGAA

CGAGGAGCTCAACAAGACCAAGCCCATCTGGACGCGCAACCCATCCGAGATCACCGCTGAGGAGTACGCT

GCGTTCTACAAGAGCTTGTCGAACGACTGGGAGGAGCACCTCGCTGTCAAGCACTTCTCCGTCGAGGGTC

AGCTCGAGTTCAAGGCGATCCTCTTCATCCCCAAGCGTGCTCCCTTCGACCTCTTCGAGTCCAAGAAGAA

GCGCAACAACATCAAGCTCTACGTTCGCCGCGTCTTCATCATGGACGACTGCGAGGACCTCATCCCCGAG

TACCTCAACTTCGTCAAGGGTATCGTCGACTCTGAGGACCTTCCTCTCAACATCTCGCGTGAGACCCTTC

AGCAAAACAAGATTCTCAAGGTCATCCGCAAGAACATTGTCAAGAAGTGCATGGACCTGTTCTCCGAGAT

CGCCGAGGACAAGGACAACTTCAACAAGTTCTACGAGGCGTTCGGCAAGAACATCAAGCTCGGTATTCAC

GAGGATGCTCAGAACCGCAGCAAGCTCGCCGAGTTCCTGCGCTTCTACTCGACCAAGTCCTCTGACGAGA

TGACCTCGCTCAAGGACTACATCACTCGCATGCCCGAGGTCCAGAAGAACATCTACTACCTCACCGGCGA

GTCGCTCTCCGCGGTCAAGGACTCGCCCTTCCTCGAGGTCCTCAAGAAGAAGGGCTTCGAGGTCCTCCTC

CTTGTCGACCCCATCGACGAGTACGCCATCACCCAGCTCAAGGAGTTCGACGGCCACAAGCTTGTCTGTG

TCTCCAAGGAGGGTCTCGAGCTCGAGGAGACCGAGGAGGAGAAGAAGGCGCGCGAGGAGGAGGCCAAGCA

GTTCGAGGACCTCTGCAAGGCTGTCAAGGAGGCTCTCGGTGACAAGGTCGAGAAGGTCGTTGTCTCCAAC

CGTATTTCCGACTCGCCCTGCGTGCTCGTCACCGGCCAGTTCGGCTGGTCGTCGAACATGGAGCGTATCA

TGAAGGCGCAGGCGCTCCGCGACTCGTCGATGTCGTCGTACATGGCCTCGAAGAAGACGCTCGAGCTCAA

CCCGCACAACCCGATCGTCAAGGAGCTCAAGGCCAAGGTCGCCGAGGACAAGGCCGACAAGTCTGTCCGC

GACCTCACGTACCTGCTCTTCGAGACCGCGCTCCTCACTTCCGGCTTCTCGCTCGACGACCCGACGTCGT

TCGCGAAGCGCATCCACCGCATGATCGCACTCGGCCTCGACGTCGACGAGGAGGAGTCTGCGCCCGCCGC

AAGCTCGTCGTCGGACGTCCCCCCGCCGGTGGAGGCTGCGTCCTCGTCCGCGATGGAGGAGATTGACTAG

>scaffold_8*_Phanerochaete_chrysosporium*_699aa

MASESFGFQAEISQLLDLIINTFYSNKEIFLRELISNASDALDKIRYQSLTDPSVLDSGKDLFIRITPDK

ENKILSIRDTGIGMTKADMVNNLGTIAKSGTKGFMEALSSGADISMIGQFGVGFYSAYLVAERVQVISKH

NDDEQYIWESAAGGTFTITPDTVNPPLGRGTEIRLYLKEDQLEYLEEKRIKEIVKKHSEFISYPIQLAVT

KEVEKEVEDEEEAAEEEDKEKPKVEEVDDEEDKKKKTKKVKEKTVENEELNKTKPIWTRNPSEITAEEYA

AFYKSLSNDWEEHLAVKHFSVEGQLEFKAILFIPKRAPFDLFESKKKRNNIKLYVRRVFIMDDCEDLIPE

YLNFVKGIVDSEDLPLNISRETLQQNKILKVIRKNIVKKCMDLFSEIAEDKDNFNKFYEAFGKNIKLGIH

EDAQNRSKLAEFLRFYSTKSSDEMTSLKDYITRMPEVQKNIYYLTGESLSAVKDSPFLEVLKKKGFEVLL

LVDPIDEYAITQLKEFDGHKLVCVSKEGLELEETEEEKKAREEEAKQFEDLCKAVKEALGDKVEKVVVSN

RISDSPCVLVTGQFGWSSNMERIMKAQALRDSSMSSYMASKKTLELNPHNPIVKELKAKVAEDKADKSVR

DLTYLLFETALLTSGFSLDDPTSFAKRIHRMIALGLDVDEEESAPAASSSSDVPPPVEAASSSAMEEID

>scaffold_18_*Phlebia_brevispora*_HHB-7030_SS6_cds_2100bp

ATGGCTTCTGAGAGCTTTGGCTTCCAGGCTGAGATCAGTCAGCTCCTGGACCTGATCATCAACACCTTCT

ACTCCAACAAGGAGATCTTCCTTCGTGAAATAATCTCGAACGCCTCCGATGCGCTCGACAAGGTTAGGTA

CCAGTCGCTCACGGACCCGTCTGTGCTCGATTCTGGCAAGGATCTGTACATCCGCATCATCCCCGATAAG

GAGAATAAGACCCTTTCCATTCGCGATACTGGTATCGGTATGACTAAGGCGGACATGGTAAACAACCTTG

GTACCATCGCGAAGTCGGGTACGAAGGGCTTCATGGAGGCTCTGTCATCTGGCGCAGACATTTCCATGAT

TGGTCAGTTCGGTGTCGGTTTCTACTCTGCGTACCTCGTCGCCGAGCGTGTGCAGGTCATCTCGAAACAC

AACGATGACGAGCAATACATCTGGGAGTCCGCGGCTGGTGGTACCTTTACCATCACCCCTGACCATTTCA

ACCCACCTCTTGGCCGTGGTACCGAGGTCCGCCTTTACCTCAAGGAGGACCAGCTTGAGTACCTCGAGGA

GAAGCGTATTAAAGATATCGTCAAGAAGCACTCCGAGTTCATCTCGTACCCGATCCAGCTTGCGGTGACC

AAGGAGGTTGAGAAGGAGGTCGAGGACGAAGAGCAGGAAGAGACCAAGGAGGGTGAGGAGAAGCCCAAGA

TCGAGGAGGTCGAGGAGGACGAGGAGAAGAAGAAGAAGACGAAGAAGATCAAGGAGAAGACTGTTGAGAA

TGAGGAGCTCAACAAGACGAAGCCCATCTGGACGCGTAACCCTCAGGACATCACTCAGGAGGAATACGCT

GCGTTCTACAAGAGCTTGACGAACGACTGGGAGGAGCACTTGGCCGTGAAGCACTTCTCTGTTGAGGGTC

AGCTCGAGTTTAAGGCTATCCTCTACGTGCCCAAGCGTGCACCCTTCGACCTCTTCGAGACGAAGAAGAA

GCGCAACAACATCAAGCTCTACGTCCGTCGCGTCTTCATCATGGACGACTGCGAGGACCTCATCCCCGAG

TACTTGAACTTCGTAAAGGGTATCGTCGACTCCGAAGATCTTCCTCTCAACATCTCCCGCGAGACTCTCC

AGCAAAACAAGATCCTCAAGGTCATCCGCAAGAACATCGTCAAGAAGTGCATGGACTTGTTCAGCGAGAT

CGCAGAGGACAAGGACAACTTCAACAAGTTCTACGAGGCCTTCGGCAAGAACATCAAGCTCGGTATCCAC

GAGGATGCTCAGAACCGTAGCAAGCTCGCCGAGTTCTTGCGCTTCTACTCGACCAAGTCTACCGAGGAGT

TGACCTCCCTGAAGGATTACATCACTCGCATGCCCGAGGTTCAGAAGAACATCTACTACCTCACTGGCGA

ATCTCTCTCCGCCGTCAAGGACTCACCGTTCCTTGAGGTGCTCAAGAAGAAGGGCTTCGAAGTTCTCCTT

CTCGTCGACCCCATCGACGAATATGCGATCACTCAGCTCAAGGAGTTTGAGAACCACAAGCTCATCTGTG

TGTCCAAGGAGGGCCTCGAGCTCGAGGAGACCGAGGAGGAGAAGAAGGAGCGCGAGGAGGAGGCCAAGCA

GTTTGAGGATCTCTGCAAGGCCGTCAAGGACGCGCTCGGCGACAAGGTGGAGAAGGTCGTCGTCTCAAAC

CGTATCACCGATTCGCCCTGCGTACTGGTCACTGGCCAGTTCGGCTGGTCGTCGAACATGGAGCGTATCA

TGAAGGCGCAGGCGCTCCGTGACTCGTCGATGTCGTCGTATATGGCCTCGAAGAAGACCCTTGAGCTTAA

CCCCCACAACGCGATCGTGAAGGAGCTCAAGAAGAAGGTCGCCGAGGACAAGGCGGACAAGTCTGTCCGC

GACCTTACCTTCCTGCTCTTCGAGACTGCACTCCTCACCTCTGGTTTCTCGCTTGAGGACCCAACGTCAT

TCGCTAAGCGCATCCACCGCATGATTGCCCTTGGCCTCGACGTCGATGAGGACGAGGAGGCTCCTGCGGC

AGCCGCGGAGACCGCTGAAGTTCCCGCAGCAGAGGCAGCTGCCACCTCTGCCATGGAGGAGATTGACTAG

>scaffold_18_*Phlebia_brevispora*_HHB-7030_SS6_699aa

MASESFGFQAEISQLLDLIINTFYSNKEIFLREIISNASDALDKVRYQSLTDPSVLDSGKDLYIRIIPDK

ENKTLSIRDTGIGMTKADMVNNLGTIAKSGTKGFMEALSSGADISMIGQFGVGFYSAYLVAERVQVISKH

NDDEQYIWESAAGGTFTITPDHFNPPLGRGTEVRLYLKEDQLEYLEEKRIKDIVKKHSEFISYPIQLAVT

KEVEKEVEDEEQEETKEGEEKPKIEEVEEDEEKKKKTKKIKEKTVENEELNKTKPIWTRNPQDITQEEYA

AFYKSLTNDWEEHLAVKHFSVEGQLEFKAILYVPKRAPFDLFETKKKRNNIKLYVRRVFIMDDCEDLIPE

YLNFVKGIVDSEDLPLNISRETLQQNKILKVIRKNIVKKCMDLFSEIAEDKDNFNKFYEAFGKNIKLGIH

EDAQNRSKLAEFLRFYSTKSTEELTSLKDYITRMPEVQKNIYYLTGESLSAVKDSPFLEVLKKKGFEVLL

LVDPIDEYAITQLKEFENHKLICVSKEGLELEETEEEKKEREEEAKQFEDLCKAVKDALGDKVEKVVVSN

RITDSPCVLVTGQFGWSSNMERIMKAQALRDSSMSSYMASKKTLELNPHNAIVKELKKKVAEDKADKSVR

DLTFLLFETALLTSGFSLEDPTSFAKRIHRMIALGLDVDEDEEAPAAAAETAEVPAAEAAATSAMEEID

>scaffold_27_*Phlebiopsis_gigantea*_cds_2094bp

ATGGCCACCGAGAGCTTTGGCTTCCAGGCTGAGATCTCTCAGCTTTTGGACTTGATCATCAACACCTTCT

ACTCCAACAAGGAGATCTTCCTGCGAGAACTCATCTCCAACGCCTCGGATGCTCTCGACAAGATCCGCTA

CCAGTCATTGACGGACCCCTCGGTCCTTGACACTGGCAAGGAGCTCTTCATCCGTATCGTCCCCGACAAG

GAGAACAAAATATTGTCCATCCGGGATGCTGGTATTGGTATGACCAAGGCGGACATGGTCAACAACCTGG

GTACCATCGCCAAGTCTGGCACAAAGGGCTTCATGGAGGCTCTCAGCTCCGGTGCCGACATTTCCATGAT

TGGTCAATTCGGTGTCGGGTTCTACTCCGCGTATCTCGTCGCCGAGCGTGTCCAGGTCATCTCCAAGCAC

AACGACGACGAGCAGTACATTTGGGAGTCTGCCGCTGGTGGCACTTTCACCATCACCACTGACACTGTCA

ATCCGGCTCTTGGTCGTGGTACCGAGATCCGCCTCTACCTCAAGGAAGATCAGCTCGAATATCTTGAGGA

GAAGCGCATCAAGGATATTGTCAAGAAACATTCTGAGTTCATCTCTTATCCTATTCAGCTGGCGGTGACC

AAGGAGGTTGAGAAGGAAGTTGAGGACGAGGAAGAGGTCTCTGAGGACTCCGAGGACAAGCCCAAGATCG

AGGAGGTCGACGAGGATGAGGACAAGAAGAAGAAGACCAAGACGATCAAGGAGAAGACAGTTGAGAACGA

GGAGCTCAACAAGACCAAGCCCATCTGGACACGCAACCCCCAGGACATCACCTCTGAAGAGTACGCTGCA

TTCTACAAGAGCTTGTCCAACGACTGGGAGGAACACCTCGCTGTCAAGCATTTCTCAGTCGAGGGCCAGC

TTGAGTTCAAGGCTATTCTCTACATTCCGAAGCGTGCTCCTTTCGACCTCTTTGAGACAAAGAAGAAGCG

TAACAACATTAAGCTCTATGTCCGCCGTGTGTTCATCATGGACGACTGCGAGGACATCATTCCCGAGTAT

CTCAACTTCGTGAAGGGTATCGTCGACTCCGAGGACCTTCCCCTTAACATCTCCCGCGAGACCCTGCAAC

AGAACAAGATCCTCAAGGTCATCCGTAAGAACATCGTGAAGAAGTGCATGGACCTCTTCTCCGAGATCGC

TGAGGACAAGGATAATTTCGCGAAGTTCTATGAGGCGTTCGGCAAGAACATGAAGCTCGGTATCCACGAG

GACTCCCAGAACCGCAGCAAGCTCGCCGAGTTCTTGCGCTTCTACTCGACGAAGTCGACTGAGGAACTCA

CATCGCTGAAGGACTACATCACGCGCATGCCTGAGGTCCAGAAGAACATCTACTACCTCACTGGCGAGTC

TCTGTCGGCTGTCAAGGAGTCGCCCTTCCTTGAGGTTCTGAAGAAGAAGGGCTTCGAGGTCCTCCTCATG

GTTGACCCCATCGACGAATACGCTGTTACCCAGCTCAAGGAGTTCGACGGCCACAAGCTCATTTGCGTGT

CAAAGGAAGGCCTCGAGCTTGAGGAAACTGAGGAGGAGAAGAAGGCCCGTGAGGAGGAGGCCACTCAGTT

CGAGGACCTCTGCAAAGCCGTCAAAGAGGCACTCGGCGACAAGGTTGAGAAGGTCATTGTCTCCAACCGT

ATCTCCGACTCTCCTTGCGTTCTCGTCACCGGCCAGTTCGGCTGGTCGTCAAACATGGAGCGTATCATGA

AGGCGCAGGCGCTCCGCGATTCTTCCATGTCGTCGTACATGGCGTCTAAGAAGACTCTTGAGCTCAACCC

GCACAATCCCATTGTAAAGGAACTCAAGAGCAAGGTCGCCGAGGACAAGGCCGACAAATCTGTCCGCGAT

CTCACCTACCTCCTCTTCGAGACCGCTCTCCTCACCTCCGGCTTCTCGCTTGACGATCCAACATCGTTCG

CCAAGCGCATTCACCGCATGATCGCTCTTGGTCTCGATGTCGACGAGGAGGCTGAGGTTCCTGCCGCGTC

TACGTCTGAGGTTCCTCCGCCAGCAGAAGCCTCCGGCGCGTCTGCGATGGAGGAGATCGACTAA

>scaffold_27_*Phlebiopsis_gigantea*_697aa

MATESFGFQAEISQLLDLIINTFYSNKEIFLRELISNASDALDKIRYQSLTDPSVLDTGKELFIRIVPDK

ENKILSIRDAGIGMTKADMVNNLGTIAKSGTKGFMEALSSGADISMIGQFGVGFYSAYLVAERVQVISKH

NDDEQYIWESAAGGTFTITTDTVNPALGRGTEIRLYLKEDQLEYLEEKRIKDIVKKHSEFISYPIQLAVT

KEVEKEVEDEEEVSEDSEDKPKIEEVDEDEDKKKKTKTIKEKTVENEELNKTKPIWTRNPQDITSEEYAA

FYKSLSNDWEEHLAVKHFSVEGQLEFKAILYIPKRAPFDLFETKKKRNNIKLYVRRVFIMDDCEDIIPEY

LNFVKGIVDSEDLPLNISRETLQQNKILKVIRKNIVKKCMDLFSEIAEDKDNFAKFYEAFGKNMKLGIHE

DSQNRSKLAEFLRFYSTKSTEELTSLKDYITRMPEVQKNIYYLTGESLSAVKESPFLEVLKKKGFEVLLM

VDPIDEYAVTQLKEFDGHKLICVSKEGLELEETEEEKKAREEEATQFEDLCKAVKEALGDKVEKVIVSNR

ISDSPCVLVTGQFGWSSNMERIMKAQALRDSSMSSYMASKKTLELNPHNPIVKELKSKVAEDKADKSVRD

LTYLLFETALLTSGFSLDDPTSFAKRIHRMIALGLDVDEEAEVPAASTSEVPPPAEASGASAMEEID

>scaffold_35_*Phlebiopsis_gigantea*_cds_2100bp

ATGTATTTGCGCGACCGCGGCGCCGCAGCTGCTTTCTCGTCGCCTGCTTTCCTGCACACCTTCTACTCCA

ACAAGGAGATCTTCCTTCGAGAGCTCATCTCCAACGCGTCCGATGCTCTCGACAAGATTCGGTACCAATC

CTTGACGGATCCGTCGGTCCTTGACTCTGGCAAGGACCTCATTATCCGTATCATCCCCGACAAGGAGAAC

AAGATCCTCTCCCTTCGCGATACCGGTATAGGCTTGACCAAGGCCGACATGGTCAACAACCTCGGCACCA

TCGCAAAGTCCGGCACAAAGGGCTTCATGGAGGCTCTTACCTCTGGTGCCGATATTTCCATGATCGGCCA

GTTCGGTGTCGGCTTCTACTCCGCGTACCTCGTCGCCGAGCGTGTGCAGGTCATCTCGAAGCACAACGAC

GACGAGCAGTATATCTGGGAGTCCGCGGCCGGTGGCACCTTCACCATCACCGCCGATACCGTCAACCCCC

CACTCGGCCGTGGTACCGAGATCCGCCTCTACCTGAAGGAGGATCAGCTCGAGTACCTCGAGGAAAAACG

CATCAAGGACATTGTCAAGAAGCACTCCGAGTTCATCTCGTACCCCATTCAGTTGGCGGTGACGAAGGAG

GTTGAGAAGCTTCAACAGGAAGTCGAAGACGACGAGGAGGTCGCTGAAGAGGAGTCAGAAGACAAGCCCA

AGATCGAGGAAGTCGACGACGAGGAGGACAAGAAGAAGAAGACGAAGAAGATCAAGGAGAAGACCGTCGA

GAATGAGGAACTGAACAAGACCAAGCCCATCTGGACGCGCAACCCTCAGGACATCACGGCGGAGGAGTAT

GCCGCGTTCTACAAGAGTTTGTCGAACGACTGGGAGGAGCACCTCGCCGTGAAACACTTCTCTGTGGAGG

GTCAGCTCGAGTTCAAGGCTATCCTCTTCATTCCTAAGCGCGCTCCGTTCGACCTCTTCGAGACTAAGAA

GAAGCGTAACAACATCAAGCTCTACGTCCGCCGCGTCTTCATCATGGACGACTGCGAGGACATTATTCCC

GAGTACCTCAACTTCGTCAAGGGTATCGTCGACTCCGAGGACCTGCCACTCAACATCTCCCGCGAGACCC

TTCAACAGAACAAGATCCTCAAGGTGATCCGCAAGAACATCGTCAAAAAGTGCATGGACCTCTTCTCCGA

AATCGCCGAGGACAAGGACAACTTCGCCAAGTTCTACGAGGCGTTCGGCAAGAACATCAAGCTCGGTGTA

CACGAGGATTCGCAGAACCGTAGCAAACTCGCCGAATTCCTGCGCTTCTACTCGACAAAGTCGACTGAGG

AGTTGACCTCGCTGAAGGACTACATCACTCGTATGCCCGAGGTCCAGAAGAACATTTACTACCTCACCGG

CGAGTCGCTGTCTTCTGTCAAGGAATCACCGTTCCTTGAGGTACTCAAGAAGAAGGGCTTCGAGGTCCTA

CTCATGGTCGACCCCATCGACGAGTACGCCGTCACCCAGCTCAAGGAGTTCGACGGCCACAAACTCGTCT

GTGTCTCCAAGGAGGGTCTCGAGCTCGAGGAGACCGACGAGGAGAAGAAGGCTCGCGAGGAGGAGGCGAA

GCAATTCGAGGACCTCTGCAAGGCGGTGAAGGAAGCCCTCGGCGACAAGGTTGAGAAGGTCGTCGTTTCC

AACCGTATCTCCGACTCCCCCTGCGTTCTCGTCACCGGCCAGTTCGGTTGGTCGTCGAACATGGAGCGCA

TCATGAAGGCGCAGGCGCTGCGCGACTCCTCCATGTCGTCGTACATGGCGTCGAAGAAGACCCTCGAGCT

CAACCCGCACAATCCCATCGTCCGCGAGCTCAAGAGCAAGGTTGCCGAGGACAAGGCGGACAAGTCTGTT

CGCGACCTCACCTATCTCCTCTTCGAGACCGCTCTTCTCACCTCTGGCTTCTCCCTGGACGACCCCACCT

CGTTCGCGAAGCGTATCCACCGCATGATCGCCCTTGGTCTCGATGTTGACGAGGACGCGGAAGTATCCGC

GCCCACACCTGCCTCGGAGGCACCGATAGAGGAGGCCGTTGGCGCGTCGGCGATGGAGGAGATTGACTAG

>scaffold_35_*Phlebiopsis_gigantea*_699aa

MYLRDRGAAAAFSSPAFLHTFYSNKEIFLRELISNASDALDKIRYQSLTDPSVLDSGKDLIIRIIPDKEN

KILSLRDTGIGLTKADMVNNLGTIAKSGTKGFMEALTSGADISMIGQFGVGFYSAYLVAERVQVISKHND

DEQYIWESAAGGTFTITADTVNPPLGRGTEIRLYLKEDQLEYLEEKRIKDIVKKHSEFISYPIQLAVTKE

VEKLQQEVEDDEEVAEEESEDKPKIEEVDDEEDKKKKTKKIKEKTVENEELNKTKPIWTRNPQDITAEEY

AAFYKSLSNDWEEHLAVKHFSVEGQLEFKAILFIPKRAPFDLFETKKKRNNIKLYVRRVFIMDDCEDIIP

EYLNFVKGIVDSEDLPLNISRETLQQNKILKVIRKNIVKKCMDLFSEIAEDKDNFAKFYEAFGKNIKLGV

HEDSQNRSKLAEFLRFYSTKSTEELTSLKDYITRMPEVQKNIYYLTGESLSSVKESPFLEVLKKKGFEVL

LMVDPIDEYAVTQLKEFDGHKLVCVSKEGLELEETDEEKKAREEEAKQFEDLCKAVKEALGDKVEKVVVS

NRISDSPCVLVTGQFGWSSNMERIMKAQALRDSSMSSYMASKKTLELNPHNPIVRELKSKVAEDKADKSV

RDLTYLLFETALLTSGFSLDDPTSFAKRIHRMIALGLDVDEDAEVSAPTPASEAPIEEAVGASAMEEID

>scaffold_2_*Phycomyces_blakesleeanus*_NRRL1555_cds_2115bp

ATGGTTGAAACAGAAACATTTTCCTTCCAGGCTGAGATCTCTCAGTTGATGAGCTTGATCATCAATACAT

TCTACTCAAATAAGGAAATCTTCTTGCGTGAATTGATCTCAAACTCCTCTGATGCACTGGACAAGATCCG

TTACCAATCCTTGACAGATCCTTCTGTCTTGGACTCTGAAAAGGAATTCTTCATCCGTATCACCCCCGAC

AAAGAGAACAAAGTTCTTTCCATCCGTGATTCCGGTATCGGCATGACCAAAGCCGACCTGGTCAACAACT

TGGGTACAATTGCCAAATCTGGTACCAAGGCATTCATGGAAGCACTTTCCTCAGGCGCAGACATCTCCAT

GATCGGTCAGTTTGGTGTCGGTTTCTACTCTGCATATCTCGTAGCAGACAAAGTCCAGGTCATAACCAAG

CACAATGATGACGAACAGTACATCTGGGAGTCTGCCGCTGGTGGCTCGTTTACCATCACTCGCGATGAGG

TCAATCCTTCCATTGGTCGTGGCACTGAAATGCGCCTCTTCATGAAGGAGGATCAGTTAGAATACCTTGA

GGAGAAGAAGATCAAGGATATCGTAAAGAAACACTCAGAGTTTATCTCCTACCCTATCCAGCTTGTAGTT

GAAAAGGAAGTCGAGAAGGAAGTCTCAGATGACGAAGAAATGAAGGAGGCTGATTCCGAAGAAAAGCCCA

AAATTGAGGAGATCGAAGACGAAGATGACAAGAAAGAGGAAAAGAAAAAGAAAACCATCAAGGAAACCGT

CACTGAGACTGAGGAGCTCAACAAGACCAAGCCTCTTTGGACCCGAAACCCTGAAGAAGTCAAGCTCGAG

GAGTACACCCAATTCTACAAAGCTCTCACTAATGACTGGGAAGACCATCTGGCTGTTAAGCACTTTTCTG

TCGAAGGTCAGCTCGAATTCCGTGCTATCTTGTTTGTTCCCAAGCGTGCTCCCTTTGACATGTTTGAGAC

CAAGAAGAAGCGCAACAACATCAAGCTTTACGTCCGTCGCGTGTTTATCATGGATGACTGTGATGAGCTG

ATCCCCGAATGGCTTAACTTTGTCAAGGGTGTTGTTGACTCTGAAGATCTTCCTCTCAATATTTCGCGTG

AGATGCTCCAGCAAAACAAGATTCTCAAAGTAATCCGCAAGAATCTCGTCAAGAAATGCCTTGAGATGTT

TGCTGAGATTGCTGAAGACAAAGAAAACTTTGACAAGTTCTATGAGGCTTTCTCCAAGAACCTCAAATTG

GGTATCCATGAAGACACTCAAAACCGTGCCAAGATTGCCGACCTTTTGCGTTACAGCTCTACCAAATCAG

GAGATGAAATGACTTCTCTAAAAGACTATGTTACCCGTATGCACGAGAAGCAAAAGAACATCTACTATAT

TACTGGCGAGTCTCGTGCTTCCATTGAAAACTCACCCTTCCTTGAAGGGTTTAAGAAAAAGAACATCGAG

GTTTTGTTAATGACTGACCCAATTGATGAATATAGCACTCAGCAATTGAAAGAATACGATGGCAAGCCCC

TTGTCTGTATCACCAAGGAAGGTGTCGAGATCGAAGAAGATGAAGATGAAAAGAAGGCGCGAGAGGAAGA

AGAAAAGAAGTGCGAAGGTCTCTGCAAGGCCGTTAAGGAGATTCTTGGCGACAAGGTCGAAAAGGTCGTT

GTCTCTAACAAGCTTACTGACTCTCCGTGTGTCCTCACGACCGGTCAATTCGGTTGGTCTGCCAACATGG

AGCGTATCATGAAAGCCCAGGCTCTTCGTGACTCTTCCATGTCATCTTACATGGCTTCTAAGAAGACTTT

GGAGCTTAACCCTCATCATGCTATTATCAAGAGTCTTGCTGCCAAGGTTGAGGCTGATAGTGGTGACCGA

ACTGTCAAAGACTTGACTACACTCTTGTACGAGACTTCGTTGCTCACCTCTGGCTTCTCTTTGGACGACC

CCAGCTCATTTGCTCACCGTATCCACCGCATGGTTGCTCTTGGTCTCAGCATTGACGAAGAAGATTTGCC

TACAGCCGAGACTGACGATGCACCTCCTGCTGAGTCAGCGTCTGCGTCTGCAGAGGAATCCTCTAAGATG

GAAGAAGTCGATTAA

>scaffold_2_*Phycomyces_blakesleeanus*_NRRL1555_704aa

MVETETFSFQAEISQLMSLIINTFYSNKEIFLRELISNSSDALDKIRYQSLTDPSVLDSEKEFFIRITPD

KENKVLSIRDSGIGMTKADLVNNLGTIAKSGTKAFMEALSSGADISMIGQFGVGFYSAYLVADKVQVITK

HNDDEQYIWESAAGGSFTITRDEVNPSIGRGTEMRLFMKEDQLEYLEEKKIKDIVKKHSEFISYPIQLVV

EKEVEKEVSDDEEMKEADSEEKPKIEEIEDEDDKKEEKKKKTIKETVTETEELNKTKPLWTRNPEEVKLE

EYTQFYKALTNDWEDHLAVKHFSVEGQLEFRAILFVPKRAPFDMFETKKKRNNIKLYVRRVFIMDDCDEL

IPEWLNFVKGVVDSEDLPLNISREMLQQNKILKVIRKNLVKKCLEMFAEIAEDKENFDKFYEAFSKNLKL

GIHEDTQNRAKIADLLRYSSTKSGDEMTSLKDYVTRMHEKQKNIYYITGESRASIENSPFLEGFKKKNIE

VLLMTDPIDEYSTQQLKEYDGKPLVCITKEGVEIEEDEDEKKAREEEEKKCEGLCKAVKEILGDKVEKVV

VSNKLTDSPCVLTTGQFGWSANMERIMKAQALRDSSMSSYMASKKTLELNPHHAIIKSLAAKVEADSGDR

TVKDLTTLLYETSLLTSGFSLDDPSSFAHRIHRMVALGLSIDEEDLPTAETDDAPPAESASASAEESSKM

EEVD

>scaffold_20_*Phycomyces_blakesleeanus*_NRRL1555_cds_2094bp

ATGACTGTCGAAACCGAGACCTTTTCTTTCCAGGCTGAGATCTCTCAGTTGATGAGCTTGATCATCAACA

CCTTCTACTCTAACAAGGAAATTTTCTTGCGTGAGTTGATCTCCAACTCTTCCGATGCTATCGACAAGAT

CCGTTACCAGTCTTTGACCGACTCATCCCTCTTGGACTCCGAGAAGGAGCTCTTCGTCCGTATCACCCCC

GACAGAGAGAACAACATTCTCTCCATCCGTGATTCCGGTATCGGTATGACCAAGGCTGATCTCGTCAACA

ACCTCGGTACTATTGCCAAGTCTGGTACCAAGGCTTTCATGGAGGCTCTCTCTTCCGGTGCTGACATTTC

CATGATTGGTCAGTTCGGCGTCGGTTTCTACTCTGCCTACCTTGTTGCCGACAAGGTCCAGGTCATCACC

AAGCACAACGATGATGAGCAGTACATCTGGGAGTCTGCTGCCGGTGGTTCCTTCACCATCACCCGTGATG

AGGTCAACCCCTCCATTGGTCGCGGTACTGAGATGCGCCTCTTCATGAAGGAGGACCAGCTCGAATACCT

CGAGGAGAAGAAGATTAAGGACATCGTCAAGAAGCACTCCGAGTTCATCTCCTACCCCATCCAGCTCGTC

GTCGAGAAGGAAGTCGAGAAGGAGGTCTCCGATGATGAAGAGGAGGTCAAGGAAGGCGAGAAGCCCAAGA

TTGAGGAAATCGAAGATGAGGATGACAAGAAGGACAAGAAGAAGAAGACCATCAAGGAGAAGTCCACCGA

GACCGAGGAGCTCAACAAGACCAAGCCCCTCTGGACCCGCAACCCCGAGGATGTCAAGGCTGATGAGTAC

TCTCAGTTCTACAAGGCCCTCACCAACGACTGGGAAGACCATCTTGCCGTCAAGCACTTCTCTGTCGAAG

GTCAGCTCGAGTTCCGTGCCATCCTCTTCATCCCCAAGCGTGCTCCCTTTGACATGTTCGAGACCAAGAA

GAAGCGTAACAACATCAAGCTCTACGTCCGTCGTGTCTTCATCATGGACGACTGTGAGGACCTCATCCCC

GAGTGGCTCAACTTCGTCAAGGGTGTTGTTGACTCTGAGGATCTCCCCCTCAACATCTCCCGTGAGATGC

TCCAGCAGAACAAGATCCTCAAGGTCATCCGTAAGAACCTCGTCAAGAAGTGTCTCGAGATGTTCGCTGA

GATCGCCGAGGACAAGGAGAACTTTGACAAGTTCTACGAGTCCTTCTCCAAGAACCTCAAGTTGGGTATC

CACGAGGACACCCAGAACCGCGGTAAGATCGCCGAGCTCCTCCGTTACTCCTCTACCAAGTCTGGTGAGG

AGTTGACCTCCCTCAAGGACTACGTTACCCGTATGCCCGAGAAGCAGAAGAACATCTACTACATCACTGG

CGAGTCCCGCGCTGCCATTGAGAACTCTCCTTTCCTCGAAGGTTTCAAGAAGAAGAACGTCGAAGTCTTG

TTGATGACCGACCCCATTGATGAGTACAGCACTCAGCAATTGAAGGAGTACGATGGCAAGACCCTCGTCT

GCATCACCAAGGAAGGTGTTGAGATCGAAGAGGATGAGGAGGAGAAGAAGGCCCGTGAAGAGGAGGAGAA

GAAGTTCGAGGGTCTCTGCAAGGCTGTCAAGGAGATCCTTGGCGAGAAGGTCGAGAAGGTCGTCATCTCC

AACAAGTTGACTGACTCTCCCTGTGTCCTCACCACTGGTCAGTTCGGCTGGTCTGCCAACATGGAGCGTA

TCATGAAGGCCCAGGCTCTCCGTGACTCTTCCATGTCCTCTTACATGGCTTCCAAGAAGACCTTTGAGTT

GAACCCTGAGCATGCCATCATCAAGAGCCTCGCCACCAAGATTGCTGCTGACAGCAACGACCGTTCCGTC

AAGGATTTGACCACTCTCCTCTACGAGACCTCTTTGTTGACCTCCGGTTTCTCCCTTGATGAGCCCAGCT

CATTCGCCAACCGCATTCACCGCATGGTTTCCCTCGGTCTCAGCATCGATGAGGAGGAGCTCCCTACCCA

GAGCAACTCTGAAGTCCCTGCTCTCGAGACCATCGAGACCTCCAAGATGGAGGAAGTCGATTAA

>scaffold_20_*Phycomyces_blakesleeanus*_NRRL1555_697aa

MTVETETFSFQAEISQLMSLIINTFYSNKEIFLRELISNSSDAIDKIRYQSLTDSSLLDSEKELFVRITP

DRENNILSIRDSGIGMTKADLVNNLGTIAKSGTKAFMEALSSGADISMIGQFGVGFYSAYLVADKVQVIT

KHNDDEQYIWESAAGGSFTITRDEVNPSIGRGTEMRLFMKEDQLEYLEEKKIKDIVKKHSEFISYPIQLV

VEKEVEKEVSDDEEEVKEGEKPKIEEIEDEDDKKDKKKKTIKEKSTETEELNKTKPLWTRNPEDVKADEY

SQFYKALTNDWEDHLAVKHFSVEGQLEFRAILFIPKRAPFDMFETKKKRNNIKLYVRRVFIMDDCEDLIP

EWLNFVKGVVDSEDLPLNISREMLQQNKILKVIRKNLVKKCLEMFAEIAEDKENFDKFYESFSKNLKLGI

HEDTQNRGKIAELLRYSSTKSGEELTSLKDYVTRMPEKQKNIYYITGESRAAIENSPFLEGFKKKNVEVL

LMTDPIDEYSTQQLKEYDGKTLVCITKEGVEIEEDEEEKKAREEEEKKFEGLCKAVKEILGEKVEKVVIS

NKLTDSPCVLTTGQFGWSANMERIMKAQALRDSSMSSYMASKKTFELNPEHAIIKSLATKIAADSNDRSV

KDLTTLLYETSLLTSGFSLDEPSSFANRIHRMVSLGLSIDEEELPTQSNSEVPALETIETSKMEEVD

>scaffold_515_*Piromyces_sp._E2*_cds_2091bp

ATGGCTGAATCCGAAACATTTGCCTTCCAAGCTGAAATCTCTCAATTAATGAGTTTGATTATCAACACTT

TCTACTCTAACAAGGAAATTTTCTTAAGAGAACTTATTTCTAACGCTTCTGATGCTTTAGATAAGATTAG

ATACCAATCTCTTACTGATGCTTCCCAACTTGATACCGAAAAGGAACTTTTCATTCGTATTACTCCAGAC

AAAGAAAACAAGATTCTTGAAATCAGAGATTCTGGTATTGGTATGACCAAGGCCGATCTTGTCAACAATC

TTGGTACCATTGCCAAGTCTGGTACTAAGGCCTTCATGGAAGCTCTTCAATCTGGTGCTGATATTTCTAT

GATTGGTCAATTTGGTGTCGGTTTCTACTCTGCTTACTTAGTTGCCGATAAGGTTCAAGTTATCACCAAG

CACAATGATGATGAACAATACATCTGGGAATCTGCTGCTGGTGGTAGTTTCACCATTACTCGTGATACCG

TCAATGAAAAGATTGGTCGTGGTTCCATTATCAGACTTTTCATGAAGGAAGACCAACTTGAATACCTTGA

AGAAAAGCGTATTAAGGAAATTGTTAAGAAGCACTCTGAATTCATTGGTTACCCAATTCAATTAGTTGTT

GAAAAGGAAGTTGAAAAGGAAGTTGAAGATGAAGAAGAAGAAGCTAAGGAAGAAGATGGTCCAAAGATTG

AAGAAATTGATGAAGAAAAGGAAAAGGAAGAAAAGAAGAAGAAGACCATCAAGGAAATCAAGAAGGAAAC

TGAAGAATTAAACAAGACCAAGCCACTCTGGACCAGAAACCCAGATGACATTACCAACGAAGAATACGGT

GCCTTCTACAAGAGCATTACCAACGATTGGGAAGACCATCTTGCTGTCAAGCACTTCTCTGTTGAAGGTC

AATTAGAATTCCGTGCCTTATTATTCGTTCCACGTCGTGCTCCATTCGATATGTTTGAACAAAAGAAGAA

GAGAAACAACATTAAGCTTTACGTCCGTCGTGTCTTCATTATGGATGACTGTGAAGAATTAATCCCAGAA

TGGCTTAACTTCATTAAGGGTGTTGTCGATTCTGAAGATCTTCCACTTAACATTTCTCGTGAAATGTTAC

AACAAAACAAGATCTTAAAGGTTATCCGTAAGAACCTTGTCAAGAAGTGCATTGAATTATTCAATGAAAT

TTACGAAGATGCCGAAAACAAGAAGAAGTTCTACGAAGCCTTCTCCAAGAACATTAAGTTAGGTATTCAC

GAAGACAGCACCAACCGTGCCAAGTTATCTGAATTCCTTATGTTCTACTCTACCAAGTCTGGTGAAGAAA

TGACTTCTCTTAAGGACTACGTTACTCGTATGCCAGAAAAGCAAAAGAACATTTACTACGTCACTGGTGA

ATCCAAGGCTGCTGTCGAAAACTCTCCATTCCTTGAAGCTCTCAAGAAGAAGGGATTCGAAGTTTTATAC

CTTGTTGATCCAATTGATGAATACATGGTTCAACAACTTAAGGAATACGATGGTCACACTCTCCAAAGTG

TTACCAAGGAAGGTCTTGAATTAGAAGAAGACGAAGATGAAAAGAAGAAGCGTGAAGAAGAAAAGGCTCA

ATTCGAAGAATTATGTAAGACCATTAAGGAAATTCTTGGTGACAAGGTTGAAAAGGTTGTCCTTTCTAAC

CGTATTGTTAACTCTCCATGTGTTTTAGTTACTGGTCAATACGGTTGGTCTGCTAACATGGAACGTATCA

TGAGAGCTCAAGCTCTTCGTGATTCTGCTATGTCTGCTTACATGGCCTCCAAGAAGACTATGGAAATTAA

CCCAGAACACGCTATCATCAAGTCTCTTAAGGCTAAGGACAAGAACGACAAGACCTTAAAGGATCTTGTC

TTCTTATTATACGAAACTGCCTTATTATCTTCTGGTTTCACTCTTGAAGATCCATCCAACTTTGCCTCTC

GTATTCACCGTATGATTAAGTTAGGTTTAAGTATTGATGAAGGCGAAGAAGCTGAAATGGAAACTGATGC

TCAAGATGATATGCCAGAATTAGAAGAAGTTGGTGAATCTAAGATGGAAGAAGTTGATTAA

>scaffold_515_*Piromyces_sp._E2*_696aa

MAESETFAFQAEISQLMSLIINTFYSNKEIFLRELISNASDALDKIRYQSLTDASQLDTEKELFIRITPD

KENKILEIRDSGIGMTKADLVNNLGTIAKSGTKAFMEALQSGADISMIGQFGVGFYSAYLVADKVQVITK

HNDDEQYIWESAAGGSFTITRDTVNEKIGRGSIIRLFMKEDQLEYLEEKRIKEIVKKHSEFIGYPIQLVV

EKEVEKEVEDEEEEAKEEDGPKIEEIDEEKEKEEKKKKTIKEIKKETEELNKTKPLWTRNPDDITNEEYG

AFYKSITNDWEDHLAVKHFSVEGQLEFRALLFVPRRAPFDMFEQKKKRNNIKLYVRRVFIMDDCEELIPE

WLNFIKGVVDSEDLPLNISREMLQQNKILKVIRKNLVKKCIELFNEIYEDAENKKKFYEAFSKNIKLGIH

EDSTNRAKLSEFLMFYSTKSGEEMTSLKDYVTRMPEKQKNIYYVTGESKAAVENSPFLEALKKKGFEVLY

LVDPIDEYMVQQLKEYDGHTLQSVTKEGLELEEDEDEKKKREEEKAQFEELCKTIKEILGDKVEKVVLSN

RIVNSPCVLVTGQYGWSANMERIMRAQALRDSAMSAYMASKKTMEINPEHAIIKSLKAKDKNDKTLKDLV

FLLYETALLSSGFTLEDPSNFASRIHRMIKLGLSIDEGEEAEMETDAQDDMPELEEVGESKMEEVD

>contig_60_*Saccharomyces_bayanus*_MCYC_623_cds_2121bp

ATGGCTGGTGAAACCTTTGAATTTCAAGCAGAAATCACTCAGTTGATGAGTTTGATCATCAACACTGTCT

ATTCTAACAAGGAGATTTTCTTGAGAGAACTGATATCCAACGCCTCAGATGCGTTAGACAAGATCAGGTA

CAAAGCTTTGTCCGACCCAAAGCAACTGGAAACTGAACCTGAATTATTCATTAGAATCACTCCAAGACCA

GAAGAAAAGGTCTTGGAAATCAGGGATTCCGGTATTGGTATGACCAAGGCGGAATTGATCAATAACTTAG

GTACCATCGCCAAGTCTGGTACCAAGGCCTTCATGGAAGCTCTTTCTGCTGGTGCCGATGTCTCCATGAT

CGGTCAATTCGGTGTTGGTTTCTACTCCTTATTCTTAGTCGCTGATAGAGTCCAAGTTATCTCTAAGAAC

AACGACGATGAACAATACATCTGGGAGTCCAACGCAGGTGGTTCTTTCACTGTTACATTAGACGAAGTTA

ACGAAAAGATCGGTAGAGGTACAGTCTTGAGGTTGTTCATGAAGGACGACCAATTGGAGTACCTGGAGGA

AAAGAAGATTAAGGAAGTCATCAAGAGACACTCCGAATTTGTTGCCTACCCAATCCAGTTACTAGTCACC

AAGGAAGTCGAAAAGGAAGTTCCAGTCCCAGAAGAAGAAGAAGAAAAGAAGGACGAAGAAAAGAAGGATG

AAGATGACAAGAAACCAAAATTGGAAGAAGTCGACGAAGAAGAAGAAAAGAAGCCAAAGACCAAGAAAGT

CAAGGAAGAAGTTCAAGAATTAGAAGAACTAAACAAGACTAAGCCTTTATGGACCAGAAACCCATCCGAA

ATTACGCAAGAAGAATACAATGCTTTCTACAAATCCATCTCCAACGACTGGGAAGACCCACTATATGTTA

AGCATTTCTCTGTCGAAGGTCAATTAGAATTCAGAGCTATCTTGTACATTCCAAAGAGAGCTCCATTTGA

CCTATTCGAAAGTAAGAAGAAGAAGAACAACATTAAGTTGTACGTTCGTCGTGTTTTCATCACCGACGAA

GCAGAAGACTTGGTTCCAGAATGGATGTCATTCGTCAAGGGTGTTGTTGACTCCGAAGATTTACCATTGA

ACTTGTCCAGAGAAATGTTGCAACAAAACAAGATCATGAAGGTCATCAGAAAGAACATCGTTAAGAAGAT

GATTGAATCCTTCAACGAAATCGCTGAAGACTCTGAACAATTCGAAAAGTTCTACTCTGCCTTCGCTAAG

AACATCAAATTGGGTGTTCATGAAGACACTCAAAACAGAGCTGCCTTGGCCAAATTGCTACGTTACAACT

CCACCAAGTCCGTTGACGAATTGACTTCTTTGACTGATTATATTACCAGAATGCCAGAACACCAAAAGAA

CATCTACTATATCACAGGTGAGTCTTTGAAGGCTGTTGAAAAATCCCCATTCTTAGACGCTTTGAAAGCT

AAGAACTTTGAAGTTTTGTTCTTGACTGATCCAATCGATGAATACGCCTTCACTCAATTAAAGGAATTCG

AAGGTAAGACTTTAGTCGATATCACCAAGGATTTCGAGCTGGAAGAAACTGACGAAGAGAAAGCTGAAAG

AGAAAAGGAAGTTAAAGAATTCGAACCATTGACCAAGGCCTTGAAAGACATCTTGGGTGAACAAGTTGAA

AAGGTTGTTGTCTCTTACAAACTAGTGGATGCCCCAGCTGCCATTAGAACTGGCCAATTCGGTTGGTCCG

CTAACATGGAAAGAATCATGAAGGCTCAAGCTTTGAGAGACTCTTCCATGTCCTCTTACATGTCCTCCAA

GAAGACTTTCGAAATCTCTCCAAAATCTCCAATTATCAAGGAATTGAAAAAGAGAGTTGATGAAGGCGGT

GCTCAAGATAAGACTGTCAAGGATTTGACCAACTTATTATTCGAAACCGCTCTGTTAACTTCTGGTTTCA

GTCTGGAAGAACCAACCTCGTTTGCTTCAAGAATTAACAGATTGATTTCTTTGGGTTTGAACATTGATGA

AGAAGAAGAGACTGAAGCTGCTACTGATGCAGCTACTGATGCTCCAGTCGAAGAAGTCCCAGCTGACACC

GAAATGGAAGAAGTCGATTAG

>contig_60_*Saccharomyces_bayanus*_MCYC_623_706aa

MAGETFEFQAEITQLMSLIINTVYSNKEIFLRELISNASDALDKIRYKALSDPKQLETEPELFIRITPRP

EEKVLEIRDSGIGMTKAELINNLGTIAKSGTKAFMEALSAGADVSMIGQFGVGFYSLFLVADRVQVISKN

NDDEQYIWESNAGGSFTVTLDEVNEKIGRGTVLRLFMKDDQLEYLEEKKIKEVIKRHSEFVAYPIQLLVT

KEVEKEVPVPEEEEEKKDEEKKDEDDKKPKLEEVDEEEEKKPKTKKVKEEVQELEELNKTKPLWTRNPSE

ITQEEYNAFYKSISNDWEDPLYVKHFSVEGQLEFRAILYIPKRAPFDLFESKKKKNNIKLYVRRVFITDE

AEDLVPEWMSFVKGVVDSEDLPLNLSREMLQQNKIMKVIRKNIVKKMIESFNEIAEDSEQFEKFYSAFAK

NIKLGVHEDTQNRAALAKLLRYNSTKSVDELTSLTDYITRMPEHQKNIYYITGESLKAVEKSPFLDALKA

KNFEVLFLTDPIDEYAFTQLKEFEGKTLVDITKDFELEETDEEKAEREKEVKEFEPLTKALKDILGEQVE

KVVVSYKLVDAPAAIRTGQFGWSANMERIMKAQALRDSSMSSYMSSKKTFEISPKSPIIKELKKRVDEGG

AQDKTVKDLTNLLFETALLTSGFSLEEPTSFASRINRLISLGLNIDEEEETEAATDAATDAPVEEVPADT

EMEEVD

>contig_936_*Saccharomyces_bayanus*_MCYC_623_cds_2127bp

ATGGCTGGTGAAACTTTTGAATTTCAAGCTGAAATTACTCAGTTGATGAGTTTGATCATCAACACTGTCT

ATTCTAACAAGGAAATTTTCTTGAGAGAACTGATCTCCAATGCTTCCGATGCTTTGGACAAGATCAGGTA

CAAAGCTTTGTCCGACCCAAAGGAATTGGACTCTGAACCTGAATTATTCATTAGAATCACTCCAAGACCA

GAAGAAAAGGTCTTGGAAATCAGAGATTCCGGTATTGGTATGACCAAGGCAGAATTGATCAACAATTTGG

GTACTATCGCTAAGTCTGGTACTAAGGCCTTTATGGAAGCTCTTTCTGCTGGTGCCGATGTCTCCATGAT

CGGTCAATTCGGTGTTGGTTTCTACTCCTTATTCTTGGTCGCCGATAGAGTCCAAGTTATCTCTAAGAAC

AACGACGATGAACAATACATCTGGGAGTCCAATGCAGGTGGTTCTTTCTCTGTTACTCTAGACGAAGTTA

ACGAAAGTATCGGTAGAGGTACTGTCTTGAGATTATTCTTGAAGGATGACCAATTGGAATACTTAGAAGA

AAAAAAGATCAAGGAAGTCATCAAGAGACACTCCGAATTTGTTGCTTATCCAATCCAATTGCTAGTCACC

AAGGAAGTCGAAAAGGAAGTTCCAATCCCAGAAGAAGAAGAAGAAAAGAAGGATGAAGAAAAGAAGGATG

AAGATGACAAGAAACCAAAATTGGAAGAAGTCGACGAAGAAGAAGAAGAAAAGAAGCCAAAGACCAAGAA

AGTCAAGGAAGAAGTTCAAGAATTAGAAGAACTAAACAAGACTAAGCCTCTATGGACCAGAAACCCATCC

GAAATCACCGAAGAAGAATACAATGCCTTCTACAAATCCATTTCCAACGACTGGGAAGACCCACTATATG

TTAAGCACTTCTCTGTCGAAGGTCAACTAGAATTCAGAGCTATCTTGTACATTCCAAAGAGAGCTCCATT

TGACCTTTTCGAAAGTAAGAAGAAGAAGAACAACATCAAGTTATACGTTCGTCGTGTTTTCATTACCGAT

GAAGCTGAAGACTTGATTCCAGAATGGATGTCCTTCGTCAAGGGTGTTGTTGACTCCGAAGATTTACCAT

TGAACTTGTCCAGAGAAATGTTACAACAAAACAAGATCATGAAGGTTATCAGAAAGAACATCGTTAAGAA

GTTGATTGAATCCTTCAACGAAATCGCTGAAGACTCTGAACAATTCGAAAAGTTCTACTCTGCCTTCGCT

AAGAACATCAAATTGGGTGTTCATGAAGACACTCAAAACAGAGCTGCCTTGGCTAAATTGCTACGTTACA

ACTCCACCAAGTCCGTTGACGAATTAACTTCTTTGACTGATTACATTACCAGAATGCCAGAACACCAAAA

GAACGTTTACTATATCACTGGTGAATCTCTAAAGGCCGTTGAAAAATCCCCATTCTTGGACGCTTTGAAG

GCTAAGAACTTTGAAGTTTTGTTCTTGACTGATCCAATCGATGAATACGCTTTCACTCAATTGAAGGAAT

TCGAAGGTAAGACTTTGGTCGATATCACCAAGGATTTCGAATTGGAAGAAACTGACGAAGAAAAAGCTGA

AAGAGAGAAGGAAGTTAAAGAATACGAACCATTGACCAAGGCCTTGAAAGACATCTTGGGTGAACAAGTT

GAAAAGGTTGTTGTCTCTTACAAACTAGTGGATGCTCCAGCTGCCATTAGAACTGGCCAATTCGGTTGGT

CCGCTAACATGGAAAGAATCATGAAGGCTCAAGCTTTGAGAGACTCTTCCATGTCCTCTTACATGTCCTC

CAAGAAGACTTTCGAAATCTCTCCAAAATCTCCAATTATCAAGGAATTGAAAAAGAGAGTTGATGAAGGT

GGTGCTCAAGATAAGACTGTCAAGGATTTGACCAACTTATTATTCGAAACTGCTTTGTTAACTTCTGGTT

TCAGTTTGGAAGAACCAACCTCTTTCGCCTCAAGAATTAACAGATTGATTTCTTTGGGTTTGAACATTGA

TGAAGAAGAAGAAGAAACTGAAGCTGCTACTGATGCTGCTACCGATGCTCCAGTCGAAGAAGTCCCAGCT

GACACCGAAATGGAAGAAGTTGATTAA

>contig_936_*Saccharomyces_bayanus*_MCYC_623_708aa

MAGETFEFQAEITQLMSLIINTVYSNKEIFLRELISNASDALDKIRYKALSDPKELDSEPELFIRITPRP

EEKVLEIRDSGIGMTKAELINNLGTIAKSGTKAFMEALSAGADVSMIGQFGVGFYSLFLVADRVQVISKN

NDDEQYIWESNAGGSFSVTLDEVNESIGRGTVLRLFLKDDQLEYLEEKKIKEVIKRHSEFVAYPIQLLVT

KEVEKEVPIPEEEEEKKDEEKKDEDDKKPKLEEVDEEEEEKKPKTKKVKEEVQELEELNKTKPLWTRNPS

EITEEEYNAFYKSISNDWEDPLYVKHFSVEGQLEFRAILYIPKRAPFDLFESKKKKNNIKLYVRRVFITD

EAEDLIPEWMSFVKGVVDSEDLPLNLSREMLQQNKIMKVIRKNIVKKLIESFNEIAEDSEQFEKFYSAFA

KNIKLGVHEDTQNRAALAKLLRYNSTKSVDELTSLTDYITRMPEHQKNVYYITGESLKAVEKSPFLDALK

AKNFEVLFLTDPIDEYAFTQLKEFEGKTLVDITKDFELEETDEEKAEREKEVKEYEPLTKALKDILGEQV

EKVVVSYKLVDAPAAIRTGQFGWSANMERIMKAQALRDSSMSSYMSSKKTFEISPKSPIIKELKKRVDEG

GAQDKTVKDLTNLLFETALLTSGFSLEEPTSFASRINRLISLGLNIDEEEEETEAATDAATDAPVEEVPA

DTEMEEVD

>contig_10_*Saccharomyces*_*mikatae*_IFO_1815_cds_2121bp

ATGGCAGGTGAAACTTTTGAATTTCAAGCAGAAATTACTCAGTTGATGAGTTTAATTATCAACACTGTCT

ACTCTAACAAGGAAATTTTCTTGAGAGAACTGATATCCAATGCCTCAGATGCTTTAGACAAGATCAGGTA

TAAGTCTTTGTCTGACCCAAAACAATTAGAAACGGAACCTGATCTGTTCATCAGAATCACGCCAAAACCA

GAGGAAAAAGTTTTGGAAATCAGAGATTCTGGTGTTGGTATGACAAAGGCAGAACTGATCAATAACTTGG

GTACGATTGCCAAGTCCGGTACTAAAGCTTTCATGGAAGCCCTCTCTGCTGGTGCCGATGTGTCTATGAT

TGGTCAGTTTGGTGTCGGTTTTTACTCTTTATTCTTGGTCGCTGACAGAGTCCAAGTTATTTCAAAGAGT

AATGACGACGAACAATACATTTGGGAGTCGAACGCGGGTGGTTCTTTCACTGTTACTCTAGATGAGGTCA

ATGAAAAAATTGGCAGAGGTACCGTTTTAAGATTATTCTTAAAGGATGACCAACTGGAATACTTGGAGGA

AAAGAGAATAAAGGAAGTTATTAAGAGACACTCCGAATTTGTTGCTTACCCAATTCAGTTGGTTGTCACT

AAGGAAGTGGAAAAAGAAGTTCCAATTCCAGAGGAAGAAAAGAATAAAGAGGAAAAGAAGGAGGAGGATG

ACAAGAAACCAAAACTAGAAGAAGTCGATGAAGAAGAAGAAGAAGAAAAGAAGCCAAAGACAAAGAAAGT

CAAGGAAGAAGTTCAAGAAATAGAAGAACTGAATAAGACTAAACCATTGTGGACTAGAAACCCATCTGAT

ATCACTCAAGAAGAATACAATGCTTTTTACAAATCTATTTCTAACGACTGGGAAGACCCATTATATGTTA

AGCATTTCTCTGTTGAAGGTCAATTGGAATTCAGAGCCATCTTATTCATTCCGAAGAGAGCTCCATTCGA

CTTGTTTGAAAGTAAGAAGAAGAAGAATAACATTAAGCTATACGTACGTCGTGTTTTCATAACTGATGAA

GCTGAAGACTTGATTCCAGAATGGTTATCTTTCGTTAAGGGTGTTGTTGACTCTGAGGATTTGCCATTGA

ACTTATCCAGAGAGATGCTACAACAAAACAAGATCATGAAGGTTATTAGAAAGAACATTGTCAAGAAGTT

GATTGAAGCTTTTAACGAAATTGCTGAAGACTCTGAACAATTTGACAAATTTTACTCTGCCTTTTCCAAG

AACATTAAATTGGGTGTACATGAAGACACTCAAAACAGAGCTGCCTTGGCTAAATTGCTACGTTACAACT

CCACCAAGTCCGTCGACGAATTGACCTCATTGACCGACTATGTCACCAGAATGCCTGAACACCAAAAGAA

CATTTACTATATCACCGGTGAATCTCTAAAGGCTGTCGAAAAGTCTCCATTCTTGGATGCTTTAAAGGCT

AAGAACTTTGAGGTTTTGTTCTTGACTGATCCAATTGATGAATACGCCTTCACTCAGTTGAAGGAGTTTG

AAGGTAAGACTTTGGTTGACATTACTAAAGACTTCGAATTGGAAGAAACTGACGAGGAGAAGGCACAAAG

AGAGAAAGAAATAAAAGAATACGAACCATTAACAAAAGCTTTGAAGGACATCTTGGGTGACCAAGTGGAA

AAGGTTGTTGTTTCTTACAAATTGTTGGACGCTCCAGCTGCCATCAGAACTGGTCAGTTCGGCTGGTCCG

CTAACATGGAGAGAATTATGAAGGCCCAGGCCTTGAGAGATTCCTCCATGTCCTCATATATGTCTTCCAA

GAAGACTTTTGAAATATCTCCAAGATCGCCAATTATAAAGGAATTGAAAAAGAGAGTCGACGAAGGTGGT

GCTCAGGATAAAACTGTCAAAGATTTGACTAACCTATTATTTGAGACTGCTTTGTTGACTTCTGGTTTCA

CTTTAGATGAACCAACTTCGTTTGCTTCCAGAATCAACAGGTTGATCTCCTTAGGTTTGAATATCGATGA

TGATGGTGAAGAAACTGGAGAGACTGAAGCTACCGCTGAAGCTCCTGTCGAAGAAGTTCCGGCTGACACC

GAAATGGAAGAAGTCGATTAG

>contig_10_*Saccharomyces*_*mikatae*_IFO_1815_706aa

MAGETFEFQAEITQLMSLIINTVYSNKEIFLRELISNASDALDKIRYKSLSDPKQLETEPDLFIRITPKP

EEKVLEIRDSGVGMTKAELINNLGTIAKSGTKAFMEALSAGADVSMIGQFGVGFYSLFLVADRVQVISKS

NDDEQYIWESNAGGSFTVTLDEVNEKIGRGTVLRLFLKDDQLEYLEEKRIKEVIKRHSEFVAYPIQLVVT

KEVEKEVPIPEEEKNKEEKKEEDDKKPKLEEVDEEEEEEKKPKTKKVKEEVQEIEELNKTKPLWTRNPSD

ITQEEYNAFYKSISNDWEDPLYVKHFSVEGQLEFRAILFIPKRAPFDLFESKKKKNNIKLYVRRVFITDE

AEDLIPEWLSFVKGVVDSEDLPLNLSREMLQQNKIMKVIRKNIVKKLIEAFNEIAEDSEQFDKFYSAFSK

NIKLGVHEDTQNRAALAKLLRYNSTKSVDELTSLTDYVTRMPEHQKNIYYITGESLKAVEKSPFLDALKA

KNFEVLFLTDPIDEYAFTQLKEFEGKTLVDITKDFELEETDEEKAQREKEIKEYEPLTKALKDILGDQVE

KVVVSYKLLDAPAAIRTGQFGWSANMERIMKAQALRDSSMSSYMSSKKTFEISPRSPIIKELKKRVDEGG

AQDKTVKDLTNLLFETALLTSGFTLDEPTSFASRINRLISLGLNIDDDGEETGETEATAEAPVEEVPADT

EMEEVD

>contig_448_*Saccharomyces*_*mikatae*_IFO_1815_cds_2133bp

ATGGCTGGTGAAACTTTTGAATTTCAAGCTGAAATTACTCAGTTGATGAGTTTGATCATCAACACTGTCT

ATTCTAACAAGGAAATTTTCTTGAGAGAACTGATATCCAACGCTTCCGATGCTTTAGACAAGATCAGATA

CCAGGCTTTGTCTGACCCAAAGCAATTGGAAACTGAACCAGATTTGTTCATTAGAATTACACCAAGACCA

GAGGAAAAAGTTTTGGAAATCAGAGATTCTGGTATTGGTATGACCAAGGCGGAATTGATTAATAACTTGG

GTACGATTGCCAAATCCGGTACTAAAGCTTTCATGGAAGCTCTCTCTGCTGGTGCAGATGTCTCTATGAT

TGGTCAATTTGGTGTCGGTTTCTACTCTTTATTCTTGGTCGCTGACAGAGTCCAAGTTATCTCCAAGAAC

AACGATGATGAGCAATATATTTGGGAATCCAATGCCGGTGGTTCTTTCACTGTTACTTTGGACGAAGTAA

ATGAAAGAATTGGTAGAGGTACTGTCTTGAGATTATTCCTGAAAGATGACCAATTGGAATACTTGGAAGA

AAAGAGAATTAAGGAAGTCATTAAGAGACATTCTGAATTTGTTGCTTACCCTATCCAACTTTTAGTCACC

AAAGAGGTTGAAAAGGAAGTTCCAATTCCAGAAGAAGAAAAGAAGGATGAAGAAAAGAAGGATGAGGAAA

AGAAGGATGAAGATGACAAAAAGCCAAAGTTGGAAGAAGTCGATGAAGAAGAAGAAGAAAAGAAGCCAAA

AACTAAAAAGGTTAAGGAGGAAGTTCAAGAATTAGAAGAATTAAACAAGACTAAACCATTATGGACTAGA

AACCCATCTGAAATCACTCAAGAAGAATACAATGCTTTCTACAAGTCTATTTCCAACGACTGGGAAGACC

CATTATACGTTAAGCATTTCTCTGTCGAAGGTCAATTAGAATTCAGAGCTATCTTATTCATTCCAAAGAG

AGCTCCATTCGACTTGTTTGAAAGTAAGAAGAAGAAGAATAACATTAAGCTATATGTTCGTCGTGTTTTC

ATCACTGATGAAGCTGAAGACTTGATTCCAGAATGGTTATCTTTCGTCAAGGGTGTTGTTGACTCTGAGG

ATTTACCATTGAACTTATCCAGAGAAATGTTGCAACAAAATAAGATCATGAAGGTTATTAGAAAGAACAT

TGTCAAGAAGTTGATTGAATCATTTAACGAAATTGCTGAAGACTCTGAGCAATTTGACAAGTTTTACTCT

GCCTTTGCTAAGAACATCAAATTGGGTGTCCACGAAGACACTCAAAACAGAGTTGCTTTGGCTAAGTTGC

TACGCTACAACTCCACCAAGTCTGTCGACGAATTGACTTCCTTGACCGACTACGTCACCAGAATGCCTGA

ACACCAAAAGAACATCTACTACATCACTGGTGAATCTCTAAAGGCTGTTGAAAAGTCTCCATTCTTGGAC

GCTTTGAAGGCTAAGAACTTTGAAGTTTTGTTTTTGACTGATCCAATTGATGAATACGCCTTCACTCAAT

TGAAGGAATTTGAAGGTAAGACTTTGGTTGATATTACCAAGGACTTTGAATTGGAAGAAACTGATGAAGA

GAAGGCTGAAAGGGAGAAGGAAATCAAAGAATTCGAACCATTGACCAAGGCCTTGAAGGACATCTTGGGT

GACCAAGTTGAAAAGGTTGTTGTTTCTTACAAATTGTTGGACGCTCCAGCCGCTATTAGAACCGGTCAAT

TTGGTTGGTCTGCTAACATGGAAAGAATCATGAAGGCTCAAGCTTTGAGAGATTCTTCCATGTCCTCCTA

CATGTCCTCTAAGAAGACCTTCGAAATTTCTCCAAAATCCCCAATTATTAAGGAATTGAAGAAGAGAGTT

GACGAAGGTGGTGCTCAAGATAAGACTGTCAAGGATTTGACCAACTTGCTATTCGAGACTGCTTTGTTGA

CTTCTGGTTTCAGTTTGGAAGAGCCAACTTCCTTTGCTTCAAGAATCAATAGGTTAATCTCTCTAGGCTT

GAATATTGATGACGAAGAAGAAGTAGAAGCTGCTCCAGAAGCTTCCACCGAAGCTCCAGTTGAAGAAGTC

CCAGCTGACACCGAAATGGAAGAAGTTGATTAA

>contig_448_*Saccharomyces*_*mikatae*_IFO_1815_710aa

MAGETFEFQAEITQLMSLIINTVYSNKEIFLRELISNASDALDKIRYQALSDPKQLETEPDLFIRITPRP

EEKVLEIRDSGIGMTKAELINNLGTIAKSGTKAFMEALSAGADVSMIGQFGVGFYSLFLVADRVQVISKN

NDDEQYIWESNAGGSFTVTLDEVNERIGRGTVLRLFLKDDQLEYLEEKRIKEVIKRHSEFVAYPIQLLVT

KEVEKEVPIPEEEKKDEEKKDEEKKDEDDKKPKLEEVDEEEEEKKPKTKKVKEEVQELEELNKTKPLWTR

NPSEITQEEYNAFYKSISNDWEDPLYVKHFSVEGQLEFRAILFIPKRAPFDLFESKKKKNNIKLYVRRVF

ITDEAEDLIPEWLSFVKGVVDSEDLPLNLSREMLQQNKIMKVIRKNIVKKLIESFNEIAEDSEQFDKFYS

AFAKNIKLGVHEDTQNRVALAKLLRYNSTKSVDELTSLTDYVTRMPEHQKNIYYITGESLKAVEKSPFLD

ALKAKNFEVLFLTDPIDEYAFTQLKEFEGKTLVDITKDFELEETDEEKAEREKEIKEFEPLTKALKDILG

DQVEKVVVSYKLLDAPAAIRTGQFGWSANMERIMKAQALRDSSMSSYMSSKKTFEISPKSPIIKELKKRV

DEGGAQDKTVKDLTNLLFETALLTSGFSLEEPTSFASRINRLISLGLNIDDEEEVEAAPEASTEAPVEEV

PADTEMEEVD

>contig_161_*Saccharomyces_paradoxus*_NRRL_Y-17217_cds_2133bp

ATGGCTGGTGAAACTTTTGAATTTCAAGCTGAAATCACTCAGTTGATGAGTTTGATCATCAACACTGTCT

ATTCTAACAAGGAAATTTTCTTGAGAGAACTGATCTCCAACGCTTCCGATGCTTTAGACAAGATTAGATA

CCAAGCTTTGTCTGACCCAAAGCAATTGGAAACCGAACCAGATTTGTTCATTAGAATCACCCCAAAACCA

GAAGAAAAAGTTTTGGAAATTAGAGATTCCGGTATTGGTATGACCAAGGCGGAATTGATCAATAACTTGG

GTACCATTGCCAAGTCTGGTACTAAAGCTTTCATGGAAGCTCTTTCCGCTGGTGCCGATGTATCCATGAT

CGGTCAATTCGGTGTTGGTTTCTATTCTTTATTCTTGGTCGCTGACAGAGTTCAGGTTATTTCAAAGAAC

AATGACGACGAACAATATATTTGGGAATCCAACGCTGGCGGTTCTTTCACTGTTACTTTAGACGAAGTTA

ATGAAAGAATTGGCAGAGGTACCGTCTTGAGACTATTTTTGAAGGATGACCAGTTGGAGTACTTGGAAGA

AAAGAGAATTAAGGAAGTCATCAAGAGACACTCTGAATTCGTCGCTTACCCAATCCAACTTCTAGTCACC

AAGGAAGTTGAAAAGGAAGTTCCAATTCCAGAAGAAGAAAAGAAGGACGAAGAAAAGAAAGACGAGGAAA

AGAAGGATGAAGATGACAAGAAACCAAAATTAGAAGAAGTCGATGAAGAAGAAGAAGAAAAGAAGCCAAA

AACCAAAAAAGTTAAGGAAGAAGTTCAAGAATTAGAAGAATTAAACAAGACTAAACCATTATGGACCAGA

AACCCTTCTGACATCACTCAAGAAGAATACAATGCTTTCTACAAGTCTATTTCTAACGACTGGGAAGACC

CATTATATGTCAAGCATTTCTCTGTCGAGGGTCAATTGGAATTTAGAGCTATCTTATTCATTCCAAAGAG

AGCACCATTCGACTTGTTTGAAAGTAAGAAGAAGAAGAATAACATTAAGTTGTACGTTCGTCGTGTGTTC

ATCACTGATGAAGCTGAAGACTTGATTCCAGAATGGTTGTCTTTCGTCAAGGGTGTTGTTGACTCTGAAG

ATTTACCATTGAACTTGTCCAGAGAAATGTTACAACAGAATAAGATCATGAAGGTTATTAGAAAGAACAT

TGTCAAGAAGTTGATCGAATCCTTTAACGAAATTGCTGAGGACTCCGAGCAATTTGACAAATTTTACTCC

GCCTTTGCCAAGAACATTAAACTGGGTGTACATGAAGACACTCAAAACAGAGTTGCTTTGGCTAAGTTGC

TACGTTACAACTCTACCAAGTCCGTCGATGAATTGACTTCCTTGAGCGATTACGTCACCAGAATGCCAGA

ACACCAAAAGAACATTTACTACATTACCGGTGAATCTCTAAAGGCTGTCGAAAAGTCGCCATTCTTGGAC

GCTTTGAAGGCTAAGAACTTTGAAGTTTTGTTCTTGACCGATCCAATTGATGAATACGCTTTCACTCAAT

TGAAGGAATTCGAAGGTAAGACTTTGGTTGACATCACCAAAGACTTTGAATTGGAAGAAACAGATGAAGA

GAAAGCCGAAAGAGAGAAGGAAATTAAAGAATACGAACCATTGACCAAGGCCTTGAAGGACATCTTGGGT

GACCAAGTGGAAAAGGTTGTCGTTTCGTACAAATTGTTGGACGCTCCAGCTGCCATCAGAACTGGTCAAT

TCGGTTGGTCCGCTAACATGGAAAGAATCATGAAGGCTCAAGCCTTGAGGGATTCCTCCATGTCCTCTTA

CATGTCTTCCAAGAAGACTTTCGAAATTTCTCCAAAATCTCCAATTATTAAGGAATTGAAAAAGAGAGTT

GATGAAGGTGGTGCTCAAGATAAGACTGTCAAGGATTTGACTAACTTATTATTCGAGACCGCTTTGTTGA

CTTCTGGTTTCAGTTTGGAAGAGCCAACTTCCTTTGCTTCAAGAATAAATAGATTGATCTCCTTAGGTTT

GAATATTGATGAGGAAGAAGAAACAGAAGCCGCTCCAGAAGCTTCTACCGAAGCTCCAGTTGAAGAGGTT

CCAGCTGACACCGAAATGGAAGAAGTTGATTAA

>contig_161_*Saccharomyces_paradoxus*_NRRL_Y-17217_710aa

MAGETFEFQAEITQLMSLIINTVYSNKEIFLRELISNASDALDKIRYQALSDPKQLETEPDLFIRITPKP

EEKVLEIRDSGIGMTKAELINNLGTIAKSGTKAFMEALSAGADVSMIGQFGVGFYSLFLVADRVQVISKN

NDDEQYIWESNAGGSFTVTLDEVNERIGRGTVLRLFLKDDQLEYLEEKRIKEVIKRHSEFVAYPIQLLVT

KEVEKEVPIPEEEKKDEEKKDEEKKDEDDKKPKLEEVDEEEEEKKPKTKKVKEEVQELEELNKTKPLWTR

NPSDITQEEYNAFYKSISNDWEDPLYVKHFSVEGQLEFRAILFIPKRAPFDLFESKKKKNNIKLYVRRVF

ITDEAEDLIPEWLSFVKGVVDSEDLPLNLSREMLQQNKIMKVIRKNIVKKLIESFNEIAEDSEQFDKFYS

AFAKNIKLGVHEDTQNRVALAKLLRYNSTKSVDELTSLSDYVTRMPEHQKNIYYITGESLKAVEKSPFLD

ALKAKNFEVLFLTDPIDEYAFTQLKEFEGKTLVDITKDFELEETDEEKAEREKEIKEYEPLTKALKDILG

DQVEKVVVSYKLLDAPAAIRTGQFGWSANMERIMKAQALRDSSMSSYMSSKKTFEISPKSPIIKELKKRV

DEGGAQDKTVKDLTNLLFETALLTSGFSLEEPTSFASRINRLISLGLNIDEEEETEAAPEASTEAPVEEV

PADTEMEEVD

>contig_381_*Saccharomyces_paradoxus*_NRRL_Y-17217_cds_2133bp

ATGGCTGGGGAAACTTTTGAATTTCAAGCTGAAATTACTCAGTTGATGAGTTTGATCATCAACACTGTCT

ACTCTAACAAGGAAATTTTTCTGAGAGAACTGATATCCAATGCTTCGGATGCGTTAGATAAAATCAGATA

TAAGTCTTTGTCTGACCCAAAGCAGTTGGAAACCGAACCGGATTTATTCATTAGAATCACTCCAAAACCA

GAGCAAAAAGTTTTGGAAATCAGAGATTCTGGTATTGGTATGACCAAGGCGGAATTGATCAATAACTTGG

GTACTATTGCCAAGTCTGGTACCAAAGCTTTCATGGAAGCTCTTTCTGCTGGTGCGGATGTGTCCATGAT

CGGTCAATTTGGTGTTGGTTTTTACTCTTTATTCTTGGTTGCAGACAGGGTCCAGGTTATTTCAAAGAGC

AATGACGACGAACAATACATCTGGGAATCCAACGCTGGCGGTTCTTTCACTGTTACTTTAGATGAGGTTA

ATGAAAGAATTGGCAGAGGTACCGTTTTAAGACTATTTTTGAAGGACGACCAGTTGGAATACTTGGAGGA

AAAGAGAATAAAGGAGGTCATTAAGAGACACTCTGAATTCGTGGCTTATCCAATCCAATTAGTTGTCACT

AAGGAAGTTGAAAAGGAAGTTCCAATTCCAGAAGAAGAAAAGAAAGACGAAGAAAAGAAAGAAGAGGAAA

AGAAGGATGAAGATGACAAGAAACCAAAATTGGAAGAAGTCGATGAAGATGAAGAAGAAAAGAAGCCAAA

AACAAAGAAAGTTAAGGAGGAAGTTCAAGAAATAGAAGAATTGAACAAGACTAAACCATTATGGACCAGA

AATCCATCTGACATCACTCAAGAAGAATACAATGCTTTCTACAAGTCTATTTCTAACGACTGGGAAGACC

CATTATATGTCAAGCATTTCTCTGTCGAGGGTCAATTGGAATTTAGAGCTATCTTATTCATTCCAAAGAG

AGCACCATTCGACTTGTTTGAAAGTAAGAAGAAGAAGAATAACATTAAGTTGTACGTTCGTCGTGTGTTC

ATCACTGATGAAGCTGAAGACTTGATTCCAGAATGGTTGTCTTTCGTCAAGGGTGTTGTTGACTCGGAAG

ATTTACCATTGAACTTGTCCAGAGAAATGTTACAACAGAATAAGATCATGAAGGTTATTAGAAAGAACAT

TGTCAAGAAGCTGATTGAAGCCTTCAACGAAATTGCTGAAGACTCTGAACAATTCGAAAAGTTCTACTCT

GCTTTCTCCAAAAATATCAAGTTGGGTGTGCATGAAGATACTCAAAACAGGGTTGCTTTGGCTAAGTTGC

TACGTTACAACTCTACCAAGTCCGTCGATGAGTTGACTTCCTTGACCGATTACGTCACCAGAATGCCAGA

ACATCAAAAGAACATTTACTACATTACCGGTGAATCTCTAAAGGCTGTCGAAAAATCGCCATTCTTGGAC

GCTTTGAAAGCTAAGAACTTTGAAGTTTTGTTCTTGACCGATCCAATTGATGAATATGCTTTCACCCAAC

TGAAGGAATTCGAAGGTAAGACTTTGGTTGACATCACCAAAGACTTTGAATTGGAAGAAACTGATGAAGA

GAAAGCCGAAAGAGAGAAAGAAATTAAAGAATACGAACCATTGACCAAGGCCTTGAAGGACATCTTGGGT

GACCAAGTGGAGAAAGTTGTCGTTTCTTACAAATTGTTGGATGCTCCAGCTGCCATCAGAACTGGCCAAT

TCGGTTGGTCCGCTAACATGGAAAGAATCATGAAGGCACAAGCCTTGAGGGACTCTTCCATGTCCTCCTA

CATGTCTTCCAAGAAGACTTTCGAAATTTCTCCAAAATCTCCAATTATCAAGGAATTGAAAAAGAGAGTT

GATGAAGGTGGTGCTCAAGATAAGACTGTCAAGGATTTGACTAACTTACTATATGAAACAGCATTGTTGA

CTTCCGGCTTCAGTTTGGACGAGCCAACCTCTTTCGCATCAAGAATTAACAGATTGATTTCCTTAGGTTT

GAACATTGATGAAGACGAAGAAACAGAGGCTGCTCCAGAGGCTTCCGCCGAAGCTCCTGTTGAAGAGGTT

CCAGCTGACACTGAAATGGAAGAAGTCGATTAG

>contig_381_*Saccharomyces_paradoxus*_NRRL_Y-17217_710aa

MAGETFEFQAEITQLMSLIINTVYSNKEIFLRELISNASDALDKIRYKSLSDPKQLETEPDLFIRITPKP

EQKVLEIRDSGIGMTKAELINNLGTIAKSGTKAFMEALSAGADVSMIGQFGVGFYSLFLVADRVQVISKS

NDDEQYIWESNAGGSFTVTLDEVNERIGRGTVLRLFLKDDQLEYLEEKRIKEVIKRHSEFVAYPIQLVVT

KEVEKEVPIPEEEKKDEEKKEEEKKDEDDKKPKLEEVDEDEEEKKPKTKKVKEEVQEIEELNKTKPLWTR

NPSDITQEEYNAFYKSISNDWEDPLYVKHFSVEGQLEFRAILFIPKRAPFDLFESKKKKNNIKLYVRRVF

ITDEAEDLIPEWLSFVKGVVDSEDLPLNLSREMLQQNKIMKVIRKNIVKKLIEAFNEIAEDSEQFEKFYS

AFSKNIKLGVHEDTQNRVALAKLLRYNSTKSVDELTSLTDYVTRMPEHQKNIYYITGESLKAVEKSPFLD

ALKAKNFEVLFLTDPIDEYAFTQLKEFEGKTLVDITKDFELEETDEEKAEREKEIKEYEPLTKALKDILG

DQVEKVVVSYKLLDAPAAIRTGQFGWSANMERIMKAQALRDSSMSSYMSSKKTFEISPKSPIIKELKKRV

DEGGAQDKTVKDLTNLLYETALLTSGFSLDEPTSFASRINRLISLGLNIDEDEETEAAPEASAEAPVEEV

PADTEMEEVD

>contig0.9_*Saccharomyces_pastorianus*_Weihenstephan_cds_2139bp

ATGGCTGGTGAAACTTTTGAATTTCAAGCTGAAATTACTCAGTTGATGAGTTTAATCATCAACACTGTTT

ATTCTAACAAGGAAATTTTCTTGAGAGAACTGATCTCCAATGCCTCTGATGCTTTGGACAAGATCAGATA

CCAAGCTTTGTCCAACCCAAAGGAATTGGAAACTGAACCTGAATTATTCATTAGAATCACTCCAAGACCA

GAAGAAAAAGTCTTGGAAATCAGGGATTCCGGTATTGGTATGACCAAGGCGGAATTGATCAACAACTTGG

GTACCATCGCTAAGTCTGGTACCAAGGCCTTTATGGAAGCTCTTTCTGCTGGTGCCGATGTCTCCATGAT

CGGTCAATTCGGTGTTGGTTTCTACTCTTTATTCTTGGTCGCTGATAGAGTCCAAGTTATCTCTAAGAAC

AACGAAGATGAACAATACATTTGGGAATCCAACGCAGGTGGTTCTTTCTCTGTTACTTTAGACGAAGTCA

ACGAAAAGATCGGTAGAGGTACTGTCTTGAGATTATTCTTGAAGGATGACCAATTGGAATACTTGGAAGA

AAAGAAGATTAAGGAAGTCATCAAGAGACACTCCGAATTCGTTGCTTACCCAATCCAATTGCTAGTCACC

AAGGAAGTTGAAAAGGAAGTTCCAATCCCAGAAGAAGAAGAAGAAAAGAAAGACGAAGAAAAGAAGGATG

AAGAAAAAAAGGATGAAGATGACAAGAAACCAAAATTGGAAGAAGTCGATGAAGAAGAAGAGGAAAAGAA

GCCAAAGACCAAGAAAGTCAAGGAAGAAGTTCAAGAATTAGAAGAACTAAACAAGACTAAGCCTCTATGG

ACCAGAAACCCATCCGAAATCACCGAAGAAGAATACAATGCCTTCTACAAATCTATCTCCAACGACTGGG

AAGACCCACTATATGTTAAGCATTTCTCCGTTGAAGGTCAATTAGAATTCAGAGCCATCTTGTATATTCC

AAAGAGAGCACCATTTGACTTATTCGAAAGTAAGAAGAAGAAGAACAACATCAAGTTGTACGTTCGTCGT

GTTTTCATCACCGACGAAGCTGAAGACTTGATTCCAGAATGGATGTCCTTCGTCAAGGGTGTTGTTGACT

CTGAAGATTTACCATTGAACTTGTCCAGAGAAATGTTACAACAAAACAAGATCATGAAGGTTATCAGAAA

GAACATTGTCAAGAAGGTAATTGAAGCCTTCAACGAAATTGCTGAAGACTTTGAACAATTCGAAAAGTTC

TACTCTGCCTTCGCTAAAAACATCAAATTGGGTGTCCACGAAGACACTCAAAACAGAGCTGCCTTGGCTA

AGTTGCTACGTTACAACTCCACCAAGTCCGTCGACGAATTAACTTCTTTGACCGATTACATCACCAGAAT

GCCAGAACACCAAAAGAACGTTTACTATATCACAGGTGAGTCTCTAAAGGCCGTTGAAAAATCCCCATTC

TTGGACGCTTTGAAGGCTAAGAACTTTGAAGTTTTGTTCTTGACTGATCCAATCGATGAATACGCTTTCA

CCCAATTGAAGGAATTCGAAGGTAAGACTTTGGTCGATATCACCAAGGATTTCGAACTGGAAGAAACTGA

CGAAGAAAAAGCTGAAAGAGAGAAGGAAGTCAAAGAATACGAACCATTGACCAAGGCCTTGAAAGAAATC

TTGGGCGAACAAGTCGAAAAGGTTGTTGTCTCTTATAAACTAGTGGACGCCCCAGCTGCCATTAGAACCG

GCCAATTCGGTTGGTCTGCTAACATGGAAAGAATTATGAAGGCTCAAGCCTTGAGAGACTCTTCCATGTC

CTCTTACATGTCCTCCAAGAAGACTTTCGAAATCTCTCCAAGATCTCCAATCATTAAGGAATTGAAAAAG

AGAGTCGACGAAGGTGGTGCTCAAGATAAGACTGTCAAGGATTTGACCAACTTATTATTCGAAACCGCTT

TATTGACTTCTGGTTTCAGTTTGGAAGAACCAACTTCTTTCGCTTCAAGAATCAACAGATTGATCTCTTT

GGGTTTGAACATCGATGAAGAAGAAGAGACTGAAGCTGCTACTGAGACTGCTACTGATGCTCCAGTCGAA

GAAGTTCCAGCTGACACCGAAATGGAAGAAGTTGATTAA

>contig0.9_*Saccharomyces_pastorianus*_Weihenstephan_712aa

MAGETFEFQAEITQLMSLIINTVYSNKEIFLRELISNASDALDKIRYQALSNPKELETEPELFIRITPRP

EEKVLEIRDSGIGMTKAELINNLGTIAKSGTKAFMEALSAGADVSMIGQFGVGFYSLFLVADRVQVISKN

NEDEQYIWESNAGGSFSVTLDEVNEKIGRGTVLRLFLKDDQLEYLEEKKIKEVIKRHSEFVAYPIQLLVT

KEVEKEVPIPEEEEEKKDEEKKDEEKKDEDDKKPKLEEVDEEEEEKKPKTKKVKEEVQELEELNKTKPLW

TRNPSEITEEEYNAFYKSISNDWEDPLYVKHFSVEGQLEFRAILYIPKRAPFDLFESKKKKNNIKLYVRR

VFITDEAEDLIPEWMSFVKGVVDSEDLPLNLSREMLQQNKIMKVIRKNIVKKVIEAFNEIAEDFEQFEKF

YSAFAKNIKLGVHEDTQNRAALAKLLRYNSTKSVDELTSLTDYITRMPEHQKNVYYITGESLKAVEKSPF

LDALKAKNFEVLFLTDPIDEYAFTQLKEFEGKTLVDITKDFELEETDEEKAEREKEVKEYEPLTKALKEI

LGEQVEKVVVSYKLVDAPAAIRTGQFGWSANMERIMKAQALRDSSMSSYMSSKKTFEISPRSPIIKELKK

RVDEGGAQDKTVKDLTNLLFETALLTSGFSLEEPTSFASRINRLISLGLNIDEEEETEAATETATDAPVE

EVPADTEMEEVD

>contig4.9_*Saccharomyces_pastorianus*_Weihenstephan_cds_2124bp

ATGGCTGGTGAAACCTTTGAATTTCAAGCAGAAATCACTCAGTTGATGAGTTTGATCATCAACACTGTCT

ATTCTAACAAGGAGATTTTCTTGAGAGAACTGATATCTAACGCCTCCGATGCGTTGGACAAGATCAGATA

TAAGGCTTTGTCCGACCCAAAGGAATTAGAAACTGAACCTGAATTGTTCATTAGAATCACTCCAAGACCG

GAAGAAAAAGTCTTGGAAATCAGAGATTCTGGTATTGGTATGACCAAGGCGGAATTGATCAACAACTTGG

GTACCATCGCTAAGTCTGGTACCAAGGCCTTTATGGAGGCTCTTTCTGCTGGTGCCGATGTCTCCATGAT

TGGCCAATTCGGTGTTGGTTTCTACTCTTTATTCTTGGTCGCTGATAGGGTCCAAGTTATCTCTAAGAAC

AACGACGATGAACAATACATCTGGGAGTCCAACGCAGGCGGTTCTTTCACTGTTACTCTAGACGAAGTTA

ACGAAAAGATCGGTAGAGGTACTGTCTTGAGGTTATTCATGAAGGACGACCAATTGGAATACCTGGAGGA

AAAGAAGATTAAGGAAGTCATCAAGAGACATTCTGAATTTGTTGCTTACCCAATCCAATTGCTAGTCACC

AAGGAAGTTGAAAAGGAAGTTCCAATCCCAGAAGAAGAAGAAGAAAAGAAAGACGAAGAAAAGAAGGATG

AAGATGACAAGAAACCAAAATTGGAAGAAGTCGATGAAGAAGAAGAGGAAAAGAAGCCAAAGACCAAGAA

AGTCAAGGAAGAAGTTCAAGAATTAGAAGAACTAAACAAGACTAAGCCTCTATGGACCAGAAACCCATCC

GAAATCACCGAAGAAGAATACAATGCCTTCTACAAATCCATCTCCAACGATTGGGAAGACCCACTATATG

TTAAGCACTTCTCCGTCGAAGGTCAGTTAGAATTCAGAGCCATCTTGTACATTCCAAAGAGAGCTCCATT

TGACTTATTCGAAAGTAAGAAGAAGAAGAACAACATCAAGTTGTATGTTCGTCGTGTTTTCATCACCGAC

GAAGCTGAAGACTTGATCCCAGAATGGATGTCCTTTGTCAAGGGTGTTGTTGACTCTGAAGACTTACCAT

TGAACCTGTCCAGAGAAATGCTACAACAAAACAAGATCATGAAGGTTATCAGAAAGAACATCGTCAAGAA

GGTAATTGAAGCTTTCAACGAAATTGCTGAAGACTCTGAACAATTCGAAAAGTTCTACTCTGCCTTCGCT

AAGAACATCAAATTGGGTGTCCACGAAGACACTCAAAACAGAGCTGCCTTGGCTAAGTTACTACGTTACA

ACTCCACCAAGTCCGTCGACGAATTAACTTCTTTGACCGATTACATCACCAGAATGCCAGAACACCAAAA

GAACATTTACTACATCACAGGTGAATCTCTAAAGGCCGTTGAAAAATCCCCATTCTTGGACGCTTTGAAG

GCTAAGAACTTTGAAGTTTTGTTCTTGACTGATCCAATCGATGAATACGCTTTCACCCAATTGAAGGAAT

TCGAAGGTAAGACTTTGGTCGATATCACCAAGGATTTCGAACTGGAAGAAACTGACGAAGAAAAAGCTGA

AAGAGAGAAGGAGATCAAAGAATATGAACCATTGACCAAGGCCTTGAAAGAAATTTTGGGTGACCAAGTG

GAGAAAGTTGTTGTTTCTTACAAATTGTTGGATGCCCCAGCTGCTATCAGAACTGGTCAATTTGGTTGGT

CTGCTAACATGGAAAGAATCATGAAGGCTCAAGCCTTGAGAGACTCTTCCATGTCCTCCTACATGTCTTC

CAAGAAGACTTTCGAAATTTCTCCAAAATCTCCAATTATCAAGGAATTGAAAAAGAGAGTTGACGAAGGT

GGTGCTCAAGACAAGACTGTCAAGGACTTGACTAAGTTATTATATGAAACTGCTTTGTTGACTTCCGGCT

TCAGTTTGGACGAACCAACTTCCTTTGCATCAAGAATTAACAGATTGATCTCTTTGGGTTTGAACATTGA

TGAGGATGAAGAAACAGAGACTGCTCCAGAAGCATCCACCGCAGCTCCGGTTGAAGAGGTTCCAGCTGAC

ACCGAAATGGAAGAGGTAGATTAG

>contig4.9_*Saccharomyces_pastorianus*_Weihenstephan_707aa

MAGETFEFQAEITQLMSLIINTVYSNKEIFLRELISNASDALDKIRYKALSDPKELETEPELFIRITPRP

EEKVLEIRDSGIGMTKAELINNLGTIAKSGTKAFMEALSAGADVSMIGQFGVGFYSLFLVADRVQVISKN

NDDEQYIWESNAGGSFTVTLDEVNEKIGRGTVLRLFMKDDQLEYLEEKKIKEVIKRHSEFVAYPIQLLVT

KEVEKEVPIPEEEEEKKDEEKKDEDDKKPKLEEVDEEEEEKKPKTKKVKEEVQELEELNKTKPLWTRNPS

EITEEEYNAFYKSISNDWEDPLYVKHFSVEGQLEFRAILYIPKRAPFDLFESKKKKNNIKLYVRRVFITD

EAEDLIPEWMSFVKGVVDSEDLPLNLSREMLQQNKIMKVIRKNIVKKVIEAFNEIAEDSEQFEKFYSAFA

KNIKLGVHEDTQNRAALAKLLRYNSTKSVDELTSLTDYITRMPEHQKNIYYITGESLKAVEKSPFLDALK

AKNFEVLFLTDPIDEYAFTQLKEFEGKTLVDITKDFELEETDEEKAEREKEIKEYEPLTKALKEILGDQV

EKVVVSYKLLDAPAAIRTGQFGWSANMERIMKAQALRDSSMSSYMSSKKTFEISPKSPIIKELKKRVDEG

GAQDKTVKDLTKLLYETALLTSGFSLDEPTSFASRINRLISLGLNIDEDEETETAPEASTAAPVEEVPAD

TEMEEVD

**ARTHROPODS**

>cont10747_*Anopheles_darlingi*_cds_342bp

ATGCCGGAAGCACCAGAAACCGAGACCTTTGCGTTCCAGGCTGAGATCGCTCAGCTGATGTCCCTGATCA

TCAACACATTCTACTCGAACAAGGAAATCTTCCTGCGTGAGTTGATCTCGAACTCATCGGATGCGCTGGA

TAAGATCCGCTATGAGTCGCTGACTGACCCCACGAAACTGGACTCGGGCAAGGAGCTGTTCATCAAGATC

ATCCCGAACAAGGAGGCGGGTACGCTGACGCTCATCGATACCGGTATCGGTATGACGAAGGCGGATCTTG

TGAACAACCTCGGTACGATTGCCAAGTCCGGCACCAAGGCGTTCATGGAGGCTCTGCAGGCC

>cont10747_*Anopheles_darlingi*_114aa

MPEAPETETFAFQAEIAQLMSLIINTFYSNKEIFLRELISNSSDALDKIRYESLTDPTKLDSGKELFIKI

IPNKEAGTLTLIDTGIGMTKADLVNNLGTIAKSGTKAFMEALQA

>cont10259_*Anopheles_darlingi*_cds_2166bp

ATGCCGGAAGCACCAGAAACCGAGACCTTTGCGTTCCAGGCTGAGATCGCTCAGCTGATGTCCCTGATCA

TCAACACATTCTACTCGAACAAGGAAATCTTCCTGCGTGAGTTGATCTCGAACTCATCGGATGCGCTGGA

TAAGATCCGCTATGAGTCGCTGACTGACCCCACGAAACTGGACTCGGGCAAGGAGCTGTTCATCAAGATC

ATCCCGAACAAGGAGGCGGGTACGCTGACGCTCATCGATACCGGTATCGGTATGACGAAGGCGGATCTGG

TGAACAACCTCGGTACGATTGCCAAGTCCGGCACCAAGGCGTTCATGGAGGCTCTGCAGGCCGGTGCCGA

CATCAGCATGATCGGTCAGTTCGGTGTGGGTTTCTACTCGGCCTACCTGGTGGCCGATAAGGTGGTGGTC

ACGTCCAAGAACAACGATGACGAGCAGTACGTATGGGAGTCGTCGGCCGGAGGTTCGTTCACCGTGCGCT

CCGATAGCGGAGAGCCGCTTGGCCGCGGTACCAAGATCGTGCTGCACATCAAGGAGGACCAGCTGGAGTA

TCTGGAGGAAAGCAAGATCAAGCAGATCGTCAACAAGCACTCGCAGTTCATCGGTTATCCGATCAAGCTG

CTGGTGGAGAAGGAGCGCGAGAAGGAGGTGAGCGACGATGAGGCCGAGGATGATAAGAAGGAAGAGAAGA

AGGAGGAGGACAAGAAGGACGATGAGCCAAAGCTGGAGGACGCTGAGGATGACGAAGATAAGAAGGACAA

GAAGAAGAAGACGGTGAAGGTGAAGTACACCGAGGACGAGGAGCTGAACAAGACGAAGCCGATCTGGACG

CGCAACGCCGACGATATCTCTCAGGAGGAGTACGGCGAGTTCTACAAATCGCTGACCAACGACTGGGAGG

ACCATCTGGCGGTGAAGCACTTCTCGGTCGAGGGCCAGTTGGACTTCCGTGCGCTGCTGTTTGTCCCGCG

TCGTATGCCGTTCGATATGTTCGAAAACAAGAAGAAGAAGAACAACATCAAGCTGTACGTGCGCCGCGTG

TTCATCATGGACAACTGCGAAGAGCTGATCCCTGATTATCTGAACTTCATCAAGGGCGTCGTCGATTCCG

AGGATCTGCCTCTAAACATCTCGCGTGAAATGCTGCAGCAGAACAAGATCCTGAAGGTGATCCGCAAGAA

CTTGGTGAAGAAGTGCATGGAGCTGTTCGAGGAACTGGCCGAGGACAAGGAGACGTACAAGAAGTTCTAC

GATCAGTTCAGCAAGAACCTGAAGCTGGGCGTGCACGAGGACAGCCAGAACCGTCAGAAGCTGGCCGATC

TGCTGCGCTTCAACACGTCGGCATCGGGCGATGAGTACTGCTCGCTGAACGACTACGTTGGCCGTATGAA

GGAGAACCAGACGCAGATCTACTTCATCACGGGCGAGAGCATCGAGCAGGTGAAGAACTCGGCATTCGTG

GAGCGCGTGAAGAAGCGCGGCTTCGAGGTGATCTACATGACGGAGGCCATCGATGAGTACGTGATCCAGC

AGCTGAAGGAGTACAAGGGCAAGCAGCTGGTATGCGTGACGAAGGAAGGTCTGGAGCTGCCAGAGGATGA

GGCCGAGAAGAAGAAGCGCGAGGAGGACAAGGCCAAGTTCGAGAACCTGTGCAAGGTGATGAAGTCGGTC

CTGGAGAGCAAGGTGGAGAAGGTGGTGGTATCGAACCGTCTGGTGGACTCGCCCTGCTGTATCGTGACGT

CGCAGTACGGCTGGTCGGCCAACATGGAGCGTATCATGAAGGCACAGGCTCTGCGCGACTCGTCTGCCAT

GGGTTACATGGCTGGCAAGAAGCACCTGGAGATCAATCCCGATCATGCCATCATCGAAACGCTGCGACAG

CGCGCCGATGCCGATAAGAACGATAAGGCGGTGAAGGATCTGGTGATCCTGTTGTTCGAGACTGCCCTGC

TATCCTCTGGCTTCTCGCTCGATGAGCCTGGAACGCACGCCGCCCGCATCTACCGCATGGTGAAGCTTGG

TCTGGGCATCGACGATGACGAACCGATGACGACGGATGAAGTGAGTGGTGCTAGCGCACCGACGACGGCT

GCCGGAGATGCGCCACCGCTGGTGGACGATTCCGAGGATCTGTCACACATGGAGGAGGTCGATTAA

>cont10259_*Anopheles_darlingi*_721aa

MPEAPETETFAFQAEIAQLMSLIINTFYSNKEIFLRELISNSSDALDKIRYESLTDPTKLDSGKELFIKI

IPNKEAGTLTLIDTGIGMTKADLVNNLGTIAKSGTKAFMEALQAGADISMIGQFGVGFYSAYLVADKVVV

TSKNNDDEQYVWESSAGGSFTVRSDSGEPLGRGTKIVLHIKEDQLEYLEESKIKQIVNKHSQFIGYPIKL

LVEKEREKEVSDDEAEDDKKEEKKEEDKKDDEPKLEDAEDDEDKKDKKKKTVKVKYTEDEELNKTKPIWT

RNADDISQEEYGEFYKSLTNDWEDHLAVKHFSVEGQLDFRALLFVPRRMPFDMFENKKKKNNIKLYVRRV

FIMDNCEELIPDYLNFIKGVVDSEDLPLNISREMLQQNKILKVIRKNLVKKCMELFEELAEDKETYKKFY

DQFSKNLKLGVHEDSQNRQKLADLLRFNTSASGDEYCSLNDYVGRMKENQTQIYFITGESIEQVKNSAFV

ERVKKRGFEVIYMTEAIDEYVIQQLKEYKGKQLVCVTKEGLELPEDEAEKKKREEDKAKFENLCKVMKSV

LESKVEKVVVSNRLVDSPCCIVTSQYGWSANMERIMKAQALRDSSAMGYMAGKKHLEINPDHAIIETLRQ

RADADKNDKAVKDLVILLFETALLSSGFSLDEPGTHAARIYRMVKLGLGIDDDEPMTTDEVSGASAPTTA

AGDAPPLVDDSEDLSHMEEVD

>cont13917_*Anopheles_darlingi*_cds_354bp

TCTGCCATGGGCTACATGGCTGGCAAGAAGCACCTGGAGATCAATCCCGATCATGCCATCATCGAAACGC

TGCGACAGCGCGCCGATGCCGATAAGAACGATAAGGCGGTGAAGGATCTGGTGATCCTGTTGTTCGAGAC

TGCCCTGCTATCCTCTGGCTTCTCGCTCGATGAGCCTGGAACGCACGCCGCCCGCATCTACCGCATGGTG

AAGCTTGGTCTGGGCATCGACGATGACGAACCGATGACGACGGATGAAGTGAGTGGTGCTAGCGCACCGA

CGACGGCTGCCGGAGATGCGCCACCGCTGGTGGACGATTCCGAGGATCTGTCACACATGGAGGAGGTCGA

TTAA

>cont13917_*Anopheles_darlingi*_117aa

SAMGYMAGKKHLEINPDHAIIETLRQRADADKNDKAVKDLVILLFETALLSSGFSLDEPGTHAARIYRMV

KLGLGIDDDEPMTTDEVSGASAPTTAAGDAPPLVDDSEDLSHMEEVD

>2L-b_*Anopheles_gambiae*_cds_>1191bp

ATGCCGGAAGGACCAGAAGCCGAGACCTTCGCATTCCAGGCTGAGATTGCTCAGCTGATGTCCCTGATCA

TCAACACGTTCTACTCGAACAAGGAAATCTTCCTGCGTGAGTTGATCTCGAACTCGTCCGATGCGCTGGA

CAAGATCCGCTATGAATCGCTGACGGATCCCTCGAAGCTGGAGTCGGGCAAGGAGCTGTTCATCAAGATC

ATCCCGAACAAGGAGGCCGGCACACTGACGCTGATCGATACCGGTATCGGCATGACGAAGGCCGACCTGG

TGAACAACCTCGGTACGATTGCCAAGTCGGGCACGAAGGCGTTCATGGAGGCGCTGCAGGCCGGCGCAGA

CATCAGCATGATCGGCCAGTTCGGTGTCGGTTTCTACTCGGCGTACCTGGTCGCCGACAAGGTGGTGGTG

ACGTCGAAGAACAACGACGACGAGCAGTACGTGTGGGAGTCGTCGGCCGGCGGCTCGTTCACGGTGCGCC

CGGACAGCGGGGAGCCGCTGGGCCGCGGTACCAAGATCGTGCTGCACATCAAGGAGGACCAGCTGGAGTA

CCTGGAGGAGAGCAAGATCAAGCAGATCGTGAACAAGCACTCGCAGTTCATCGGCTACCCGATCAAGCTG

CTGGTGNNNNNNNNNNNNNNNNNNNNNNNNNNNNNNNNNNNNNNNNNNNNNNNNNNNNNNNNNNNNNNNN

NNNNNNNNNNNNNNNNNNNNNNNNNNNNNNNNNNNNGCCGAGAAGAAGAAGCGCGAGGAGGACAAGGCCA

AGTTCGAGAACCTGTGCAAGGTGATGAAGTCGGTGCTGGAGAGCAAGGTGGAGAAGGTGATGGTGTCGAA

CCGGCTGGTGGACTCGCCCTGCTGCATCGTGACGTCGCAGTACGGCTGGTCGGCCAACATGGAGCGCATC

ATGAAGGCGCAGGCGCTGCGTGACTCGTCGGCCATGGGCTACATGGCGGGCAAGAAGCATCTCGAGATCA

ACCCGGACCATGCCATCATCGAGACGCTGCGCCAGCGCGCCGAGGCGGACAAGAACGATAAGGCGGTGAA

GGATCTGGTGATTCTGCTGTTCGAGACGGCGCTGCTGTCCTCCGGCTTCTCGCTGGACGAGCCCGGAACC

CATGCGTCCCGCATCTACCGCATGATCAAGCTGGGTCTGGGCATCGACGAGGACGAGCCGATGACGACGG

ACGAGAGCAGCAGCGGTGCTGCGGCGGCGGCCCCAGCGTCGGGTGATGCTCCGCCGCTGGTGGACGACTC

GGAGGATCTGTCGCACATGGAGGAGGTCGATTAA

>2L-b_*Anopheles_gambiae*_397aa

MPEGPEAETFAFQAEIAQLMSLIINTFYSNKEIFLRELISNSSDALDKIRYESLTDPSKLESGKELFIKI

IPNKEAGTLTLIDTGIGMTKADLVNNLGTIAKSGTKAFMEALQAGADISMIGQFGVGFYSAYLVADKVVV

TSKNNDDEQYVWESSAGGSFTVRPDSGEPLGRGTKIVLHIKEDQLEYLEESKIKQIVNKHSQFIGYPIKL

LVAEKKKREEDKAKFENLCKVMKSVLESKVEKVMVSNRLVDSPCCIVTSQYGWSANMERIMKAQALRDSS

AMGYMAGKKHLEINPDHAIIETLRQRAEADKNDKAVKDLVILLFETALLSSGFSLDEPGTHASRIYRMIK

LGLGIDEDEPMTTDESSSGAAAAAPASGDAPPLVDDSEDLSHMEEVD

>2L-c

ATGCCGGAACCACAAGAGGGCGAGACCTTCGCATTCCAGGCTGAGATTGCTCAGCTGATGTCCCTGATCA

TCAACACGTTCTACTCGAACAAGGAAATCTTCCTGCGTGAGTTGATCTCGAACTCGTCCGATGCGCTGGA

CAAGATCCGCTATGAATCGCTGACGGATCCCTCGAAGCTGGAGTCGGGCAAGGAGCTGTTCATCAAGATC

ATCCCGAACAAGGAGGCCGGCACACTGACGCTGATCGATACCGGTATCGGCATGACGAAGGCCGACCTGG

TGAACAACCTCGGTACGATTGCCAAGTCGGGCACGAAGGCGTTCATGGAGGCGCTGCAGGCCGGCGCAGA

CATCAGCATGATCGGCCAGTTCGGTGTCGGTTTCTACTCGGCGTACCTGGTCGCCGACAAGGTGGTGGTG

ACGTCGAAGAACAACGACGACGAGCAGTACGTGTGGGAGTCGTCGGCCGGCGGCTCGTTCACGGTGCGCC

CGGACAGCGGGGAGCCGCTGGGCCGCGGTACCAAGATCGTGCTGCACATCAAAGGAGGACCAGCTGGAGT

ACCTGGAGGAGAGCAAGATCAAGCAGATCGTGAACAAGCACTCGCAGTNNNNNNNNNNNNNNNNNNNNNN

NNNNNNNNNNNNNNNNNNNNNNNNNNNNNNNNNNNNNNNNNNNNNNNNNNNNNNNNNNNNNNNNNNNNNN

NNNNNNNNCGCGAGGAGGACAAGGCCAAGTTCGAGAACCTGTGCAAGGTGATGAAGTCGGTGCTGGAGAG

CAAGGTGGAGAAGGTGATGGTGTCGAACCGGCTGGTGGACTCGCCCTGCTGCATCGTGACGTCGCAGTAC

GGCTGGTCGGCCAACATGGAGCGCATCATGAAGGCGCAGGCGCTGCGTGACTCGTCGGCCATGGGCTACA

TGGCGGGCAAGAAGCATCTCGAGATCAACCCGGACCATGCCATCATCGAGACGCTGCGCCAGCGCGCCGA

GGCGGACAAGAACGATAAGGCGGTGAAGGATCTGGTGATTCTGCTGTTCGAGACGGCGCTGCTGTCCTCC

GGCTTCTCGCTGGACGAGCCCGGAACCCATGCGTCCCGCATCTACCGCATGATCAAGCTGGGTCTGGGCA

TCGACGAGGACGAGCCGATGACGACGGAGGACAGCAGCAGCGGTGCTGCGGCGGCGGCCCCAGCGTCGGG

CGATGCTCCACCGCTGGTGGACGACTCGGAGGATCTGTCGCACATGGAGGAGGTCGATTAA

>2L-c_*Anopheles_gambiae_*383aa

MPEPQEGETFAFQAEIAQLMSLIINTFYSNKEIFLRELISNSSDALDKIRYESLTDPSKLESGKELFIKI

IPNKEAGTLTLIDTGIGMTKADLVNNLGTIAKSGTKAFMEALQAGADISMIGQFGVGFYSAYLVADKVVV

TSKNNDDEQYVWESSAGGSFTVRPDSGEPLGRGTKIVLHIKGGPAGVPGGEQDQADREQALAVREEDKAK

FENLCKVMKSVLESKVEKVMVSNRLVDSPCCIVTSQYGWSANMERIMKAQALRDSSAMGYMAGKKHLEIN

PDHAIIETLRQRAEADKNDKAVKDLVILLFETALLSSGFSLDEPGTHASRIYRMIKLGLGIDEDEPMTTE

DSSSGAAAAAPASGDAPPLVDDSEDLSHMEEVD

>scaffold01645_*Apis_florea*_cds_2265bp

ATGAGCAAACCTACTCAACGCGCGTCTTCATTCAATGAACTGCTAGGTGCTCGGTACTGTGTTTATAAAT

TTTTGAAGTGTATTTTAACGTTGGTTCAACTGAATATCAAGATGTCAACAGAAATAGAAACAAAAGCAGA

AGATGTGGAAACTTTTGCATTCCAGGCAGAGATTGCTCAACTTATGTCATTGATTATTAATACTTTTTAT

TCGAATAAAGAAATTTTTTTAAGAGAACTTATTTCAAACTCTAGTGACGCTCTTGATAAGATTCGTTATG

AATCTTTAACTGACCCTTCTAAGCTTGATAATGGCAAAGAATTATTTATTAAAATTATTCCAAACAAGAA

TGATGGTACTTTGACTATTATTGATACTGGTATTGGTATGACTAAGGCTGATTTGGTAAATAATCTTGGT

ACAATTGCAAAATCTGGTACCAAAGCTTTTATGGAAGCACTTCAAGCTGGTGCAGATATTTCCATGATTG

GACAATTTGGTGTAGGTTTTTATTCTGCTTATTTAATAGCAGACAAAGTCACTGTTATTTCAAAACACAA

TGATGATGAACAATATCTATGGGAATCTAGTGCTGGTGGATCATTTACTGTTAGACATGATAATGGAGAA

ACATTAGGAAGAGGAACAAAAATTGTTTTACATGTAAAAGAAGATCAAACTGAGTATTTAGAAGAGAGTA

AAATAAAAGAAATCGTAAAAAAACATTCTCAATTTATTGGTTATCCAATTAAATTAGTTGTGCAGAAGGA

ACGTGAAAAAGAACTGAGTGAGGATGAAGCTGAAGAAGAGGAAGAGAAAAAGGAAGATGATGGAAAACCA

AAAATTGAAGATGTAGATGAAAATGAAGGAACATCAGAGGAAGAAGGAAAAAAGAAGAAGAAGAAGACTA

TTAAAGAGAAATATACTGAAGATGAAGAATTAAACAAAACAAAACCTATCTGGACTAGGAACTCTGATGA

TATTACACAAGAAGAATATGGTGAATTTTACAAATCATTGACAAATGATTGGGAAGATCATTTGGCTGTT

AAGCATTTTTCTGTGGAAGGCCAATTAGAATTTAGAGCTTTACTTTTTATTCCAAAACGTATGCCATTTG

ATTTATTTGAAAATAAGAAAAGGAAAAATAATATTAAACTATATGTGAGAAGAGTATTCATTATGGACAA

CTGTGAACAGTTAATTCCAGAATATTTAAATTTCATAAAAGGAGTAGTTGATAGTGAAGATCTTCCTTTG

AACATTTCTCGTGAAATGCTTCAACAAAATAAAATTTTAAAGGTTATTCGTAAAAATCTTGTAAAAAAAT

GCATAGAATTGTTTGAAGAACTTGCAGAAGACAAAGACAACTATAAAAAATTCTATGAACAATTTAGCAA

AAACATTAAATTAGGTATTCATGAGGACAGTTCTAATCGTAGTAAATTGTCAGATCTTTTGAGATATAAT

ACTTCAGCTTCTGGAGATGAAGTCTGTTCTCTTAAAGATTATGTAGGCAGAATGAAAGAAAATCAAAAAC

ATATTTACTTCATTACTGGAGAGAACAAAGACCAAGTTGCAAACAGTTCATTTGTTGAACGTGTAAAAAA

ACGTGGCTTTGAAGTTGTTTATATGACAGAACCTATTGATGAATATGTAGTACAACAAATGAAAGAATTT

GATGGAAAACAATTAGTATCTGTAACCAAAGAAGGTTTAGAGTTTCCTGAAGATGAAGATGAAAAGAAAA

AAAGAGAAGAAGATAAAGCTAAATATGAAAATTTATGTAAAGTAATGAAAAATATTCTTGATAATAAAGT

GGAGAAAGTTGTAGTTTCAAATCGATTGGTTGATTCACCTTGCTGTATAGTTACTTCACAATATGGTTGG

ACTGCTAATATGGAAAGAATTATGAAAGCTCAAGCACTTAGAGACACATCTACCATGGGATATATGGCTG

CAAAAAAACATCTTGAAATAAATCCCGACCATACTATTATTGAAACTTTACATCAGAAAGCAGAAACAGA

TAAAAATGATAAAGCAGTAAAAGATTTAGTAATTTTATTGTTTGAAACGGCACTTTTATCTTCTGGTTTT

ACTTTAGATGAGCCACAAGTTCATGCTGCAAGAATTTACAGGATGATCAAATTGGGACTTGGTATTGATG

AAGAAGAATCTGTACCAGAAGAACAAACTACAGAAGAAATTCCTCCTCTGGAAGGAGATACAGAAGATAC

TTCACGAATGGAAGAAGTAGATTAA

>scaffold01645_*Apis_florea*_754aa

MSKPTQRASSFNELLGARYCVYKFLKCILTLVQLNIKMSTEIETKAEDVETFAFQAEIAQLMSLIINTFY

SNKEIFLRELISNSSDALDKIRYESLTDPSKLDNGKELFIKIIPNKNDGTLTIIDTGIGMTKADLVNNLG

TIAKSGTKAFMEALQAGADISMIGQFGVGFYSAYLIADKVTVISKHNDDEQYLWESSAGGSFTVRHDNGE

TLGRGTKIVLHVKEDQTEYLEESKIKEIVKKHSQFIGYPIKLVVQKEREKELSEDEAEEEEEKKEDDGKP

KIEDVDENEGTSEEEGKKKKKKTIKEKYTEDEELNKTKPIWTRNSDDITQEEYGEFYKSLTNDWEDHLAV

KHFSVEGQLEFRALLFIPKRMPFDLFENKKRKNNIKLYVRRVFIMDNCEQLIPEYLNFIKGVVDSEDLPL

NISREMLQQNKILKVIRKNLVKKCIELFEELAEDKDNYKKFYEQFSKNIKLGIHEDSSNRSKLSDLLRYN

TSASGDEVCSLKDYVGRMKENQKHIYFITGENKDQVANSSFVERVKKRGFEVVYMTEPIDEYVVQQMKEF

DGKQLVSVTKEGLEFPEDEDEKKKREEDKAKYENLCKVMKNILDNKVEKVVVSNRLVDSPCCIVTSQYGW

TANMERIMKAQALRDTSTMGYMAAKKHLEINPDHTIIETLHQKAETDKNDKAVKDLVILLFETALLSSGF

TLDEPQVHAARIYRMIKLGLGIDEEESVPEEQTTEEIPPLEGDTEDTSRMEEVD

>GL576082_*Apis_florea*_cds_2175bp

ATGCCGGAGGACGTCACCATGGCTAATGCCGGAGAGGTTGAAACTTTCGCTTTCCAAGCTGAAATTGCAC

AATTAATGAGTTTAATTATTAATACATTTTATTCCAATAAAGAAATTTTTATTCGAGAATTGATTTCAAA

TGCATCTGATGCATTAGACAAAATTCGCTATGAATCTCTTACGGATCCATCTAAATTGGATACATGCAAG

GAATTATTTATTAAAATTGTTCCTAATAAAAATGATCGTACTTTAACCATACTTGATTCTGGTATTGGTA

TGACAAAAGCTGATCTTGTGAACAATTTGGGTACCATTGCTAAATCTGGTACAAAAGCATTTATGGAAGC

TCTTCAAGCTGGTGCCGATATTTCTATGATTGGCCAGTTTGGTGTAGGTTTTTATTCTGCTTACCTTGTA

GCAGATAAAGTTGTTGTAATTTCAAAACACAATGATGACGAACAATATGTATGGGAGTCTTCTGCTGGTG

GTTCTTTCACTGTCCGCCCTGACAATGGAGAACCAATTGGAAGAGGTACCAAAATTATTTTACATATTAA

GGAAGATCAAACTGAATATTTGGAAGAATCTAAAATCAAAGAAATTGTGAAGAAGCATTCTCAATTCATT

GGTTATCCAATTAAACTTGTTGTTGAGAAGGAAAGAGATAAAGAATTAAGTGAAGATGAAGAAGAGGAAG

AAGAACCTGCAAAAGAAGAAGGTGAAGATACAGGTAAACCTAAGATTGAAGAAGTTGGAGAAGATGAAGA

TGAAGACAAACCTAAAGATGAAAAGAAGAAAAAGAAGAAAACTATTAAAGAGAAATACACTGAAGATGAG

GAATTAAATAAGACAAAACCAATCTGGACAAGAAATCCAGATGATATTTCTCAAGAAGAATATGGTGAAT

TTTACAAGAGTTTAACAAATGATTGGGAAGATCATTTGGCTGTCAAACATTTTTCAGTTGAAGGTCAATT

GGAATTCCGTGCATTGCTTTTCATTCCGCGACGTGCACCTTTTGATCTTTTTGAAAATAAAAAGAGAAAG

AATAACATCAAATTATATGTACGTAGAGTCTTCATTATGGATAATTGTGAAGATCTTATTCCAGAGTATC

TTAACTTTATTAAGGGTGTAGTTGATAGTGAAGATCTTCCATTGAATATTTCTCGTGAAATGTTACAACA

AAATAAAATTCTTAAAGTCATCAGAAAGAATCTTGTCAAGAAATGCTTAGAATTATTTGAAGAATTATCT

GAAGATAAGGAAAGTTATAAGAAATGCTATGAACAATTTAGCAAGAATATAAAATTAGGTATTCATGAAG

ACAGTCAAAATAGGAAAAAACTGTCAGAATTACTTAGATATCATACATCTGCATCTGGAGATGAAATGTG

TTCATTAAAGGATTATGTTGGAAGAATGAAAGAAAATCAGAAACACATTTACTATATCACTGGTGAAAGC

AGAGAACAAGTAGCCAATAGTTCATTTGTAGAAAGGGTTAAGAAGCGTGGTTTCGAAGTAGTATACATGA

CAGAACCCATTGATGAATATGTTGTACAACAATTGAAGGAGTTTGATGGCAAACAGTTGGTCTCTGTAAC

GAAAGAAGGTTTGGAACTTCCAGAAGATGAAGAAGAAAAGAAAAAACGTGAAGAAGACAAAGCCAAATTT

GAGAATCTCTGTAAAGTTATGAAAGATATTTTAGACAAAAAAGTAGAAAAAGTTGTAGTATCTAACAGGT

TAGTCGATTCTCCATGCTGTATTGTTACATCACAATATGGATGGACTGCAAATATGGAAAGAATTATGAA

AGCTCAAGCTCTCCGAGATGCATCTACTATGGGATACATGGCTGCCAAAAAGCATTTAGAAATTAATCCA

GATCATCCAATTATGGAAAACTTGAGGCAAAAGGCTGAAGCTGACAAGCATGATAAGTCTGTGAAAGATT

TGGTCATGCTTCTTTTTGAAACAGCTTTGTTGTCTTCTGGCTTTGCCCTTGAAGATCCACAAGTACATGC

TTCCAGAATATATAGAATGATTAAACTTGGTCTTGGTTTTGATGATGATGACACACCAAATGTAGAGGAT

GAAAAAATGGATACAGAAGTTCCTCCACTAGAAGATGACACAGAAGAAGCATCTAGGATGGAAGAAGTAG

ATTAA

>scaffold01062_*Apis_florea*_724aa

MPEDVTMANAGEVETFAFQAEIAQLMSLIINTFYSNKEIFIRELISNASDALDKIRYESLTDPSKLDTCK

ELFIKIVPNKNDRTLTILDSGIGMTKADLVNNLGTIAKSGTKAFMEALQAGADISMIGQFGVGFYSAYLV

ADKVVVISKHNDDEQYVWESSAGGSFTVRPDNGEPIGRGTKIILHIKEDQTEYLEESKIKEIVKKHSQFI

GYPIKLVVEKERDKELSEDEEEEEEPAKEEGEDTGKPKIEEVGEDEDEDKPKDEKKKKKKTIKEKYTEDE

ELNKTKPIWTRNPDDISQEEYGEFYKSLTNDWEDHLAVKHFSVEGQLEFRALLFIPRRAPFDLFENKKRK

NNIKLYVRRVFIMDNCEDLIPEYLNFIKGVVDSEDLPLNISREMLQQNKILKVIRKNLVKKCLELFEELS

EDKESYKKCYEQFSKNIKLGIHEDSQNRKKLSELLRYHTSASGDEMCSLKDYVGRMKENQKHIYYITGES

REQVANSSFVERVKKRGFEVVYMTEPIDEYVVQQLKEFDGKQLVSVTKEGLELPEDEEEKKKREEDKAKF

ENLCKVMKDILDKKVEKVVVSNRLVDSPCCIVTSQYGWTANMERIMKAQALRDASTMGYMAAKKHLEINP

DHPIMENLRQKAEADKHDKSVKDLVMLLFETALLSSGFALEDPQVHASRIYRMIKLGLGFDDDDTPNVED

EKMDTEVPPLEDDTEEASRMEEVD

>scaffold00044_*Atta_cephalotes*_cds_2175bp

ATGCCTGAGGACGTGAGCATGACGGATGCCGGTGAGGTGGAGACCTTCGCCTTCCAGGCCGAAATCGCTC

AATTGATGAGCCTGATCATCAACACCTTTTATTCGAATAAGGAGATTTTCATCCGAGAATTGATCTCCAA

CTCGTCCGATGCGTTGGACAAAATTCGATATGAATCTCTCACGGATCCATCCAAGTTGGATACGTGTAAG

GAATTGTTTATTAAGATCGTACCAAACAAGAACGACCGTACCCTGACGATCCTTGATTCTGGTATTGGCA

TGACCAAAGCCGATCTTGTAAATAATTTGGGTACCATTGCCAAATCTGGTACAAAGGCGTTCATGGAAGC

TCTGCAGGCAGGTGCTGATATCTCCATGATTGGACAGTTCGGTGTAGGTTTTTACTCGGCGTACCTCGTG

GCCGATAAAGTCATCGTGATCTCCAAACACAACGACGACGAACAATATCTGTGGGAGTCTTCCGCTGGTG

GTTCCTTCACGGTTCGTCCCGACAATGGTGAACCGATCGGACGGGGCACCAAGATCATCCTTCATATTAA

GGAGGATCAGACAGAATATCTGGAGGAGTCTAAAATCAAAGAAATCGTCAAGAAGCATTCGCAGTTCATC

GGCTATCCTATCAAGCTCGTCGTCGAGAAAGAACGTGACAAGGAACTGAGCGAGGACGAGGAGGAAGAGG

AAGAGAAAAAGGAAGAAGAAAAGGCAGAGGATGACAAACCCAAGATCGAAGATGTCGGCGAGGATGAGGA

AGAGGATAAGCCTAAGGAAGAGAAGAAAAAGAAGAAGAAGACGATCAAGGAAAAATACACTGAAGATGAG

GAGTTGAACAAGACAAAACCGATTTGGACAAGAAATCCGGATGACATCACGCAGGAAGAGTATGGCGAGT

TCTATAAGAGCTTGACTAACGATTGGGAAGACCACTTGGCTGTGAAACACTTCTCCGTGGAAGGACAGTT

GGAATTCAGAGCGCTGCTGTTTATTCCACGTCGTGCACCTTTCGATTTGTTTGAGAATAAGAAGAGAAAG

AACAATATTAAATTGTACGTACGTCGTGTTTTTATCATGGATAACTGCGAAGACCTCATTCCGGAATATC

TAAACTTTATCAAGGGTGTCGTAGACAGCGAGGATCTGCCTCTTAACATTTCTCGTGAGATGCTGCAACA

GAATAAGATTCTGAAGGTCATTAGGAAGAATCTTGTCAAGAAATGCTTGGAACTCTTCGAGGAATTGTCG

GAAGACAAGGAGAACTACAAAAAATGTTACGAGCAATTCAGCAAGAATCTGAAATTGGGTATTCACGAGG

ACAGCCAGAACAGGAAAAAGCTTTCAGAGTTGCTTCGCTATCACACTTCTGCATCCGGCGATGAGATGTG

CTCACTTAAAGATTACGTTGGTAGAATGAAGGAGAACCAGAAGCACGTCTACTACATCACTGGCGAGAGC

AGGGAGCAGGTAGCCAATAGCTCGTTTGTAGAACGCGTGAAGAAGCGTGGCTTCGAGGTTGTGTACATGA

CTGAGCCCATTGACGAGTATGTCGTCCAGCAACTGAAAGAATTCGACGGAAAACAGTTGGTATCTGTTAC

AAAAGAGGGCTTAGAACTTCCAGAAGACGAAGATGAGAAAAAGAAGCGCGAGGAAGACAAGGCCAAGTTC

GAGAGCCTTTGTAAAGTTATGAAGGACATCTTAGATAAGAAAGTGGAGAAAGTTGTAGTATCTAACAGAT

TGGTTGACTCTCCTTGTTGTATCGTCACATCGCAGTATGGCTGGACGGCGAACATGGAGAGAATCATGAA

GGCGCAAGCACTTCGAGACACATCCACCATGGGATACATGGCCGCGAAGAAACACCTCGAGATCAATCCC

GATCATCCTATCATGGAAAACTTAAGACAAAAGGCTGAGGCAGATAAGCACGATAAGTCGGTGAAAGATC

TAGTCATGTTGCTCTTTGAGACTGCACTCTTGTCGTCCGGTTTTGCCCTCGAAGATCCGCAAGTACATGC

TTCCAGAATATATAGAATGATCAAGTTAGGTCTAGGTTTTGACGACGAAGATACGTCGAACGCAGAGGAC

GAGAAGATGGACATGGAAGTGCCCACGTTGGAGGGTGACTCAGAAGAAGCGTCGAGAATGGAAGAAGTGG

ATTAA

>scaffold00044_*Atta_cephalotes*_724aa

MPEDVSMTDAGEVETFAFQAEIAQLMSLIINTFYSNKEIFIRELISNSSDALDKIRYESLTDPSKLDTCK

ELFIKIVPNKNDRTLTILDSGIGMTKADLVNNLGTIAKSGTKAFMEALQAGADISMIGQFGVGFYSAYLV

ADKVIVISKHNDDEQYLWESSAGGSFTVRPDNGEPIGRGTKIILHIKEDQTEYLEESKIKEIVKKHSQFI

GYPIKLVVEKERDKELSEDEEEEEEKKEEEKAEDDKPKIEDVGEDEEEDKPKEEKKKKKKTIKEKYTEDE

ELNKTKPIWTRNPDDITQEEYGEFYKSLTNDWEDHLAVKHFSVEGQLEFRALLFIPRRAPFDLFENKKRK

NNIKLYVRRVFIMDNCEDLIPEYLNFIKGVVDSEDLPLNISREMLQQNKILKVIRKNLVKKCLELFEELS

EDKENYKKCYEQFSKNLKLGIHEDSQNRKKLSELLRYHTSASGDEMCSLKDYVGRMKENQKHVYYITGES

REQVANSSFVERVKKRGFEVVYMTEPIDEYVVQQLKEFDGKQLVSVTKEGLELPEDEDEKKKREEDKAKF

ESLCKVMKDILDKKVEKVVVSNRLVDSPCCIVTSQYGWTANMERIMKAQALRDTSTMGYMAAKKHLEINP

DHPIMENLRQKAEADKHDKSVKDLVMLLFETALLSSGFALEDPQVHASRIYRMIKLGLGFDDEDTSNAED

EKMDMEVPTLEGDSEEASRMEEVD

>Cont3.343_*Culex quinquefasciatus*_cds_216bp

ATGCCGGAAGTCGAGACCGAGACCTTTGCGTTCCAGGCTGAGATCGCTCAGCTGATGTCGCTGATCATCA

ACACCTTCTACTCGAACAAGGAAATCTTCCTGCGAGAACTGATCTCCAACTCTTCGGATGCCCTGGACAA

GATCCGCTACGAGTCGTTGACCGACCCCTCAAACTGGACAATGGCAAGGAGCTGTTCATCAAGCTGATCC

CGAACA

>Cont3.343_*Culex quinquefasciatus*_72aa

MPEVETETFAFQAEIAQLMSLIINTFYSNKEIFLRELISNSSDALDKIRYESLTDPSKLDNGKELFIKLI

PN

>scaffold1836_*Danaus_plexippus*_cds_2157bp

ATGCCTGAAGAAATGGAGACAGCGCCCGCAGAGGTGGAGACCTTCGCCTTCCAGGCGGAGATCGCCCAGC

TCATGTCATTGATCATCAACACCTTCTATTCAAACAAAGAAATTTTCCTCCGTGAGTTAATCTCAAATTC

CTCCGACGCTTTAGACAAGATCCGCTATGAATCACTTACGGATCCCTCGAAGCTTGACAGTGGTAAGGAA

TTGTACATTAAAATTATTCCCAACAAGAGTGAGGGAACCCTTACAATTATTGATACCGGTATTGGTATGA

CCAAGGCTGACCTCGTGAACAACTTGGGTACCATCGCTAAGTCAGGTACTAAGGCGTTCATGGAAGCATT

GCAAGCTGGAGCCGATATCAGCATGATTGGTCAATTTGGTGTGGGTTTCTACTCTTGCTACTTGGTAGCC

GACAGGGTCACAGTACATTCCAAGCACAATGATGACGAGCAATATGTGTGGGAGTCGGCAGCGGGTGGAT

CCTTCACAGTCCGCCCTGACCAAGGTGAGCCCCTCGGACGCGGTACCAAGATCGTGCTCCACGTTAAAGA

GGACTTAGCCGAATACATGGAAGAACATAAGATTAAAGAAATCGTCAAGAAACACTCTCAATTCATCGGC

TACCCCATCAAGTTAGTAGTAGAGAAGGAACGCGAAAAGGAGCTGTCTGATGATGAAGCCGAGGAAGAGA

AGAAAGAAGATGAGAAGGAAGACGAGAAGCCTAAGATTGAAGATGTTGGAGAAGATGATGAGGAAGACAG

CAAAGACAAGAAGAAAAAGAAGAAGACCATCAAGGAGAAGTACACAGAAGATGAAGAACTTAACAAGACG

AAGCCCATCTGGACAAGAAACGCCGATGACATCACCCAGGAGGAGTATGGTGACTTCTACAAATCCCTGA

CCAACGACTGGGAAGACCATTTGGCGGTCAAGCACTTCAGCGTTGAAGGACAGCTCGAATTCCGCGCTCT

CCTGTTTGTACCCCGCCGCGCTCCCTTCGATCTTTTTGAGAATAAGAAGCGCAAGAACAACATCAAGCTG

TACGTCCGCAGGGTTTTCATCATGGACAACTGTGAAGATCTTATTCCAGAATACCTGAATTTCATCAAGG

GTGTGGTCGACAGTGAGGATCTGCCTCTAAACATCTCTCGTGAGATGCTCCAACAGAACAAGATCCTTAA

GGTCATTAGGAAGAACTTAGTCAAGAAATGCCTTGAACTGTTTGAAGAGTTGGCCGAAGACAAGGAGAAC

TACAAGAAATACTACGAACAGTTCAGCAAGAACTTGAAACTCGGAATCCACGAGGACTCTCAAAATCGCT

CCAAACTAGCAGACTTGCTGCGTTACCACACCTCCGCCTCTGGCGATGAGGCGTGCTCTCTTAAGGAGTA

CGTGTCACGTATGAAGGAGAACCAGAAACACATCTACTACATCACTGGTGAAAACAGAGACCAAGTAGCC

AACTCTTCCTTCGTCGAGAGGGTCAAGAAACGTGGTTATGAAGTAGTGTACATGACCGAGCCGATCGACG

AGTATGTAGTACAACAGATGAGAGAATACGACGGCAAGACACTCGTCTCCGTAACAAAGGAAGGCTTGGA

GCTTCCCGAGGACGAGGAAGAGAAGAAGAAACGCGAGGAGGACAAAGTCAAGTTTGAGAATCTCTGCAAG

GTGATGAAGAACATCCTGGACAGCAAGGTGGAGAAGGTGGTCGTGTCCAACAGGCTGGTGGAATCTCCTT

GCTGTATTGTCACGGCGCAGTACGGATGGTCGGCTAACATGGAAAGGATCATGAAGGCCCAGGCTCTCCG

CGACACATCCACTATGGGATACATGGCGGCCAAGAAACACCTCGAGATCAACCCCGACCATTCCATCGTG

GAGACTCTTAGGCAGAAGGCCGAGGCCGACAAGAACGACAAGGCCGTTAAGGACTTGGTCATCTTGTTAT

ACGAGACTGCCTTGCTGTCGTCCGGCTTCGCCCTCGACGAGCCCCAGGTACACGCCTCCAGAATCTACCG

CATGATCAAGTTGGGTCTCGGCATCGACGAGGAGGAACCTATCCCGGTTGAAGAAGGAAGCGCCGGAGAC

GTGCCGCCGCTGGAGGGAGACGCCGACGACGCGTCTCGCATGGAGGAGGTCGATTAA

>scaffold1836_*Danaus_plexippus*_718aa

MPEEMETAPAEVETFAFQAEIAQLMSLIINTFYSNKEIFLRELISNSSDALDKIRYESLTDPSKLDSGKE

LYIKIIPNKSEGTLTIIDTGIGMTKADLVNNLGTIAKSGTKAFMEALQAGADISMIGQFGVGFYSCYLVA

DRVTVHSKHNDDEQYVWESAAGGSFTVRPDQGEPLGRGTKIVLHVKEDLAEYMEEHKIKEIVKKHSQFIG

YPIKLVVEKEREKELSDDEAEEEKKEDEKEDEKPKIEDVGEDDEEDSKDKKKKKKTIKEKYTEDEELNKT

KPIWTRNADDITQEEYGDFYKSLTNDWEDHLAVKHFSVEGQLEFRALLFVPRRAPFDLFENKKRKNNIKL

YVRRVFIMDNCEDLIPEYLNFIKGVVDSEDLPLNISREMLQQNKILKVIRKNLVKKCLELFEELAEDKEN

YKKYYEQFSKNLKLGIHEDSQNRSKLADLLRYHTSASGDEACSLKEYVSRMKENQKHIYYITGENRDQVA

NSSFVERVKKRGYEVVYMTEPIDEYVVQQMREYDGKTLVSVTKEGLELPEDEEEKKKREEDKVKFENLCK

VMKNILDSKVEKVVVSNRLVESPCCIVTAQYGWSANMERIMKAQALRDTSTMGYMAAKKHLEINPDHSIV

ETLRQKAEADKNDKAVKDLVILLYETALLSSGFALDEPQVHASRIYRMIKLGLGIDEEEPIPVEEGSAGD

VPPLEGDADDASRMEEVD

>contigGmorY1_*Glossina_morsitans*_cds_2148bp

ATGGTAGAAGTTGAAACATTTGCTTTCCAAGCTGAGATTGCTCAGCTTATGTCGCTGATTATCAACACAT

TCTATTCGAATAAAGAGATTTTCTTGCGTGAATTGATTTCTAATGCTTCTGATGCTTTGGATAAGATTCG

CTACGAATCGTTGATCGATCCATCGAAACTGGATACCGGCAAGGAACTTTACATCAAACTAATTCCTAAC

AAGACAGCTGGCACATTGACTATCCTAGATACGGGCATTGGCATGACTAAGTCCGACTTGGTTAACAATT

TAGGTACTATTGCAAAATCGGGCACAAAAGCTTTTATGGAAGCTTTGCAGGCCGGTGCCGATATTTCCAT

GATTGGTCAGTTCGGGGTGGGTTTCTATTCCGCTTATTTGGTCGCTGACAAAGTCACTGTCACATCAAAA

AATAACGATGACGAGCAGTACATTTGGGAATCATCAGCTGGCGGTTCGTTTACGGTGAAGTCTGATAATT

CCGAGCCTTTGGGTCGCGGTACTAAAATAGTTTTGCACATCAAGGAAGATCAGGCTGAATATTTGGAAGA

AAGTAAGATCAAGGAAATCGTGACTAAACACTCACAGTTTATAGGTTACTCTATCAAATTACTGGTAGAG

AAAGAGCGCGATCAAGAAATCAGCGATGACGACGCAGAAGATGATAAAAAGGAAGAAGGGAAGAAAGAGA

TGCAAACCGATGAACCAAAACTTGAGGATGTTGGCGAAGATGAAGATGCCGATAAAAAGGAAAAGGATAG

CAAAAAGAAAAAAACCTTGAAGGTTAAATACACCGAAGACGAGGAGTTGAATAAGACTAAGCCAATATGG

ACTCGCAATCCCGACAATATTTCTCAGGCCGAATATGGTGAATTCTATAAATCTTTGACTAATGACTGGG

AAGAACATTTGGCCGTTAAGCATTTTTCAGTAGAAGGTCAGTTGGAGTTCCGCGCTTTACTTTTCGTTCC

TCGGCGTATCCCATTCGATGCATTTGAAAACCAAAAGAAACGCAATAATATCAAATTGTACGTACGCCGC

GTCTTTATCATGGACAACTGCGAAGATCTCATTCCCGAATACTTGAATTTCATCAAAGGTGTTGTCGATT

CTGAGGATTTGCCATTGAACATTTCTCGTGAAATGTTGCAACAAAGTAAAGTCCTCAAAGTTATTCGCAA

GAATATCGTTAAGAAAACCATGGAACTAATCGAGGAGCTTACTGAAGATAAGGAGCTTTACAAAAAGTTT

TATGATCAATTCAATAAAAATCTCAAATTGGGTGTTCACGAAGATAGCAGCAATCGCGCCAAATTAGCCG

ATTTCTTACGCTTCCACACCTCGGCTTCTGGCGATGACTTCTGCTCATTAGCTGATTATGTGTCACGTAT

GAAGGATATTCAAAAATATATCTACTTCATCACTGGTGAATCCAAAGAACAAGTGGCCGATTCGGCTTTC

GTTGAACGAGTTAAAGCGCGTGGTTTTGAAGTAGTATACATGACCGACCCTATTGATGAGTATATAATTC

AGCACTTGAAAGAATATAAGGGCAAACAGTTGGTGTCTGTAACCAAAGAAGGTTTGGAATTACCAGAGAC

CGATGCTGAAAAGAAGAAACGTGAAGAAGATAAAGCTAAATTGGAAAATTTGTGCAAGCTTATGAAATCG

ATACTCGACAACAAGATAGACAAAGTCACTGTGTCAAATCGTTTAGTTGAATCGCCATGCTGTATTGTCA

CCTCTCAGTATGGCTGGTCAGCCAATATGGAACGTATTATGAAAGCCCAGGCTTTGCGCGACACCTCTAC

CATGGGCTACATGTCTGGCAAGAAGCATTTAGAAATCAATCCTGATCATCCCATTATCGAGACATTACGA

CAAAAAGCTGAAGTCGATAAAAATGACAAAGCTGTAAAAGATTTGGTCATTTTGTTGTTCGAAACTTCTT

TATTGTCATCTGGCTTCTCTCTACAAAGCCCTCAAACTCATGCCAGCCGCATTTATCGTATGATAAAGCT

CGGCCTTGGCATTGACGATGATGAACCAATAGTTACTGAAGATGCTCAAAGTGTCGGTGATGCTCCGCCT

TTAATGGATGACTCTGAGGACGCATCCCATATGGAGGAAGTTGATTAG

>contigGmorY1_*Glossina_morsitans*_715aa

MVEVETFAFQAEIAQLMSLIINTFYSNKEIFLRELISNASDALDKIRYESLIDPSKLDTGKELYIKLIPN

KTAGTLTILDTGIGMTKSDLVNNLGTIAKSGTKAFMEALQAGADISMIGQFGVGFYSAYLVADKVTVTSK

NNDDEQYIWESSAGGSFTVKSDNSEPLGRGTKIVLHIKEDQAEYLEESKIKEIVTKHSQFIGYSIKLLVE

KERDQEISDDDAEDDKKEEGKKEMQTDEPKLEDVGEDEDADKKEKDSKKKKTLKVKYTEDEELNKTKPIW

TRNPDNISQAEYGEFYKSLTNDWEEHLAVKHFSVEGQLEFRALLFVPRRIPFDAFENQKKRNNIKLYVRR

VFIMDNCEDLIPEYLNFIKGVVDSEDLPLNISREMLQQSKVLKVIRKNIVKKTMELIEELTEDKELYKKF

YDQFNKNLKLGVHEDSSNRAKLADFLRFHTSASGDDFCSLADYVSRMKDIQKYIYFITGESKEQVADSAF

VERVKARGFEVVYMTDPIDEYIIQHLKEYKGKQLVSVTKEGLELPETDAEKKKREEDKAKLENLCKLMKS

ILDNKIDKVTVSNRLVESPCCIVTSQYGWSANMERIMKAQALRDTSTMGYMSGKKHLEINPDHPIIETLR

QKAEVDKNDKAVKDLVILLFETSLLSSGFSLQSPQTHASRIYRMIKLGLGIDDDEPIVTEDAQSVGDAPP

LMDDSEDASHMEEVD

>DS942764_*Ixodes_scapularis*_cds_627bp

ATGCCCGAAGAAGTGCGCATGGAGGATGCCGCCGGCGAGGCGGAGACCTTCGCTTTCCAGGCGGAGATCG

CTCAGCTTATGAGCCTGATCATCAACACCTTCTACTCCAACAAGGAGATCTTCCTCCGGGAGCTCATCTC

CAACTCGTCCGACGCTCTGGACAAGATCCGCTACGAGTCTCTCACAGACCCGACCAAGCTGGACGCCCAG

AAGGAGCTCTTCATCAAGATCATCCCCAACCGCGATGACCGGACCCTGACCCTCATCGACACTGGCATTG

GCATGACCAAGGCCGACCTTATCAACAACCTGGGCACAATCGCAAAGTCCGGCACCAAGGCATTTATGGA

AGCGCTGCAGGCCGGCGCCGACATCAGCATGATCGGCCAGTTCGGTGTGGGATTCTACTCGGCGTACCTG

GTCGCCGACAAGGTGACTGTCACGTCCAAACACAACGACGACGAGCAGTACACGTGGGAGTCGTCCGCCG

GCGGTTCCTTCACCATCCGCACGGACAACTGCGAACCGCTGGGACGCGGCACCAAGATCGTGCTGCACCT

CAAGGAAGACCAGACTGAGTACCTGGAGGGACGCCGCATTAAGGACGTTGTGAAGAAGCACTCGCAG

>DS942764_*Ixodes_scapularis*_209aa

MPEEVRMEDAAGEAETFAFQAEIAQLMSLIINTFYSNKEIFLRELISNSSDALDKIRYESLTDPTKLDAQ

KELFIKIIPNRDDRTLTLIDTGIGMTKADLINNLGTIAKSGTKAFMEALQAGADISMIGQFGVGFYSAYL

VADKVTVTSKHNDDEQYTWESSAGGSFTIRTDNCEPLGRGTKIVLHLKEDQTEYLEGRRIKDVVKKHSQ

>scf7180001004854_*Linepithema_humile*_cds_2172bp

ATGCCAGAAGACGTATCCATGGAGAACGCCGGCGAGGTGGAGACCTTCGCCTTTCAGGCCGAAATTGCTC

AATTAATGAGCTTAATTATCAATACCTTCTATTCGAACAAGGAAATCTTTATCCGAGAATTGATCTCAAA

CTCGTCTGATGCGTTGGACAAAATTCGATATGAATCTCTTACGGAGCCATCCAAGCTTGATACATGCAAA

GACCTGTTCATCAAAATTGTCCCGAATAAGAATGATCGCACTCTTACTATTCTTGATTCTGGTATTGGTA

TGACCAAGGCCGATCTCGTGAATAACTTAGGTACCATCGCTAAATCTGGCACCAAGGCATTTATGGAAGC

TCTGCAAGCAGGCGCTGACATTTCCATGATTGGACAGTTTGGCGTAGGTTTCTACTCGGCATATCTTGTG

GCTGATAGGGTCATCGTTATCTCGAAGCACAATGATGACGAGCAATATTTGTGGGAGTCTTCCGCCGGTG

GTTCGTTTACTGTACGCCCTGACAATGGCGAGCCGATCGGCCGTGGCACGAAAATTGTTTTGCACATCAA

GGAGGACCAGACTGAATACCTGGAAGAAGCAAAGATTAAAGAGATTGTGAAAAAGCACTCTCAATTCATT

GGCTATCCCATCAAGCTCGTTGTGGAGAAGGAACGTGATAAGGAACTGAGCGAGGATGAGGAGGAGGAGC

CTGCAAAGGAAGAAGAAAAAACTGAGGACGACAAGCCTAAGATCGAGGATGTCGGCGAGGATGAGGATGA

GGACAAGCCCAAAGACGAGAAGAAGAAGAAGAAGAAGACCATCAAGGAGAAGTACACTGAGGACGAGGAA

CTCAACAAGACAAAGCCGATCTGGACGAGAAATCCAGATGACATCACGCAGGAAGAATATGGCGAATTCT

ACAAGAGCTTGACTAATGACTGGGAGGATCATTTAGCTGTGAAACACTTTTCCGTGGAAGGACAGCTGGA

ATTTAGGGCGCTGTTGTTTATCCCGCGTCGTGCGCCTTTCGATTTGTTCGAGAATAAAAAGAGGAAGAAC

AACATCAAGTTGTACGTACGACGCGTCTTCATCATGGACAATTGCGAGGACCTGATTCCAGAATATCTGA

ACTTCATCAAGGGCGTTGTGGATAGCGAGGATCTACCTTTGAACATCTCTCGTGAGATGTTGCAGCAGAA

TAAGATCTTGAAGGTTATCAGGAAGAATCTCGTTAAGAAATGCTTGGAACTCTTCGAGGAATTGGCTGAG

GATAGGGAGAATTACAAGAAGTGCTACGAGCAGTTCAGCAAGAATCTGAAACTCGGCATCCACGAGGACA

GCCAGAACAGGAAGAAGCTATCCGAATTGCTCCGTTATCACACTTCCGCATCCGGTGATGAGTTGTGCTC

GCTAAAGGACTACGTCGGTAGGATGAAGGAGAACCAGAAACATGTCTACTACATCACCGGCGAGAGCAGG

GAACAAGTCGCCAACAGCTCGTTTGTTGAACGCGTGAAGAAGCGCGGCTTTGAGGTCGTGTACATGACTG

AGCCCATCGACGAGTACGTCGTTCAACAGCTGAAGGAATTTGACGGAAAGCAGCTGGTGTCTGTCACGAA

AGAGGGCTTGGAATTGCCGGAGGATGAGGAGGAGAAGAAGAAGCGCGAGGAGGACAAGGCCAAGTTCGAG

AGTCTCTGCAAAGTCATGAAGGATATTCTCGACAAGAAGGTCGAAAAGGTGGTGGTGTCCAACAGGCTGG

TCGACTCGCCATGCTGCATCGTCACGTCGCAGTACGGTTGGACGGCGAACATGGAGAGGATCATGAAGGC

CCAGGCGCTCCGAGATACATCCACCATGGGTTACATGGCCGCAAAGAAACACTTGGAGATCAATCCGGAT

CATCCCATCATGGAGAACTTGCGGCAAAAGGCTGAGGTTGACAAACATGACAAATCTGTTAAAGATTTAG

TCATGTTGCTGTTCGAGACTGCTCTCTTGTCCTCTGGTTTCGCACTTGAGGATCCGCAAGTACACGCATC

TAGAATATATAGAATGATCAAACTCGGCTTGGGCTTTGAGGACGAAGACACGCCTAATGCTGAGGACGAT

AAGATGGAAACTGAAGTGCCGACTTTAGAAGGTGACGCCGAAGAGGCGTCGAGGATGGAAGAAGTAGATT

AA

>scf7180001004854_*Linepithema_humile*_723aa

MPEDVSMENAGEVETFAFQAEIAQLMSLIINTFYSNKEIFIRELISNSSDALDKIRYESLTEPSKLDTCK

DLFIKIVPNKNDRTLTILDSGIGMTKADLVNNLGTIAKSGTKAFMEALQAGADISMIGQFGVGFYSAYLV

ADRVIVISKHNDDEQYLWESSAGGSFTVRPDNGEPIGRGTKIVLHIKEDQTEYLEEAKIKEIVKKHSQFI

GYPIKLVVEKERDKELSEDEEEEPAKEEEKTEDDKPKIEDVGEDEDEDKPKDEKKKKKKTIKEKYTEDEE

LNKTKPIWTRNPDDITQEEYGEFYKSLTNDWEDHLAVKHFSVEGQLEFRALLFIPRRAPFDLFENKKRKN

NIKLYVRRVFIMDNCEDLIPEYLNFIKGVVDSEDLPLNISREMLQQNKILKVIRKNLVKKCLELFEELAE

DRENYKKCYEQFSKNLKLGIHEDSQNRKKLSELLRYHTSASGDELCSLKDYVGRMKENQKHVYYITGESR

EQVANSSFVERVKKRGFEVVYMTEPIDEYVVQQLKEFDGKQLVSVTKEGLELPEDEEEKKKREEDKAKFE

SLCKVMKDILDKKVEKVVVSNRLVDSPCCIVTSQYGWTANMERIMKAQALRDTSTMGYMAAKKHLEINPD

HPIMENLRQKAEVDKHDKSVKDLVMLLFETALLSSGFALEDPQVHASRIYRMIKLGLGFEDEDTPNAEDD

KMETEVPTLEGDAEEASRMEEVD

>scf7180001005039_*Linepithema_humile*_cds_2172bp

ATGCCAGAAGACGTGACCATGGAGAACGCCGGCGAGGTGGAGACCTTTGCCTTTCAGGCTGAAATCGCTC

AATTAATGAGCTTAATCATCAATACCTTCTACTCGAACAAAGAAATCTTTATCCGAGAATTAATCTCAAA

CTCGTCTGATGCGTTGGACAAAATTCGATATGAATCTCTTACGGAGCCATCCAAGCTTGATACATGCAAA

GAACTGTTCATCAAAATCGTCCCGAACAAGAATGACCGCACTCTCACAATTCTTGATTCTGGTATTGGTA

TGACCAAGGCTGATCTCGTGAATAACTTGGGTACCATCGCTAAATCTGGCACCAAAGCGTTTATGGAAGC

TCTGCAGGCAGGTGCTGACATTTCCATGATTGGACAGTTTGGTGTAGGTTTCTATTCGGCATATCTTGTG

GCTGATAGAGTCATCGTAATCTCGAAGCACAATGATGATGAGCAGTATTTGTGGGAGTCTTCCGCCGGTG

GTTCGTTTACGGTACGCCCGGACAATGGCGAGCCGATCGGTCGTGGCACAAAAATTGTTTTGCACATCAA

GGAGGACCAGACTGAATACTTGGAAGAGTCAAAGATTAAGGAGATTGTAAAGAAGCACTCTCAGTTCATT

GGCTATCCCATTAAGCTCGTTGTAGAGAAGGAACGTGATAAGGAACTGAGCGAGGATGAGGAGGAGGAGC

CCGCAAAGGAGGAAGAAAAAACTGAGGACGACAAGCCTAAGATCGAGGATGTCGGCGAGGATGAGGATGA

GGATAAGCCCAAAGAGGAGAAAAAGAAGAAGAAGAAGACCATCAAAGAGAAGTACACAGAGGACGAAGAG

CTCAACAAGACAAAGCCGATCTGGACGAGAAATCCAGATGAAATCACGCAGGAAGAATATGGCGAATTCT

ATAAGAGCTTGACGAATGACTGGGAGGACCACTTGGCTGTGAAGCACTTCTCCGTGGAAGGACAGCTGGA

ATTCAGGGCGCTGCTGTTCATCCCGCGTCGTGCGCCTTTCGACTTGTTCGAAAACAAAAAGAGGAAGAAC

AACATTAAGTTGTACGTACGACGCGTCTTCATCATGGACAATTGTGAGGACCTGATTCCAGAATATTTGA

ACTTCATCAAGGGCGTCGTAGACAGTGAGGATCTTCCTTTGAACATCTCCCGTGAGATGTTACAGCAGAA

TAAGATCCTGAAGGTTATCAGGAAGAATCTCGTTAAGAAATGCTTGGAGCTCTTCGAGGAATTGGCCGAG

GACAAGGAAAATTACAAGAAGTGCTACGAGCAATTCAGCAAAAATCTGAAACTCGGCATCCACGAAGACA

GCCAAAACAGGAAAAAGCTGTCGGAATTGCTCCGCTATCACACTTCCGCATCCGGCGATGAAATGTGCTC

GCTGAAAGACTACGTCGGCAGGATGAAGGAGAATCAGAAACATGTCTACTACATCACCGGCGAGAGCAGG

GAGCAAGTCGCTAACAGCTCGTTCGTTGAACGTGTGAAGAAGCGCGGCTTCGAGGTCGTGTATATGACTG

AGCCCATTGACGAGTACGTCGTTCAACAGCTGAAAGAATTCGATGGAAAGCAATTGGTGTCCGTCACGAA

AGAGGGCTTGGAATTGCCGGAGGATGAGGAGGAGAAGAAGAAACGCGAGGAGGACAAAACCAAATTCGAG

AGTCTCTGCAAAATTATGAAGGACATTCTCGACAAGAAGGTCGAAAAGGTAGTAGTATCGAACAGACTGG

TCGACTCGCCATGCTGCATCGTCACGTCGCAATACGGTTGGACGGCGAACATGGAGAGGATCATGAAGGC

TCAGGCGCTCCGAGATACATCCACCATGGGATACATGGCCGCAAAGAAACACTTGGAGATCAATCCGGAT

CATCCCATCATGGAGAACTTGCGGCAAAAGGCTGAGGCTGACAAGCATGACAAATCGGTTAAAGACTTGG

TCATGTTGCTGTTCGAGACTGCTCTCTTGTCCTCTGGTTTCGCGCTCGAGGATCCACAAGTACACGCATC

TAGAATATATAGAATGATCAAACTCGGCTTGGGCTTTGATGACGAGGACACGCCGAACGCTGAGGACGAT

AAGATGGAAACGGAAGTGCCGGCGTTAGAAGGTGACGCTGAAGAGGCGTCGAGGATGGAAGAAGTAGATT

AA

>scf7180001005039_*Linepithema_humile*_723aa

MPEDVTMENAGEVETFAFQAEIAQLMSLIINTFYSNKEIFIRELISNSSDALDKIRYESLTEPSKLDTCK

ELFIKIVPNKNDRTLTILDSGIGMTKADLVNNLGTIAKSGTKAFMEALQAGADISMIGQFGVGFYSAYLV

ADRVIVISKHNDDEQYLWESSAGGSFTVRPDNGEPIGRGTKIVLHIKEDQTEYLEESKIKEIVKKHSQFI

GYPIKLVVEKERDKELSEDEEEEPAKEEEKTEDDKPKIEDVGEDEDEDKPKEEKKKKKKTIKEKYTEDEE

LNKTKPIWTRNPDEITQEEYGEFYKSLTNDWEDHLAVKHFSVEGQLEFRALLFIPRRAPFDLFENKKRKN

NIKLYVRRVFIMDNCEDLIPEYLNFIKGVVDSEDLPLNISREMLQQNKILKVIRKNLVKKCLELFEELAE

DKENYKKCYEQFSKNLKLGIHEDSQNRKKLSELLRYHTSASGDEMCSLKDYVGRMKENQKHVYYITGESR

EQVANSSFVERVKKRGFEVVYMTEPIDEYVVQQLKEFDGKQLVSVTKEGLELPEDEEEKKKREEDKTKFE

SLCKIMKDILDKKVEKVVVSNRLVDSPCCIVTSQYGWTANMERIMKAQALRDTSTMGYMAAKKHLEINPD

HPIMENLRQKAEADKHDKSVKDLVMLLFETALLSSGFALEDPQVHASRIYRMIKLGLGFDDEDTPNAEDD

KMETEVPALEGDAEEASRMEEVD

>cont2256_*Mayetiola_destructor*_cds_2145bp

ATGCCAGAAGAAGTTGAAACTTTTGCATTTCAAGCTGAAATTGCTCAGCTTATGTCATTGATCATCAACA

CCTTCTACTCGAACAAAGAAATTTTCCTTCGAGAATTGATTTCCAATTCATCCGATGCTTTGGATAAGAT

CCGTTATGAATCACTCACCGATCCATCAAAATTGGACAGCGGCAAAGAATTGTACATTAAAATTATTCCA

AACAAAGCTGCTGGTACACTCACCATTATTGATACTGGTATTGGTATGACAAAAGCCGATTTGGTCAACA

ATCTTGGTACAATTGCCAAATCAGGAACCAAAGCTTTCATGGAAGCTTTGCAAGCTGGTGCTGATATTAG

TATGATTGGTCAATTTGGTGTCGGTTTCTATTCCGCTTATTTGGTTGCCGATCGTGTTACCGTTACATCG

AAACATAATGACGATGAACAATATGTTTGGGAATCGTCAGCCGGTGGCTCATTCACCATTCGTACCGATA

ATTCGGAACCACTTGGTCGCGGTACAAAAATTGTTTTGCACATCAAAGAAGATCAAACCGAATACTTGGA

AGAAAGTAAAATCAAGAGCATTGTCACCAAGCACAGTCAATTTATTGGCTATCCAATTAAATTGTTGGTC

GAAAAAGAGCGCGAACAAGAAGTAAGCGACGACGAGGCTGAAGATGAAAAGGAAAAAAAAGAAGAAGAAC

CAAAGAAGGAATCCGAAGAGGGTGAAGAGCCCAAGATCGAAGATGTTGACGAAACTACCGAAGAAAAAAA

GAAAAAGAAAACGATCAAAGTAAAATACACTGAAGACGAAGAGTTGAACAAAACAAAACCAATTTGGACC

CGTAATGCTGACGACATTTCACAAGAAGAATACGGTGAATTTTATAAGTCACTAACCAACGATTGGGAAG

ATCATCTTGCCGTCAAACATTTCAGTGTTGAAGGTCAACTCGAATTCCGTGCATTGTTGTTTGTTCCACG

CCGTGTCCCATTCGATTTGTTCGAAAACAAAAAGAAGAAGAACAACATCAAATTGTACGTTCGTCGTGTA

TTCATCATGGATAACTGCGAGGATTTGATACCAGACTACTTGAATTTTATTCGTGGTGTTGTCGATTCCG

AAGATTTACCATTGAATATTTCTCGTGAAATGTTGCAACAAAACAAAATTCTCAAGGTTATCCGCAAAAA

TATCGTCAAAAAGTGTTTGGAACTTTTCGAAGAACTTGCTGAGGACAAAGAAAATTACAAGAAATTCTAT

GCTCAATTCAGCAAAAATTTGAAATTGGGCGTTCACGAAGACAGTCAAAACCGTGCAAAATTGGCAGAAT

TACTTCGATTCCATACATCAGCATCTGGTGATGAAGAGACTTCATTGGCTGATTACGTTAGCCGCATGAA

GGAAAACCAAAAACACATCTATTGTATCAGCGGTGAAAGCCGTGAACAAGTTTCAAATTCATCATTTGTT

GAACGTCTCAAGAAACGTGGCTACGAAATCATTTACATGACTGAACCAATTGATGAATATGTCATTCAAC

AATTGAAAGAATTCCAAGGAAAGCAATTGGTATCAGTTACCAAAGAAGGTTTGGAATTGCCAGAAGATGA

TGAAGAAAAGAAAAAGATCGAAGAAGACAAAGCCAAATTCGAAGGCTTGTGCAAAGTAATCAAATCCGTA

TTGGACAACAAAGTTGAAAAAGTTCTTGTTTCACATCGTTTGGTGCAATCACCATGCTGCGTTGTTACAT

CGCAATACGGTTGGTCCGCCAACATGGAACGTATTATGAAAGCTCAAGCACTTCGTGACACCTCCACCCT

TGGCTACATGGCCGGCAAAAAACATTTGGAAATCAATCCAGACCATCCAATCATTGAGACATTACGTCAA

AAAGCCGATGCCGACAAAAATGACAAATCAGTTAAAGATTTGGTTATTTTGTTGTTCGAAACAGCTTTAC

TTTCATCCGGTTTTTCATTGGAAGAGCCACAAGTTCATGCATCCCGTATCTACCGCATGATCAAATTGGG

TCTTGGCATTGATGAAGACGAACCAATGTCCGCCGAAGAGACACCAGCTGCTGGCGATTTGCCACCATTG

GTCGATGACGCAGAAGATGCATCCCATATGGAAGAAGTTGATTAA

>cont2256_*Mayetiola_destructor*_714aa

MPEEVETFAFQAEIAQLMSLIINTFYSNKEIFLRELISNSSDALDKIRYESLTDPSKLDSGKELYIKIIP

NKAAGTLTIIDTGIGMTKADLVNNLGTIAKSGTKAFMEALQAGADISMIGQFGVGFYSAYLVADRVTVTS

KHNDDEQYVWESSAGGSFTIRTDNSEPLGRGTKIVLHIKEDQTEYLEESKIKSIVTKHSQFIGYPIKLLV

EKEREQEVSDDEAEDEKEKKEEEPKKESEEGEEPKIEDVDETTEEKKKKKTIKVKYTEDEELNKTKPIWT

RNADDISQEEYGEFYKSLTNDWEDHLAVKHFSVEGQLEFRALLFVPRRVPFDLFENKKKKNNIKLYVRRV

FIMDNCEDLIPDYLNFIRGVVDSEDLPLNISREMLQQNKILKVIRKNIVKKCLELFEELAEDKENYKKFY

AQFSKNLKLGVHEDSQNRAKLAELLRFHTSASGDEETSLADYVSRMKENQKHIYCISGESREQVSNSSFV

ERLKKRGYEIIYMTEPIDEYVIQQLKEFQGKQLVSVTKEGLELPEDDEEKKKIEEDKAKFEGLCKVIKSV

LDNKVEKVLVSHRLVQSPCCVVTSQYGWSANMERIMKAQALRDTSTLGYMAGKKHLEINPDHPIIETLRQ

KADADKNDKSVKDLVILLFETALLSSGFSLEEPQVHASRIYRMIKLGLGIDEDEPMSAEETPAAGDLPPL

VDDAEDASHMEEVD

>scaffold_Un.16375*_Mayetiola_destructor*_cds_513bp

GCTTTGGACAAAATCCGCTATGAATCACTCACTGGTCCATCAAAACTCGACAGCGGCAAGGAATTGTACA

TCAAAATCATTCCAAACACAGCCGCCGGTACTCTTACCATCATTGATACTGGTATTGGTATGACTAAAGC

CGATTTGGTCAACAATCTTGGTACAATAGCTAAATCCGAAACAAAGGCATCCATGGAGGCATTGCAAGCT

GGTGCTGACATCAGTAAGATTGGACAATTCGGTGTTGGTTTCTACTTAGCCTATTTGGTTGCCGATAAGG

TCACCGTTACATCGAAACACAACGATGATGAGCAATACATTTGGGAGTCGTCAGCTGGCGGTTCATTCAC

CATTCGTGCCGATAACTCCGAACCATTGGGCCGTGGCGCCAAGATCTTTTTGCACATCAAAGAAGACCAA

ACCGAATACTTGGAAGAAAGCAAAATCAAGCAAATCTTCACCAAGGTATCTGGATTTATTCGCTATCCAG

TCCAGTTGGTCAAAGAAGAGCGC

>scaffold_Un.16375*_Mayetiola_destructor*_171aa

ALDKIRYESLTGPSKLDSGKELYIKIIPNTAAGTLTIIDTGIGMTKADLVNNLGTIAKSETKASMEALQA

GADISKIGQFGVGFYLAYLVADKVTVTSKHNDDEQYIWESSAGGSFTIRADNSEPLGRGAKIFLHIKEDQ

TEYLEESKIKQIFTKVSGFIRYPVQLVKEER

>scaffold1_*Nasonia_giraulti*_cds_1897>bp

ATGCCGGAGGACGTCGTAATGGAACAAAGCGGCGAGGTGGAAACCTTCGCCTTCCAGGCCGAAATCGCCC

AGCTTATGAGCCTGATCATCAACACCTTCTACTCGAACAAGGAGATCTTCATTCGAGAATTGATTTCCAA

CTCGTCTGATGCCTTGGATAAGATTCGCTATGAGTCGCTCACAGATCCATCCAAGCTCGACTCGTGCAAG

GAGCTGTTCATCAAGATCATCCCTAACAAGAACGATCGCACACTCACCATCATTGACTCTGGAATTGGTA

TGACAAAGGCTGACTTGGTGAACAACCTTGGTACCATCGCAAAATCTGGCACAAAGGCGTTCATGGAAGC

TCTGCAGGCTGGAGCTGACATTTCGATGATTGGTCAGTTTGGTGTTGGTTTCTACTCAGCTTACCTTGTT

GCTGACAAAGTAACTGTTATCTCCAAACACAATGATGACGAGCAGTACATCTGGGAGTCTTCGGCTGGTG

GTTCCTTCACAGTTCGTTCTGACAATGGCGAACCCATTGGCAGAGGAACCAAGATCATCCTGCACATCAA

GGAAGATCAGACTGAGTACTTGGAAGAATCCAAGATCAAGGAGATTGTCAAGAAGCACTCTCAGTTCATT

NNNNNNNNNNNNNNNNNNNNNNNNNNNNNNNNNNNNNNNNNNNNNNNNNNNNNNNNNNNNNNNNNNNNNN

NNNNNNNNNNNNNNNNNNNNNNNNNNNNNNGAATTTTACAAGAGCTTGACCAACGACTGGGAGGACCATC

TTGCTGTCAAACACTTCTCGGTTGAGGGTCAGCTTGAGTTCCGGGCCCTCCTCTTCGTTCCTCGTCGGGC

ACCCTTCGATCTCTTTGAGAACAAGAAGAAGAAGAATAACATCAAGTTGTACGTGAGGAGGGTATTCATC

ATGGACAACTGCGAGGACCTCATCCCAGAGTACCTTAACTTCATCCGAGGTGTGGTGGACAGCGAAGATC

TTCCATTGAACATCTCTCGTGAAATGCTCCAGCAGAACAAGATTCTCAAAGTCATCAGGAAGAATCTTGT

AAAGAAATGCTTGGAACTCTTCGAGGAGCTCGCCGAAGACAAGGAGAACTACAAGAAGTGTTACGAGCAG

TTCAGCAAGAACCTGAAGCTGGGTATCCACGAGGACAGCCAGAACAGGAAAAAGCTGTCGGAGCTTCTGC

GCTACCACACCTCCGCCTCTGGAGATGAGCAGTGCTCGTTGAAGGACTACGTCGGCCGTATGAAGGAGAA

CCAGAAACACATCTACTACATCACCGGCGAGAGCAAAGACCAGGTGGCCAACAGCTCGTTCGTCGAAAGA

GTAAAGAAGCGTGGCTTCGAGGTTGTCTACATGACTGAGCCGATCGACGAGTACGTCGTCCAACAGCTGA

AGGAGTTCGACGGCAAGCAGCTGGTCTCTGTCACGAAGGAAGGCCTCGAGCTGCCCGAGGACGAGGAGGA

GAAGAAGAAGCGCGAGGAGGACAAAGCCAAGTTCGAGAACCTCTGCAAAGTCATGAAGGACATCCTCGAC

AAGAAGGTGGAGAAGGTCGTGGTATCCAACAGGCTGGTCGACTCGCCATGCTGCATCGTCACCTCGCAGT

ACGGCTGGACCGCCAACATGGAAAGGATCATGAAGGCCCAGGCTCTTCGCGACACCTCGACCATGGGCTA

CATGGCTGCAAAGAAACATCTTGAGATCAACCCCGACCACCCGATCATGGAGAACCTCCGACAGAAGGCC

GAGACCGACAAGCATGACAAATCCGTCAAGGACTTGGTCATGCTGCTGTTCGAGACCGCTCTGCTGTCGT

CTGGCTTTGGCCTGGAAGACCCACAGGTCCATGCCGCAAGGATCTACAGGATGATTAAGCTTGGTCTTGG

CTTTGACGATGACGAGATGACCGTGGAAGAAGAGAAGGTCGACAACGAAGTTCCTCCACTAGAAGGCGAC

ACGGAAGAGGCTTCCAGGATGGAGGAAGTCGATTAA

>scaffold1_*Nasonia_giraulti*_631aa

MPEDVVMEQSGEVETFAFQAEIAQLMSLIINTFYSNKEIFIRELISNSSDALDKIRYESLTDPSKLDSCK

ELFIKIIPNKNDRTLTIIDSGIGMTKADLVNNLGTIAKSGTKAFMEALQAGADISMIGQFGVGFYSAYLV

ADKVTVISKHNDDEQYIWESSAGGSFTVRSDNGEPIGRGTKIILHIKEDQTEYLEESKIKEIVKKHSQFI

EFYKSLTNDWEDHLAVKHFSVEGQLEFRALLFVPRRAPFDLFENKKKKNNIKLYVRRVFIMDNCEDLIPE

YLNFIRGVVDSEDLPLNISREMLQQNKILKVIRKNLVKKCLELFEELAEDKENYKKCYEQFSKNLKLGIH

EDSQNRKKLSELLRYHTSASGDEQCSLKDYVGRMKENQKHIYYITGESKDQVANSSFVERVKKRGFEVVY

MTEPIDEYVVQQLKEFDGKQLVSVTKEGLELPEDEEEKKKREEDKAKFENLCKVMKDILDKKVEKVVVSN

RLVDSPCCIVTSQYGWTANMERIMKAQALRDTSTMGYMAAKKHLEINPDHPIMENLRQKAETDKHDKSVK

DLVMLLFETALLSSGFGLEDPQVHAARIYRMIKLGLGFDDDEMTVEEEKVDNEVPPLEGDTEEASRMEEV

D

>scaffold6_*Nassonia_giraulti_*cds_2172bp

ATGGTTGACGCTAAGACTGACAAAATGGAGACTGCAGGAGGAGAGGTTGAGACCTTTGCTTTCCAAGCTG

AAATCGCCCAGTTGATGTCCCTCATCATCAACACTTTCTACTCAAACAAAGAAATCTTCCTCCGAGAATT

GATTTCCAACTCCAGTGATGCTCTGGACAAGATCCGTTACGAATCTTTGACAGACCCCACAAAGTTGGAG

GCTTGCAAGGAACTCTACATCAAGATTATTCCCAACAAGAATGATCGCACACTGACCATCATCGACACTG

GTATTGGTATGACTAAAGCCGATTTGGTTAACAACCTTGGAACCATCGCCAAGTCCGGAACTAAGGCCTT

CATGGAGGCTCTCCAGGCTGGTGCTGACATTTCTATGATTGGTCAGTTTGGTGTTGGTTTCTACTCGGCC

TACCTCGTTGCTGACAAAGTCGTTGTTGTGTCGAAGAACAATGATGACGAGCAGTACATTTGGGAATCCA

GTGCCGGAGGATCTTTCACTGTCAAGGTAGACAATGGTGAGCCTCTTGAACGTGGAACCAAGATCATCCT

CCACATCAAGGAAGACCAATCTGAGTATTTGGAGGAAAGCAAGATCAAGGAAATCGTCAAGAAGCACTCC

CAGTTCATTGGCTACCCCATCAAACTTGTAGTACAAAAGGAGCGTGAGAAGGAACTCAGCGACGACGAAG

CTGAGGCTGAGGAGGAGAAGAAGGAAGAAGATGATGGAAAGCCCAAGGTTGAGGATGTCGGCGAAGACGA

GGAGGAAGACACTGACAAGGAAAAGAAGAAGAAGAAGAAGACCATTAAAGAAAAGTATGAAGAAGATGAG

GAACTCAACAAGACTAAGCCTATTTGGACCAGAAATGCTGACGACATCACCCAGGAAGAGTACGGTGAAT

TTTACAAGTCGTTGACCAACGACTGGGAAGACCACCTGGCTGTAAAGCACTTCTCTGTAGAAGGTCAATT

GGAGTTCAGAGCTCTTCTGTTCGCGCCAAGGCGTATGCCCTTCGATCTCTTTGAGAACAAGAAGAGGAAG

AACAACATCAAGTTGTACGTACGTCGTGTCTTCATCATGGACAACTGCGAAGAGTTGATCCCCGAGTACT

TGAACTTCATGAAGGGAGTCGTCGACAGCGAAGATCTTCCTCTGAACATTTCTCGTGAGATGTTGCAGCA

GAACAAGATCCTTAAGGTCATCAGGAAGAACTTGGTCAAGAAATGTCTGGAACTTTTCGAAGAACTCACC

GAAGACAAAGAGTCCTACAAGAAGTTCTATGAACAATTCAGCAAGAACATCAAGCTTGGAATCCACGAAG

ACAGCGCCAACCGCAGCAAGCTGGCCGATTTGCTCCGTTACCACACGTCCGCTTCCGGTGATGAGGCGTG

CTCCCTCAAGGACTACGTTGGTAGGATGAAGGAAAACCAGAAGCACATCTACTACATCACTGGTGAAAGC

AAGGAGCAGGTAGCCAACAGCTCGTTCGTCGAGCGAGTAAAGAAGCGTGGTTTCGAGGTCGTCTACATGA

CCGAGCCCATCGATGAGTACGTCGTTCAGCAAATGAAAGAATACGATGGAAAGCAGCTGGTCTCTGTCAC

CAAGGAGGGCTTGGAGCTGCCTGAAGATGAAGAAGAGAAGAAGAAGCACGAGGAAGACAAGTCCAAGTTC

GAGAACCTCTGCAAGGTCATGAAGAACATCCTGGACAACAAGGTCGAGAAGGTTCTTGTATCCAACCGTC

TTGTTGACTCTCCATGCTGTATTGTCACCTCTCAGTACGGCTGGACCGCCAACATGGAAAGGATCATGAA

GGCTCAGGCTCTCCGTGATGCTTCCACCATGGGTTACATGGCAGCCAAGAAGCACCTCGAGATCAACCCT

GACCACCCAGTCATCAATACTCTGAGGGAGAAGGCTGAGGCTGACAAGAACGACAAGTCCGTGAAAGACT

TGGTCGTTCTCCTGTTCGAGACTGCCCTCTTGTCGTCTGGTTTCAGTCTGGACGAGCCCCAGGTTCATGC

CGCCCGCATTTACAGAATGGTCAAGCTTGGACTTGGAATCGACGAGGAAGAGCCTGTCCCCGAAGAAACC

AAAGTCGCCGAGGAGGTGCCGCCCCTGGAGGGTGGTGAGGATGATGCGTCGCGCATGGAGGAAGTCGATT

AA

>scaffold6_*Nassonia_giraulti­_*723aa

MVDAKTDKMETAGGEVETFAFQAEIAQLMSLIINTFYSNKEIFLRELISNSSDALDKIRYESLTDPTKLE

ACKELYIKIIPNKNDRTLTIIDTGIGMTKADLVNNLGTIAKSGTKAFMEALQAGADISMIGQFGVGFYSA

YLVADKVVVVSKNNDDEQYIWESSAGGSFTVKVDNGEPLERGTKIILHIKEDQSEYLEESKIKEIVKKHS

QFIGYPIKLVVQKEREKELSDDEAEAEEEKKEEDDGKPKVEDVGEDEEEDTDKEKKKKKKTIKEKYEEDE

ELNKTKPIWTRNADDITQEEYGEFYKSLTNDWEDHLAVKHFSVEGQLEFRALLFAPRRMPFDLFENKKRK

NNIKLYVRRVFIMDNCEELIPEYLNFMKGVVDSEDLPLNISREMLQQNKILKVIRKNLVKKCLELFEELT

EDKESYKKFYEQFSKNIKLGIHEDSANRSKLADLLRYHTSASGDEACSLKDYVGRMKENQKHIYYITGES

KEQVANSSFVERVKKRGFEVVYMTEPIDEYVVQQMKEYDGKQLVSVTKEGLELPEDEEEKKKHEEDKSKF

ENLCKVMKNILDNKVEKVLVSNRLVDSPCCIVTSQYGWTANMERIMKAQALRDASTMGYMAAKKHLEINP

DHPVINTLREKAEADKNDKSVKDLVVLLFETALLSSGFSLDEPQVHAARIYRMVKLGLGIDEEEPVPEET

KVAEEVPPLEGGEDDASRMEEVD

>scaffold1_*Nasonia_longicornis*_cds_>1896bp

ATGCCGGAGGACGTCGCAATGGAACAAAGCGGCGAGGTGGAAACCTTCGCCTTCCAAGCTGAAATCGCCC

AGCTTATGAGCCTGATCATCAACACCTTCTACTCGAACAAGGAGATCTTCATTCGAGAATTGATTTCCAA

CTCGTCTGATGCCTTGGATAAGATTCGCTATGAGTCGCTCACAGATCCATCCAAGCTTGAATCGTGCAAG

GAGCTGTTCATCAAGATCATCCCTAACAAGAACGATCGCACACTCACCATCATTGACTCTGGAATTGGTA

TGACAAAGGCTGACTTGGTGAACAACCTTGGTACCATCGCAAAATCTGGCACAAAGGCGTTCATGGAAGC

TCTGCAGGCTGGAGCTGACATTTCGATGATTGGTCAGTTTGGTGTTGGTTTCTACTCAGCTTACCTTGTT

GCTGACAAAGTAACTGTCATCTCCAAACACAATGATGACGAGCAGTACATCTGGGAGTCTTCGGCTGGTG

GTTCCTTCACAGTTCGTTCTGACAATGGCGAACCCATTGGCAGAGGAACCAAGATCATCCTGCACATCAA

GGAAGATCAGACTGAGTACTTGGAAGAATCCAAGATCAAGGAGATTGTCAAGAAGCACTCTCAGTTCATT

NNNNNNNNNNNNNNNNNNNNNNNNNNNNNNNNNNNNNNNNNNNNNNNNNNNNNNNNNNNNNNNNNNNNNN

NNNNNNNNNNNNNNNNNNNNNNNNNNNNNNGAATTCTACAAGAGCTTGACCAACGACTGGGAGGACCATC

TTGCTGTCAAACACTTCTCGGTTGAGGGTCAGCTTGAGTTCCGGGCCCTCCTCTTCGTTCCTCGTCGGGC

ACCCTTCGATCTCTTTGAGAACAAGAAGAAGAAGAATAACATCAAGTTGTACGTGAGGAGGGTATTCATC

ATGGACAACTGCGAGGACCTCATCCCAGAGTACCTTAACTTCATCCGAGGTGTGGTGGACAGCGAAGATC

TTCCATTGAACATCTCTCGTGAAATGCTCCAGCAGAACAAGATTCTCAAAGTCATCAGGAAGAATCTTGT

AAAGAAATGCTTGGAACTCTTCGAGGAGCTCGCCGAAGACAAGGAGAACTACAAGAAGTGTTACGAGCAG

TTCAGCAAGAACCTGAAGCTGGGTATCCACGAGGACAGCCAGAACAGGAAAAAACTGTCGGAGCTTCTGC

GCTACCACACCTCCGCCTCTGGAGATGAGCAGTGCTCGTTGAAGGACTACGTCGGCCGTATGAAGGAGAA

CCAGAAACACATCTACTACATCACCGGCGAGAGCAAAGACCAGGTGGCCAACAGCTCGTTCGTCGAAAGA

GTAAAGAAGCGTGGCTTCGAGGTTGTCTACATGACTGAGCCGATCGACGAGTACGTCGTCCAACAGCTGA

AGGAGTTCGACGGCAAGCAGCTGGTTTCTGTCACGAAGGAAGGCCTCGAGCTGCCCGAGGACGAGGAGGA

GAAGAAGAAGCGCGAGGAGGACAAGGCCAAGTTCGAGAACCTCTGCAAAGTCATGAAGGACATCCTCGAC

AAGAAGGTGGAGAAGGTCGTGGTATCCAATAGGCTGGTCGACTCGCCATGCTGCATCGTCACCTCGCAGT

ACGGCTGGACCGCCAACATGGAAAGGATCATGAAGGCCCAGGCTCTTCGCGACACCTCGACCATGGGCTA

CATGGCTGCAAAGAAACATCTTGAGATCAACCCCGACCACCCGATCATGGAGAACCTCCGACAGAAGGCC

GAGACCGACAAGCATGACAAATCCGTCAAGGACTTGGTCATGCTGCTGTTCGAGACCGCCCTGCTGTCGT

CTGGCTTTGGCCTGGAAGACCCACAGGTCCATGCCGCAAGGATCTACAGGATGATTAAGCTTGGTCTTGG

CTTTGACGATGACGAGATGACCGTGGAAGAAGAGAAGGTCGACAACGAAGTTCCTCCACTAGAAGGCGAC

ACGGAAGAGGCTTCCAGGATGGAGGAAGTCGATTAA

>scaffold1_*Nasonia_longicornis*_631aa

MPEDVAMEQSGEVETFAFQAEIAQLMSLIINTFYSNKEIFIRELISNSSDALDKIRYESLTDPSKLESCK

ELFIKIIPNKNDRTLTIIDSGIGMTKADLVNNLGTIAKSGTKAFMEALQAGADISMIGQFGVGFYSAYLV

ADKVTVISKHNDDEQYIWESSAGGSFTVRSDNGEPIGRGTKIILHIKEDQTEYLEESKIKEIVKKHSQFI

EFYKSLTNDWEDHLAVKHFSVEGQLEFRALLFVPRRAPFDLFENKKKKNNIKLYVRRVFIMDNCEDLIPE

YLNFIRGVVDSEDLPLNISREMLQQNKILKVIRKNLVKKCLELFEELAEDKENYKKCYEQFSKNLKLGIH

EDSQNRKKLSELLRYHTSASGDEQCSLKDYVGRMKENQKHIYYITGESKDQVANSSFVERVKKRGFEVVY

MTEPIDEYVVQQLKEFDGKQLVSVTKEGLELPEDEEEKKKREEDKAKFENLCKVMKDILDKKVEKVVVSN

RLVDSPCCIVTSQYGWTANMERIMKAQALRDTSTMGYMAAKKHLEINPDHPIMENLRQKAETDKHDKSVK

DLVMLLFETALLSSGFGLEDPQVHAARIYRMIKLGLGFDDDEMTVEEEKVDNEVPPLEGDTEEASRMEEV

D

>scaffold6_*Nasonia_longicornis*_cds_2172bp

ATGGTTGACGCTAAGACTGACAAAATGGAGACTGCAGGAGGAGAGGTTGAGACCTTTGCTTTCCAAGCTG

AAATCGCCCAGTTGATGTCCCTCATCATCAACACTTTCTACTCGAACAAAGAAATCTTCCTCCGAGAATT

GATTTCCAACTCCAGTGATGCCCTGGACAAGATCCGTTACGAATCTTTGACAGACCCCACAAAGTTGGAG

GCTTGCAAGGAACTCTACATCAAGATTATTCCCAACAAGAATGATCGCACACTGACCATCATCGACACTG

GTATTGGTATGACTAAAGCCGATTTGGTTAACAACCTTGGAACCATCGCCAAGTCCGGAACTAAGGCCTT

CATGGAGGCTCTCCAGGCTGGTGCTGACATTTCTATGATTGGTCAGTTTGGTGTTGGTTTCTACTCGGCC

TACCTCGTTGCTGACAAAGTCGTTGTTGTGTCGAAGAACAATGATGACGAGCAGTACATTTGGGAATCCA

GCGCCGGAGGATCTTTCACTGTCAAGGTAGACAATGGTGAGCCTCTTGAACGTGGAACCAAGATCATCCT

CCACATCAAGGAAGACCAATCTGAGTATTTGGAGGAAAGCAAGATCAAGGAAATCGTAAAGAAGCACTCC

CAGTTCATTGGCTACCCCATCAAACTTGTAGTACAAAAGGAGCGTGAAAAGGAACTCAGCGACGACGAAG

CTGAGGCTGAGGAGGAGAAGAAGGAAGAAGATGATGGAAAGCCCAAGGTTGAGGATGTCGGCGAAGACGA

GGAGGAAGACACTGACAAGGAAAAGAAGAAGAAGAAGAAGACCATTAAAGAAAAGTATGAAGAAGATGAG

GAACTCAACAAGACTAAGCCTATTTGGACCAGAAATGCTGACGACATCACCCAGGAAGAGTACGGTGAAT

TTTACAAGTCGTTGACCAACGACTGGGAAGACCACCTGGCTGTAAAGCACTTCTCTGTAGAAGGTCAATT

GGAGTTCAGAGCTCTTCTGTTCGCGCCAAGGCGTATGCCCTTCGATCTCTTTGAGAACAAGAAGAGGAAG

AACAACATCAAGTTGTACGTACGTCGTGTCTTCATCATGGATAACTGCGAAGAGTTGATCCCCGAGTACT

TGAACTTCATGAAGGGAGTCGTCGACAGCGAAGATCTTCCTCTGAACATTTCTCGTGAGATGTTGCAGCA

GAACAAGATCCTTAAGGTCATCAGGAAGAACTTGGTCAAGAAATGTCTGGAACTTTTCGAAGAACTCACC

GAAGACAAAGAGTCCTACAAGAAGTTCTATGAACAATTCAGCAAGAACATCAAGCTTGGAATCCACGAAG

ACAGCGCCAACCGCAGCAAGCTGGCCGATTTGCTCCGTTACCACACGTCCGCTTCCGGTGATGAGGCGTG

CTCCCTCAAGGACTACGTTGGTAGGATGAAGGAAAACCAGAAGCACATCTACTACATCACTGGTGAAAGC

AAGGAGCAGGTAGCCAACAGCTCGTTCGTCGAGCGAGTAAAGAAGCGTGGTTTCGAGGTCGTCTACATGA

CCGAGCCCATCGATGAGTACGTCGTTCAGCAAATGAAAGAATACGATGGAAAGCAGCTGGTCTCTGTCAC

CAAGGAGGGCTTGGAGCTGCCTGAAGACGAAGAAGAGAAGAAGAAGCACGAGGAAGACAAGTCCAAGTTC

GAGAACCTCTGCAAGGTCATGAAGAACATCCTGGACAACAAGGTCGAGAAGGTTCTTGTATCCAACCGTC

TTGTTGACTCTCCATGCTGTATTGTCACCTCTCAGTACGGCTGGACCGCCAACATGGAAAGGATCATGAA

GGCTCAGGCTCTCCGTGATGCTTCCACCATGGGTTACATGGCAGCCAAGAAGCACCTCGAGATCAACCCT

GACCACCCAGTCATCAATACTCTGAGGGAGAAGGCTGAGGCTGACAAGAACGACAAGTCCGTGAAAGACT

TGGTCGTTCTCCTGTTCGAGACTGCCCTCTTGTCGTCTGGTTTCAGTCTGGACGAGCCCCAGGTTCATGC

CGCCCGCATTTACAGAATGGTCAAGCTTGGACTTGGAATCGACGAGGAAGAGCCTGTCCCCGAAGAAACC

AAAGTCGCCGAGGAGGTGCCGCCCCTGGAGGGTGGTGAGGATGATGCGTCGCGCATGGAGGAAGTCGATT

AA

>scaffold6_*Nasonia_longicornis*_723aa

MVDAKTDKMETAGGEVETFAFQAEIAQLMSLIINTFYSNKEIFLRELISNSSDALDKIRYESLTDPTKLE

ACKELYIKIIPNKNDRTLTIIDTGIGMTKADLVNNLGTIAKSGTKAFMEALQAGADISMIGQFGVGFYSA

YLVADKVVVVSKNNDDEQYIWESSAGGSFTVKVDNGEPLERGTKIILHIKEDQSEYLEESKIKEIVKKHS

QFIGYPIKLVVQKEREKELSDDEAEAEEEKKEEDDGKPKVEDVGEDEEEDTDKEKKKKKKTIKEKYEEDE

ELNKTKPIWTRNADDITQEEYGEFYKSLTNDWEDHLAVKHFSVEGQLEFRALLFAPRRMPFDLFENKKRK

NNIKLYVRRVFIMDNCEELIPEYLNFMKGVVDSEDLPLNISREMLQQNKILKVIRKNLVKKCLELFEELT

EDKESYKKFYEQFSKNIKLGIHEDSANRSKLADLLRYHTSASGDEACSLKDYVGRMKENQKHIYYITGES

KEQVANSSFVERVKKRGFEVVYMTEPIDEYVVQQMKEYDGKQLVSVTKEGLELPEDEEEKKKHEEDKSKF

ENLCKVMKNILDNKVEKVLVSNRLVDSPCCIVTSQYGWTANMERIMKAQALRDASTMGYMAAKKHLEINP

DHPVINTLREKAEADKNDKSVKDLVVLLFETALLSSGFSLDEPQVHAARIYRMVKLGLGIDEEEPVPEET

KVAEEVPPLEGGEDDASRMEEVD

>scf7180000350230_*Pogonomyrmex_barbatus*_cds_2169_bp

ATGCCCGAAGACGTGAGCATGACGGATGCCGGCGAGGTGGAGACCTTCGCCTTCCAGGCCGAGATCGCCC

AGTTGATGAGTTTGATCATCAATACCTTTTACTCGAACAAGGAAATCTTCATCCGAGAATTGATTTCCAA

CTCGTCCGATGCGTTGGACAAGATCCGCTATGAATCACTTACGGATCCATCAAAGCTGGATACCTGTAAG

GAACTATTCATTAAAATTATTCCGAACAAGAACGATCGTACCCTGACAATTCTTGATTCTGGCATTGGCA

TGACTAAGGCTGATCTTGTAAATAACCTGGGTACAATCGCCAAGTCAGGCACAAAGGCATTCATGGAGGC

CCTGCAAGCAGGCGCTGATATTTCCATGATTGGACAGTTTGGTGTAGGTTTCTACTCGGCGTACCTCGTA

GCTGATAAAGTCATCGTAATCTCCAAGCATAACGATGACGAGCAGTACTTGTGGGAGTCTTCTGCTGGTG

GTTCGTTCACCGTTCGTCCTGATAACGGCGAACCTATTGGACGAGGCACTAAGATCATCCTGCACATCAA

GGAAGATCAGACGGAATACCTCGAGGAGTCGAAGATCAAAGAAATCGTCAAGAAGCATTCTCAGTTCATT

GGCTATCCCATTAAACTTGTCGTCGAGAAGGAACGAGACAAGGAACTGAGTGAGGACGAGGAGGAGGAGC

CCGCAAAGGAGGGTGAGACAGAGGATGAGAAGCCCAAGATTGAGGATGTCGGCGAGGATGAGGAAGAAGA

TAAACCTAAGGATGAGAAGAAAAAGAAGAAGAAGACCATCAAAGAAAAATACACCGAAGACGAGGAGTTG

AACAAGACGAAACCGATCTGGACGAGAAATCCGGATGACATTACGCAGGAGGAGTATGGCGAGTTCTACA

AGAGCTTGACCAACGACTGGGAAGACCATCTGGCTGTGAAGCATTTCTCGGTTGAAGGACAGCTTGAATT

CAGAGCTCTTTTGTTTATCCCACGTCGCGCGCCCTTCGACTTGTTTGAGAACAAGAAGAGGAAGAACAAC

ATTAAACTGTATGTGCGTCGCGTCTTTATTATGGACAATTGCGAGGACTTGATCCCAGAATACCTGAACT

TCATCAAGGGCGTCGTAGACAGCGAAGATTTACCCCTGAACATTTCTCGTGAGATGCTGCAACAAAATAA

GATTCTAAAGGTTATCAGAAAGAATCTTGTCAAGAAATGCCTGGAACTCTTCGAGGAATTGTCAGAAGAC

AAAGAGAATTACAAGAAGTGCTACGAGCAATTCAGCAAGAATTTGAAACTGGGTATCCACGAAGATAGCC

AGAATAGAAAGAAGCTGTCGGAGCTGTTGCGTTATCACACTTCTGCATCCGGCGACGAGATGTGTTCGCT

CAAGGATTATGTTGGTAGAATGAAGGAGAACCAGAAGCACATTTACTACATCACTGGCGAGAGCAGGGAG

CAGGTAGCCAACAGTTCGTTTGTGGAGCGCGTGAAGAAGCGTGGCTTCGAGGTCGTCTACATGACGGAGC

CCATCGATGAGTATGTCGTTCAGCAATTGAAGGAATTCGATGGAAAGCAGTTGGTGTCTGTCACAAAGGA

GGGTCTGGAATTGCCAGAGGATGAGGAGGAGAAGAAGAAGCGCGAGGAGGATAAAGCCAAATTTGAAAAT

CTTTGCAAAGTTATGAAGGATATCTTGGATAAGAAGGTAGAGAAGGTGGTAGTATCCAACAGGTTGGTCG

ATTCTCCTTGCTGCATCGTCACGTCGCAGTACGGCTGGACGGCAAACATGGAGAGAATCATGAAGGCACA

GGCACTCCGTGACACGTCTACCATGGGATATATGGCTGCGAAAAAACATCTCGAGATCAACCCCGATCAT

CCTATCATGGAGAATCTGAGGCAAAAGGCTGAGGCCGATAAGCACGACAAGTCTGTCAAAGATCTAGTCA

TGTTGTTGTTCGAGACTGCTCTCTTGTCGTCCGGCTTCGCGCTCGAGGACCCGCAGGTGCATGCATCTAG

AATATACAGAATGATCAAGCTCGGTCTGGGCTTTGACGACGAAGATACGTCGAACGCTGAAGACGAGAAG

ATGGATATGGAAGTGCCCACGTTGGAGGGTGACACGGAAGAGGCGTCGAGGATGGAGGAAGTAGATTAA

>scf7180000350230_*Pogonomyrmex_barbatus*_722aa

MPEDVSMTDAGEVETFAFQAEIAQLMSLIINTFYSNKEIFIRELISNSSDALDKIRYESLTDPSKLDTCK

ELFIKIIPNKNDRTLTILDSGIGMTKADLVNNLGTIAKSGTKAFMEALQAGADISMIGQFGVGFYSAYLV

ADKVIVISKHNDDEQYLWESSAGGSFTVRPDNGEPIGRGTKIILHIKEDQTEYLEESKIKEIVKKHSQFI

GYPIKLVVEKERDKELSEDEEEEPAKEGETEDEKPKIEDVGEDEEEDKPKDEKKKKKKTIKEKYTEDEEL

NKTKPIWTRNPDDITQEEYGEFYKSLTNDWEDHLAVKHFSVEGQLEFRALLFIPRRAPFDLFENKKRKNN

IKLYVRRVFIMDNCEDLIPEYLNFIKGVVDSEDLPLNISREMLQQNKILKVIRKNLVKKCLELFEELSED

KENYKKCYEQFSKNLKLGIHEDSQNRKKLSELLRYHTSASGDEMCSLKDYVGRMKENQKHIYYITGESRE

QVANSSFVERVKKRGFEVVYMTEPIDEYVVQQLKEFDGKQLVSVTKEGLELPEDEEEKKKREEDKAKFEN

LCKVMKDILDKKVEKVVVSNRLVDSPCCIVTSQYGWTANMERIMKAQALRDTSTMGYMAAKKHLEINPDH

PIMENLRQKAEADKHDKSVKDLVMLLFETALLSSGFALEDPQVHASRIYRMIKLGLGFDDEDTSNAEDEK

MDMEVPTLEGDTEEASRMEEVD

>supercontigGL563022_*Rhodnius_prolixus*_cds_2184bp

ATGCCAGAAGACGTAGAAATGGGCGAAGTTGAGACTTTTGCCTTTCAGGCAGAAATTGCCCAACTTATGT

CTTTGATCATCAATACGTTCTACTCAAATAAGGAAATATTTCTAAGAGAATTAATCTCAAATTCATCTGA

TGCTTTGGATAAAATAAGGTACGAGTCTTTGACGGACCCATCAAGACTGGAGAATGGGAAAGAATTATAC

ATTAAGATCATACCTAACAAAAATGATAGGACTCTTACAATCATTGATACAGGCATCGGCATGACAAAAG

CAGATTTGGTGAACAATCTGGGAACCATTGCTAAATCCGGAACAAAAGCATTTATGGAAGCTTTGCAGGC

TGGAGCTGATATATCCATGATTGGACAGTTTGGTGTCGGTTTCTATTCAGCTTATTTGGTAGCTGATAAA

GTCACAGTAAGCTCTAAACATAACGATGATGAACAATATCTTTGGGAGTCTTCAGCTGGAGGATCATTCA

CCGTTCGACCAGATCCTGGTGAACCACTTGGTAGAGGCACAAAAATTGTACTGTACGTTAAAGAAGATCA

AACAGAATATCTAGAAGAAAGAAAAATTAAGGAAGTAGTCAAGAAACATTCTCAGTTCATTGGATACCCA

ATCAAGTTACTAGTTGAAAAAGAAAGAAATAAGGAAATCAGCGATGATGAAGCTGAGGAAGACAAAGATG

ATAAAGGCGAAGGAGATACCGACAAAGAGAAAGCTGAAGATGACCAGCCTAAAATTGAAGATGTTGGTGA

AGATGAAGAAGAAGATGATAAAGAGGGCAAAGATAAAAAAAAGAAAAAGACGGTGGTGGAAAAATATACT

GAAGACGAAGAATTGAACAAGACCAAACCAATTTGGACTAGAAATCCTGATGACATTTCACAAGATGAAT

ATGGAGAATTTTACAAATCTTTAACCAATGATTGGGAAGATCACTTAGCTGTTAAGCACTTCTCTGTAGA

AGGACAATTAGAATTCCGTGCACTTCTGTTTGTACCTAGGCGAGCTCCATTTGATTTATTTGAAAATAAG

AAGCGTAAGAACAATATAAAATTGTACGTCAGGAGAGTGTTTATTATGGATAATTGTGAAGATCTTATTC

CAGAATATCTCAACTTCATCAAAGGAGTTGTTGATAGTGAGGATTTACCACTAAATATCTCCAGAGAGAT

GCTGCAGCAAAATAAAATACTCAAAGTAATCCGTAAAAATCTTGTGAAGAAATGTCTAGAATTATTTGAT

GAACTTGCTGAAGATAAAGATAACTACAAGAAGTTTTATGAACAATTTAGTAAAAACCTCAAACTTGGAA

TTCATGAAGACTCTCAGAACCGAAAGAAACTATCCGAATTATTAAGGTATTACACTTCAGCATCTGGCGA

TGAAGCTTGTTCATTGAAAGATTACGTTGCTCGAATGAAGGAAAATCAGAAACATATCTATTTCATTACT

GGTGAAAACAAAGAGCAAGTAGCAAATTCATCATTTGTCGAAAGAGTTAAGAAACGAGGATTTGAAGTTG

TTTATATGACAGAACCAATTGATGAATATGTAGTTCAGCAAATGAAAGAGTTTGACGGTAAACAGTTAAT

CTCGGTTACCAAAGAAGGTCTTGAACTACCAGAAGATGAAGAAGAAAAGAAGAAGAGAGAAGAAGATAAA

GCAAGATTTGAAGGATTGTGTAAAGTAATGAAGGATATATTGGACAAAAAGGTAGAAAAAGTTGCTGTGA

GTAACAGACTAGTTGATTCCCCATGTTGTATAGTTACTTCACAGTATGGTTGGACAGCAAACATGGAGAG

AATAATGAAAGCTCAAGCTCTGAGAGATACTTCCACAATGGGTTATATGGCTGCTAAGAAACATCTAGAA

ATAAATCCAGATCATCCTATCATTGAAACTCTTAGACAGAAAGCTGAAGCAGATAAAAATGATAAGGCTG

TAAAAGATTTAGTGATTCTACTTTTCGAAACATCTCTATTATCTTCTGGATTTGCGCTAGAAGAACCGCA

AGTACATGCTTCCCGTATTTATAGGATGATTAAACTTGGACTTGGTATTGAAGATGATGATGTGGCAGGA

ACTGAGGAACAGCCAGTAGTAGAAGATATGCAAACCGTTGAGGGAGGAACGGAAGATGCTTCGAGAATGG

AAGAAGTAGATTAA

>supercontigGL563022_*Rhodnius_prolixus*_727aa

MPEDVEMGEVETFAFQAEIAQLMSLIINTFYSNKEIFLRELISNSSDALDKIRYESLTDPSRLENGKELY

IKIIPNKNDRTLTIIDTGIGMTKADLVNNLGTIAKSGTKAFMEALQAGADISMIGQFGVGFYSAYLVADK

VTVSSKHNDDEQYLWESSAGGSFTVRPDPGEPLGRGTKIVLYVKEDQTEYLEERKIKEVVKKHSQFIGYP

IKLLVEKERNKEISDDEAEEDKDDKGEGDTDKEKAEDDQPKIEDVGEDEEEDDKEGKDKKKKKTVVEKYT

EDEELNKTKPIWTRNPDDISQDEYGEFYKSLTNDWEDHLAVKHFSVEGQLEFRALLFVPRRAPFDLFENK

KRKNNIKLYVRRVFIMDNCEDLIPEYLNFIKGVVDSEDLPLNISREMLQQNKILKVIRKNLVKKCLELFD

ELAEDKDNYKKFYEQFSKNLKLGIHEDSQNRKKLSELLRYYTSASGDEACSLKDYVARMKENQKHIYFIT

GENKEQVANSSFVERVKKRGFEVVYMTEPIDEYVVQQMKEFDGKQLISVTKEGLELPEDEEEKKKREEDK

ARFEGLCKVMKDILDKKVEKVAVSNRLVDSPCCIVTSQYGWTANMERIMKAQALRDTSTMGYMAAKKHLE

INPDHPIIETLRQKAEADKNDKAVKDLVILLFETSLLSSGFALEEPQVHASRIYRMIKLGLGIEDDDVAG

TEEQPVVEDMQTVEGGTEDASRMEEVD

>supercontigGL563086_*Rhodnius_prolixus­*_cds_2172bp

ATGCCTGAAGACGTTGAAATGAATGGAGAAGTGGAGACATTTGCCTTTCAGGCAGAAATTGCCCAGCTTA

TGTCTCTTATTATTAACACTTTTTATTCTAATAAAGAAATTTTTCTCCGAGAACTTATTTCTAATTCATC

TGATGCATTGGATAAAATACGATATGAGTCACTAACTGATGCCTCAAGATTAGAAAGTGGGAAAGAATTA

TTTATTAAGATAGTTCCAAACAAGGATGACCGGACACTTACCATTATTGATTCTGGTATTGGTATGACAA

AGGCAGATCTTGTTAACAATTTGGGAACCATCGCTAAATCCGGAACAAAGGCGTTTATGGAAGCTCTGCA

AGCAGGAGCCGATATATCAATGATTGGGCAGTTTGGTGTTGGTTTCTACTCTGCCTATTTAGTAGCCGAC

AAAGTAACCGTCAGCTCCAAACATAATGACGACGAGCAGTACTTGTGGGAGTCTTCAGCAGGAGGATCAT

TCACAATTCGCCCTGACCCCGGCGAGCCGCTTGGTAGAGGAACTAAAATTGTTCTTCACATTAAAGAAGA

TCAAACTGAATATCTCGAAGAACGAAAAATAAAGGAAGTTGTAAAGAAGCATTCACAGTTTATTGGATAT

CCCATTAAACTTCTAGTCGAAAAGGAAAGAGACAAAGAATTAAGCGAGGATGAAGAAGAGGAAGAAAAGA

AGGAAGGAGAAGAAGAAAAAAAGGATGAAGATAAACCGAAAATAGAGGACGTTGGTGAAGATGAGGAAGA

AGATGCCGATAAAGAAAAGAAAAAGAAGAAGAAAACAGTTAAAGAAAAATATACTGAAGATGAGGAATTA

AATAAAACAAAACCAATTTGGACAAGAAACCCTGATGATATATCTCAGGATGAATACGGAGAGTTTTATA

AATCTCTAACAAACGACTGGGAGGATCATCTAGCTGTTAAACATTTTTCGGTTGAAGGACAGTTAGAGTT

CCGGGCTTTACTTTTTGTGCCTAGAAGAGCTCCATTCGATCTCTTTGAAAACAAAAAGAGAAAGAACAAT

ATAAAATTATACGTCAGAAGGGTTTTCATAATGGACAACTGTGAAGATTTAATACCAGAGTACCTTAATT

TCATCAAAGGTGTGGTTGATAGTGAAGATTTACCATTGAATATTTCTAGAGAAATGTTACAACAAAACAA

AATTTTAAAAGTCATTAGGAAGAATCTCGTGAAGAAATGTTTAGAACTTTTTGATGAACTAGCCGAGGAT

AAAGACAATTACAACAAATTTTATGCTCAGTTTGGTAAAAACTTGAAGTTAGGAATCCATGAAGATTCAC

AAAACAGGAAAAAGTTGTCTGAACTACTGCGGTATCATACTTCTGCTTCTGGTGATGAAGCTTGTCCACT

TAAAGATTATGTTGCGAGAATGAAAGATAATCAGAAACATATTTATTACATTACCGGAGAGAACAAAGAA

CAAGTTGCGAATTCATCATTTGTAGAAAGAGTTAAGAAACGTGGTTTCGAAGTCGTCTACATGACTGAAC

CAATAGATGAATATGTAGTACAACAAATGAAAGAATTTGATGGAAAACAACTCGTGTCTGTCACAAAGGA

AGGATTGGAATTGCCAGAAGACGAAGAGGAGAAAAAGAAAAGGGAAGAAGATAAAGCTAAGTTCGAGAGT

CTATGTAAAGTTATGAAAGATATCCTGGACAAGAAAGTTGAAAAAGTGGTGGTAAGCAACAGACTGGTTG

ATTCACCTTGTTGTATTGTTACCTCGCAGTATGGTTGGACTGCAAATATGGAGAGGATAATGAAAGCACA

GGCTTTAAGAGATACGTCTACAATGGGTTATATGGCGGCCAAAAAACACCTCGAAATTAACCCCGATCAT

CCTATTGTTGATACTTTGCGACAGAAAGCAGAAGCAGACAAGAATGATAAGGCCGTCAAAGATCTAGTCA

TCCTTCTTTTCGAAACTTCTCTCCTTTCATCCGGATTCGGTCTTGATGATCCTCAGTTGCACGCTTCCAG

AATTTATCGCATGATTAAGCTCGGTCTTGGTATAGATGATGACCTAACGCCCTCTTCTGATGAAACCGAA

ACAGCAGATGCCGAAATGCCACCTCTTGAAGCTAGTACGGAAGACACTTCAAGGATGGAAGAAGTAGATT

AA

>supercontigGL563086_*Rhodnius_prolixus­*_723aa

MPEDVEMNGEVETFAFQAEIAQLMSLIINTFYSNKEIFLRELISNSSDALDKIRYESLTDASRLESGKEL

FIKIVPNKDDRTLTIIDSGIGMTKADLVNNLGTIAKSGTKAFMEALQAGADISMIGQFGVGFYSAYLVAD

KVTVSSKHNDDEQYLWESSAGGSFTIRPDPGEPLGRGTKIVLHIKEDQTEYLEERKIKEVVKKHSQFIGY

PIKLLVEKERDKELSEDEEEEEKKEGEEEKKDEDKPKIEDVGEDEEEDADKEKKKKKKTVKEKYTEDEEL

NKTKPIWTRNPDDISQDEYGEFYKSLTNDWEDHLAVKHFSVEGQLEFRALLFVPRRAPFDLFENKKRKNN

IKLYVRRVFIMDNCEDLIPEYLNFIKGVVDSEDLPLNISREMLQQNKILKVIRKNLVKKCLELFDELAED

KDNYNKFYAQFGKNLKLGIHEDSQNRKKLSELLRYHTSASGDEACPLKDYVARMKDNQKHIYYITGENKE

QVANSSFVERVKKRGFEVVYMTEPIDEYVVQQMKEFDGKQLVSVTKEGLELPEDEEEKKKREEDKAKFES

LCKVMKDILDKKVEKVVVSNRLVDSPCCIVTSQYGWTANMERIMKAQALRDTSTMGYMAAKKHLEINPDH

PIVDTLRQKAEADKNDKAVKDLVILLFETSLLSSGFGLDDPQLHASRIYRMIKLGLGIDDDLTPSSDETE

TADAEMPPLEASTEDTSRMEEVD

>scaffold08068_*Solenopsis_invicta*_cds_2172_bp

ATGCCCGAAGACGTGAGCATGGCGGATTCCGGCGAGGTGGAGACCTTCGCCTTCCAGGCCGAGATCGCTC

AGTTGATGAGCTTGATCATCAACACTTTCTACTCGAACAAAGAAATTTTCATCCGAGAATTGATCTCCAA

TTCATCCGATGCATTGGACAAAATTCGATATGAATCTCTTACGGAGCCAAGCAAGTTGGACACTTGCAAG

GAGTTGTTCATTAAGATTGTCCCAAACAAGAACGACCGCACCTTGACTATTCTCGATTCTGGCATTGGCA

TGACCAAAGCTGATCTTGTAAATAACCTGGGTACCATTGCCAAGTCTGGTACGAAGGCATTCATGGAAGC

TCTGCAGGCAGGCGCTGATATCTCCATGATTGGACAGTTTGGTGTGGGTTTCTACTCTGCATATCTTGTA

GCTGATAAAGTCAGTGTTATCTCCAAGCACAATGACGATGAGCAGTACCTGTGGGAGTCTTCCGCTGGCG

GTTCATTCACAGTTCGCCCCGACGATGGCGAACCGATTGGACGAGGTACTAAGATCATCCTGCACATTAA

GGAGGATCAGACGGAATATCTGGAGGAGTCAAAAATTAAAGAAATTGTGAAGAAGCACTCTCAGTTTATT

GGCTATCCCATCAAGCTCGTCGTCGAGAAGGAGCGCGACAAGGAGCTCAGCGAGGATGAGGAGGAGGAAC

CTGCGAAGGAGGGAGAGAAACCAGAGGATGAGAAACCAAAAATTGAGGATGTAGGCGAAGATGAAGATGA

AGATAAGCCCAAGGATGAAAAGAAAAAGAAGAAGAAGACCATCAAAGAGAAATATACTGAAGATGAGGAG

TTGAACAAGACAAAACCGATTTGGACGAGAAATCCAGATGATATCACACAAGAGGAGTACGGCGAATTCT

ACAAGAGCTTGACCAATGATTGGGAAGATCATTTGGCTGTGAAACATTTCTCTGTGGAGGGACAGTTGGA

ATTCAGAGCACTGCTATTCATCCCGCGTCGCGCGCCTTTCGACTTGTTCGAAAATAAGAAGAGGAAGAAT

AACATAAAATTATACGTGCGCCGTGTCTTCATTATGGATAACTGCGAAGATCTGATTCCAGAATATCTAA

ACTTCATCAAGGGCGTCGTGGACAGCGAAGATCTGCCTCTCAACATCTCTCGTGAGATGCTGCAACAGAA

TAAGATTCTCAAAGTCATCAGGAAGAATCTTGTCAAGAAGTGTCTGGAGCTCTTCGAGGAACTGTCGGAA

GACAAAGAGAACTACAAGAAGTGCTATGAGCAATTCAGCAAGAATCTGAAATTGGGTATCCACGAGGACA

GCCAGAACAGAAAGAAACTTTCAGAACTACTGCGATACCACACTTCCGCATCTGGAGATGAGATGTGTTC

ACTCAAGGACTATGTTGGTAGAATGAAGGAGAGCCAGAAACATGTCTACTATATCACTGGCGAAAGCAGG

GAGCAAGTAGCCAACAGTTCGTTTGTAGAGCGCGTGAAGAAGCGCGGTTTCGAGGTTGTATACATGACAG

AGCCCATCGATGAATATGTCGTCCAGCAGCTGAAGGAATTCGACGGCAAGCAGCTAGTGTCTGTCACGAA

AGAAGGTCTGGAATTGCCAGAGGATGAGGACGAGAAGAAGAAACGCGAGGAGGACAAAGCCAAATTTGAA

AATCTCTGCAAAGTCATGAAAGATATCTTAGACAAGAAGGTGGAGAAAGTCGTGGTATCCAACAGGCTGG

TCGACTCTCCTTGCTGTATCGTCACGTCACAGTATGGCTGGACGGCGAACATGGAGAGAATCATGAAGGC

TCAGGCTCTTCGTGACACATCGACCATGGGATACATGGCTGCAAAGAAGCATCTTGAAATCAATCCTGAT

CATCCCATCATGGAGAACTTGAGACAAAAGGCTGAGGCAGACAAACACGACAAATCTGTCAAGGATCTAG

TCATGCTTCTCTTCGAGACTGCCCTCTTGTCGTCCGGTTTTGCGCTAGAGGACCCACAGGTACATGCGTC

TAGAATCTACAGAATGATCAAGCTCGGCCTGGGTTTCGACGACGAGGAAACGTCGAATCCTGAGGACGAA

AAGATGGACATGGAAGTACCGACGTTGGACGGTGATTCGGAAGAGGCGTCAAGAATGGAGGAAGTAGATT

AA

>scaffold08068_*Solenopsis_invicta*_723aa

MPEDVSMADSGEVETFAFQAEIAQLMSLIINTFYSNKEIFIRELISNSSDALDKIRYESLTEPSKLDTCK

ELFIKIVPNKNDRTLTILDSGIGMTKADLVNNLGTIAKSGTKAFMEALQAGADISMIGQFGVGFYSAYLV

ADKVSVISKHNDDEQYLWESSAGGSFTVRPDDGEPIGRGTKIILHIKEDQTEYLEESKIKEIVKKHSQFI

GYPIKLVVEKERDKELSEDEEEEPAKEGEKPEDEKPKIEDVGEDEDEDKPKDEKKKKKKTIKEKYTEDEE

LNKTKPIWTRNPDDITQEEYGEFYKSLTNDWEDHLAVKHFSVEGQLEFRALLFIPRRAPFDLFENKKRKN

NIKLYVRRVFIMDNCEDLIPEYLNFIKGVVDSEDLPLNISREMLQQNKILKVIRKNLVKKCLELFEELSE

DKENYKKCYEQFSKNLKLGIHEDSQNRKKLSELLRYHTSASGDEMCSLKDYVGRMKESQKHVYYITGESR

EQVANSSFVERVKKRGFEVVYMTEPIDEYVVQQLKEFDGKQLVSVTKEGLELPEDEDEKKKREEDKAKFE

NLCKVMKDILDKKVEKVVVSNRLVDSPCCIVTSQYGWTANMERIMKAQALRDTSTMGYMAAKKHLEINPD

HPIMENLRQKAEADKHDKSVKDLVMLLFETALLSSGFALEDPQVHASRIYRMIKLGLGFDDEETSNPEDE

KMDMEVPTLDGDSEEASRMEEVD

**MOLLUSCA**

>cont2.16199_*Aplysia_californica*_cds_2190bp

ATGCCTGAAATTCAACCTGCTGAAGAGATGGAGGGGGAGAAGGAAACTTTTGCCTTTCAAGCTGAGATTG

CACAGTTGATGAGCTTGATCATCAACACATTCTACAGCAACAAGGAAATCTTCTTGAGAGAGTTGATCTC

AAATGCTTCTGATGCCTTAGACAAAATCAGATATGAATCTCTGACAGATCCAAGCAGATTAGACTCTGGA

AAGGATTTGCAAATCAGAATCGTACCTGACAAGGAGAACAAGACCCTCATCATTGAGGACTCTGGAATTG

GCATGACAAAGGCTGACTTGGTGAACAACCTAGGAACCATTGCCAAATCAGGTACGAAGGCTTTCATGGA

AGCCCTGCAAGCTGGGGCTGACATTTCTATGATTGGACAGTTTGGTGTAGGTTTCTACTCTGCTTACCTG

ATAGCTGACCGTGTTGTGGTGGAGTCCAAACACAACGATGATGAGCTGTACACATGGGAGTCTTCAGCTG

GTGGTTCCTTCACAATAGCCTCTGGCACTGGAGCCCCGCTGGCTCGTGGTACAAGAATCACTCTGTATGT

CAAGGAAGATCAGGTGGAATATTTGGAAGAGAAAAGAATTAAAGATGTTATCAAGAAACACAGCCAGTTC

ATTGGTTATCCCATCAAACTGTTGGTGGAGAAGGAACGGGAGAAGGAAGTTTCTGATGATGAAGAGGAGG

AAGAAAAAAAAGAGGATGACAAGGAAGAGAGCAAAGAAGAGGACAAGCCCAAAGTGGAGGATTTGGATGA

AAATGATGATGAAGATGCAGAGAGTAAGGACAAGAGAAAGAAGAAGACCGTCAAAGAAAAATATAACGAA

GAGGAAGAGCTGAACAAGACCAAGCCTCTGTGGACAAGAAATGCTGACGACATTACGCAAGAGGAGTATG

CTGAGTTCTACAAGTCCCTTACCAATGACTGGGAAGACCATCTGGCAGTGAAACATTTCTCAGTAGAAGG

GCAGTTGGAATTCAGAGCTCTCCTATTTATTCCAAAGAGGGCTCCTTTTGATATGTTTGAGAATAAGAAA

AAGAAGAACAACATCAAATTGTATGTACGAAGAGTATTTATTATGGACAACTGTGAAGATCTCATTCCAG

AGTATTTGAACTTTGTGAAGGGTGTTGTGGACTCTGAAGATCTCCCTCTGAATATCTCGAGAGAAATGTT

GCAGCAAAGCAAAATTCTCAAAGTCATCCGAAAGAATCTGGTCAAGAAATGTATTGAACTCATTGAAGAT

GTGGCAGAAGACAAGGAAAATTACAAGAAGTTGTACGAACAGTTTGCCAAAAGTTTAAAGCTCGGCATTC

ATGAAGACAGCACGAACAGGAAAAAGCTGGCTGACTTCTTGAGATACTATTCATCACAGTCAGGAGATGA

ATACACGTCGCTGAAAGACTATGTTTCCCGAATGAAGGAAAACCAAAAGGACATCTACTACATTACTGGT

GAGAGCCGAGAGTCTGTCCAGAACTCAGCATTTGTCGAGCGGGTGAAGAAGAGGGGATTCGAAGTGATCT

ACATGACTGACCCTATTGATGAATACTCTGTGCAGCAGCTGAAGGAGTATGAAGGCAAAAACCTTGTGTG

TGTCACAAAGGAGGGCTTGGAACTCCCAGAGGATGAAGAGCAGAAGAAGAAATTTGAGGAGGCAAAGGCT

CGGTTTGAAGGACTTTGCAAAGTCATGAAGGAGATCCTTGACAAGAAAGTTGAGAAGGTCGTTGTGTCAA

ACCGCCTTGACACATCACCCTGCTGCATCGTCACCTCTCAGTATGGTTGGTCAGCCAATATGGAGAGAAT

CATGAAGGCTCAGGCGCTGCGGGACACCAGCACCATGGGCTACATGGCAGCTAAGAAGCATATGGAGATC

AACCCTGATCATCCTATCATCAACACGCTGAAGGAGAAGGTCGATGCCGACAAGAATGACAAAGCTGTCA

AAGACTTGTGTTTGTTGCTGTTCGAGACATCACTTCTTGCCTCTGGTTTCAGCTTGGAAGATCCTTCTTC

TCATGCCAACAGAATTCATCGAATGGTCAAGCTTGGTTTGGGCATTGATGACGATGATGGTGCCTCTGGT

GATGCTGCTGACGAGGCTGCTGCCGGAGATGAGATTCCAGCTCTGGAAGGAGATGATGATGATGCGTCTC

GCATGGAGGAAGTGGATTAA

>cont2.16199_*Aplysia_californica*_729aa

MPEIQPAEEMEGEKETFAFQAEIAQLMSLIINTFYSNKEIFLRELISNASDALDKIRYESLTDPSRLDSG

KDLQIRIVPDKENKTLIIEDSGIGMTKADLVNNLGTIAKSGTKAFMEALQAGADISMIGQFGVGFYSAYL

IADRVVVESKHNDDELYTWESSAGGSFTIASGTGAPLARGTRITLYVKEDQVEYLEEKRIKDVIKKHSQF

IGYPIKLLVEKEREKEVSDDEEEEEKKEDDKEESKEEDKPKVEDLDENDDEDAESKDKRKKKTVKEKYNE

EEELNKTKPLWTRNADDITQEEYAEFYKSLTNDWEDHLAVKHFSVEGQLEFRALLFIPKRAPFDMFENKK

KKNNIKLYVRRVFIMDNCEDLIPEYLNFVKGVVDSEDLPLNISREMLQQSKILKVIRKNLVKKCIELIED

VAEDKENYKKLYEQFAKSLKLGIHEDSTNRKKLADFLRYYSSQSGDEYTSLKDYVSRMKENQKDIYYITG

ESRESVQNSAFVERVKKRGFEVIYMTDPIDEYSVQQLKEYEGKNLVCVTKEGLELPEDEEQKKKFEEAKA

RFEGLCKVMKEILDKKVEKVVVSNRLDTSPCCIVTSQYGWSANMERIMKAQALRDTSTMGYMAAKKHMEI

NPDHPIINTLKEKVDADKNDKAVKDLCLLLFETSLLASGFSLEDPSSHANRIHRMVKLGLGIDDDDGASG

DAADEAAAGDEIPALEGDDDDASRMEEVD

>cont2.30811_*Aplysia_californica*_cds_

ATGCCTGAAATTCAGACCGCCGATGAGATGGAAGCGGAGAAGGAGACCTTCGCCTTCCAGGCTGAAATTG

CTCAGCTGATGAGTTTGATCATCAACACATTCTACAGTAACAAGGAAATCTTCCTGAGAGAGTTGATCTC

TAATGCTTCTGATGCCTTAGATAAAATCCGATATGAGTCACTCACAGATCCCAGCAGACTGGACTCTGGA

AAAGATCTCCACATTAGAATTGTTCCAGACAGGGACAACAAGACTCTGACCATTGAGGACTCTGGAATTG

GCATGACCAAGGCAGATTTGGTGAACAACCTGGGTACCATTGCCAAGTCAGGAACCAAGGCTTTCATGGA

GGCCTTGCAAGCTGGTGCTGACATTTCTATGATTGGGCAATTTGGTGTAGGTTTCTATTCTGCGTATCTG

GTGGCAGACCGTGTTGTTGTCGAGTCCAAGAACAACGATGATGAGCAGTACACGTGGGAGTCTTCAGCTG

GGGGTTCCTTTACAGTGGCCACTGGAGTTGGCTCTCCTCTGGCCCGTGGCACAAGAATCACTCTGCACTT

CAAAGAGGATCAGATTGAATATCTAGAAGAGAAGAGGATCAAGGATGTGATCAAGAAACACAGCCAGTTC

ATTGGCTATCCCATCAAACTGTTGGTTGAGAAGGAGCGTGACAAGGAAGTGTCTGATGATGAAGAGGAAG

AAGAGAAGAAGGAAGATGACAAGGAGGAGAACAAAGATGAGGACAAGCCCAAAGTGGAGGATTTGGATGA

AAACGATGATGAAGATGCTGACAAAGACAAAAAGAAAAAGAAAACAATCAAAGAGAAGTATAATGAGGAG

GAAGAGTTGAACAAAACCAAACCCCTGTGGACAAGAAATGCAGATGACATTACACAAGAGGAGTATGCAG

AGTTTTACAAATCTCTTACCAATGACTGGGAAGATCACCTGGCTGTGAAACATTTTTCTGTGGAAGGACA

GCTGGAATTTAGAGCTCTTCTCTTCATCCCAAAAAGGGCCCCTTTTGACATGTTTGAGAACAAGAAAAAG

AAGAACAATATCAAATTGTATGTGAGAAGAGTGTTCATCATGGACAACTGTGAAGATCTCATCCCAGAGT

ATTTGAACTTTGTCAAGGGTGTCGTGGACTCTGAAGATCTCCCTCTGAATATTTCAAGAGAAATGTTGCA

ACAAAGCAAAATTCTGAAGGTCATTCGTAAGAACCTTGTCAAAAAGTGTGTTGAACTCATTGAAGACCTC

GCAGAAGACAAAGAGAATTACAAGAAGTTCTATGAACAGTTCGGCAAAAATCTGAAGCTGGGCATCCATG

AAGACAGTACAAACAGGAAGAAGCTGGCAGACTTTTTGAGATACTATTCATCACAGTCTGGAGATGAGAT

GACATCTTTGAAAGATTATGTTTCACGGATGAAGGAGAATCAAAAAGACATCTACTACATCACAGGTGAG

AGCAGAGAAGCTGTCCAAAACTCTGCTTTTGTAGAGCGTGTGAAGAAGAGGGGATTTGAAGTGATCTATA

TGATTGACCCCATTGATGAGTACTCTGTACAGCAGCTGAAGGAGTATGAGGGCAAGAACCTCGTGTGTGT

CACAAAGGAGGGCTTGGAGCTCCCTGAGGATGAAGAGGAAAAGAAGAAGTTTGAGGAGGCCAAGGCCCGC

TTTGAGGGACTCTGCAAAGTCATGAAGGAAATTCTTGACAAGAAGGTTGAAAAGGTTGTTGTGTCAAACC

GCCTGGTCACATCCCCATGCTGTATTGTCACATCTCAATATGGCTGGTCCGCCAACATGGAGAGAATCAT

GAAGGCTCAAGCACTCCGTGATACCAGCACTATGGGTTACATGGCAGCCAAAAAGCACCTGGAGATCAAC

CCAGATCATCCTATTGTCAACACCCTGAAGGAGAAAGCAGATGCTGACAAGAATGACAAAGCTGTGAAGG

ACTTGTGCTTGTTGCTGTTTGAGACCTCTCTTCTTGCTTCAGGTTTCAGCCTTGAAGACCCCACCTCCCA

TGCCAACAGAATTCACCGAATGATCAAGCTCGGTTTGGGAATTGATGATGACGATGGTGCCTCTGGTGAT

GCTGCTGTCGAGGCTGCAGGAGAAGAGATTCCACCTCTGGAAGGAGATGATGATGATGCATCTCGCATGG

AGGAAGTGGACTAA

>cont2.30811_*Aplysia_californica*_727aa

MPEIQTADEMEAEKETFAFQAEIAQLMSLIINTFYSNKEIFLRELISNASDALDKIRYESLTDPSRLDSG

KDLHIRIVPDRDNKTLTIEDSGIGMTKADLVNNLGTIAKSGTKAFMEALQAGADISMIGQFGVGFYSAYL

VADRVVVESKNNDDEQYTWESSAGGSFTVATGVGSPLARGTRITLHFKEDQIEYLEEKRIKDVIKKHSQF

IGYPIKLLVEKERDKEVSDDEEEEEKKEDDKEENKDEDKPKVEDLDENDDEDADKDKKKKKTIKEKYNEE

EELNKTKPLWTRNADDITQEEYAEFYKSLTNDWEDHLAVKHFSVEGQLEFRALLFIPKRAPFDMFENKKK

KNNIKLYVRRVFIMDNCEDLIPEYLNFVKGVVDSEDLPLNISREMLQQSKILKVIRKNLVKKCVELIEDL

AEDKENYKKFYEQFGKNLKLGIHEDSTNRKKLADFLRYYSSQSGDEMTSLKDYVSRMKENQKDIYYITGE

SREAVQNSAFVERVKKRGFEVIYMIDPIDEYSVQQLKEYEGKNLVCVTKEGLELPEDEEEKKKFEEAKAR

FEGLCKVMKEILDKKVEKVVVSNRLVTSPCCIVTSQYGWSANMERIMKAQALRDTSTMGYMAAKKHLEIN

PDHPIVNTLKEKADADKNDKAVKDLCLLLFETSLLASGFSLEDPTSHANRIHRMIKLGLGIDDDDGASGD

AAVEAAGEEIPPLEGDDDDASRMEEVD

>cont2.59716_*Aplysia_californica*_cds_2184bp

ATGCCTGAAATTCAGACCGCCGATGAGATGGAAGCGGAGAAGGAGACCTTCGCCTTCCAGGCTGAAATTG

CTCAGCTGATGAGTTTGATCATCAACACATTCTACAGTAACAAGGAAATCTTCCTGAGAGAGTTGATCTC

TAATGCTTCTGATGCCTTAGATAAAATCCGATATGAGTCACTCACAGATCCCAGCAGACTGGACTCTGGA

AAAGATCTCCACATTAGAATTGTTCCAGACAGGGACAACAAGACTCTGACCATTGAGGACTCTGGAATTG

GCATGACCAAGGCAGATTTGGTGAACAACCTGGGTACCATTGCCAAGTCAGGAACCAAGGCTTTCATGGA

GGCCTTGCAAGCTGGTGCTGACATTTCTATGATTGGGCAATTTGGTGTAGGTTTCTATTCTGCGTATCTG

GTGGCAGACCGTGTTGTTGTCGAGTCCAAGAACAACGATGATGAGCAGTACACGTGGGAGTCTTCAGCTG

GGGGTTCCTTTACAGTGGCCACTGGAGTTGGCTCTCCTCTGGCCCGTGGCACAAGAATCACTCTGCACTT

CAAAGAGGATCAGATTGAATATCTAGAAGAGAAGAGGATCAAGGATGTGATCAAGAAACACAGCCAGTTC

ATTGGCTATCCCATCAAACTGTTGGTTGAGAAGGAGCGTGACAAGGAAGTGTCTGATGATGAAGAGGAAG

AAGAGAAGAAGGAAGATGACAAGGAGGAGAACAAAGATGAGGACAAGCCCAAAGTGGAGGATTTGGATGA

AAACGATGATGAAGATGCTGACAAAGACAAAAAGAAAAAGAAAACAATCAAAGAGAAGTATAATGAGGAG

GAAGAGTTGAACAAAACCAAACCCCTGTGGACAAGAAATGCAGATGACATTACACAAGAGGAGTATGCAG

AGTTTTACAAATCTCTTACCAATGACTGGGAAGATCACCTGGCTGTGAAACATTTTTCCGTGGAAGGACA

GCTGGAATTTAGAGCTCTTCTCTTCATCCCAAAAAGGGCCCCTTTTGACATGTTTGAGAACAAGAAAAAG

AAGAACAATATCAAATTGTATGTGAGAAGAGTGTTCATCATGGACAACTGTGAAGATCTCATCCCAGAGT

ATTTGAACTTTGTCAAGGGTGTCGTGGACTCTGAAGATCTCCCTCTGAATATTTCAAGAGAAATGTTGCA

ACAAAGCAAAATTCTGAAGGTCATTCGTAAGAACCTTGTCAAAAAGTGTGTTGAACTCATTGAAGACCTC

GCAGAAGACAAAGAGAATTACAAGAAGTTCTATGAACAGTTCGGCAAAAATCTGAAGCTGGGCATCCATG

AAGACAGTACAAACAGGAAGAAGCTGGCAGACTTTTTGAGATACTATTCATCACAGTCTGGAGATGAGAT

GACATCTTTGAAAGATTATGTTTCACGGATGAAGGAGAATCAAAAAGACATCTACTACATCACAGGTGAG

AGCAGAGAAGCTGTCCAAAACTCTGCTTTTGTAGAGCGTGTGAAGAAGAGGGGATTTGAAGTGATCTACA

TGATTGACCCCATTGATGAGTACTCTGTACAGCAGCTGAAGGAGTATGAGGGCAAGAACCTCGTGTGTGT

CACAAAGGAGGGCTTGGAGCTCCCTGAGGATGAAGAGGAAAAGAAGAAGTTTGAGGAGGCCAAGGCCCGC

TTTGAGGGACTCTGCAAAGTCATGAAGGAAATTCTTGACAAGAAGGTTGAAAAGGTTGTTGTGTCAAACC

GCCTGGTCACATCCCCATGCTGTATTGTCACATCTCAATATGGCTGGTCCGCCAACATGGAGAGAATCAT

GAAGGCTCAAGCACTCCGTGATACCAGCACTATGGGTTACATGGCAGCCAAAAAGCACCTGGAGATCAAC

CCAGATCATCCTATTGTCAACACCCTGAAGGAGAAAGCAGATGCTGACAAGAATGACAAAGCTGTGAAGG

ACTTGTGCTTGTTGCTGTTTGAGACCTCTCTTCTTGCTTCAGGTTTCAGCCTTGAAGACCCCACCTCCCA

TGCCAACAGAATTCACCGAATGATCAAGCTCGGTTTGGGAATTGATGATGACGATGGTGCCTCTGGTGAT

GTTGCTGTCGAGGCTGCAGGAGAAGAGATTCCACCTCTGGAAGGAGATGATGATGATGCATCTCGCATGG

AGGAAGTGGACTAA

>cont2.59716_*Aplysia_californica*_727aa

MPEIQTADEMEAEKETFAFQAEIAQLMSLIINTFYSNKEIFLRELISNASDALDKIRYESLTDPSRLDSG

KDLHIRIVPDRDNKTLTIEDSGIGMTKADLVNNLGTIAKSGTKAFMEALQAGADISMIGQFGVGFYSAYL

VADRVVVESKNNDDEQYTWESSAGGSFTVATGVGSPLARGTRITLHFKEDQIEYLEEKRIKDVIKKHSQF

IGYPIKLLVEKERDKEVSDDEEEEEKKEDDKEENKDEDKPKVEDLDENDDEDADKDKKKKKTIKEKYNEE

EELNKTKPLWTRNADDITQEEYAEFYKSLTNDWEDHLAVKHFSVEGQLEFRALLFIPKRAPFDMFENKKK

KNNIKLYVRRVFIMDNCEDLIPEYLNFVKGVVDSEDLPLNISREMLQQSKILKVIRKNLVKKCVELIEDL

AEDKENYKKFYEQFGKNLKLGIHEDSTNRKKLADFLRYYSSQSGDEMTSLKDYVSRMKENQKDIYYITGE

SREAVQNSAFVERVKKRGFEVIYMIDPIDEYSVQQLKEYEGKNLVCVTKEGLELPEDEEEKKKFEEAKAR

FEGLCKVMKEILDKKVEKVVVSNRLVTSPCCIVTSQYGWSANMERIMKAQALRDTSTMGYMAAKKHLEIN

PDHPIVNTLKEKADADKNDKAVKDLCLLLFETSLLASGFSLEDPTSHANRIHRMIKLGLGIDDDDGASGD

VAVEAAGEEIPPLEGDDDDASRMEEVD

>Lotgi1 sca_30_*Lottia_gigantea*_cds_2175bp

ATGCCGGAAATTCAACAGACTGATGATGGTGGGGAGGTTGAAACCTTCGCATTTCAAGCGGAAATTGCTC

AGTTGATGAGTTTGATCATCAACACCTTTTACTCTAACAAAGAAATCTTCTTGAGAGAACTGATCTCCAA

CGCTTCTGATGCTTTAGATAAAATCCGATATGAAAGTTTGACAGATCCATCCAAATTGGACAGTGGAAAA

GATCTCCAAATCAGAATTGTCCCTGACCGAGAAAACAAAACTTTGACCATTGAAGACACTGGTATTGGAA

TGACCAAGGCTGATTTAGTTAACAACTTAGGTACCATTGCCAAATCTGGAACCAAAGCTTTCATGGAGGC

TCTCCAAGCTGGTGCCGATATTTCTATGATTGGTCAATTTGGTGTGGGTTTCTACTCTGCCTATTTGGTA

GCTGATAAAGTGGTTGTAGAATCTAACAATAACGATGATGAGGCCTATATTTGGGAATCCTGTGCTGGAG

GTTCATTCACTGTACGACACATTGACAATCCAACTGTAAGCAGAGGAACCAAGATAACTCTGTACCTGAA

AGAAGATCAAGCTGAATATTTAGAGGAGCGAAGAATCAAGGAAGTGATCAAGAAACACAGTCAGTTTATT

GGGTATCCTATTAAATTATTGGTCGAGAAAGAACGAGAAAAGGAAGTGTCAGATGATGAAGAGGAAGAAG

AGGAAAAGAAGGAAGAAAAAGATGATGAAGGACAAGATAAACCAAAAGTAGAAGATCTTGAAGAAGATGA

AGATGCTGATAAAGAAGGAAAAGACAAGAAAAAGAAGAAAATTAAAGAAAAATACACAGAAGATGAGGAA

TTGAACAAAACTAAGCCAATCTGGACTAGAAATCCTGATGATATTACTCAAGAAGAATATGCTGAATTCT

ATAAATCTTTAACCAACGATTGGGAAGATCATTTAGCTGTCAAACATTTCTCTGTAGAAGGTCAACTTGA

GTTCCGTGCTTTGTTATATCTACCAAAACGAGCTCCATTTGACATGTTCGAAAACAAGAAAAAGAAAAAC

AACATCAAATTATACGTACGAAGAGTATTCATTATGGACAATTGTGAGGAGATTATCCCAGAATATCTTA

ACTTTATTCGTGGTGTGGTGGATTCGGAAGATTTACCTCTGAATATTTCTCGTGAAATGTTGCAACAGAG

TAAAATCCTGAAAGTTATTCGAAAGAATCTGGTGAAGAAGGCCATGGAACTTTTTGATGACATCGCTGCT

GATAAAGACAACTTTAAAAAATTCTACGAACAATTCAGCAAAAACTTGAAGCTTGGTATTCATGAAGATA

CAACCAATCGTAAGAAACTGGCTGATCTATTGAGATATTATACCTCTAGCTCTGGTGATGAGATGACCAG

TCTTAAAGAATATGTATCTAGAATGAAGGAGAACCAGAAGTCTATTTACTACATTACTGGTGAAACCAAA

GAAGCAGTTCAGAATTCTGCTTTTGTTGAACGAGTACGAAAGCGAGGATTTGAAGTTATTTACATGATTG

ATCCTATTGATGAGTACGCCATCCAACAATTGAAAGAGTATGACGGCAAGACGCTGGTCTGTGTTACCAA

GGAAGGTCTGGAATTACCAGAAGATGAAGAGGAAAAGAAGAGATTCGAAGAAGCCAAGGCCAACTTTGAA

GGATTATGTAAAGTCATCAAAGAAATCCTTGATAAGAAAGTTGAAAAGGTTGTGGTATCAAACAGATTGG

TATCATCGCCGTGTTGTATCGTCACCAGTCAGTACGGTTGGTCAGCCAACATGGAGAGGATCATGAAGGC

ACAAGCATTACGTGATACTAGCACCATGGGATACATGGCTGCCAAAAAACATTTAGAAATCAACCCAGAT

CATCCTATTGTGAAAACATTGAAAGAGAAGGCTGATGCTGACAAAAATGACAAGAGTGTTAAAGACTTAG

TTTTGTTGTTATTCGAAACCTCTTTATTAGCTTCTGGATTTACACTGGAGGATCCTACATCACACGCAAA

CCGAATTCATAGAATGGTCAAGTTAGGTCTAGGAATCGATATCGATGATGATGGCAGTGGCGATGCTATG

GAAGCAGCTACAGACGACATGCCGCCATTAGAAGGTGACGATGATGATACATCCAAGATGGAGGAAGTGG

ATTAA

>Lotgi1 sca_30_*Lottia_gigantea*_724aa

MPEIQQTDDGGEVETFAFQAEIAQLMSLIINTFYSNKEIFLRELISNASDALDKIRYESLTDPSKLDSGK

DLQIRIVPDRENKTLTIEDTGIGMTKADLVNNLGTIAKSGTKAFMEALQAGADISMIGQFGVGFYSAYLV

ADKVVVESNNNDDEAYIWESCAGGSFTVRHIDNPTVSRGTKITLYLKEDQAEYLEERRIKEVIKKHSQFI

GYPIKLLVEKEREKEVSDDEEEEEEKKEEKDDEGQDKPKVEDLEEDEDADKEGKDKKKKKIKEKYTEDEE

LNKTKPIWTRNPDDITQEEYAEFYKSLTNDWEDHLAVKHFSVEGQLEFRALLYLPKRAPFDMFENKKKKN

NIKLYVRRVFIMDNCEEIIPEYLNFIRGVVDSEDLPLNISREMLQQSKILKVIRKNLVKKAMELFDDIAA

DKDNFKKFYEQFSKNLKLGIHEDTTNRKKLADLLRYYTSSSGDEMTSLKEYVSRMKENQKSIYYITGETK

EAVQNSAFVERVRKRGFEVIYMIDPIDEYAIQQLKEYDGKTLVCVTKEGLELPEDEEEKKRFEEAKANFE

GLCKVIKEILDKKVEKVVVSNRLVSSPCCIVTSQYGWSANMERIMKAQALRDTSTMGYMAAKKHLEINPD

HPIVKTLKEKADADKNDKSVKDLVLLLFETSLLASGFTLEDPTSHANRIHRMVKLGLGIDIDDDGSGDAM

EAATDDMPPLEGDDDDTSKMEEVD

>*Mytilus_californianus_*cds_1365 (library SHGC)

ATGCCAGAACCAGAACAAACGATGGATGAAGGAGAGGTTGAAACTTTTGCTTTCCAGGCAGAAATTGCCC

AGCTGATGAGTTTGATCATCAATACTTTCTATTCCAACAAAGAAACCTTCTTGAGAGAATTGATCTCCAA

CTCATCTGATGCCTTGGATAAGATCAGATATGAAAGTTTGACTGATCCTTCAAAACTGGACCTTGGAAAA

GACCTGGAAATCAGAATAATACCAGACAAGGACAACAACACACTGACCATCATTGATACTGGTATTGGAA

TGACCAAAGCTGACCTGGTCAATAACTTGGGTACCATTGCCAAGTCTGGTACTAAAGCTTTCATGGAGGC

TCTTCAAGCTGGAGCTGATATTTCTATGATTGGACAGTTTGGTGTAGGTTTCTACTCTGCCTACCTTGTT

GCTGATAAAGTAATTGTCCAGACAAGGAACAACGATGATGAGGAATATATCTGGGAATCAGCAGCAGGTG

GATCATTTACAGTCAAAACAGCAACAGGCGAGTCCATAGGTAGAGGTACCAAGATTACACTTTACATGAA

AGAAGACCAGGCTGAATATTTGGAAGAAAAAAGAATCAAAGAAGTTGTGAAGAAACACAGTCAGTTCATT

GGTTATCCAATCAAATTATTGGTAGAGAAAGAACGTGACAAAGAAGTTTCAGATGATGCCTTTGTTGAGA

GACTGAGGAAGCGAGGACTTGAAGTTATTTACATGATTGACCCCATTGACGAGTATGCCGTACAGCAATT

GAAAGAGTATGATGGCAAAAATCTGGTCAGTGTAACCAAAGAAGGTCTAGAACTCCCAGAAGATGAGGAG

GAAAAGAAAAAGTTTGAGGAGGATAAAGCTGCTTTTGAAGGCTTGTGCAAAGTAATGAAAGACATCCTGG

ACAAGAAAGTAGAGAAGGTAACAGTTTCCAACAGACTGGTAACCTCCCCTTGTTGCATTGTAACAAGTCA

ATACGGTTGGTCAGCTAACATGGAAAGGATCATGAAAGCCCAAGCTTTGAGAGATACCAGCACCATGGGT

TACATGGCAGCCAAGAAACATTTAGAAATTAATCCGGAGCATGCAATTATCAAATCGCTGAAAGAAAAAT

CTGATGCTGATAAGAATGACAAAGCTGTGAAAGACTTAGTTGTGTTGTTGTTTGAAACGTCTTTGTTAGC

ATCCGGGTTCTCACTAGAGGAGCCACAGAGTCATGCCAACAGAATTCATAGAATGATCAAATTAGGTTTA

GGAATTGATGAAGAAGATGTACCAGTAGAACAAACAACAACAGAAGAAATGCCACCATTAGAAGGAGACG

AAGATGATGCATCACGGATGGAAGAAGTCGACTAA

>*Mytilus_californianus_*454aa (library SHGC)

MPEPEQTMDEGEVETFAFQAEIAQLMSLIINTFYSNKETFLRELISNSSDALDKIRYESLTDPSKLDLGK

DLEIRIIPDKDNNTLTIIDTGIGMTKADLVNNLGTIAKSGTKAFMEALQAGADISMIGQFGVGFYSAYLV

ADKVIVQTRNNDDEEYIWESAAGGSFTVKTATGESIGRGTKITLYMKEDQAEYLEEKRIKEVVKKHSQFI

GYPIKLLVEKERDKEVSDDAFVERLRKRGLEVIYMIDPIDEYAVQQLKEYDGKNLVSVTKEGLELPEDEE

EKKKFEEDKAAFEGLCKVMKDILDKKVEKVTVSNRLVTSPCCIVTSQYGWSANMERIMKAQALRDTSTMG

YMAAKKHLEINPEHAIIKSLKEKSDADKNDKAVKDLVVLLFETSLLASGFSLEEPQSHANRIHRMIKLGL

GIDEEDVPVEQTTTEEMPPLEGDEDDASRMEEVD

>pfu_ver1.0_*Pinctada_fucata­*_cds_2178bp

ATGCCTGAACCAGAACAAATGCAGGAGGAGGGAGAGGTTGAGACCTTTGCTTTTCAAGCAGAGATTGCTC

AGCTTATGAGCTTGATCATCAACACCTTCTACTCAAACAAAGAAATCTTTCTTAGAGAGTTGATTTCCAA

CTCATCTGACGCCTTGGACAAAATCAGATATGAAAGCCTGACAGATCCATCCAAGCTCGACTCTGGCAAG

GATCTATTTATCAAGATCACCCCTGACAAGGATGCCAAAACTTTGACCATTGAGGACACAGGGATTGGTA

TGACCAAGGCTGATCTTGTCAACAACTTGGGTACTATTGCTAGATCAGGTACCAAGGCTTTCATGGAGGC

CCTGCAAGCTGGAGCCGACATCTCCATGATTGGACAGTTTGGTGTAGGTTTCTACTCCGCCTACTTGGTT

GCTGACAAGGTAGTTGTCGAGACCAAGAACAACGATGATGAGCAGTACATTTGGGAGTCCAGCGCTGGTG

GTTCATTCTCAGTCAGACAAACCAAGGAAACTGATAACTGGAGAGGTACTCGTATCACCTTGCACCTGAA

GGAGGACCAAGTTGAGTATTTGGAGGAACGTCGCGTGAAGGAGGTGGTGAAGAAACACAGCCAGTTCATT

GGCTATCCCATCAAACTGCTCGTGGAGAAAGAGAGGGACAAGGAAGTATCTGATGATGAGGAAGAGGAGG

AAAAGAAAGAGGAGGACAAAGATGAGGAGAAGAAAGAGGAAGACAAACCAAAGGTTGAAGATTTAGATGA

GGATGAAGAAGACGAAGGCAAGAAGGACACTAAGAAGAAGAAGAAGATCAAGGAAAAATACACAGAAGAT

GAGGAACTGAACAAAACCAAGCCAATTTGGACCCGTAACCCAGACGATATCACCCAAGAAGAATATGGAG

AGTTCTACAAATCACTCACCAATGATTGGGAGGACCATTTGGCTGTAAAGCACTTCTCAGTGGAAGGTCA

GCTTGAATTCCGCGCCCTGCTCTTCATCCCACGACGTGCTCCATTCGACTTGTTTGAAAACAAAAAGAAG

AAGAACAACATCAAATTGTATGTGCGTAGAGTATTCATCATGGACAATTGTGAAGAGTTGATTCCCGAGT

ATTTGAACTTTGTGCGTGGTGTTGTAGACTCTGAGGACTTGCCATTAAACATTTCCAGAGAAATGTTGCA

ACAAAGTAAAATCCTGAAAGTGATCAGGAAAAATCTGGTGAAAAAGTGTGTTGAACTCTTTGACGAGATT

GCAGAAGACAAAGATAACTACAAAAAATTCTACGAACAATTCAGCAAGAATCTCAAGCTTGGTATCCATG

AAGATTCCACCAACAGGAAGAAGCTTGCAGATTACTTGCGTTACTACTCCTCTCAGTCCGGAGACGAATT

GACGTCACTCAAAGATTACGTTTCCAGAATGAAGGAGAACCAGAAATGCATTTACTACATCACCGGTGAG

AGCAAGGAGGCTGTGATGAACTCAGCATTTGTGGAACGTGTGAAGAAGAGGGGAATGGAGGTCATCTACA

TGGTAGACCCCATCGATGAATACTCAGTCCAACAGCTGAAGGAATACGACGGCAAGACCTTAACAAACGT

CACAAAAGAAGGTCTGGAACTCCCAGAAGATGAGGAGGAGAAGAAAAAGTTTGAGGAAGCCAAGGCAGAA

TTTGAAGGACTGTGCAAGACCATGAAGGAAATCCTTGACAAAAAAGTGGAAAAGGTGGTAGTATCAAACA

GACTGGTCACATCTCCGTGCTGCATCGTCACATCACAATACGGTTGGTCAGCTAACATGGAGAGAATCAT

GAAGGCCCAGGCGCTAAGGGACTCGAGCACCATGGGATACATGGCCGCCAAGAAACACCTTGAAGTTAAT

CCTGACCATTCCATTATCAAAGCATTGAAAACCAAAGCAGAAGCAGACAAAAATGATAAGAGTGTGAAAG

ACTTAGTATTACTTTTGTTTGAAACATCTCTGTTATCATCTGGATTCACATTAGAGGAACCTGGACTACA

CGCCAGCAGAATACACAGAATGATCAACCTTGGTCTAGGTATTGACGAAGAGGAAACACAAACGACAGAG

ACGACGGAAGGATCAGAAGAAATGCCACCGCTAGAGGGAGATGATGATGATGCTTCAAGGATGGAAGAAG

TTGATTAA

>pfu_ver1.0_*Pinctada_fucata­*_725aa

MPEPEQMQEEGEVETFAFQAEIAQLMSLIINTFYSNKEIFLRELISNSSDALDKIRYESLTDPSKLDSGK

DLFIKITPDKDAKTLTIEDTGIGMTKADLVNNLGTIARSGTKAFMEALQAGADISMIGQFGVGFYSAYLV

ADKVVVETKNNDDEQYIWESSAGGSFSVRQTKETDNWRGTRITLHLKEDQVEYLEERRVKEVVKKHSQFI

GYPIKLLVEKERDKEVSDDEEEEEKKEEDKDEEKKEEDKPKVEDLDEDEEDEGKKDTKKKKKIKEKYTED

EELNKTKPIWTRNPDDITQEEYGEFYKSLTNDWEDHLAVKHFSVEGQLEFRALLFIPRRAPFDLFENKKK

KNNIKLYVRRVFIMDNCEELIPEYLNFVRGVVDSEDLPLNISREMLQQSKILKVIRKNLVKKCVELFDEI

AEDKDNYKKFYEQFSKNLKLGIHEDSTNRKKLADYLRYYSSQSGDELTSLKDYVSRMKENQKCIYYITGE

SKEAVMNSAFVERVKKRGMEVIYMVDPIDEYSVQQLKEYDGKTLTNVTKEGLELPEDEEEKKKFEEAKAE

FEGLCKTMKEILDKKVEKVVVSNRLVTSPCCIVTSQYGWSANMERIMKAQALRDSSTMGYMAAKKHLEVN

PDHSIIKALKTKAEADKNDKSVKDLVLLLFETSLLSSGFTLEEPGLHASRIHRMINLGLGIDEEETQTTE

TTEGSEEMPPLEGDDDDASRMEEVD

**CHONDRICHTHYES**

>*Callorhinchus_milii*_cds_2193bp

ATGCCCGAGGAAGTCCAGAACGCTTCTTCTCAAGACATGGCGACGGAGGAAGAAGTTGAAACTTTTGCCT

TCCAGGCAGAAATCGCCCAGTTGATGTCCCTGATCATCAATACGTTCTACTCCAACAAAGAGATCTTTCT

GAGAGAGATTATTTCTAACTCCTCTGATGCACTTGATAAAATCCGCTATGAAAGCCTGACTGATCCAAGC

AAGCTGGATTCTGGCAAAGAGCTGAAGATTGACATTACGCCAAACCAAAAGGAACGCACTTTAACCATTT

GGGATACTGGTATTGGGATGACCAAAGCTGATCTCATTAACAACCTGGGTACCATTGCTAAGTCTGGAAC

AAAGGCCTTTATGGAAGCTTTGCAGGCAGGTGCAGATATCTCCATGATTGGCCAGTTTGGTGTTGGCTTC

TATTCTGCCTATCTTGTTGCAGAAAAGGTTACTGTCATCACCAAACACTGTGATGATGAACAGTATATTT

GGGAATCATCTGCTGGAGGATCGTTCACAGTGAGATTAGATCAGGGTGAGCCACTGGGTCGTGGGACCAA

AATAACCTTGCACTTGAAAGAGGATCAAATGGAATATATAGAAGAGAAGAGGGTCAAAGAGGTTGTGAAG

AAGCACTCTCAGTTCATTGGCTACCCAATCACACTTTATGTGGAGAAAGAACGTGATAAGGAGATCAGTG

ATGATGAAGCAGAAGAGAAGGAAGAGAAGAAAGAAGATGCAAGTGAGGAAGACAAACCAAAAATAGAAGA

TGTTGGATCAGATGAAGAAGAAGATGAAAAGAAAGAAGGTGAGAAGAAAAAGAAGAAGAAGATCAAGGAA

AAATACATTGATCAAGAGGAGCTGAACAAGACCAAACCACTTTGGACTAGGAATCCTGAGGACATTACCA

CTGAAGAGTATGGAGAATTTTATAAGAGTCTGACAAATGACTGGGAGGAACACTTGGCTGTAAAGCATTT

CTCTGTTGAGGGCCAGCTGGAGTTCAGAGCTTTACTCTTTGTTCCACGACGGGCACCATTTGACTTATTT

GAAAACCGAAAGAAGAAGAACAATATCAAGCTCTATGTACGCAGAGTCTTCATCATGGATAATTGTGATG

AGCTTATTCCAGAATATCTTAATTTTATGAAAGGTGTTGTGGACTCTGAGGACCTACCATTAAACATTTC

TCGTGAAACGTTGCAGCAAAGCAAGATTCTGAAAGTTATTCGTAAGAATCTTGTTAAGAAATGTATGGAG

CTCTTCATAGAATTGTCTGAGGACAAGGACAACTACAAGAAATTATATGATCAGTTTTCCAAAAACCTTA

AGCTTGGAATCCATGAGGACTCCCAGAATCGTAAGAAGCTCTCAGAGCTGCTCAGGTACCATACCTCAGT

GAGTGGTGATGAAATGGTGTCCCTCAAAGACTATGTCTCACGCATGAAAGACAACCAAAAGCACATTTAC

TACATAACTGGTGAAACCAAGGATCAAGTGGCTAACTCTGCTTTTGTAGAGTGGCTGAGAAAACATGGAT

TGGAAGTGATCTATATGACTGAGCCAATTGATGAATACTGTGTGCAGCAGTTGAAGGAATTTGATGGCAA

GACTTTGGTTTCAGTCACTAAAGAGGGTTTAGAGCTTCCAGAAGATGAAGAAGAGAAAAAGAAACAAGAA

GAGAAAAGGAGCAGTTATGAGAATCTGTGCAAGATCATGAAAGACATATTGGAGAAGAAAATTGAGAAAG

TAACTGTCTCCAACCGCTTAGTTTCCTCTCCGTGTTGCATTGTGACCAGCACTTATGGGTGGACTGCCAA

CATGGAAAGAATCATGAAGGCTCAGGCTCTTCGTGATAACTCTACAATGGGTTATATGGCAGCCAAGAAA

CATCTAGAGATCAATCCTGATCATCCAATCATTGCAACACTGAAGCAGAAGGCAGAAGATGACAAAAATG

ACAAGTCGGTGAAGGATCTTGTCATTCTTCTCTTCGAAACGGCACTACTATCTTCAGGTTTTTCGTTGGA

AGATCCACAAACTCATGCTAATCGAATCTACAGGATGATCAAACTTGGTCTTGGAATTGATGAGGAGGAC

CCAATTGCAGATGATGCTGCCCAGCCAGCTACAGAGGAGATGCCACCATTAGAAGACGATGATGATGCAT

CTCGAATGGAAGAAGTTGACTGA

# >*Callorhinchus_milii*_Hsp90_730_aa

MPEEVQNASSQDMATEEEVETFAFQAEIAQLMSLIINTFYSNKEIFLREIISNSSDALDKIRYESLTDPS

KLDSGKELKIDITPNQKERTLTIWDTGIGMTKADLINNLGTIAKSGTKAFMEALQAGADISMIGQFGVGF

YSAYLVAEKVTVITKHCDDEQYIWESSAGGSFTVRLDQGEPLGRGTKITLHLKEDQMEYIEEKRVKEVVK

KHSQFIGYPITLYVEKERDKEISDDEAEEKEEKKEDASEEDKPKIEDVGSDEEEDEKKEGEKKKKKKIKE

KYIDQEELNKTKPLWTRNPEDITTEEYGEFYKSLTNDWEEHLAVKHFSVEGQLEFRALLFVPRRAPFDLF

ENRKKKNNIKLYVRRVFIMDNCDELIPEYLNFMKGVVDSEDLPLNISRETLQQSKILKVIRKNLVKKCME

LFIELSEDKDNYKKLYDQFSKNLKLGIHEDSQNRKKLSELLRYHTSVSGDEMVSLKDYVSRMKDNQKHIY

YITGETKDQVANSAFVEWLRKHGLEVIYMTEPIDEYCVQQLKEFDGKTLVSVTKEGLELPEDEEEKKKQE

EKRSSYENLCKIMKDILEKKIEKVTVSNRLVSSPCCIVTSTYGWTANMERIMKAQALRDNSTMGYMAAKK

HLEINPDHPIIATLKQKAEDDKNDKSVKDLVILLFETALLSSGFSLEDPQTHANRIYRMIKLGLGIDEED

PIADDAAQPATEEMPPLEDDDDASRMEEVD

>*Leucoraja*_*erinacea*_cds_2199bp

ATGCCTGAAGAGATTCGCACTGACGATGAGTTGGAGACGTTTGCCTTCCAGGCTGAGATTGCTCAGCTTA

TGTCCTTGATCATCAACACCTTCTATTCCAACAAAGAAATTTTCCTCCGAGAGCTCATCTCCAATGCTTC

CGATGCCCTGGACAAGATCCGATATGAAAGCCTGACTGATCCTTCAAAAATGGAAAGTTGCAAGGACCTG

AAGATTGAAATTATTCCCAATATACGGGAGCGTACACTGACCGTCATTGACACCGGGATTGGCATGACCA

AGGCTGACCTGATCAACAACCTGGGCACTATTGCCAAGTCTGGCACAAAGGCCTTCATGGAAGCTCTGCA

GGCTGGTGCCGACATTTCCATGATCGGGCAGTTTGGTGTTGGCTTCTACTCTGCTTACCTGGTGGCCGAG

AAGGTTACAGTGTTCACCAAGCACAACGATGACGAGTCATACTGCTGGGAATCGTCCGCTGGAGGCTCCT

TCACTGTCAAAACCCACAGTGGGAAGGGTGATGCTGGTGTGCCTGATACAATCACTCGCGGTACAACAGT

TATCCTACAGCTAAAGGAGGATCAGGCTGAGTATCTGGAGGAGAAGAGAATCAAGGAGGTTGTGAAGAAA

CACTCTCAGTTCATCGGCTATCCCATCACTCTCTTTGTGGAGAAGGAGCGGGAGAAGGAGATCAGTGATG

ACGAGGCAGAGCCGGAGGACAAGGCCGAGAAGGAGGAAGGAGTGGAGGCAGAGGAAGGGGAGAAGCCCAA

GATCGAAGATGTGGGCTCAGATGAAGAGGAGGACGGTGAAAAAAAGGACAAGAAGAAAACCAAGAAAATC

AAGGAGAAGTATATAGACCAGGAGGAACTCAACAAAACCAAGCCCATCTGGACCCGCAATCCTGACGACA

TCACACAGGAGGAGTATGGCGAGTTCTATAAGAGTCTCACCAATGACTGGGAGGACCACCTGGCTGTGAA

GCACTTTTCTGTCGAAGGGCAGCTAGAATTCCGAGCCCTGTTGTTCCTTCCAAGGCGGGCTCCCTTCGAC

CTCTTTGAAAACAAGAAGAAGAAGAACAACATCAAGCTGTACGTCCGCAGAGTCTTCATTATGGATAGCT

GCGAGGAGCTGATCCCCGAATACCTAAACTTTGTGCGGGGTGTGGTGGACTCCGAGGACCTGCCCTTGAA

TATCTCCCGAGAGATGCTTCAGCAGAGCAAAATTCTAAAGGTCATCCGCAAGAACATTGTCAAGAAATGC

ATGGAATTGTTTGGTGAACTGTCAGAAGACAAGGAGAACTACAAAAAATTCTATGAACAATTCTCAAAGA

ATCTCAAGCTTGGTATCCATGAGGATTCTCAGAATCGCAAGAAGCTGTCAGAAATGCTGCGTTACCATAC

GTCTCAGTCCGGAGATGAGCTCACACCCCTGTCTGACTACGTCTCTCGCATGAAGGAGAACCAGAAGTGC

ATTTACTACATTACTGGTGAAAGCAAGGACCAGGTGGCTAACTCCGCATTCGTGGAGCGCGTGAGGAAGC

GGGGCTTCGAGGTGGTCTACATGACTGAGCCCATCGACGAGTACTGTGTGCAGCAGCTGAAGGAGTTTGA

TGGCAAGCAGCTGGTGTCTGTCACCAAAGAGGGGCTGGAGTTGCCTGAGGATGATGATGAGAAGAAGAAG

CGTGAAACTGACCGGACCAAGTACGATGGTCTCTGCAAACTGATGAAGGAGATCCTGGACAAGAAGGTGG

AGAAGGTGACCGTTTCCAACCGTCTGGTTTCCTCGCCTTGCTGCATCGTGACCAGCACCTATGGCTGGAC

TGCCAACATGGAAAGGATCATGAAGGCTCAGGCCCTGAGGGACAACTCCACCATGGGCTACATGATGGCC

AAGAAGCACCTGGAGATCAACCCTGACCACCCGATCGTGGACACCCTGAGGCAGAAGGCCGACGCAGACA

AGAATGATAAGGCTGTGAAGGACCTAGTGATCCTGCTCTTCGAGACTGCCCTCTTGTCCTCTGGGTTTTC

ACTGGATGACCCCCAGACACACTCCAACAGAATCTACCGCATGATCAAATTGGGTCTCGGTCTCGACGAG

GAGGAGGTGATTGTGGATGAGGTCACTCCACAACCTGCTGAAGAGATCCCGCCCCTGGAGGGAGACGAGG

ATGCATCACGCATGGAAGAGGTGGACTAA

>*Leucoraja*_*erinacea*_732aa

MPEEIRTDDELETFAFQAEIAQLMSLIINTFYSNKEIFLRELISNASDALDKIRYESLTDPSKMESCKDL

KIEIIPNIRERTLTVIDTGIGMTKADLINNLGTIAKSGTKAFMEALQAGADISMIGQFGVGFYSAYLVAE

KVTVFTKHNDDESYCWESSAGGSFTVKTHSGKGDAGVPDTITRGTTVILQLKEDQAEYLEEKRIKEVVKK

HSQFIGYPITLFVEKEREKEISDDEAEPEDKAEKEEGVEAEEGEKPKIEDVGSDEEEDGEKKDKKKTKKI

KEKYIDQEELNKTKPIWTRNPDDITQEEYGEFYKSLTNDWEDHLAVKHFSVEGQLEFRALLFLPRRAPFD

LFENKKKKNNIKLYVRRVFIMDSCEELIPEYLNFVRGVVDSEDLPLNISREMLQQSKILKVIRKNIVKKC

MELFGELSEDKENYKKFYEQFSKNLKLGIHEDSQNRKKLSEMLRYHTSQSGDELTPLSDYVSRMKENQKC

IYYITGESKDQVANSAFVERVRKRGFEVVYMTEPIDEYCVQQLKEFDGKQLVSVTKEGLELPEDDDEKKK

RETDRTKYDGLCKLMKEILDKKVEKVTVSNRLVSSPCCIVTSTYGWTANMERIMKAQALRDNSTMGYMMA

KKHLEINPDHPIVDTLRQKADADKNDKAVKDLVILLFETALLSSGFSLDDPQTHSNRIYRMIKLGLGLDE

EEVIVDEVTPQPAEEIPPLEGDEDASRMEEVD

**HYPEROARTIA**

>*Petromyzon_marinus*_Hsp90-1_cds_2190bp

ATGCCTGGAGAAACAACTGGTCCGACCATGGATGAAGAGACTGAGACCTTCGCCTTCCAGGCGGAGATCG

CCCAGTTGATGTCCCTCATCATTAACACCTTCTACTCCAACAAGGAAATCTTTCTCAGGGAGCTCATCTC

CAACAGCTCTGATGCTCTGGATAAGATCCGCTATGAGAGTCTGACAGACCCCAGCAAAATGGACTCTGGC

AAGGAGCTCAAGATCGACGTGATCCCCAACAGAAATGAGCGTACCCTCACCATCAGCGACACGGGCATCG

GCATGACCAAGGCTGATCTCATCAACAACTTGGGCACCATCGCCAAGTCGGGCACCAAGGCCTTCATGGA

GGCGCTGCAAGCGGGGGCCGACATCTCCATGATTGGCCAGTTCGGCGTGGGCTTCTACTCTGCCTACCTG

ATCGCCGAACGGGTGTCCGTCGTCACCAAACACAACGATGACGAACAGTACTTGTGGGAGTCATCCGCGG

GGGGTTCCTTCACGGTGCGCCTTGACAATGGTGAAAGCATTGGCAGGGGCACTAAGATCGTGCTGCACCT

GAAGGAGGACCAATCTGAGTACCTGGAGGAGAAGCGCATCAAAGACATCGTGAAGAAGCACTCTCAGTTC

ATCGGGTATCCCATCACCCTCTTTGTCGAGAAGGAACGCGACAAGGAGGTGAGTGACGATGAGGCCGAGG

AGGAGAAGGAGGAAAAGAAAGAGGAAGAAAAGGAGGCCGAGACAGATGACGACAAGCCCAAGATTGAGGA

TGTTGGCTCGGACGACGAGGCCGACAAGGAGGGTGACAAGGACAAGAAGAAGAAAAAGAAGATCAAGGAG

AAGTACGTCGACAAGGAGGAGCTCAACAAGACCAAGCCTCTGTGGACACGCAATCCAGACGACATCTCGC

AGGAGGAGTATGGAGAGTTCTACAAGAGTCTCACCAACGATTGGGAGGATCACTTGGCTGTCAAGCACTT

CTCCGTGGAGGGCCAGCTAGAGTTCCGGGCCCTGCTGTTCGTGCCACGCCGTGCGCCCTTCGACCTCTTT

GAGAACAAGAAGAAAAAGAACAACATCAAGCTGTACGTGCGCCGTGTGTTCATCATGGACAACTGCGAAG

ACCTCATCCCTGAGTACCTCAACTTCATCAAAGGTGTGGTGGACTCGGAAGACCTCCCCCTGAACATCTC

GCGAGAGATGCTCCAGCAGAGCAAGATCCTCAAGGTCATCCGCAAGAACATCGTGAAGAAGTGCATGGAG

CTGTTTACAGGCCTTGCTGAAGACAAAGAAAACTACAAGAAGTTCTACGAGCAGTTTTCCAAGAACATGA

AGCTGGGAATCCACGAGGACTCTCAGAATCGCAAGCGTCTTTCCGAAATGCTGCGGTACCACACGTCGGT

GACGGGCGAGGATGTGTGCTCCCTCAAGGAGTACGTGTCACGCATGAAGGAGAACCAGAAGCACATCTAC

TACATCACTGGCGAGTCCAAAGAACAGGTGTCGAACTCTGCATTTGTGGAGCGCGTGCGCAAGCGGGGCC

TGGAGGTGGTGTACATGACGGAGCCCATCGACGAGTACTGCGTGCAGCAGCTCAAGGAGTTTGACGGCAA

AACGCTCGTCTCGGTCACCAAGGAGGGGCTGGAACTGCCTGAGGATGAGGACGAGAAGAAGAAGCAGGAA

GAGGCTAAAGCGAAGTACGAAAACCTCTGCAAGGTGGTCAAGGAGATCCTGGACAAGAAGGTGGAGAAGG

TGACCGTTTCCAACCGGCTGGTGGCCTCTCCATGCTGCATCGTGACCAGCACTTACGGCTGGACGGCCAA

CATGGAGCGAATCATGAAGGCACAGGCTCTTCGTGACTCGTCCACCATGGGCTACATGGCCGCCAAGAAG

CACTTGGAGATCAACCCAGAACACCCCATCGTGGAGACGCTGCGCCAGAAGGCGGAGGCAGACAAGAACG

ACAAGTCTGTCAAGGACCTCATCATTCTGCTCTTCGAGACGGCCCTGCTGTCTTCGGGCTTCTCGCTCGA

TGACCCCCAGACGCACTCCAACCGCATCTACCGCATGATCAAGCTGGGGCTCGGTATTGATGATGACGAA

CCCATACCAGATGAGAGCACGCCAACTGTCACTGAGGAGATGCCACCACTGGAAGGAGAGGATGCTTCCC

GGATGGAAGAGGTTGATTAA

>*Petromyzon_marinus*_Hsp90-1_729aa

MPGETTGPTMDEETETFAFQAEIAQLMSLIINTFYSNKEIFLRELISNSSDALDKIRYESLTDPSKMDSG

KELKIDVIPNRNERTLTISDTGIGMTKADLINNLGTIAKSGTKAFMEALQAGADISMIGQFGVGFYSAYL

IAERVSVVTKHNDDEQYLWESSAGGSFTVRLDNGESIGRGTKIVLHLKEDQSEYLEEKRIKDIVKKHSQF

IGYPITLFVEKERDKEVSDDEAEEEKEEKKEEEKEAETDDDKPKIEDVGSDDEADKEGDKDKKKKKKIKE

KYVDKEELNKTKPLWTRNPDDISQEEYGEFYKSLTNDWEDHLAVKHFSVEGQLEFRALLFVPRRAPFDLF

ENKKKKNNIKLYVRRVFIMDNCEDLIPEYLNFIKGVVDSEDLPLNISREMLQQSKILKVIRKNIVKKCME

LFTGLAEDKENYKKFYEQFSKNMKLGIHEDSQNRKRLSEMLRYHTSVTGEDVCSLKEYVSRMKENQKHIY

YITGESKEQVSNSAFVERVRKRGLEVVYMTEPIDEYCVQQLKEFDGKTLVSVTKEGLELPEDEDEKKKQE

EAKAKYENLCKVVKEILDKKVEKVTVSNRLVASPCCIVTSTYGWTANMERIMKAQALRDSSTMGYMAAKK

HLEINPEHPIVETLRQKAEADKNDKSVKDLIILLFETALLSSGFSLDDPQTHSNRIYRMIKLGLGIDDDE

PIPDESTPTVTEEMPPLEGEDASRMEEVD

>*Petromyzon_marinus*_Hsp90-2_cds_1833bp

ATGCCCGAGGTGTCGGAGACGACGCAGCCCATGGCGGGCAGTGGCGAGGAGGCGGAGACCTTCGCCTTCC

AGGCGGAGATCGCCCAGCTCATGTCCCTCATCATCAACACCTTCTACTCCAACAAGGAGATCTTCCTGCG

CGAGCTCATCTCCAACAGCTCCGACGCGCTGGACAAGATCCGCTATGAGAGCCTCACTGACCCCAGCAAG

CTGGACACTGGCAAGGAGCTCAAGATCGACATCATCCCCAATAAGATCGACCGCACACTCACACTCGTCG

ACACGGGCATCGGCATGACCAAGGCCGACCTCGTCAACAACCTAGGCACCATCGCCAAGTCGGGCACCAA

AGCCTTCATGGAGGCCCTGCAGGCGGGCGCCGACATCTCGATGATCGGCCAGTTCGGCGTGGGCTTCTAT

TCTGCCTACCTGGTGGCCGAGCGCGTCACCGTCGTCACCAAGCACAACGATGACGAGCAATATGTGTGGG

AGTCTTCGGCCGGCGGCTCGTTTACCGTGAGGCCCGACAACGACGGGGAGCCTCTCGGTCGTGGCACCAA

GATCGTGCTCCACATTAAGGAGGACCAGACTGAGTACCTGGAGGAGAGGTCAATTAAGGACATTGTCAAG

AAGCACTCCCAGTTCATCGGCTACCCCATCACGCTTTACGTGGAGAAAGAACGCAACAAGGAGGTGAGCG

ATGACGAGGAGGACAAGGAGGAGGCGGCTCCTGATAAACCTAAGGAAGAGAGGTCTGACGACGACACTCC

CAACATCGAAGACGTAGGCTCCGATGAGGAGGAGGACAAGGCCAAGGACTCGGACAAGAAGAAAAAGAAG

AAGAAGATCAAGGAGAAGTACACGGACAAGGAGGAGCTGAACAAGACAAAGCCGCTGTGGACGCGCAACC

CGGACGACATCACGGCGGAGGAGTATGGCGAGTTCTACAAGAGCCTCACCAACGACTGGGAGGACCACCT

TGCCGTGAAGCACTTCTCGGTGGAGGGGCAGCTGGAATTCCGGGCGCTGCTCTTCGTCCCCCGGCGGGCG

GCTTTCGACCTCTTTGAAAACAAGAAGAAGAAAAACAACATCAAGCTGTACGTTCGACGTGTCTTCATCA

TGGACAATTGTGAAGACCTCATCCCGGAGTATCTCAACTTCATGAAGGGAGTGGTGGACTCCGAGGACCT

TCCCCTCAACATCTCGCGTGAGATGCTGCAGCAGAGCAAAATCCTCAAGGTGATCCGCAAGAACATCGTC

AAGAAATCCATGGAGCTGTTCGCGGAGCTCGCCGAGGACAAAGATAACTACAAGAAGTTCTATGAGCAGT

TCTCCAAAAACATCAAGCTGGGAATCCACGAGGACTCTCAGAACCGGAAGAAGCTAGCGGAGCTGCTCCG

CTACCATACGTCCATGTCGGGCGAGGACATGTGCTCGCTGAAGGACTACGTGTCTCGAATGAAAGAGAAC

CAGAAGCACATCTACTACATCACCGGCGAGTCAAAAGCGCAAGTGGCCAACTCTGCGTTTGTGGAGCGCG

TGCGCAAGCGTGGCCTGGAGGTGGTGTACATGGTGGAGCCCATCGATGAGTACTGCGTGCAGCAGCTGAA

GGAGTTTGACGGGAAGACCCTCGTCTCCGTCACCAAGGAGGGCATGGAGCTTCCGGAAGATGAGGAAGAG

AAAAAGAGACAGGAGGAGGCCAAGGCCAAGTTTGAGAATCTCTGCAAGGTGGTCAAGGAGATCCTGGAGA

AGAAGGTGGAGAAGGTGACCGTGTCAAATAGGCTGGTCTCCTCTCCGTGCTGCATCGTGACCAGCACCTA

CGGCTGGACGGCC

>*Petromyzon_marinus*_Hsp90-2_611aa

MPEVSETTQPMAGSGEEAETFAFQAEIAQLMSLIINTFYSNKEIFLRELISNSSDALDKIRYESLTDPSK

LDTGKELKIDIIPNKIDRTLTLVDTGIGMTKADLVNNLGTIAKSGTKAFMEALQAGADISMIGQFGVGFY

SAYLVAERVTVVTKHNDDEQYVWESSAGGSFTVRPDNDGEPLGRGTKIVLHIKEDQTEYLEERSIKDIVK

KHSQFIGYPITLYVEKERNKEVSDDEEDKEEAAPDKPKEERSDDDTPNIEDVGSDEEEDKAKDSDKKKKK

KKIKEKYTDKEELNKTKPLWTRNPDDITAEEYGEFYKSLTNDWEDHLAVKHFSVEGQLEFRALLFVPRRA

AFDLFENKKKKNNIKLYVRRVFIMDNCEDLIPEYLNFMKGVVDSEDLPLNISREMLQQSKILKVIRKNIV

KKSMELFAELAEDKDNYKKFYEQFSKNIKLGIHEDSQNRKKLAELLRYHTSMSGEDMCSLKDYVSRMKEN

QKHIYYITGESKAQVANSAFVERVRKRGLEVVYMVEPIDEYCVQQLKEFDGKTLVSVTKEGMELPEDEEE

KKRQEEAKAKFENLCKVVKEILEKKVEKVTVSNRLVSSPCCIVTSTYGWTA

**ACTINOPTERYGII**

>*Tetraodon_nigroviridis*_AB1_cds_2151bp

ATGCCTGAAGAAATGCACCAAGAGGCGGATGTTGAGACCTTTGCCTTCCAGGCAGAGATTGCTCAGATGT

CCCTCATCATCAACACCTTTTATTCCAACAAAGAGATCTTCCTCAGGGAGTTGATCTCCAATTCCTCTGA

TGCCTTGGACAAAATTCGCTATGAAAGTCTGACTGACCCCTCTAAGCTAGACAGTGGCAAGGAACTTAAA

ATTGACATCATTCCTGACAAAGAGGAGAACACCTTGACCATCATTGACACTGGAATCGGCATGACCAAAG

CTGACCTTATCAACAACCTGGGTACCATTGCCAAGTCTGGCACCAAGGCCTTCATGGAGGCATTACAGGC

CGGAGCCGATATTTCCATGATTGGCCAGTTTGGTGTGGGATTCTACTCTGCGTACCTTGTTGCTGAGAAG

GTGAAGGTTATCACCAAACACAATGATGATGAACAGTATGTCTGGGAATCCTCTGCCGGAGGTTCATTTA

CAGTCAGGACCGACAATGATGCACCAATTGGCCGTGGAACAAAGATCATTCTGTACCTGAAGGAGGACCA

GACTGAGTACTACGAGGAGAAGAGGATCAAGGAAATTGTCAAGAAGCACTCTCAGTTTATTGGCTACCCC

ATCACCCTATATGTGGAGAAGGAACGTGACAAGGAGGTCAGTGATGATGAAGCAGAGGAGGAAAAAGAAG

AGCAAGAGGAGGATAAGGATGCCCCCACGATTGAGGACCTTGGTTCTGAAGAAGAATCCAAAGACAAGAA

GAAGAAAAAGAAGATTAAGGAAAGGTACACCGACCAGGAAGAGCTGAACAAAACTAAGCCTATTTGGACC

AGGAACCCTGATGACATCACAAACGAGGAGTATGGCGAGTTTTACAAAAGCCTGACCAATGATTGGGAGG

AGCATCTGGCGGTCAAGCACTTTTCTGTGGAGGGCCAACTTGAATTCAGAGCACTGCTCTTTATTCCTCG

CCGTGCACCCTTTGACCTCTTTGAGAACAAGAAAAAGAAGAACAACATCAAGCTCTATGTCAGAAGGGTC

TTTATCATGGACAACTGTGAAGACCTTATCCCAGAATACCTCAACTTCATCCGTGGTGTCGTGGACTCCG

AGGACCTGCCTCTCAACATCTCTAGAGAGATGCTGCAGCAGAGCAAAATCCTGAAGGTCATTCGCAAAAA

CATTGTCAAGAAGTGTCTTGAGCTGTTTGCTGAACTGGCAGAGGACAAGGACAATTACAAAAAGTTCTAT

GAAGCCTTCTCCAAGAACATCAAGCTTGGCATCCACGAGGATTCACAAAATCGCAAGAAGCTGTCGGAGT

TGTTGCGCTACCACAGCTCTCAATCTGGAGATGAGGTGACCTCCCTCACAGAGTACATCACTCGCATGAA

GGAAAACCAGAAATCCATCTACTACATCACTGGCGAGAGCAAAGACCAGGTTGCCAACTCGGCCTTCGTT

GAGCGCGTCCGCAAGCGTGGTTTCGAGGTCCTGTATATGACGGAGCCAATTGACGAGTACTGCATCCAGC

AGCTGAAGGAGTTTGACGGCAAGAACCTGGTCTCTGTCACCAAAGAGGGTCTCGAACTGCCAGAGGATGA

AGAGGAGAAAAAGAAGATGGAGGAGGATAAGGCCAAATTTGACAGCCTGTGCAAAATTATGAAAGAAATC

CTTGACAAGAAAGTGGAGAAGGTAACCGTCTCCAACAGACTGGTCTCGTCACCCTGCTGTATTGTCACAA

GCACTTACGGCTGGACAGCCAACATGGAGAGGATCATGAAGGCCCAGCTGAGGGACAACTCCACCATGGG

TTACATGATGGCCAAGAAGCACCTAGAGATCAATCCTGACCATCCTATTGTGGAGACCCTCAGACAGAAG

GCGGAAGCGGACAAGAACGACAAGGCTGTGAAAGACCTCGTCATCCTTCTGTTTGAAACCGCTCTGCTAT

CGTCAGGTTTCTCTCTGGATGACCCCCAGACTCATTCCAACCGCATCTATAGAATGATCAAACTTGGACT

GGGCATCGATGACGATGACATTCCCACAGAGGAGACGACATCTGCATCTGTACCAGATGAGATTCCTCCC

CTGGAAGGCGAGGGTGAAGATGACGCTTCACGCATGGAAGAAGTTGACTAA

>*Tetraodon_nigroviridis*_AB1_716aa

MPEEMHQEADVETFAFQAEIAQMSLIINTFYSNKEIFLRELISNSSDALDKIRYESLTDPSKLDSGKELK

IDIIPDKEENTLTIIDTGIGMTKADLINNLGTIAKSGTKAFMEALQAGADISMIGQFGVGFYSAYLVAEK

VKVITKHNDDEQYVWESSAGGSFTVRTDNDAPIGRGTKIILYLKEDQTEYYEEKRIKEIVKKHSQFIGYP

ITLYVEKERDKEVSDDEAEEEKEEQEEDKDAPTIEDLGSEEESKDKKKKKKIKERYTDQEELNKTKPIWT

RNPDDITNEEYGEFYKSLTNDWEEHLAVKHFSVEGQLEFRALLFIPRRAPFDLFENKKKKNNIKLYVRRV

FIMDNCEDLIPEYLNFIRGVVDSEDLPLNISREMLQQSKILKVIRKNIVKKCLELFAELAEDKDNYKKFY

EAFSKNIKLGIHEDSQNRKKLSELLRYHSSQSGDEVTSLTEYITRMKENQKSIYYITGESKDQVANSAFV

ERVRKRGFEVLYMTEPIDEYCIQQLKEFDGKNLVSVTKEGLELPEDEEEKKKMEEDKAKFDSLCKIMKEI

LDKKVEKVTVSNRLVSSPCCIVTSTYGWTANMERIMKAQLRDNSTMGYMMAKKHLEINPDHPIVETLRQK

AEADKNDKAVKDLVILLFETALLSSGFSLDDPQTHSNRIYRMIKLGLGIDDDDIPTEETTSASVPDEIPP

LEGEGEDDASRMEEVD

>chromosome14_AA1-1_*Tetraodon_nigroviridis*_cds_1920bp

ATGCCTGAGAAAGCTGGACATGTTGTGGAGGAGGATGTGGAGACCTTTGCATTCCAGGCCGAGATTGCTC

AGCTTATGTCTCTTATCATCAACACTTTCTACTCAAACAAAGAGATCTTCCTTAGAGAGCTTATCTCTAA

CTCCTCAGATGCTTTGGACAAAATCAGATATGAAAGTTTGACTGATCCAACCCGCCTTGAGTCCTGCAAA

GAACTGAAGATTGAAGTCAGACCTGACCTTCATGCCCGCACCCTGACCCTGATTGACACGGGTATCGGTA

TGACCAAGGCCGACCTGATCAACAACCTGGGCACCATCGCCAAGTCTGGCACCAAGGCCTTCATGGAGGC

TCTGCAGGCTGGAGCCGACATCTCCATGATCGGGCAGTTTGGCGTGGGGTTCTACTCTGCCTACCTCGTG

GCCGAGAAGGTGACAGTCATCACAAAGCACAACGATGACGAGCAGTATGTGTGGGAGTCTGCAGCCGGAG

GCTCGTTCACAGTTAAGCCTGACACCGGAGAATCGATTGGCAGAGGAACCAAAGTTATTCTCCACCTCAA

AGAAGATCAGACAGAATACTGTGAGGAAAAACGCATAAAAGAAGTTGTAAAGAAGCACTCGCAGTTCATT

GGCTACCCTATTACTCTTTTTGTGGAGAAAACCAGGGAGAAGGAAGTGGACCTGGAAGAGGGGGAGAAAG

TGGAGGAGGTTGAGAAAGAGCCGGCAGAGCCCACCGACAAACCGAAGATCGAGGACGTGGGCTCTGACGA

GGATGAGGACACAAAGGATGGCAAGAACAAGAGGAAGAAGAAGGTCAAGGAAAAGTACATGGATGTTCAG

GAGCTGAACAAGACCAAGCCTATCTGGACCCGGAACCCTGATGACATCACCAATGAGGAGTATGGAGAGT

TCTATAAAAGCCTGACCAACGACTGGGAAGACCACCTGGCTGTCAAGCACTTCTCGGTGGAAGGTCAGCT

GGAGTTCCGTGCTTTGCTTTTCGTACCCAGAAGGGCCCCATTTGACCTCTTTGAAAACAAAAAGAAGAAG

AACAACATCAAGCTGTATGTTCGCAGGGTTTTCATCATGGATAACTGCGACGAGCTGATTCCGGAGTACC

TCAATTTCATCAGGGGTGTGGTGGACTCTGAGGATCTGCCCCTGAACATCTCCAGAGAGATGCTGCAGCA

GAGCAAGATCCTGAAGGGTGAGACCAAAGAACAGGTGGCCAACTCGGCCTTCGTGGAGCGCCTCCGTAAA

GCTGGCCTGGAGGTCATTTACATGATCGAGCCCATTGACGAGTACTGCGTCCAGCAGCTGAAGGAGTACG

ATGGCAAAAACTTGGTTTCAGTAACAAAGGAAGGGCTAGAGCTGCCTGAGGATGAGGAGGAGAAGAAGAA

GCTGGAGGAGCTCAAAAACAAATTTGAAAACCTTTGCAAGATCATGAAGGACATCCTTGATAAGAAGATT

GAAAAGGTCACTGTTTCCAATCGCCTGGTGGCTTCTCCTTGTTGTATCGTCACCAGCACGTACGGCTGGA

CTGCCAACATGGAGAGGATCATGAAGTCCCAAGCCCTGAGAGACAGCTCCACCATGGGCTACATGACAGC

AAAGAAGCATCTTGAGATCAACCCTCTGCATCCGATCATTGAAACCTTGAGGGAAAAGGCTGAAGCTGAC

AAGAACGATAAGGCAGTGAAGGACCTGGTCATCCTCCTGTACGAGACTGCCCTGCTGTCTTCAGGATTCA

CTCTGGAAGATCCTCAGACACATGCCAACCGCATCTACAGAATGATCAAGCTGGGTCTAGGCATTGACGA

TGATGATTCAGCAGTTGAAGATCTTATTCAGCCCGCTGATGAAGAAATGCCAGTCTTGGAAGGAGACGAT

GACACATCTAGAATGGAAGAAGTTGACTGA

>chromosome14_AA1-1_*Tetraodon_nigroviridis*_639aa

MPEKAGHVVEEDVETFAFQAEIAQLMSLIINTFYSNKEIFLRELISNSSDALDKIRYESLTDPTRLESCK

ELKIEVRPDLHARTLTLIDTGIGMTKADLINNLGTIAKSGTKAFMEALQAGADISMIGQFGVGFYSAYLV

AEKVTVITKHNDDEQYVWESAAGGSFTVKPDTGESIGRGTKVILHLKEDQTEYCEEKRIKEVVKKHSQFI

GYPITLFVEKTREKEVDLEEGEKVEEVEKEPAEPTDKPKIEDVGSDEDEDTKDGKNKRKKKVKEKYMDVQ

ELNKTKPIWTRNPDDITNEEYGEFYKSLTNDWEDHLAVKHFSVEGQLEFRALLFVPRRAPFDLFENKKKK

NNIKLYVRRVFIMDNCDELIPEYLNFIRGVVDSEDLPLNISREMLQQSKILKGETKEQVANSAFVERLRK

AGLEVIYMIEPIDEYCVQQLKEYDGKNLVSVTKEGLELPEDEEEKKKLEELKNKFENLCKIMKDILDKKI

EKVTVSNRLVASPCCIVTSTYGWTANMERIMKSQALRDSSTMGYMTAKKHLEINPLHPIIETLREKAEAD

KNDKAVKDLVILLYETALLSSGFTLEDPQTHANRIYRMIKLGLGIDDDDSAVEDLIQPADEEMPVLEGDD

DTSRMEEVD

>chromosome14_AA1-2_*Tetraodon_nigroviridis*_cds_1728bp

ATGCCTGAACCCCATGACCTTCAAATGGAGGAGGAGGCTGAGACCTTTGCCTTTCAGGCTGAGATCGCTC

AGCTGATGTCCTTGATCATCAACACTTTCTATTCAAACAAAGAGATCTTCCTGAGGGAGTTGATCTCCAA

CTCTTCAGATGCACTTGACAAAATCCGCTATGAGAGCCTCACTGACCCCTCCAAGCTGGACAACGGCAAA

GACCTCAAAATTGAACTGAAGCCCAACAAAGAGGACCGCACCCTGACCCTGATTGACACGGGTATCGGTA

TGACCAAGGCCGACCTGATCAACAACCTGGGCACCATCGCCAAGTCTGGCACCAAGGCCTTCATGGAGGC

TCTGCAGGCTGGAGCCGACATCTCCATGATCGGGCAGTTTGGCGTGGGGTTCTACTCTGCCTACCTCGTG

GCCGAGAAGGTGACAGTCATCACAAAGCACAACGATGACGAGCAGTACGCCTGGGAATCATCGGCCGGAG

GCTCGTTCACCGTCCGAGTGGACAACNNNNNNNNNNNNNNNNNNNNNNNNNNNNNNNNNNNNNNNNNNNN

NNNNNNNNNNNNNNNNNNNNNNNNNNNNNNNNNNNNNNNNNNNNNNNNNNNNNNNNGTGGAAGGTAGGAT

GGATTTAAGAGAAATAATTTTTGTACCTCGGCGAGCCCCTTTTGACCTCTTTGAGAATAAGAAAAAGAAG

AACAACATTAAGCTGTATGTAAGAAGGGTTTTCATCATGGACAACTGTGAGGATCTCATCCCAGAGTACC

TAAATTTTATTAAGGGTGTGGTGGACTCTGAGGATCTGCCCCTGAACATCTCCAGAGAGATGCTGCAGCA

GAGCAAGATCCTGAAGGTGATCCGCAAGAACCTGGTCAAGAAGTGCCTGGAGCTCTTCACTGAGCTGGCG

GAGGACAAGGACAACTACAAAAAATACTACGAGCAGTTTTCCAAGAACATCAAGCTGGGCATCCATGAGG

ATTCTCAAAACAGAAAGAAGCTGTCTGAGCTGCTGAGATACTACACCTCAGCCTCTGGAGATGAGATGGT

GTCTCTGAAGGACTACGTTACACGCATGAAGGACACCCAGAAACACATCTACTACATCACTGGCGAGACC

AAAGACCAGGTGGCCAACTCGGCCTTCGTGGAGCGCCTCCGTAAAGCTGGCCTGGAGGTCATTTACATGA

TCGAGCCCATTGACGAGTACTGCGTCCAGCAGCTGAAGGAGTTTGAAGGCAAGAACCTGGTTTCAGTGAC

CAAGGAGGGTCTGGAGCTGCCTGAGGATGAGGAGGAGAAGAAGAATCAGGAGGAGAAGAAGGCTCAGTTT

GAGAACCTGTGCAAGATCATGAAGGACATCCTGGAGAAGAAGGTGGAAAAGGTCACAGTCTCCAACCGCT

TGGTGTCCTCCCCCTGCTGCATCGTCACCAGCACGTACGGCTGGACGGCCAACATGGAGAGGATCATGAA

GGCCCAGGCCCTGAGGGACAACTCCACCATGGGCTACATGGCAGCTAAGAAGCACCTTGAGATCAACCCT

GACCACCCGATTATGCAGACTTTGAGGCAGAAGGCGGAGGCCGATAAGAACGACAAGTCGGTGAAGGATC

TGGTCATCCTGCTTTTCGAGACCGCGCTGCTGTCCTCGGGGTTCACCCTGGACGACCCTCAGACTCACTC

CAACCGCATCTACAGAATGATCAAGCTGGGTCTTGGCATTGACGAAGATGACGTGACACCAGAGGAGTCC

ACAGCCGCTCCCACAGAGGACATGCCTCCTCTCGAAGGGGATGATGACGACACATCCAGGATGGAGGAGG

TTGACTAA

>chromosome14_AA1-2_*Tetraodon_nigroviridis*_575aa

MPEPHDLQMEEEAETFAFQAEIAQLMSLIINTFYSNKEIFLRELISNSSDALDKIRYESLTDPSKLDNGK

DLKIELKPNKEDRTLTLIDTGIGMTKADLINNLGTIAKSGTKAFMEALQAGADISMIGQFGVGFYSAYLV

AEKVTVITKHNDDEQYAWESSAGGSFTVRVDNVEGRMDLREIIFVPRRAPFDLFENKKKKNNIKLYVRRV

FIMDNCEDLIPEYLNFIKGVVDSEDLPLNISREMLQQSKILKVIRKNLVKKCLELFTELAEDKDNYKKYY

EQFSKNIKLGIHEDSQNRKKLSELLRYYTSASGDEMVSLKDYVTRMKDTQKHIYYITGETKDQVANSAFV

ERLRKAGLEVIYMIEPIDEYCVQQLKEFEGKNLVSVTKEGLELPEDEEEKKNQEEKKAQFENLCKIMKDI

LEKKVEKVTVSNRLVSSPCCIVTSTYGWTANMERIMKAQALRDNSTMGYMAAKKHLEINPDHPIMQTLRQ

KAEADKNDKSVKDLVILLFETALLSSGFTLDDPQTHSNRIYRMIKLGLGIDEDDVTPEESTAAPTEDMPP

LEGDDDDTSRMEEVD

**TUNICATA**

>*Ciona_intestinalis*_cds_2184bp

ATGGAACAGGAACACGAAGTTCAAGAGGACTCTGGGGAAGTCTTCGCTTTCCAGGCAGAGATTGCACAGT

TGATGAGCTTGATCATCAACACTTTCTACAGCAACAAAGAAATCTTTCTTCGAGAATTGATTTCTAATGC

ATCAGATGCCTTGGACAAGATCCGATATGAAAGCTTGACAGATCCCAGCAAACTTGACAGTGGCAAGGAA

TTGAAAATTGAAATTATCCCAAACAAAGACGATTACACCCTTACACTTATTGACACTGGTGTGGGAATGA

CAAAATCTGACCTAATTAACAACCTTGGTACAATCGCAAAATCTGGTACCAAAGCATTCATGGAGGCTCT

TCAGTCTGGAGCTGACATTTCCATGATTGGTCAATTTGGTGTTGGTTTCTACTCTGCTTTCTTGGTCGCT

GACAAAGTGCGAGTGGTTTCCAAGCATAACGATGATGAAGCATATGCATGGGAATCCTCTGCTGGTGGTT

CTTTCACCGTTGCTTCCATGGATGGAGATACTTTAACCCGTGGAACAAAGATTGTTCTTCATCTTAAGGA

AGATCAACGGGAGTACTTGGAGGAAAAGAAGATCAAGGACATCGTGAAGAAGCACAGTCAGTTCATCGGG

TATCCCATCTCACTTCATGTGGAGAAAGAGAGAGAAAAGGAAATCAGTGATGATGAGGAAGATGAAGAAG

AGAAAAAAGAAGAAACTGCTGAAACCGAAGAACAAAAGGAAGATGACGACAAACCTAAAGTAGAAGATCT

TGATGAAGAAGGTGAGGAAGCAAAGGATGACAAGAAGAAGACAAAGAAGATCAAGGAAAAATACACCGAA

CTTGAAGAGTTGAACAAAACTAAACCATTGTGGACACGAAACCCTGATGATATCTCCCAAGATGAATATG

GGGAGTTCTATAAAAGTTTATCAAATGATTGGGAAGATCATCTTGCTGTGAAGCATTTCTCAGTGGAAGG

TCAACTAGAGTTCCGAGCCTTGTTGTTCGTACCAAAGCGTGCTCCATTCGATTTATTCGAGAACCGAAAA

ATGAAGAATAATATTAAGTTGTACGTAAGGAGAGTTTTCATCATGGACAACTGTGAGGATCTTATCCCAG

AATACTTGAACTTCGTTAAAGGAGTGGTCGACAGTGAAGATCTTCCCTTGAACATCTCTCGTGAAACTCT

CCAACAATCCAAGATCCTCAAGGTGATCAGGAAGAACATCGTGAAGAAGTGCGTGGAGTTGATTGGTGAG

ATTGCCGAGGACAAAGAGCAATACAAGAAGTTCTACGAGCAATTCGGGAAGAACCTTAAGCTTGGAATCC

ACGAAGATTCACAAAACCGAAAGAAGTTGGCCGGGTTCCTTCAATATCACTCATCACAATCAGGTGACGA

GATGACATCGTTGGAAAATTACGTCACTCGTATGAAGGAAAACCAGAAGGATATTTATTATATCACTGGT

GAATCAAGAGACCAGGTTTCTAACTCTGCTTTTGTTGAAAGAGTTACGAAACGTGGGTTCGAAGTCCTTT

ATATGGTTGAACCAATTGATGAATACTGTGTACAACAACTTAAGGAATTCGATGGCAAGAACTTGGTTTC

GATCACAAAGGAAGGTCTTGAACTTCCTGAAGATGAAGATGACAAGAAGAAGTTCGAAGAAGCAAAAGCA

AAGTTCGAAAATCTTTGCAAAGTCGTCAAGGAAATTCTTGACAAGAAAGTGGAAAAGGTTGTTGTTTCGA

ACCGTCTTGTACAATCCCCTTGCTGTATTGTGACATCACAATATGGTTGGTCAGCAAACATGGAGAGGAT

CATGAAGGCTCAAGCACTTCGCGATACATCAACCATGGGCTACATGGCAGCGAAGAAACATTTAGAAATC

AACCCAGACCATTCTATTATGGAACAACTCCGACAAAAAGCTGAAGCTGATAAGAATGACAAATCTGTGA

AAGATCTCGTCATGCTGTTGTACGAAACCTCCCTGCTTGCTTCGGGATTCTCCTTGGAAGATCCATCAAC

ACACGCCACTCGTATTCATAGAATGATCAAACTTGGTTTGGGCATTGATGAAGTTGATGGTGAAGAGACG

ACAGCTGCCGAGGAAATCGATGACATGCCACCATTAGAGGGCGATGGAGATGATGATGCATCGAGAATGG

AAGAAGTTGATTAA

>*Ciona_intestinalis*_727aa

MEQEHEVQEDSGEVFAFQAEIAQLMSLIINTFYSNKEIFLRELISNASDALDKIRYESLTDPSKLDSGKE

LKIEIIPNKDDYTLTLIDTGVGMTKSDLINNLGTIAKSGTKAFMEALQSGADISMIGQFGVGFYSAFLVA

DKVRVVSKHNDDEAYAWESSAGGSFTVASMDGDTLTRGTKIVLHLKEDQREYLEEKKIKDIVKKHSQFIG

YPISLHVEKEREKEISDDEEDEEEKKEETAETEEQKEDDDKPKVEDLDEEGEEAKDDKKKTKKIKEKYTE

LEELNKTKPLWTRNPDDISQDEYGEFYKSLSNDWEDHLAVKHFSVEGQLEFRALLFVPKRAPFDLFENRK

MKNNIKLYVRRVFIMDNCEDLIPEYLNFVKGVVDSEDLPLNISRETLQQSKILKVIRKNIVKKCVELIGE

IAEDKEQYKKFYEQFGKNLKLGIHEDSQNRKKLAGFLQYHSSQSGDEMTSLENYVTRMKENQKDIYYITG

ESRDQVSNSAFVERVTKRGFEVLYMVEPIDEYCVQQLKEFDGKNLVSITKEGLELPEDEDDKKKFEEAKA

KFENLCKVVKEILDKKVEKVVVSNRLVQSPCCIVTSQYGWSANMERIMKAQALRDTSTMGYMAAKKHLEI

NPDHSIMEQLRQKAEADKNDKSVKDLVMLLYETSLLASGFSLEDPSTHATRIHRMIKLGLGIDEVDGEET

TAAEEIDDMPPLEGDGDDDASRMEEVD

>*Ciona_savignyi_*cds_2175bp

ATGGCGGATGAAGTTCAAGATGATGTTGCGGAGGTGTTTGCTTTCCAAGCTGAAATTGCTCAGTTAATGA

GCTTGATTATCAATACCTTCTACAGCAACAAAGAAATCTTCTTAAGAGAATTGATTTCAAACTCTTCTGA

TGCCCTCGATAAAATTCGGTATGAAAGTTTAACCGATCCAAGCAAGCTTGACAGCGGAAAAGAATTGAAA

ATTGAAATCATCCCAAATAAGGATGACCATACCCTCACCTTGATTGATACTGGTGTAGGAATGACAAAAT

CTGATCTGATCAACAATCTAGGGACAATTGCAAAATCTGGAACAAAAGCTTTTATGGAAGCTCTTCAGTC

GGGTGCTGATATTTCTATGATTGGTCAATTTGGTGTCGGTTTCTACTCTGCATTTTTGGTAGCAGACAAA

GTTAAAGTGGTTTCAAAGCACAACGATGATGAACAATATGTGTGGGAATCTTCCGCTGGTGGCTCCTTTA

CAGTTTCCAATGTAGTTGATGATGTTCCAGTCCGTGGTACCAAGATCGTTCTTTATATGAAGGAAGATCA

ACGCGAGTACTTGGAGGAAAAGAAGATCAAGGACATCGTTAAGAAGCACAGCCAGTTTATTGGTTATCCA

ATCTCCCTTCATGTGGAGAAGGAGCGGGAAAAGGAAATCAGTGATGACGAGGAGGAGGAAGAAGAGAAGA

AAGAGGAAGCTGCCGAGGAGGTTCAGCAGGAGAATGACGACAAGCCAAAAGTAGAAGACCTCGATGAAGA

AAATGAGGAAGCGAAGGACGACAAAAAGAAAAAGAAGAAAATTAAGGAGAAATACACTGAGCTTGAAGAG

CTGAACAAAACGAAGCCACTGTGGACCAGAAACCCTGATGATATTTCACAAGATGAATACGGAGAGTTCT

ACAAAAGTTTGTCTAATGACTGGGAAGACCATCTTGCTGTTAAGCACTTCTCTGTAGAAGGACAGTTGGA

GTTCCGTGCCTTGCTCTTTGTTCCAAAGCGTGCTCCTTTTGACTTGTTCGAAAATCGGAAGCTTAAGAAC

AACATTAAGCTTTATGTCAGGAGAGTATTCATCATGGACAACTGTGAGGATCTCATTCCAGAGTACCTAA

ATTTCGTGAAGGGAGTGGTGGATAGTGAAGATCTTCCTTTAAACATCTCTCGTGAAACTCTTCAACAATC

AAAGATTCTCAAAGTCATTCGAAAGAACATTGTAAAGAAATGTGTTGAATTAATTGGCGAGATTGTAGAA

GACAAGGAACAATACAAGAAATTTTATGAACAATTCGGAAAGAACCTGAAGCTGGGTATTCATGAGGATT

CTCAAAATCGCAAGAAATTGGCTGGATTCCTTCAATACCACTCATCGCAATCTGGTGATGAAATGACAAC

TTTGGAAAACTACGTCACTCGAATGAAGGAAAACCAGAAGGACATTTATTACATTACTGGTGAATCCAGA

GACCAGGTCTCCAACTCTGCTTTTGTTGAAAGAGTCACAAAAAGAGGGTTTGAAGTCTTGTACATGGTTG

AGCCCATTGATGAATATTGTGTACAACAACTCAAGGAGTTCGATGGGAAGAATTTGGTCTCCATTACAAA

AGAAGGTCTTGAACTTCCAGAGGATGAAGATGAGAAGAAAAAGTTGGAAGAAGCAAAGGCTAAATTTGAA

AACCTTTGCAAAGTTATCAAGGAGATTCTTGACAAAAAAGTGGAGAAGGTTGTGGTATCAAATAGACTGG

TGCAGTCCCCGTGTTGTATTGTGACCTCCCAGTATGGTTGGTCTGCCAACATGGAACGAATCATGAAAGC

CCAAGCATTAAGAGACACCTCCACTATGGGTTATATGGCCGCTAAGAAACACCTGGAAATTAATCCAGAT

CATTCAATCATGGAGCAACTTCGACAAAAGGCAGAGGCCGACAAGAATGACAAGTCTGTAAAAGATCTCG

TTATGCTTTTGTATGAAACTTCCCTGCTGGCTTCTGGCTTCTCACTGGAAGATCCATCAACCCATGCCAC

CCGAATTCATAGAATGATCAAGCTCGGTTTAGGTATTGATGATGTTGATGGAGAGGACGCAGCCACCGGC

GACGATGTTGATGATATGCCGCCACTGGAGGGTGATGCTGATGACGATGCATCTCGCATGGAAGAAGTTG

ATTAA

>*Ciona_savignyi_*724aa

MADEVQDDVAEVFAFQAEIAQLMSLIINTFYSNKEIFLRELISNSSDALDKIRYESLTDPSKLDSGKELK

IEIIPNKDDHTLTLIDTGVGMTKSDLINNLGTIAKSGTKAFMEALQSGADISMIGQFGVGFYSAFLVADK

VKVVSKHNDDEQYVWESSAGGSFTVSNVVDDVPVRGTKIVLYMKEDQREYLEEKKIKDIVKKHSQFIGYP

ISLHVEKEREKEISDDEEEEEEKKEEAAEEVQQENDDKPKVEDLDEENEEAKDDKKKKKKIKEKYTELEE

LNKTKPLWTRNPDDISQDEYGEFYKSLSNDWEDHLAVKHFSVEGQLEFRALLFVPKRAPFDLFENRKLKN

NIKLYVRRVFIMDNCEDLIPEYLNFVKGVVDSEDLPLNISRETLQQSKILKVIRKNIVKKCVELIGEIVE

DKEQYKKFYEQFGKNLKLGIHEDSQNRKKLAGFLQYHSSQSGDEMTTLENYVTRMKENQKDIYYITGESR

DQVSNSAFVERVTKRGFEVLYMVEPIDEYCVQQLKEFDGKNLVSITKEGLELPEDEDEKKKLEEAKAKFE

NLCKVIKEILDKKVEKVVVSNRLVQSPCCIVTSQYGWSANMERIMKAQALRDTSTMGYMAAKKHLEINPD

HSIMEQLRQKAEADKNDKSVKDLVMLLYETSLLASGFSLEDPSTHATRIHRMIKLGLGIDDVDGEDAATG

DDVDDMPPLEGDADDDASRMEEVD
