# Supplementary figures and images for: Assessment and Reconstruction of Novel HSP90 Genes: Duplications, Gains and Losses in Fungal and Animal Lineages
Source: PLoS One. 2013 Sep 16;8(9):e73217. doi: 10.1371/journal.pone.0073217 (PMC3774752; doi:10.1371/journal.pone.0073217)

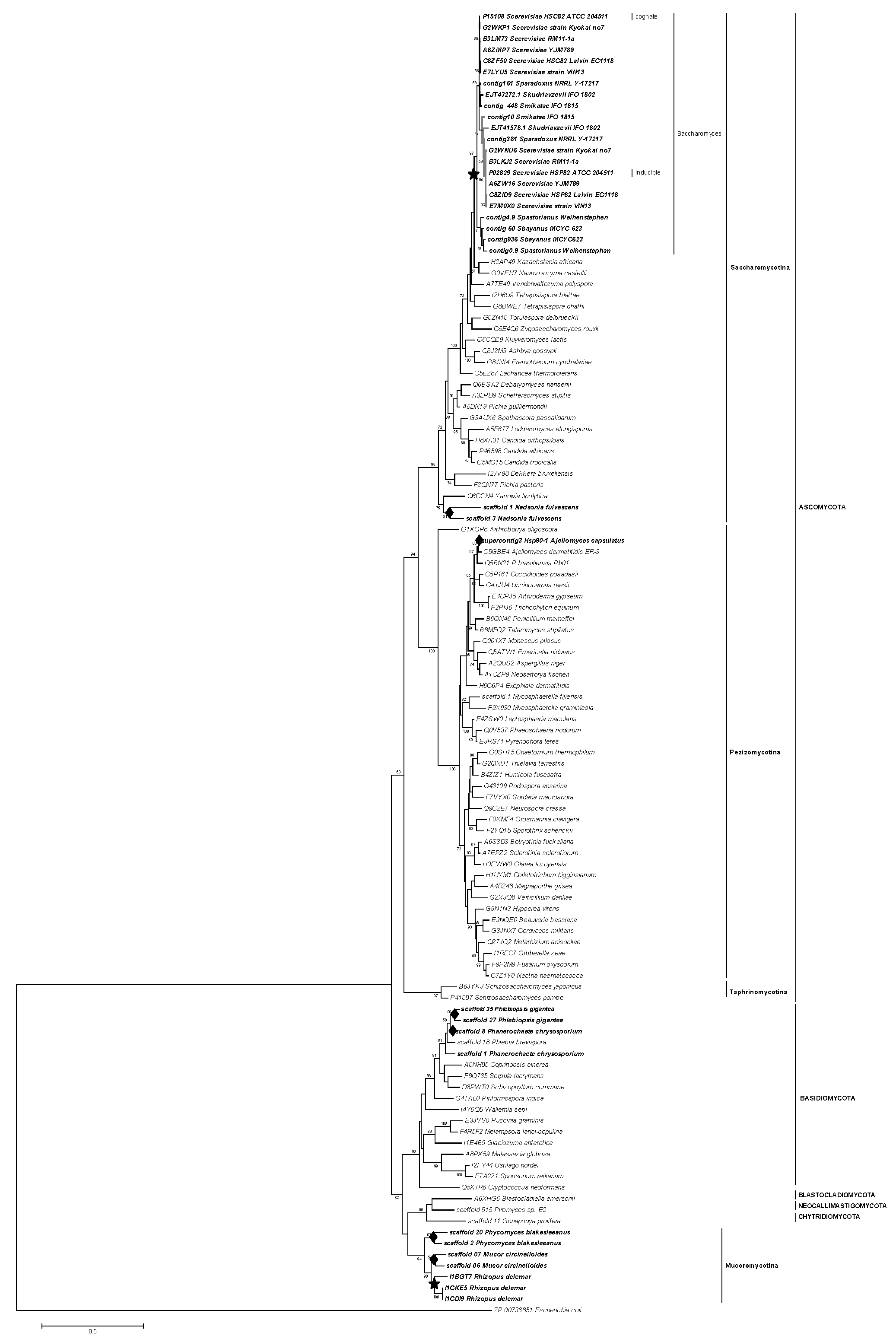

Supplement: Figure S2 — ML tree using Hsp90 protein sequences from Fungi. Species in which multiple hsp90 genes have been detected are in bold and italics. Filled diamonds denote putative species-specific duplication events, predicted by this study. Stars represent whole-genome duplications reported by previous studies. Numbers represent bootstrap values (percentages); values below 50% are not shown. (TIFF) [file pone.0073217.s002.tiff]

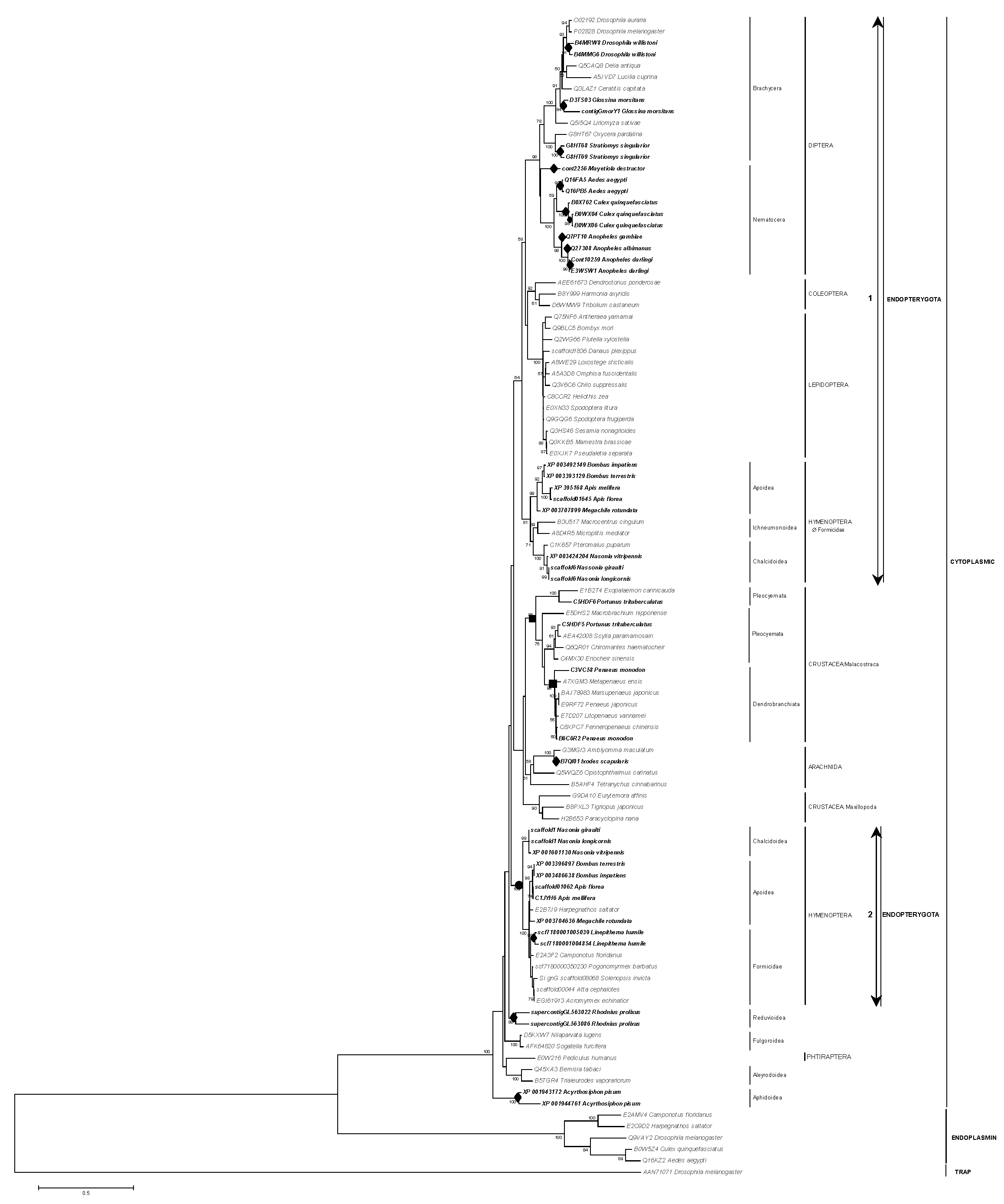

Supplement: Figure S3 — ML tree using Hsp90 protein sequences from Arthropoda. Species in which multiple hsp90 genes have been detected are in bold and italics. Filled diamonds denote putative species-specific duplication events, predicted by this study. Filled squares denote duplication events in the common ancestor of a wide taxonomic group (e.g.Pleocyemata), predicted by this study. Filled circle shows the gain of type 2 isoform in Hymenoptera; empty-strikethrough circle shows loss of type 1 isoform in Formicidae. Numbers represent bootstrap values (percentages); values below 50% are not shown. (TIF) [file pone.0073217.s003.tif]

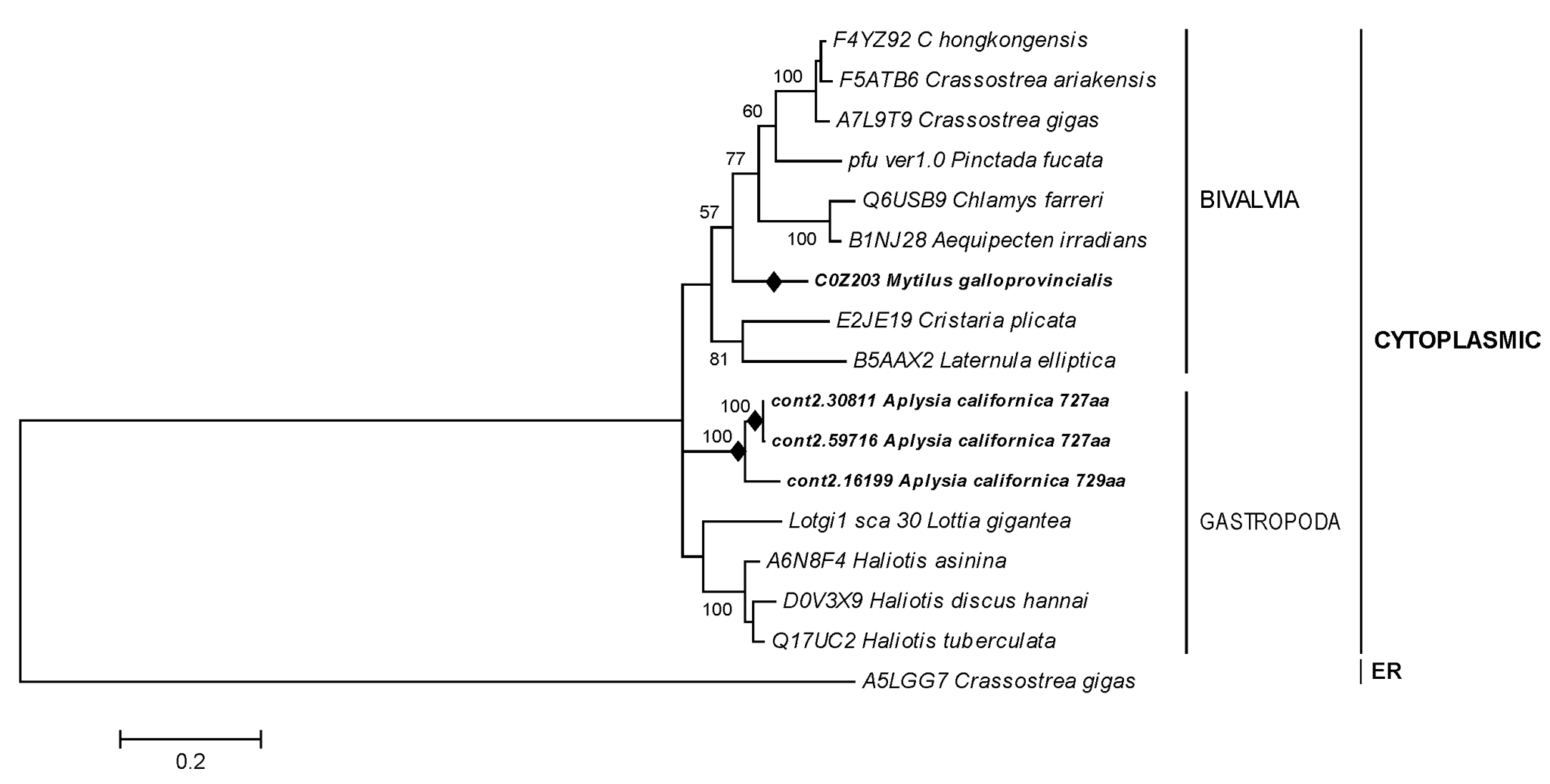

Supplement: Figure S4 — ML tree using Hsp90 protein sequences from Mollusca. Species in which multiple hsp90 genes have been detected are in bold and italics. Filled diamonds denote putative species-specific duplication events, predicted by this study. Numbers represent bootstrap values (percentages); values below 50% are not shown. (TIFF) [file pone.0073217.s004.tiff]

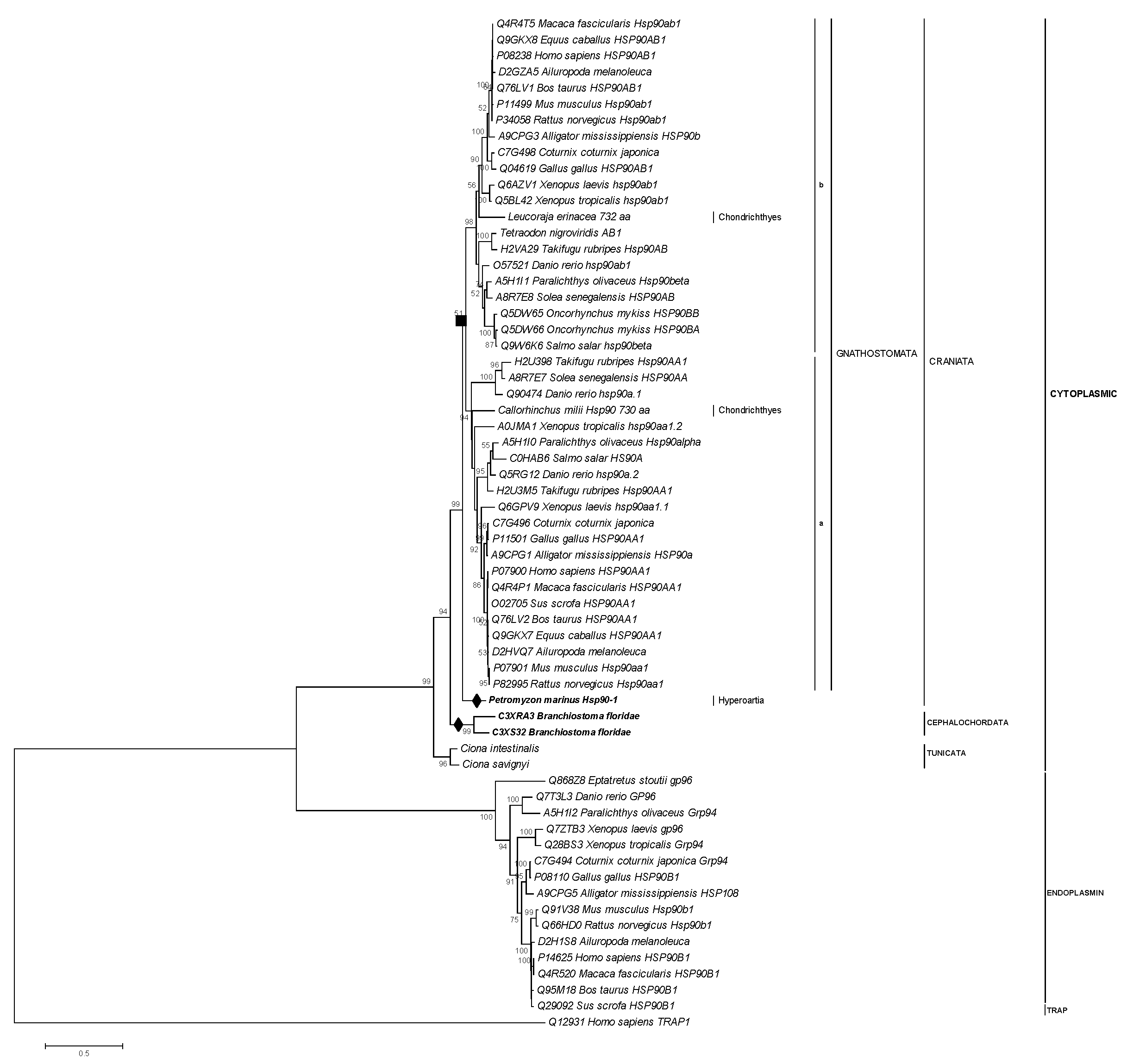

Supplement: Figure S5 — ML trees using Hsp90 protein sequences from Chordata. Species in which multiple hsp90 genes have been detected are in bold and italics. Filled diamonds denote putative species-specific duplication events, predicted by this study. Filled square denotes the duplication event resulting in the cognate and inducible isoforms of vertebrates. Numbers represent bootstrap values (percentages); values below 50% are not shown. (TIF) [file pone.0073217.s005.tif]
