# Supplementary material for: Assessment and Reconstruction of Novel HSP90 Genes: Duplications, Gains and Losses in Fungal and Animal Lineages
Source: PLoS One. 2013 Sep 16;8(9):e73217. doi: 10.1371/journal.pone.0073217 (PMC3774752; doi:10.1371/journal.pone.0073217)
Supplement: Table S1 — Species for which complete genomes were analyzed in this study, databases through which they were assessed and derived Hsp90 cytoplasmic sequences. (DOC) [file pone.0073217.s006.doc]

| **Species^1^** | | | **Database** | | **Genome Accession number – Name^2^** | | | **UniProtKB/NCBI AC – Protein Name^3^** | | **Size (aa)** | | |  |
| --- | --- | --- | --- | --- | --- | --- | --- | --- | --- | --- | --- | --- | --- |
| **FUNGI** | |  | | | |  |  | |  | | |  | |
| *Ajellomyces capsulatus*^⊕^ | | | BROAD Institute | | supercont2.3 (c2463392-2465687) | | | **supercontig3­_Hsp90-1** | | 702 | | |  |
|  | | |  | | supercont2.3 (c2457877-2459901) | | | **supercontig3­_Hsp90-2** | | 612 | | |  |
| *Gonapodya prolifera*^⊕^ | | | JGI | | scaffold_11 (c445008-447865) | | | **scaffold_11** | | 705 | | |  |
| *Mucor circinelloides*^⊕^ | | | JGI | | scaffold_06 (c712694-714858) | | | **scaffold_06** | | 699 | | |  |
|  | | |  | | scaffold_07 (823097-825334) | | | **scaffold_07** | | 700 | | |  |
| *Mycosphaerella fijiensis*^⊕^ | | | JGI | | scaffold_1 (c2547490-2549598) | | | **scaffold_1** | | 702 | | |  |
| *Nadsonia fulvescens*^⊕^ | | | JGI | | scaffold_1 (c2364883-2366970) | | | **scaffold_1** | | 695 | | |  |
|  | | |  | | scaffold_3 (207519-209627) | | | **scaffold_3** | | 702 | | |  |
| *Phanerochaete chrysosporium*^⊕^ | | | JGI | | scaffold_1 (c1105934-1108318) | | | **scaffold_1** | | 701 | | |  |
|  | | |  | | scaffold_8 (c838491-840801) | | | **scaffold_8** | | 699 | | |  |
| *Phlebia brevispora*^⊕^ | | | JGI | | scaffold_18 (c81714-84037) | | | **scaffold_18** | | 699 | | |  |
| *Phlebiopsis gigantea*^⊕^ | | | JGI | | scaffold_27 (c98305-100662) | | | **scaffold_27** | | 697 | | |  |
|  | | |  | | scaffold_35 (c51806-54600) | | | **scaffold_35** | | 699 | | |  |
| *Phycomyces blakesleeanus*^⊕^ | | | JGI | | scaffold_2 (2408563-2410843) | | | **scaffold_2** | | 704 | | |  |
|  | | |  | | scaffold_20 (993376-995722) | | | **scaffold_20** | | 697 | | |  |
| *Piromyces sp. E2*^⊕^ | | | JGI | | scaffold_515 (c373-2607) | | | **scaffold_515** | | 696 | | |  |
| *Rhizopus delemar*⯎ | | | BROAD Institute - GenBank (WGS) | | CH476743 | | | I1CKE5 | | 696 | | |  |
|  | | |  | | CH476732 | | | I1BGT7 | | 698 | | |  |
|  | | |  | | CH476740 | | | I1CDI9 | | 696 | | |  |
| *Saccharomyces bayanus*^⊕^ | | | GenBank (WGS) | | AACA01000308.1 (3863-5984) | | | **contig_60** | | 706 | | |  |
|  | | |  | | AACA01000157.1 (15581-17707) | | | **contig_936** | | 708 | | |  |
| *Saccharomyces kudriavzevii*⯎ | | | GenBank (WGS) | | AACI03000916.1 | | | EJT43272.1 | | 711 | | |  |
|  | | |  | | AACI03002002.1 | | | EJT41578.1 | | 710 | | |  |
| *Saccharomyces mikatae*^⊕^ | | | GenBank (WGS) | | AABZ01000096.1 (c5455-7575) | | | **contig_10** | | 706 | | |  |
|  | | |  | | AABZ01000258.1 (3334-5466) | | | **contig_448** | | 710 | | |  |
| *Saccharomyces paradoxus*^⊕^ | | | GenBank (WGS) | | AABY01000043.1 (c19082-21214) | | | **contig_161** | | 710 | | |  |
|  | | |  | | AABY01000006.1 (c33336-35468) | | | **contig_381** | | 710 | | |  |
| *Saccharomyces_pastorianus*^⊕^ | | | GenBank (WGS) | | ABPO01000013.1 (42368-44506) | | | **conti0.9** | | 712 | | |  |
|  | | |  | | ABPO01000177.1 (c16342-18465) | | | **conti4.9** | | 707 | | |  |
| **ARTHROPODS** | |  | | | |  |  | |  | | |  | |
| *Acromyrmex echinatior*⯎ | | | FlyBase | | GL888384 | | | EGI61913.1 | | 724 | | |  |
| *Acyrthosiphon pisum*⯎ | | | AphidBase | | NW_003383617.1 | | | XP_001943172.1 | | 728 | | |  |
|  | | |  | | NW_003383576 | | | XP_001944761.2 | | 759 | | |  |
| *Aedes aegypti*⯎ | | | VectorBase | | NW_001810125.1 | | | XP_001649751.1 | | 560 | | |  |
|  | | |  | | NW_001810125.1 | | | XP_001649752.1 (Q16FA5) | | 715 | | |  |
|  | | |  | | NW_001811357.1 | | | XP_001655641.1 (Q16PB5) | | 715 | | |  |
|  | | |  | | NW_001811357.1 | | | XP_001655642.1 | | 715 | | |  |
| *Anopheles darlingi*^◆^ | | | FlyBase | | ADMH01001383.1 | | | E3WSW1 | | 724 | | |  |
|  | | |  | | ADMH01001383.1 (4256-4612) (end of contig) | | | **Cont10747** | | 114 | | |  |
|  | | |  | | ADMH01001209.1 (92-2257) | | | **Cont10259** | | 721 | | |  |
|  | | |  | | ADMH01002361.1 (c66848-67200) (end of contig) | | | **Cont13917** | | 117 | | |  |
| *Anopheles gambiae*^◆^ | | | VectorBase | | 2L | | | Q7PT10 | | 720 | | |  |
|  | | |  | | 2L (40261180-40263218) (contains Ns) | | | **2L-b** | | 397 | | |  |
|  | | |  | | 2L (40269303-40271223) (contains Ns) | | | **2L-c** | | 383 | | |  |
| *Apis florea*^⊕^ | | | FlyBase | | GL576665.1 (178813-181543) | | | **scaffold01645** | | 754 | | |  |
|  | | |  | | GL576082.1 (4229156-4231733) | | | **scaffold01062** | | 724 | | |  |
| *Apis melifera*⯎ | | | GenBank (WGS) | | NW_003378067.1 | | | C1JYH6 | | 724 | | |  |
|  | | |  | | NW_003378177.1 | | | XP_395168 | | 755 | | |  |
| *Atta cephalotes*^⊕^ | | | FlyBase | | GL377381.1 (c330339-333777) | | | **scaffold00044** | | 724 | | |  |
| *Bombus impatiens*⯎ | | | FlyBase | | JH158117.1 | | | XP_003492149 | | 717 | | |  |
|  | | |  | | JH157828.1 | | | XP_003486638 | | 725 | | |  |
| *Bombus terrestris*⯎ | | | GenBank (WGS) | | NW_003565351 | | | XP_003396897.1 | | 725 | | |  |
|  | | |  | | NW_003565319 | | | XP_003393129.1 | | 717 | | |  |
| *Camponotus floridanus*⯎ | | | FlyBase | | GL436428.1 | | | E2A3F2 | | 722 | | |  |
| *Culex quinquefasciatus*^◆^ | | | VectorBase | | NW_001887042.1 | | | B0WX04 | | 716 | | |  |
|  | | |  | | NW_001887042.1 | | | B0WX06 | | 716 | | |  |
|  | | |  | | NW_001887042.1 (352252-352467) | | | **Cont3.343** | | 72 (partial) | | |  |
|  | | |  | | NW_001887327.1 | | | B0X762 | | 719 | | |  |
| *Danaus plexippus*^⊕^ | | | FlyBase | | JH383022.1 (c14532-16688) | | | **scaffold1836** | | 718 | | |  |
| *Drosophila willistoni*⯎ | | | FlyBase | | NW_002032468.1 | | | **B4MMG6** | | 721 | | |  |
|  | | |  | | NW_002032471.1 | | | B4MRW8 | | 716 | | |  |
| *Glossina morsitans*^◆^ | | | VectorBase | | GmorY1:scf7180000649147 (64696-66843) | | | **contigGmorY1** | | 715 | | |  |
|  | | |  | | GmorY1:scf7180000648077 | | | D3TS03 | | 716 | | |  |
| *Harpegnathos saltator*⯎ | | | FlyBase | | GL446181.1 | | | E2B7J9 | | 723 | | |  |
| *Ixodes scapularis*^◆^ | | | VectorBase (GenBank) | | NW_002835969.1 | | | B7QI01 | | 731 | | |  |
|  | | |  | | NW_002835969.1 (20939-21565) | | | **DS942764** | | 209 (partial) | | |  |
| *Linepithema humile*^⊕^ | | | FlyBase | | GL905166.1 (87272-89840) | | | **scf7180001004854** | | 723 | | |  |
|  | | |  | | GL905344.1 (c82003-84567) | | | **scf7180001005039** | | 723 | | |  |
| *Mayetiola destructor*^⊕^ | | | FlyBase GenBank (WGS) | | AEGA01002255.1 (c15771-18220) | | | **Cont2256** | | 714 | | |  |
|  | | |  | | AEGA01022387.1 (c3982-4494) | | | **scaffold_Un.16375** | | 171 (partial) | | |  |
| *Megachile rotundata*⯎ | | | FlyBase | | NW_003797713.1 | | | XP_003707899 | | 718 | | |  |
|  | | |  | | NW_003797206.1 | | | XP_003704636 | | 722 | | |  |
| *Nasonia giraulti*^⊕^ | | | FlyBase | | GL277768.1 (334733-337415) (contains Ns) | | | **scaffold1** | | 631 | | |  |
|  | | |  | | GL276173.1 (1790097-1792268) | | | **scaffold6** | | 723 | | |  |
| *Nasonia longicornis*^⊕^ | | | FlyBase | | GL277950.1 (334339-337024) (contains Ns) | | | **scaffold1** | | 631 | | |  |
|  | | |  | | GL277955.1 (1790261-1792432) | | | **scaffold6** | | 723 | | |  |
| *Nasonia vitripennis*⯎ | | | FlyBase | | NW_001820416 | | | XP_003424204 | | 723 | | |  |
|  | | |  | | NW_001815682 (contains Ns) | | | XP_001601130 | | 648 | | |  |
| *Pediculus humanus*⯎ | | | VectorBase | | NW_002987878.1 | | | E0W216 | | 725 | | |  |
| *Pogonomyrmex barbatus*^⊕^ | | | Flybase | | GL738449.1 (c83901-86834) | | | **scf7180000350230** | | 722 | | |  |
| *Rhodnius prolixus*^⊕^ | | | VectorBase | | GL563022 (1285989-1288807) | | | **supercontigGL563022** | | 727 | | |  |
|  | | |  | | GL563086 (591654-596055) | | | **supercontigGL563086** | | 723 | | |  |
| *Solenopsis invicta*^⊕^ | | | FlyBase | | GL769399.1 (606438-609467) | | | **scaffold08068** | | 723 | | |  |
| *Tribolium castaneum*⯎ | | | GenBank | | NW_001092837.1 | | | D6WMW9 | | 721 | | |  |
| **MOLLUSCA** | |  | | | |  |  | |  | | |  | |
| *Aplysia californica*^⊕^ | | | GenBank (WGS) | | AASC02016120.1 (c33066-42939) | | | **cont2.16199** | | 729 | | |  |
|  | | |  | | AASC02030812.1 (8447-19413) | | | **cont2.30811** | | 727 | | |  |
|  | | |  | | AASC02059717.1 (c2608-12922) | | | **cont2.59716** | | 727 | | |  |
| *Crassostrea gigas*⯎ | | | GenBank | | AFTI01002014.1 | | | A7L9T9 | | 717 | | |  |
| *Lottia gigantea*^⊕^ | | | JGI | | Lotgi1 sca_30 (511323-516103) | | | **Lotgi1 sca_30** | | 724 | | |  |
| *Pinctada fucata*^⊕^ | | | [OIST Marine Genomics Unit](http://www.irp.oist.jp/satoh/) | | scaffolds 182423,522320, 264369, 178011, 70523, 294616, 286028, 68915 | | | **pfu_ver1.0** | | 725 | | |  |
| **CHONDRICTHYES** | | |  | |  | | |  | |  | | |  |
| *Callorhinchus milii*^⊕^ | | | Elephant shark genome sequencing Project | | AAVX01059957, AAVX01221079, AAVX01308810 | | | ***Callorhinchus_milii_*Hsp90** | | 730 | | |  |
| **HYPEROARTIA** | |  | | | |  |  | |  | | |  | |
| *Petromyzon marinus*^⊕^ | | | Ensembl- GenBank (ESTs) | | GL479675 (8602-15195) | | | ***Petromyzon marinus hsp90-1*** | | 729 | | |  |
|  | | |  | | GL498392 (1114-2768) | | | ***Petromyzon marinus hsp90-2*** | | 611 | | |  |
| **ACTINOPTERYGII** | |  | | | |  |  | |  | | |  | |
| *Takifugu rubripes*⯎ | | | FUGU Genome Project | | Chromosome 14 | | | H2U3M5 | | 727 | | |  |
|  | | |  | | Chromosome 14 | | | H2U398 | | 724 | | |  |
|  | | |  | | Chromosome 14 | | | H2VA29 | | 723 | | |  |
| *Tetraodon nigroviridis*^⊕^ | | | Ensembl | | 14 dna:chromosome (c2462375-2465792) | | | ***Tetraodon_nigroviridis*_AB1** | | 716 | | |  |
|  | | |  | | 14 dna:chromosome (c7048749-7051386) | | | **chromosome14_AA1-1** | | 644 | | |  |
|  | | |  | | 14 dna:chromosome (c7043843-7046584) | | | **chromosome14_AA1-2** | | 564 | | |  |
| **CEPHALOCHORDATA** | |  | | | |  |  | |  | | |  | |
| *Branchiostoma floridae*⯎ | | | JGI | | scaffold_Bf_V2_6 | | | C3XRA3 | | 725 | | |  |
|  | | |  | | scaffold_Bf_V2_6 | | | C3XS32 | | 731 | | |  |
| **TUNICATA** |  | | |  | | |  | |  | |  | | |
| *Ciona intestinalis*^⊕^ | | | Ensembl | | scaffold_435 (37203-44711) | | | ***Ciona intestinalis*** | | 727 | | |  |
| *Ciona savignyi*^⊕^ | | | Ensembl | | reftig_11 (207998-211953) | | | ***Ciona savignyi* Hsp90** | | 724 | | |  |

**^1^** Species for which genome analysis in the present study revealed new (⊕) or additional (◆) copies or verified (⯎) existing data

**^2^** Numbers in brackets denote positions of translation start and stop codons for sequences predicted in the present study

**^3^** In bold the names attributed to Hsp90s predicted in this study
