# Supplementary material for: Assessment and Reconstruction of Novel HSP90 Genes: Duplications, Gains and Losses in Fungal and Animal Lineages
Source: PLoS One. 2013 Sep 16;8(9):e73217. doi: 10.1371/journal.pone.0073217 (PMC3774752; doi:10.1371/journal.pone.0073217)
Supplement: Table S2 — The species for which Hsp90s annotated in UniProt/TrEMBL were used in this study. (DOC) [file pone.0073217.s007.doc]

| **Species** | **UniProt- TrEMBL Accession number** |
| --- | --- |
| **FUNGI** |  |
| *Ajellomyces dermatitidis ER-3* | C5GBE4 |
| *Arthrobotrys oligospora* | G1XGP8 |
| *Arthroderma gypseum* | E4UPJ5 |
| *Ashbya gossypii* | Q8J2M3 |
| *Aspergillus niger* | A2QUS2 |
| *Beauveria bassiana* | E9NQE0 |
| *Blastocladiella emersonii* | A6XHG6 |
| *Botryotinia fuckeliana* | A6S3D3 |
| *Candida albicans* | P46598 |
| *Candida orthopsilosis* | H8XA31 |
| *Candida tropicalis* | C5MG15 |
| *Chaetomium thermophilum* | G0SH15 |
| *Coccidioides posadasii* | C5P161 |
| *Colletotrichum higginsianum* | H1UYM1 |
| *Coprinopsis cinerea* | A8NH85 |
| *Cordyceps militaris* | G3JNX7 |
| *Cryptococcus neoformans* | Q5K7R6 |
| *Debaryomyces hansenii* | Q6BSA2 |
| *Dekkera bruxellensis* | I2JV98 |
| *Emericella nidulans* | Q5ATW1 |
| *Eremothecium cymbalariae* | G8JNI4 |
| *Exophiala dermatitidis* | H6C6P4 |
| *Fusarium oxysporum* | F9F2M9 |
| *Gibberella zeae* | I1REC7 |
| *Glaciozyma antarctica* | I1E4B9 |
| *Glarea lozoyensis* | H0EWW0 |
| *Grosmannia clavigera* | F0XMF4 |
| *Humicola fuscoatra* | B4ZIZ1 |
| *Hypocrea virens* | G9N1N3 |
| *Kazachstania africana* | H2AP49 |
| *Kluyveromyces lactis* | Q6CQZ9 |
| *Lachancea thermotolerans* | C5E287 |
| *Leptosphaeria maculans* | E4ZSW0 |
| *Lodderomyces elongisporus* | A5E677 |
| *Magnaporthe grisea* | A4R248 |
| *Malassezia globosa* | A8PX59 |
| *Melampsora larici-populina* | F4R5F2 |
| *Metarhizium anisopliae* | Q27JQ2 |
| *Monascus pilosus* | Q001X7 |
| *Mycosphaerella graminicola* | F9X930 |
| *Naumovozyma castellii* | G0VEH7 |
| *Nectria haematococca* | C7Z1Y0 |
| *Neosartorya fischeri* | A1CZP9 |
| *Neurospora crassa* | Q9C2E7 |
| *Paracoccidioides brasiliensis Pb01* | Q5BN21 |
| *Penicillium marneffei* | B6QN46 |
| *Phaeosphaeria nodorum* | Q0V537 |
| *Pichia guilliermondii* | A5DN19 |
| *Pichia pastoris* | F2QN77 |
| *Piriformospora indica* | G4TAL0 |
| *Podospora anserina* | O43109 |
| *Puccinia graminis* | E3JVS0 |
| *Pyrenophora teres* | E3RS71 |
| *Saccharomyces cerevisiae HSC82 ATCC 204511* | P15108, P02829 |
| *Saccharomyces cerevisiae HSC82 Lalvin EC1118* | C8ZF50, C8ZID9 |
| *Saccharomyces cerevisiae RM11-1a* | B3LM73, B3LKJ2 |
| *Saccharomyces cerevisiae strain Kyokai no7* | G2WKP1, G2WNU6 |
| *Saccharomyces cerevisiae strain VIN13* | E7LYU5, E7M0X0 |
| *Saccharomyces cerevisiae YJM789* | A6ZMP7, A6ZW16 |
| *Scheffersomyces stipitis* | A3LPD9 |
| *Schizophyllum commune* | D8PWT0 |
| *Schizosaccharomyces japonicus* | B6JYK3 |
| *Schizosaccharomyces pombe* | P41887 |
| *Sclerotinia sclerotiorum* | A7EPZ2 |
| *Serpula lacrymans* | F8Q735 |
| *Sordaria macrospora* | F7VYX0 |
| *Spathaspora passalidarum* | G3AUX6 |
| *Sporisorium reilianum* | E7A221 |
| *Sporothrix schenckii* | F2YQ15 |
| *Talaromyces stipitatus* | B8MFQ2 |
| *Tetrapisispora blattae* | I2H6U9 |
| *Tetrapisispora phaffii* | G8BWE7 |
| *Thielavia terrestris* | G2QXU1 |
| *Torulaspora delbrueckii* | G8ZN18 |
| *Trichophyton equinum* | F2PIJ6 |
| *Uncinocarpus reesii* | C4JJU4 |
| *Ustilago hordei* | I2FY44 |
| *Vanderwaltozyma polyspora* | A7TE49 |
| *Verticillium dahliae* | G2X3Q8 |
| *Wallemia sebi* | I4Y6Q5 |
| *Yarrowia lipolytica* | Q6CCN4 |
| *Zygosaccharomyces rouxii* | C5E4Q6 |
| **ARTHROPODA** |  |
| *Aedes aegypti* | Q16KZ2 |
| *Amblyomma maculatum* | G3MGI3 |
| *Anopheles albimanus* | Q27308 |
| *Antheraea yamamai* | Q75NF6 |
| *Bemisia tabaci* | Q45XA3 |
| *Bombyx mori* | Q9BLC5 |
| *Camponotus floridanus* | E2AMV4 |
| *Ceratitis capitata* | Q3LAZ1 |
| *Chilo suppressalis* | Q3V6C6 |
| *Chiromantes haematocheir* | Q6QR01 |
| *Culex quinquefasciatus* | B0W5Z4 |
| *Delia antiqua* | Q5CAQ8 |
| *Dendroctonus ponderosae* | AEE61673 |
| *Drosophila auraria* | O02192 |
| *Drosophila melanogaster* | P02828, Q9VAY2, AAN71071 |
| *Eriocheir sinensis* | C4MX30 |
| *Eurytemora affinis* | G9DA10 |
| *Exopalaemon carinicauda* | E1B2T4 |
| *Fenneropenaeus chinensis* | C8XPC7 |
| *Harmonia axyridis* | B8Y999 |
| *Harpegnathos saltator* | E2C9D2 |
| *Heliothis zea* | C8CCR2 |
| *Liriomyza sativae* | Q5I5Q4 |
| *Litopenaeus vannamei* | E7D207 |
| *Loxostege sticticalis* | A8WE29 |
| *Lucilia cuprina* | A5JVD7 |
| *Macrobrachium nipponense* | E5DHS2 |
| *Macrocentrus cingulum* | B3U517 |
| *Mamestra brassicae* | Q0KKB5 |
| *Marsupenaeus japonicus* | BAJ78983 |
| *Metapenaeus ensis* | A7XGM3 |
| *Microplitis mediator* | A8D4R5 |
| *Nilaparvata lugens* | D5KXW7 |
| *Omphisa fuscidentalis* | A5A3D8 |
| *Opistophthalmus carinatus* | Q5WQZ6 |
| *Oxycera pardalina* | G8HT67 |
| *Paracyclopina nana* | H2B653 |
| *Penaeus japonicus* | E9RF72 |
| *Penaeus monodon* | C3VC58, B6C6R2 |
| *Plutella xylostella* | Q2WG66 |
| *Portunus trituberculatus* | C5HDF5, C5HDF6 |
| *Pseudaletia separata* | E0XJK7 |
| *Pteromalus puparum* | C1K657 |
| *Scylla paramamosain* | AEA42008 |
| *Sesamia nonagrioides* | Q3HS46 |
| *Sogatella furcifera* | AFK64820 |
| *Spodoptera frugiperda* | Q9GQG6 |
| *Spodoptera litura* | E0XN33 |
| *Stratiomys singularior* | G8HT68, G8HT69 |
| *Tetranychus cinnabarinus* | B5AHF4 |
| *Tigriopus japonicus* | B8PXL3 |
| *Trialeurodes vaporariorum* | B5TGR4 |
| **MOLLUSCA** |  |
| *Aequipecten irradians* | *B1NJ28* |
| *Chlamys farreri* | *Q6USB9* |
| *Crassostrea ariakensis* | *F5ATB6* |
| *Crassostrea gigas* | *A5LGG7* |
| *Crassostrea hongkongensis* | *F4YZ92* |
| *Cristaria plicata* | *E2JE19* |
| *Haliotis asinina* | *A6N8F4* |
| *Haliotis discus hannai* | *D0V3X9* |
| *Haliotis tuberculata* | *Q17UC2* |
| *Laternula elliptica* | *B5AAX2* |
| *Mytilus galloprovincialis* | *C0Z203* |
| **CHORDATE** |  |
| *Ailuropoda melanoleuca* | D2GZA5, D2HVQ7, D2H1S8 |
| *Alligator mississippiensis* | A9CPG5, A9CPG1, A9CPG3 |
| *Bos taurus* | Q76LV2, Q76LV1, Q95M18 |
| *Coturnix coturnix japonica* | C7G494, C7G498, C7G496 |
| *Danio rerio* | Q7T3L3, Q90474, Q5RG12, O57521 |
| *Eptatretus stoutii* | Q868Z8 |
| *Equus caballus* | Q9GKX7, Q9GKX8 |
| *Gallus gallus* | P11501, Q04619, P08110 |
| *Homo sapiens* | P07900, P08238, P14625, Q12931 |
| *Macaca fascicularis* | Q4R4P1, Q4R4T5, Q4R520 |
| *Mus musculus* | P07901, P11499, Q91V38 |
| *Oncorhynchus mykiss* | Q5DW65, Q5DW66 |
| *Paralichthys olivaceus* | A5H1I0, A5H1I1, A5H1I2 |
| *Rattus norvegicus* | P34058, P82995, Q66HD0 |
| *Salmo salar* | C0HAB6, Q9W6K6 |
| *Solea senegalensis* | A8R7E7, A8R7E8 |
| *Sus scrofa* | O02705, Q29092 |
| *Xenopus laevis* | Q7ZTB3, Q6GPV9, Q6AZV1 |
| *Xenopus tropicalis* | Q28BS3, B5DFP8, A0JMA1, Q5BL42 |
