# Supplementary material for: Assessment and Reconstruction of Novel HSP90 Genes: Duplications, Gains and Losses in Fungal and Animal Lineages
Source: PLoS One. 2013 Sep 16;8(9):e73217. doi: 10.1371/journal.pone.0073217 (PMC3774752; doi:10.1371/journal.pone.0073217)
Supplement: Table S3 — Molluscan ESTs bearing hsp90 sequences, analyzed in the present study. (DOC) [file pone.0073217.s008.doc]

| ***Species*** | ***Library*** | ***Accession number*** |
| --- | --- | --- |
| *Euprymna scolopes* | UI-S-GN0, UI-S-GN1, UI-S-GG0, UI-S-GG1, UI-S-GU0, UI-S-GU1, UI-S-GB0, UI-S-GB1, UI-S-HH0 | DW264174, DW265647, DW268934, DW261386, DW275268, DW281177, DW276234, DW279785, DW286567, DW251337, DW254584, DW253738, DW284163, DW284034, DW283008 |
| *Idiosepius paradoxus* | Northern pygmy squid cDNA library | DB913228, DB917181, DB917520, DB917665, DB913128, DB913362, DB914148 |
| *Lottia gigantea* | LIBEST_022382 CAXY *Lottia gigantea* from male gonad | FC703816, FC714948, FC712038, FC704990, FC711551, FC714947, FC704989, FC712390, FC712389 |
| *Mytilus californianus* | LIBEST_021155 SHGC-MUS | ES395131, ES392943, ES391224, ES392727, ES389024, ES392172, ES395272, ES393257, ES391727, ES396919, ES391071, ES387799, ES390151, ES387630, ES396755, ES389331, ES396259, ES388834 |
