# Supplementary material for: Assessment and Reconstruction of Novel HSP90 Genes: Duplications, Gains and Losses in Fungal and Animal Lineages
Source: PLoS One. 2013 Sep 16;8(9):e73217. doi: 10.1371/journal.pone.0073217 (PMC3774752; doi:10.1371/journal.pone.0073217)
Supplement: Table S4 — Petromyzon marinus, Callorhinchus milii and Leucoraja erinacea ESTs bearing hsp90 sequences, analyzed in the present study. (DOC) [file pone.0073217.s009.doc]

| ***Species*** | ***Protein name*** | ***Library*** | ***ESTs Accession numbers*** |
| --- | --- | --- | --- |
| ***Leucoraja erinacea*** | ***Leucoraja erinacea_732*** | **LIBEST_020422 Little skate embryo tissues** | ΕΕ991897, ΕΕ992480, ΕΕ988982, ΕΕ988855, ΕΕ989473, ΕΕ989918, ΕΕ988996, ΕΕ988852, ΕΕ988325, ΕΕ993011, ΕΕ988130, ΕΕ990422 |
|  |  | **LIBEST_015890 Little Skate Multiple Tissues, Normalized** | CO050635, FL592642, CV547312 |
|  |  | **LIBEST_022984 Little Skate embryo cell line 1 (LEE-1)** | FF597904 |
| ***Petromyzon marinus*** | **Hsp90-1** | **Sea lamprey LyEST** | CO543291, CO547290, CO543324, CO543455, CO544949, CO548182, CO543196, CO543157, CO550455, CO546932, CO544457, CO548671, CO545002, CO543169, CO550881, CO544679, CO544720, CO544012, CO543327, CO548506, CO546718, CO548859, CO546003, CO543551, CO550185, CO542910, CO544867, CO547489, CO549861, CO546542, CO548249, CO543029, CO547223, CO543949, CO547762, CO550962, CO542839, CO546781, CO543261, CO549651, CO547282, CO549095, CO549645, CO544366, CO545707, CO549684, CO547698, CO546407, CO547183, CO548744, CO550568, CO547162, CO544390, CO549481, CO544799, CO547302, CO543734, CO543340, CO543322, CO547817, CO544066, CO544061, CO549793, CO550870, CO546835, CO546784, CO548812, CO548696, CO550780, CO548940, CO543686, CO548570, CO550787, CO548074, CO549916, CO544931, CO547431, CO547416, CO548078, CO543781, CO549535 |
|  |  | **CAAA** | FD713393, FD722208, FD706597, FD715954, FD724951, FD726186, FD709432, FD705115, FD715590, FD707672, FD726616, FD703262, FD726919, FD713639, FD709696, FD702452, FD715290, FD704109, FD719307, FD724799, FD703583, FD721175, FD722768, FD708087, FD700527, FD726631, FD727627, FD729354, FD702507, FD721272, FD719819, FD702985, FD722918, FD717680, FD708879, FD706981, FD722701, FD701317, FD715688, FD726769, FD704834, FD721847, FD723056, FD711366, FD721270, FD719080, FD703904, FD710973, FD705457, FD703791, FD714935, FD724681, FD715505, FD713563, FD706125, FD712758, FD727984, FD718858, FD717672, FD708625, FD701432, FD701871, FD713189, FD713249, FD702547, FD717025, FD703571, FD717586, FD725546, FD727927, FD718626, FD716975, FD722784, FD723205, FD714464, FD701378, FD701606, FD728972, FD719734, FD725839, FD721838, FD700920, FD707814, FD705347, FD708179, FD716646, FD713473, FD705080, FD716208 |
|  |  | **Lamprey WGS pCMV-sport6 Petromyzon marinus** | DW021392, DW021267, DW022686, DW021610, DW021557, DW021435, DW022697, DW021615, DW021247, DW021726, DW021575, DW022688 |
|  |  | **Lamprey EST Olfactory** | EC384095, ΕΕ740259, ΕΕ739056, ΕΕ739178, ΕΒ083820, ΕΕ740346, DY252681, DY250907, DY253442, ΕΕ742655 |
|  |  | **Lamprey EST Tissues** | DY798518, EG023829, EG024045, DY795878 |
|  | **Hsp90-2** | **Sea lamprey LyEST** | CO544202, CO544214, CO551452, CO544170 |
|  |  | **CAAA** | FD729281, FD711547 |
|  |  | **Lamprey_WGS_pCMV-sport6 *Petromyzon marinus*** | DW022503 |
| ***Callorhinchus milii*** | **Hsp90_730aa** | **Elephant shark full-length cDNA library from testis** | JK883774, JK958785, JK961363, JK960813, JK960862, JK959537, JK958669, JK962804, JK960615, JK961825, JK962005, JK960081, JK962667, JK962953, JK960807, JK964574, JK957188 |
|  |  | **Elephant shark full-length cDNA library from gills** | JK928188, JK927993, JK928442, JK928428, JK928494, JK931366, JK890709 |
|  |  | **Elephant shark full-length cDNA library from kidney** | JK940794, JK940329, JK942094, JK940583, JK942735, JK942534, JK937824 |
|  |  | **Elephant shark full-length cDNA library from spleen** | JK953247 |
|  | **Hsp90**  **(Type 2)** | **Elephant shark full-length cDNA library from spleen** | JK875294, JK877193, JK875453 |
